# Transcriptomic and proteomic analyses of *Desulfovibrio vulgaris* biofilms: Carbon and energy flow contribute to the distinct biofilm growth state

**DOI:** 10.1186/1471-2164-13-138

**Published:** 2012-04-16

**Authors:** Melinda E Clark, Zhili He, Alyssa M Redding, Marcin P Joachimiak, Jay D Keasling, Jizhong Z Zhou, Adam P Arkin, Aindrila Mukhopadhyay, Matthew W Fields

**Affiliations:** 1 Department of Microbiology, Miami University, Oxford, USA; 2 Center for Biofilm Engineering, Montana State University, Bozeman, USA; 3 Institute for Environmental Genomics, University of Oklahoma, Norman, USA; 4 Physical Biosciences Division, Lawrence Berkeley National Laboratory, Berkeley, USA; 5 Department of Chemical and Biomolecular Engineering, University of California, Berkeley, USA; 6 Department of Bioengineering, University of California, Berkeley, USA; 7 Department of Microbiology, Montana State University, Bozeman, USA; 8 Environmental Networks Integrated with Molecular Assemblies (http://enigma.lbl.gov/), Bozeman, USA

## Abstract

**Background:**

*Desulfovibrio vulgaris* Hildenborough is a sulfate-reducing bacterium (SRB) that is intensively studied in the context of metal corrosion and heavy-metal bioremediation, and SRB populations are commonly observed in pipe and subsurface environments as surface-associated populations. In order to elucidate physiological changes associated with biofilm growth at both the transcript and protein level, transcriptomic and proteomic analyses were done on mature biofilm cells and compared to both batch and reactor planktonic populations. The biofilms were cultivated with lactate and sulfate in a continuously fed biofilm reactor, and compared to both batch and reactor planktonic populations.

**Results:**

The functional genomic analysis demonstrated that biofilm cells were different compared to planktonic cells, and the majority of altered abundances for genes and proteins were annotated as hypothetical (unknown function), energy conservation, amino acid metabolism, and signal transduction. Genes and proteins that showed similar trends in detected levels were particularly involved in energy conservation such as increases in an annotated *ech* hydrogenase, formate dehydrogenase, pyruvate:ferredoxin oxidoreductase, and *rnf* oxidoreductase, and the biofilm cells had elevated formate dehydrogenase activity. Several other hydrogenases and formate dehydrogenases also showed an increased protein level, while decreased transcript and protein levels were observed for putative *coo* hydrogenase as well as a lactate permease and *hyp* hydrogenases for biofilm cells. Genes annotated for amino acid synthesis and nitrogen utilization were also predominant changers within the biofilm state. Ribosomal transcripts and proteins were notably decreased within the biofilm cells compared to exponential-phase cells but were not as low as levels observed in planktonic, stationary-phase cells. Several putative, extracellular proteins (DVU1012, 1545) were also detected in the extracellular fraction from biofilm cells.

**Conclusions:**

Even though both the planktonic and biofilm cells were oxidizing lactate and reducing sulfate, the biofilm cells were physiologically distinct compared to planktonic growth states due to altered abundances of genes/proteins involved in carbon/energy flow and extracellular structures. In addition, average expression values for multiple rRNA transcripts and respiratory activity measurements indicated that biofilm cells were metabolically more similar to exponential-phase cells although biofilm cells are structured differently. The characterization of physiological advantages and constraints of the biofilm growth state for sulfate-reducing bacteria will provide insight into bioremediation applications as well as microbially-induced metal corrosion.

## Background

It is becoming increasingly clear that a mode of attached growth more closely resembles *in situ* conditions for many microorganisms in different environments and might likely be a universal feature that presents an important physiology to explore in addition to the typically conducted studies on planktonic cells [1,2]. The subsurface is a physically dynamic habitat where fluxes in water, nutrients, temperature, pH, and osmolarity can create challenges for microorganisms to survive and thrive, and dehydration events can inhibit motility and limit nutrient availability that can result in decreased microbial activity [3]. Biofilms can provide a protective habitat for bacterial cells to exist, and recent work has demonstrated *in situ* biofilms in a variety of habitats [4-7]. Biofilm matrices can retain water, sorb nutrients, and protect against rapid changes in salinity, osmolarity, pH, nutrient availability, and redox [3], and we have observed the formation of biofilms (*i.e.*, surface-attached cells) that contain *Desulfovibrio* species in sediments placed down-well at bioremediation field sites (Bowen-DeLeon and Fields, unpublished results).

Biofilms produced by sulfate-reducing bacteria (SRB) have been studied under the context of metal exposure and corrosion. For example, work done with *Desulfovibrio desulfuricans* G20 not only demonstrated the precipitation of hexavalent uranium and lead in the periplasm of the cell but also within the biofilm matrix [8-11]. SRB populations have been observed to adhere to enteric bacteria and other aerobes that have formed biofilms on underground pipelines, allowing further corrosion to occur [12]. Different corrosion studies indicate that the biofilms produced by SRB populations can be composed mainly of proteins, with minimal exopolysaccharide present [13], and this coincides with our study of *D. vulgaris* biofilms on glass in that the EPS (exopolymeric substance) was not enriched for carbohydrate compared to other bacteria [14]. Although these studies provide insights into corrosion and metal-reducing mechanisms of SRB biofilms, little is known about the general response in SRBs that underlie physiological adaptation between the biofilm and planktonic growth modes.

Previous studies done on aerobic or facultative pathogens have shown differential gene expression and protein profiles for biofilm cells compared to planktonic cells in different microorganisms [15-23], and the results suggest that biofilm formation is a complex and highly regulated process [24]. In order to understand molecular mechanisms of *D. vulgaris* survival within the subsurface and the development of SRB biofilms in different environments, we compared *D. vulgaris* Hildenborough biofilm and planktonic cell transcript and protein levels. Our results indicated that *D. vulgaris* biofilm cells altered transcript and protein abundances involved in carbon and energy metabolism, amino acid metabolism, stress response, proteases, and ribosomal proteins. These results suggested that biofilm cells were not merely analogous to planktonic cells in stationary-phase. Characterization of physiological advantages and constraints of the biofilm growth state may lead to a better understanding of how to stimulate (subsurface) or inhibit (corrosion) SRB biofilms.

## Results and discussion

### Growth and sampling

**Figure 1:**
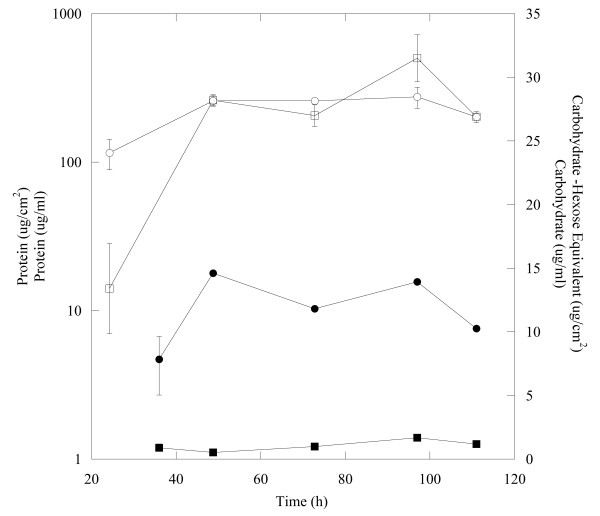
Protein (●,○) and carbohydrate (■,□) levels for biofilm and planktonic cells, respectively from continuous culture with lactate and sulfate.

Three replicates of *D. vulgaris* cultures were grown in a continuous reactor system (CDC biofilm reactors, Biosurface Technologies) with lactate (60 mM) as the growth-limiting energy and carbon source and sulfate (50 mM) as the electron acceptor. The CDC reactors were maintained at a dilution rate of 0.084 h^-1^. Planktonic and biofilm biomass was collected 70 h post-coupon insertion, and both protein and carbohydrate levels had approached steady-state under the tested growth conditions at this time point (Figure  1). By 70 h the biofilm had 12 ± 0.03 μg/cm^2^ protein and the biofilm maintained a carbohydrate:protein ratio (C:P) of approximately 0.13 (μg/μg). Planktonic cultures had a protein level of 250 ± 2.90 μg/ml with an approximate C:P ratio of 0.10 (μg/μg). These results are consistent with the fact that *D. vulgaris* does not produce a carbohydrate-rich biofilm matrix under the described growth conditions [14].

Biofilms were compared via both transcriptomics and proteomics to the planktonic counterparts grown continuously within the reactor system and with planktonic cells grown under batch conditions. Based upon the majority of studies with aerobic and facultative bacteria, the notion of heterogeneity and complexity within biofilms has become increasingly appreciated [25] and can play a role in the interpretation of overall expression analyses. Heterogeneity issues remain to be resolved for biofilm studies; however, models predicted that the *D. vulgaris* biofilms in the described study were not limited for lactate or sulfate at the given biofilm depths (10-30 μm, Additional file 1) based upon substrate diffusivity with a biofilm accumulation model [26]. In addition, complete correlation between the transcriptomic and proteomic data sets cannot be expected [23,27,28] ( Additional file 2), although some genes showed similar transcript and protein trends. Therefore each data set (transcript and protein) revealed key aspects of the physiological state of the cells. While a recent study provided an estimate of translation-related sequence features to protein levels, it is still not known to what degree different mRNA and protein molecules differ with respect to *in vivo* half-lives, although Nie et al. [27] predicted that protein stability only accounted for 5% of the variation.

### Comparison of biofilm to planktonic cells

**Figure 2:**
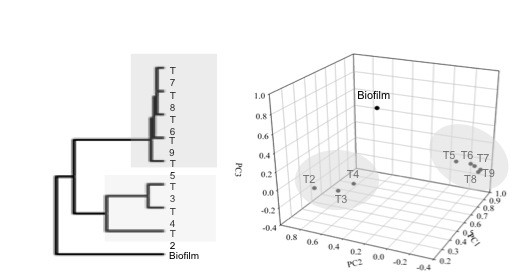
**Hierarchal clustering (a) and principal components analysis (b) of the nine samples from planktonic and biofilm (T2-T4 represent exponential-phase and T5-T9 represent stationary-phase) grown on lactate and sulfate for *Desulfovibrio vulgaris.*** The samples are grouped based upon similarities in the expression patterns of all genes with significant changes.

The mRNA levels between biofilm and planktonic cells were compared using hierarchical clustering analysis (HCL) and principle components analysis (PCA). HCL compared similarities between all significantly differentially expressed genes (i.e. log2 ratio ≥ 2.0 or ≤ -2.0) between biofilm cells and the different planktonic cells. The growth-phase differences of batch, planktonic growth have been previously described [29]. The gene expression profile for biofilm cells did not cluster with exponential- and stationary-phase planktonic cells (Figure  2a). PCA showed a similar result when genes with significantly altered expression were considered (Figure  2b).

**Figure 3:**
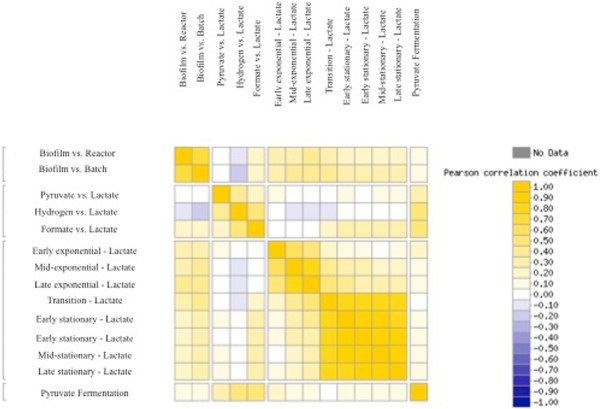
**Correlation matrix for *D. vulgaris* gene expression that compares biofilm cells to planktonic cells grown with different substrates using a centered Pearson correlation.** Cells were grown in a defined S4D medium with sulfate (except for ‘pyruvate only’), and provided with a different carbon and energy source (lactate, pyruvate, hydrogen, or formate). The correlation matrix was generated with MicrobesOnline functional genomics analysis (microbesonline.org).

The biofilm mRNA data were then compared to planktonic cells grown with different carbon/energy sources via a gene expression correlation matrix with a centered Pearson correlation based upon expression of all detected genes (Figure  3). Biofilm cells were distinct when compared to multiple physiological states and were not highly correlated with cells grown planktonically with pyruvate/sulfate (r = 0.002 to 0.03), hydrogen/sulfate (r = -0.08 to -0.2), formate/sulfate (r = 0.2), or fermentatively (r = 0.1). In addition, the comparison of biofilm cells to either reactor- or batch-planktonic cells were more similar to each other (r = 0.70) than the comparison of biofilm cells to the other growth states (*i.e.*, growth-phase and substrates). These results suggested that the biofilm is a physiological state distinct from lactate-grown, planktonic cells (exponential or stationary-phase), and that many genes in biofilm cells had similar expression levels regardless of being compared to reactor- or batch-planktonic cells. In addition, the lactate/sulfate-grown biofilm cells had a disticnt expression profile when compared to planktonic cells grown with different electron/carbon sources (*e.g.*, lactate, hydrogen, formate, pyruvate).

### Transcripts and proteins with increased abundances

**Figure 4:**
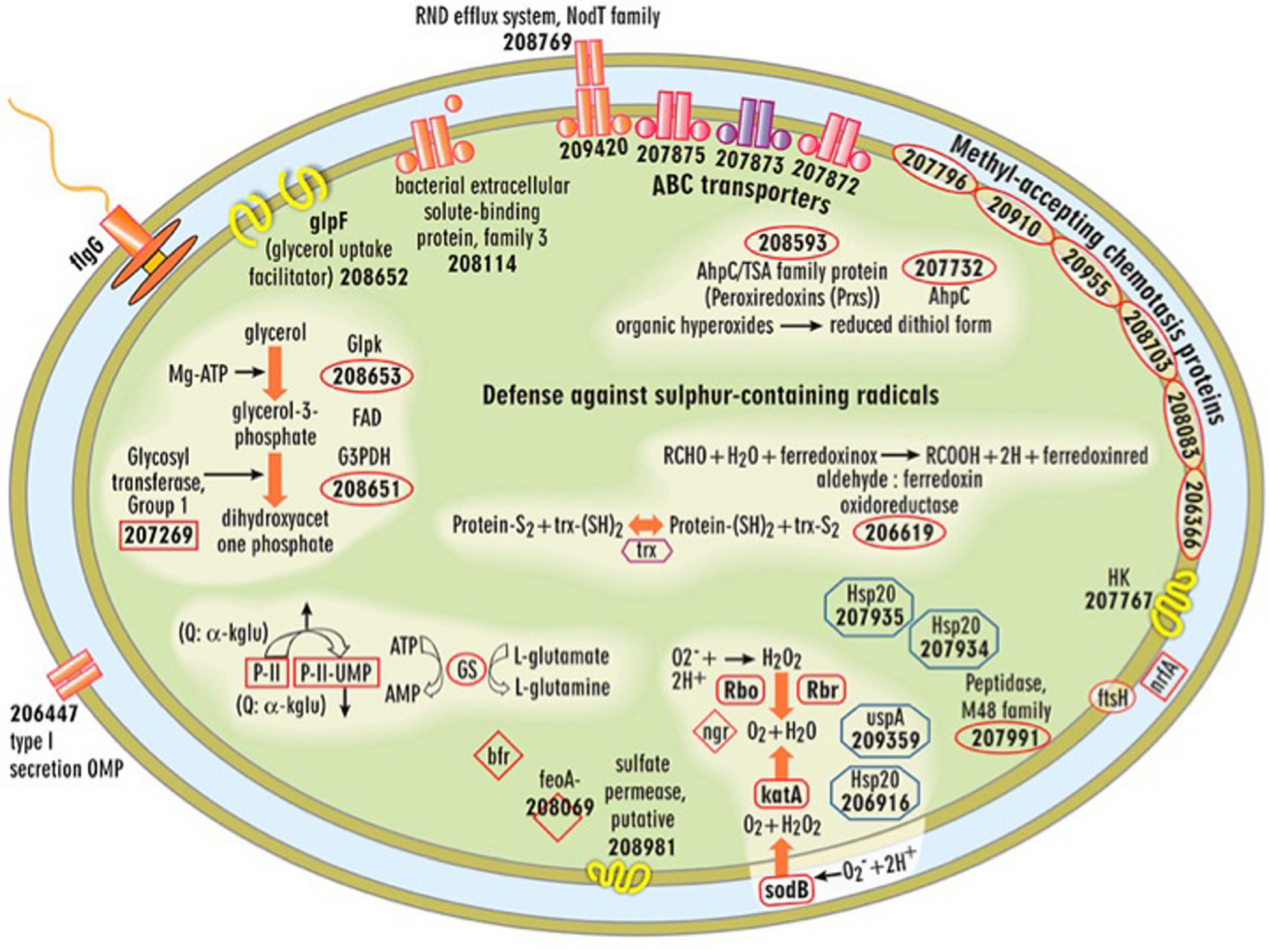
**Cell-wide depiction of significantly up-expressed transcripts for mature *D. vulgaris* biofilms compared to both batch and chemostat planktonic cells.** The following genes were also detected as significantly up-expressed proteins: SodB, KatA, Hsp20 (DVU2441), Hsp20 (DVU2442), UspA (DVU0423).

A total of 231 and 108 transcripts had increased expression in biofilm cells when compared to batch- or reactor-grown planktonic cells, respectively (Table 1 and 2; whole genome expression data in Additional file 3 and Additional file 4). When compared to either reactor- or batch-planktonic cells, most of the same hypothetical and conserved hypothetical genes had altered expression. More motility genes were up-expressed in biofilm cells when compared to batch cells; however, the five genes up-expressed in biofilm cells when compared to reactor-planktonic cells were also up-expressed when compared to batch-planktonic cells. Current work includes the elucidation of flagellar genes needed for biofilm development and maintenance. Regardless of biofilm cells being compared to reactor- or batch-planktonic cells, significantly up-expressed genes in biofilm cells were involved in amino acid metabolism, energy conservation, signal transduction, and translation (Table  1, Table  2, Figure  4).

**Table 1:** COG functional categories with altered expression (z score > 2.0) in biofilm cells compared to batch, planktonic cells (exponential-phase)

**COG Function Categories**	**Up**	**Down**
Amino acid transport and metabolism	19	20
Carbohydrate transport and metabolism	11	3
Cell cycle control, cell division, chromosome partitioning	-	-
Cell motility	15	2
Cell wall/membrane/envelope biogenesis	8	5
Chromatin structure and dynamics	-	-
Coenzyme transport and metabolism	11	8
Defense mechanisms	-	3
Energy production and conversion	44	17
Function unknown	13	9
General function prediction only	18	12
Inorganic ion transport and metabolism	12	8
Intracellular trafficking, secretion, and vesicular transport	5	6
Lipid transport and metabolism	-	10
Nucleotide transport and metabolism	3	4
Posttranslational modification, protein turnover, chaperones	11	3
Replication, recombination and repair	5	5
Secondary metabolites biosynthesis, transport and catabolism	3	3
Signal transduction mechanisms	40	9
Transcription	11	9
Translation, ribosomal structure and biogenesis	2	44

**Table 2:** COG functional categories with altered expression (z score > 2.0) in biofilm cells compared to reactor, planktonic cells (exponential-phase)

**COG Function Categories**	**Up**	**Down**
Amino acid transport and metabolism	6	28
Carbohydrate transport and metabolism	4	1
Cell cycle control, cell division, chromosome partitioning	-	2
Cell motility	5	1
Cell wall/membrane/envelope biogenesis	5	5
Chromatin structure and dynamics	-	-
Coenzyme transport and metabolism	4	11
Defense mechanisms	-	2
Energy production and conversion	22	24
Function unknown	7	5
General function prediction only	10	15
Inorganic ion transport and metabolism	4	5
Intracellular trafficking, secretion, and vesicular transport	2	4
Lipid transport and metabolism	1	5
Nucleotide transport and metabolism	-	1
Posttranslational modification, protein turnover, chaperones	8	4
Replication, recombination and repair	1	9
Secondary metabolites biosynthesis, transport and catabolism	1	3
Signal transduction mechanisms	22	3
Transcription	3	6
Translation, ribosomal structure and biogenesis	1	21

In the iTRAQ proteome analysis, a total of 560 proteins passed the data filtering criteria ( Additional file 5), and there were 11 and 17 proteins that had significantly increased levels in the biofilm cells when compared to the batch or reactor planktonic samples, respectively. Energy conversion, stress response, and unknown function proteins had increased abundances and amino acid metabolism and signal transduction had decreased abundances in biofilm cells when compared to either batch- or reactor-planktonic cells (Table  3 and 4).

**Table 3:** Comparison of biofilm to batch culture

**DVU Gene #**	**Gene Name**	**Description**	**Log**_**2**_**ratio (z-score)**
0429	*ech*F	Ech hydrogenase, putative subunit	3.41 (3.91)
0799	-	conserved hypothetical protein	3.02 (3.46)
2342	-	amino acid ABC transporter	2.34 (2.68)
1817	*cyf*	cytochrome c553	2.31 (2.64)
2781	-	hypothetical protein	2.30 (2.63)
0797	-	conserved hypothetical protein	2.15 (2.46)
0752	-	amino acid ABC transporter	1.95 (2.22)
0588	*hyb*A	formate dehydrogenase, beta subunit	1.94 (2.22)
A0108	-	-	1.87 (2.14)
2982	*leu*C	3-isopropoylmalate dehydratase	1.78 (2.03)
2333	*ndk*	nucleoside dephosphate kinase	1.77 (2.02)
0465	*trp*E	anthrnilate synthase, component I	-1.72 (-2.00)
1207	*fab*H	3-oxyacyl-acyl-carrier protein synthase	-1.72 (-2.00)
0107	*gln*H	glutamine periplasmic binding protein	-1.75 (-2.04)
1873	*ppi*B-2	peptidyl-prolyl-isomerase	-1.78 (-2.07)
0997	*met*F	5,10-methylenetetrahydrofolate red.	-1.79 (-2.09)
3029	*pta*	phosphate acetyltransferase	-1.87 (-2.17)
2449	*met*K	S-adenosylmethionen synthetase	-1.88 (-2.18)
0470	*trp*B-2	tryptophan synthase	-1.94 (-2.25)
1095	*arg*G	argininosuccinate synthase	-1.96 (-2.28)
0462	-	chorismate mutase	-1.99 (-2.31)
0607	*ahc*Y	adenosylhomocysteinase	-2.06 (-2.40)
2590	-	sensory box protein	-2.38 (-2.76)
1241	-	conserved hypothetical protein	-2.55 (-2.96)
3371	*met*E	S-methyltransferase	-2.83 (-3.28)
0494	-	aminotransferase V	-3.30 (-3.82)

This table lists all proteins that were significantly increased or decreased (|z| ≤ 2) when biofilm cells were compared to batch, planktonic cells.

**Table 4:** Comparison of biofilm to reactor, planktonic culture

**DVU Gene #**	**Gene Name**	**Description**	**Log**_**2**_**ratio (z-score)**
0799	-	-	3.65 (4.78)
0797	-	-	3.08 (4.02)
0587	*fdn*G-1	formate dehydrogenase, alpha subunit	2.59 (3.38)
1817	*cyf*	cytochrome c553	2.44 (3.18)
2135	-	hypothetical protein	2.39 (3.11)
2442	-	Hsp family	2.30 (2.99)
0251	-	putative membrane protein	2.20 (2.86)
0588	*hyb*A	formate dehydrogenase, beta subunit	2.11 (2.74)
2410	*sod*B	superoxide dismutase, Fe	2.02 (2.63)
2100	-	universal stress protien	1.85 (2.40)
A0115	*-*	-	1.81 (2.34)
2543	b0873	hybrid cluster protein	1.80 (2.34)
0429	*ech*F	EchF	1.75 (2.27)
0430	*ech*E	EchE	1.66 (2.15)
2441	*hsp*C	Hsp20 family	1.63 (2.10)
1067	-	Bmp family membrane protein	1.60 (2.07)
1876	*dna*J	DnaJ	1.56 (2.02)
0467	*trp*D	anthrnilate phosphoribosyltransferase	-1.52 (-2.06)
0415	*pep*A	cytosol aminopeptidase	-1.55 (-2.10)
3027	*glc*D	glycolate oxidase	-1.80 (-2.43)
2590	-	sensory box protein	-1.81 (-2.44)
0494	-	aminotransferase V	-1.82 (-2.47)
1257	-	RNA-binding protein	-2.00 (-2.69)
0318	-	TPR domain protein	-2.23 (-3.00)

This table lists all proteins that were significantly increased or decreased (|z| ≤ 2) when biofilm cells were compared to reactor, planktonic cells.

When comparing the transcriptomic and proteomic data, some corresponding mRNAs and proteins both displayed increased abundances. For example, a presumptive c553 cytochrome (DVU1817), *ech*F/EchF (DVU0429), hybrid cluster (DVU2543), and *hyb*A/HybA (DVU0588) (Tables 3, 4, Additional file 3 and Additional file 4), and these respective results corroborated the other (discussed below). However, as expected, there were instances that elevated mRNA levels did not correspond to increased protein abundances (*e.g.*, DVU0586, DVU3133, DVU2100, DVU0251). In addition, there were detected proteins with elevated levels and not corresponding mRNA levels when compared to planktonic, reactor cells (*e.g*., DVU0797, DVU0799, DVU2781, DVU0430-*ech*E, DVU0587-*fdn*G1, DVUA0108, DVUA0115). These results are most likely a result of differences in mRNA and protein half-lives and highlight the utility of both transcriptomic and proteomic analyses.

### Transcripts and proteins with decreased abundances

A total of 358 transcripts were down-expressed in biofilm cells compared to the planktonic counterparts grown in either the batch or reactor systems. Similar to the up-expression patterns, numerous down-expressed genes (Table 1 and 2) were annotated as hypothetical or conserved hypothetical proteins. Interestingly, 10% of the down-expressed genes were predicted to be involved with translation and ribosomal structure/biogenesis. Genes involved with translation were down-expressed for biofilm cells compared to either planktonic state (batch or reactor).

**Figure 5:**
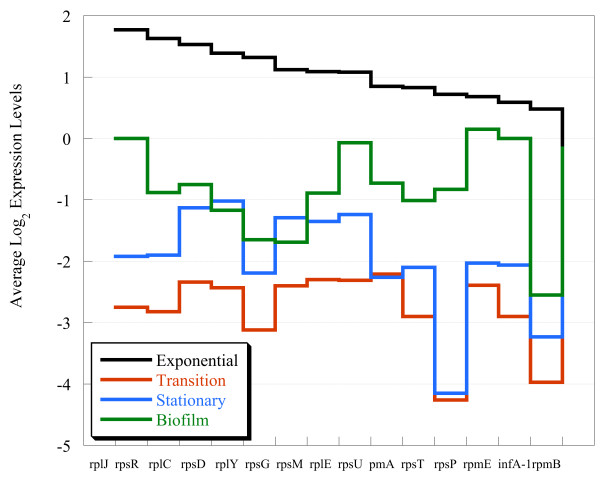
**Transcript levels for 15 ribosomal proteins compared among four (expontial, transition, stationary, and biofilm) different growth stages.** The genes are sorted highest to lowest with respect to the levels observed for exponential-phase cells.

In a previous study, ribosomal gene expression decreased by 3-fold during the exponential/stationary-phase transition for planktonic cells and remained 2-fold down throughout the stationary-phase [29]. Correspondingly, several ribosomal genes were identified in the transcriptomic and proteomic data in the current study and displayed a similar trend in decreased abundances ( Additional file 6). However, when the log-ratios of ribosomal protein transcripts with significant expression changes (n = 15; Additional file 6) were compared between biofilm and planktonic cells in different growth phases, there was less down-expression for biofilm cells compared to stationary-phase cells (Figure 5; -0.8 ± 0.7 vs. -2.1 ± 0.8).

Because the production of ribosomal transcripts/proteins typically correlates with metabolic activity, we sought to confirm this result by measuring the activity levels of planktonic and biofilm cells with the respiratory indicator, 5-cyano-2,3,-ditolyl tetrazolium chloride (CTC) as previously described [30]. Biofilm cells were only 2-fold less active than exponential-phase cells. However, stationary-phase cells were approximately 500-fold less active than exponential-phase cells and 300-fold less active than biofilm cells. The metabolic activity of biofilm cells might not result in ‘exponential’ cell growth (and/or biomass accumulation) *per se*, but may provide the energy required for nutrient uptake and biofilm maintenance. Indeed, a recent study that developed a model to explain variation in growth rate optimization and cellular stoichiometry hypothesized that resource allocation and nutrient-use efficiency was a result of distinct growth and uptake activities [31]. Our results suggested that the biofilm physiological state should be further evaluated in the context of ‘growth’ and resource allocation in comparison to planktonic cells.

The notion that biofilm cells were different from stationary-phase cells contradicts previous work that estimates the strong gene expression similarity between these growth states for different bacteria, albeit mostly aerobes [16,32]. These results corroborated the increased expression of ribosomal transcripts and protein levels that indicated biofilm cells were active and that slower cell division (*i.e.*, steady-state biofilm) does not equate to lower metabolism. In order to determine possible functions that contributed to the distinct state of *D. vulgaris* biofilms, we further analyzed various functional categories of genes and proteins in biofilm cells with altered abundances.

### (i) Energy conversion

**Figure 6:**
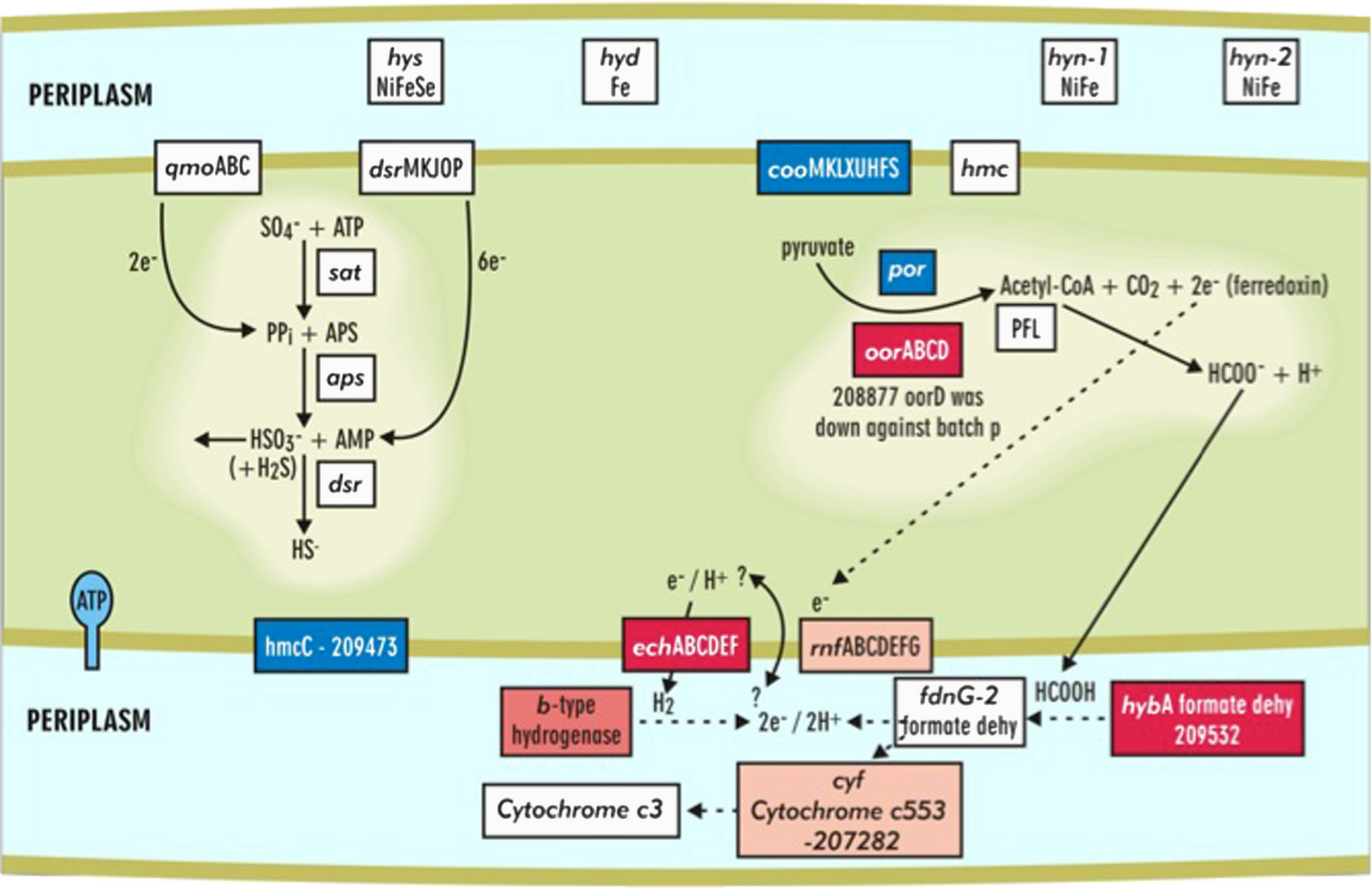
**Conceptual model (a) of the transcriptional responses in energy metabolism and pyruvate-acetate metabolic node for a mature* D. vulgaris *biofilm (red is up-expressed and blue is down-expressed) compared to planktonic cells.**

**Figure 7:**
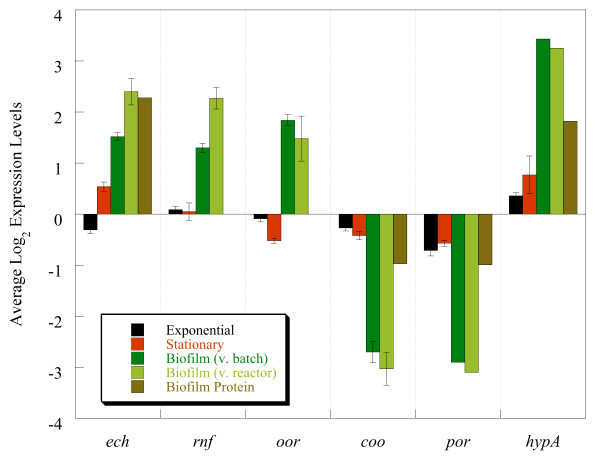
Expression levels of representative genes involved in carbon and energy flow that displayed altered expression distinct to biofilm cells when compared to reactor planktonic cells, batch exponential-phase, or batch stationary-phase.

The expression of genes involved with energy conversion and carbon flow within the biofilm is a major contributor to the uniqueness of the biofilm growth state regardless of being compared to batch- or reactor-planktonic cells. Biofilm cells had different mRNA and protein levels related to the pyruvate to acetate and formate metabolic nodes. Two annotated pyruvate:ferredoxin oxidoreductases, which convert pyruvate to acetyl-CoA, had differential expression in biofilm cells with up-expression of *oor* (DVU1944, DVU1945, DVU1946, and DVU1947) and down-expression of *por* (DVU1569 and DVU1570) compared to planktonic cells (Figure  6 and 7). Expression of the mRNA was verified via qPCR, and *oor* and *por* genes were up- and down-expressed, respectively.

Although expression for the annotated pyruvate-formate lyase was not altered, the abundance of formate dehydrogenase (DVU0588-*hyb*A transcript and protein) was increased (Figure  6, Table  3 and 4). However, only protein abundance was increased for *fdn*G1 (DVU0587) (Table  4). A cytochrome c553 (DVU1817), which is the electron transfer partner for the formate dehydrogenase in *D. vulgaris*[33], had increased abundances for both mRNA and protein levels (Figure  6, Table  3 and 4). Because formate dehydrogenase was increased in biofilms, enzymatic assays were done to compare activity within biofilm and planktonic cells. Crude extracts of biofilm cells had an approximate 26-fold higher rate of formate-dependent methyl viologen reduction than reactor planktonic cells, and these results indicated that the increased gene expression corresponded to increased enzymatic activity.

Formate, like hydrogen, has been proposed to be a mechanism of electron flow in *Desulfovibrio*[34,35]. Formate cycling within lactate-respiring biofilm cells could explain the increase in expression and activity of the formate dehydrogenases and might suggest that biofilm cells are better able to cycle formate compared to planktonic cells in a stirred, homogenous environment. Additional hydrogenases were also differentially expressed within the biofilm cells compared to planktonic cells (Figure  6; DVU3145). The possible route of proton and electron cycling in SRB has long been debated and biofilm cells could be an additional physiological state to provide significant insights.

### (ii) Carbohydrate metabolism

*D. vulgaris* biofilm cells had several genes involved with glycerol uptake and utilization shift in expression (Figure 4, Additional file 5). These results suggested that biofilm cells may use phospholipids released from lysed neighboring cells and that these may possibly serve as a carbon backbone for amino acid biosynthesis because the overall glycolytic pathway was not significantly up-expressed. In addition, an annotated fructose-1,6-bisphosphatase (DVU1539) was transcriptionally down-expressed in biofilm cells compared to reactor planktonic cells and suggested a decrease in gluconeogenesis. Decreased gluconeogenic activity may indicate a decreased requirement for new amino sugars for cell wall synthesis, and this observation corresponded with the decreased expression of genes involved with cell wall synthesis and cell division. It should be noted that biofilm cells did not appear to be smaller based upon electron microgrpahs (data not shown).

### (iii) Nitrogen metabolism

The increased expression of *nrfA* (DVU0625) may indicate the available nitrogen source (*i.e.*, ammonium) might be limited within some biofilm cells. *nrfA,* annotated as cytochrome c nitrite reductase (c552), had increased mRNA expression within biofilm cells compared to planktonic cells. This result is interesting because ammonia was added as the nitrogen input into the system and nitrite reduction has not been linked to growth [35,36]. *D. vulgaris* also up-expressed *nrf*A when cells were exposed to acetone, ethanol, nitrite, and alkaline pH [37]; He and Zhou, unpublished results], and these results suggested that *nrf*A expression may be part of a more general response. *D. vulgaris* can utilize nitrogen via the nitrogenase that is encoded on the 200-kb plasmid (pDV1), but *nif*U gene expression did not change as detected via qPCR (data not shown).

### (iv) Stress response

Various chaperone proteins, including *usp*A (DVU0423) and three *hsp*20 genes (DVU2442, 2241, and 1471), had increased transcripts within the biofilm compared to both reactor and batch planktonic cells (Figure  4). *D. vulgaris* biofilms had increased transcripts for the stress response proteins superoxide dismutase (*sodB* DVU2410), alkyl hydroperoxide reductase (*ahpC* DVU2247), *msr*B (DVU0576) and *ahp*C/TSA (thiol specific antioxidant) and an increase in protein levels for SodB (Table 4). However, preliminary results indicated that *D. vulgaris* biofilm cells do not have increased tolerance to dissolved oxygen in terms of growth initiation (Sundararajan and Fields, unpublished results).

Although mRNA levels for stress response genes were elevated in the biofilms of other organisms (*e.g.E. coli, P. aeruginosa, Legionella pneumophila* and *Staphylococcus aureus*)*,* the anaerobic, hyperthermophile *Thermotoga maritima* showed decreased expression in similar genes [16-18]. In addition, *D. vulgaris* grown on steel slides showed a similar trend in down-expression of related genes [38]. For aerobic bacteria, oxidative stress within biofilms is thought to be a result of slow growth in conjunction with a shift away from oxygen respiration at different biofilm depths [25]. For *D. vuglaris* biofilms, the relatively slower growth may be associated with lower intracellular levels of reducing equivalents that would leave the cell more susceptible to potential oxidizing agents, and a defense mechanism could be elevated levels for genes and proteins involved in potential detoxification.

These responses are also thought to increase during iron limitation due to the increase in iron sequestration and intracellular accumulation [39]. This is not surprising given the ability of some metals to form hydroxyl radical, metallo-oxo, and metallo-peroxo species [40]. The elevated abundances for genes and proteins involved in detoxification of oxidative agents also coincided with the up-expression of iron acquisition mechanisms in biofilm cells (see below) and the possible connection between iron storage and oxidative damage response may at least partially explain the observed expression profiles.

Interestingly, more genes annotated as proteases were down-expressed, including *lon* protease (DVU1337), *clp*X (DVU1336), *clp*B (DVU1874), *htr*A peptidase (DVU3278), *htp*G (DVU2643), and *hfl*C (DVU0683) than were up-expressed. A decrease in proteases for *D. vulgaris* biofilm cells contrasted with observations for stationary-phase planktonic cells [29]. Biofilm cells might simply have a slower turnover of proteins due to the biofilm growth mode, but further research is needed to determine this characteristic.

Biofilm cells had altered transcript expression profiles for methyl-accepting chemotaxis proteins, histidine kinases, response regulators, and sensory box proteins (Figure  4, Table  1, 2, 3, and 4, Additional file 5). *D. vulgaris* contains a number of two-component signal transduction systems and methyl-accepting chemotaxis proteins; however, little is known about the required signals and regulation that these proteins may impose on the cells nor how that regulation contributes to biofilm formation and/or disassembly.

### (v) Iron acquisition

*D. vulgaris* biofilms may be iron limited as indicated by the increase in transcripts for the subunit *feoA* (DVU2572), part of the iron transporter Feo, and *fep*C (DVU0103)*,* a presumptive ABC transporter for iron uptake. However, similar genes were up-expressed in *D. vulgaris* stationary-phase, planktonic cells [29], and it is possible that an iron acquisition response is the result of increased sulfides associated with *D. vulgaris* growth in general. The iron storage protein bacterioferritin (DVU1397) had increased transcripts within biofilm cells along with the ferritin storage protein (DVU1568). Increased abundances of iron sequestration proteins has been reported in other biofilm studies [17,38,39] but iron transport systems like *feoAB* were transcriptionally down-expressed in *D. vulgaris* biofilm cells grown on steel [38]. Several studies have indicated that iron plays different roles within biofilm formation of various organisms. *P. aeruginosa* uses iron as a signal to produce thick biofilms with mushroom morphology while excess iron is detrimental to *S. aureus* and *L. pneumophila* biofilms [39,41,42]*.*

### (vi) Transporters

Biofilm cells up-expressed four presumptive ABC transporter genes (DVU2387, DVU2384, DVU0484, and DVU2385) and increased abundances for two transporter proteins (DVU0752 and DVU2342) (Figure  4, Table  3). Differential expression of various ABC transporters has commonly been reported in different biofilms (*e.g., P. aeruginosa, E. coliB. subtilisThermotoga maritimeStreptococcus mutans* and *S. aureus*) [16,17,19,20]. The putative substrates have yet to be determined, but biofilm cells may transport different small molecules and solutes compared to planktonic cells.

For *D. vulgaris* biofilm cells, various subunits of the Sec transport system were transcriptionally down-expressed including *sec*E (DVU2922), *sec*Y (DVU1323), and *sec*G (DVU1676), and SecD at the protein level. Interestingly, a preprotein translocase, YajC (DVU1820), was increased at the protein level. Different types of secretion may be increased as indicated by increased transcripts of a type I system (DVU1013) and increased protein levels of an annotated type III protein secretion system (DVUA0115). The ORF, DVUA0115, is located on the *D. vulgaris* plasmid (pDV1), and a strain cured of the plasmid is biofilm deficient [14].

Biofilm cells differentially expressed two putative sulfate permeases at the mRNA level; DVU0053 was up-expressed and DVU0279 was down-expressed. A putative lactate permease mRNA (DVU2285) was down-expressed in biofilm cells, and a similar trend of expression was observed with exponential-phase, planktonic cells [29].

### (vii) Extracellular proteins

In-gel digestion analysis of the extracellular samples revealed 188 proteins that passed the proteomic analysis cutoff criteria. The list was screened against signal and transmembrane domain predictions generated by Phobius [43] and revealed a general enrichment for proteins with signal sequences and few proteins that contained transmembrane domains were identified ( Additional file 7). The most highly abundant protein in the sample was DVU1012, annotated to have a vonWillebrand factor domain (vWF). Interestingly, vWF proteins are large, multimeric glycoproteins that can have a variety of roles with different ligands that include cell adhesion, pattern formation, and signal transduction [44]. However, the presumptive protein is also annotated to contain a hemolysin-type calcium-binding domain and only has 21% homology with the closest homolog. Interestingly, a paralog of DVU1012, DVU1545, was also enriched in the extracellular protein fraction from biofilms. In addition, two presumptive proteins that had increased protein but not mRNA levels, DVU0797 and DVU0799, were also detected at significant levels in the extracellular, biofilm fraction. Hence, though the function of these proteins in *D. vulgaris* remains obscure, participation in extracellular matrix formation in biofilm structures could be a potential role. Further work is underway to determine both structural and enzymatic proteins in the extracellular biofilm.

### (viii) Fatty acid and lipid synthesis

Numerous genes and proteins involved in fatty acid synthesis had lower abudances in biofilm cells (Table  1, 2, 3 and 4, Additional file 3, Additional file 4, Additional file 5). The gene *lpxC* (lipid A production) that encodes UDP-3-0-acyl N-acetylglucosamine deacetylase (DVU2917) was decreased in biofilm cells. Transcripts for a TolB protein, which is part of the peptidoglycan-associated lipoprotein (PAL) multiprotein complex and associates with OmpA and Lpp to contribute to membrane integrity and peptidoglycan turnover, were decreased. There were also decreased protein levels for undecaprenyl diphosphate synthase (DVU0869). These results corroborated the suggestion that biofilm cells were in a different growth state relative to exponential-phase cells in terms of cellular division (*i.e.*, slower cell wall and membrane synthesis).

### (ix) Amino acid and nucleotide production

The decrease in amino acid and nucleotide biosynthesis transcripts and/or proteins within biofilm cells corresponded well with the reduction of protein synthesis and cell division (Tables  1, 2, 3 and 4). Only two genes annotated as enzymes involved with amino acid synthesis were increased in biofilm cells, *glt*A (DVU2476) and *cysK/*CysK (DVU0663) that encode putative glutamate and cysteine synthases, respectively. Cysteine synthase converts O-acetyl-serine and hydrogen sulfide to cysteine. Transcriptional analysis of *T. maritima* biofilms revealed an increase in cysteine biosynthesis enzymes as well as an increase in iron and sulfur uptake systems and proteins used for the incorporation of iron-sulfur clusters [17]. Since sulfates are usually incorporated into cells by the cysteine biosynthesis pathway, the authors suggested that an increase in transcripts for cysteine biosynthesis was a result of the demand for iron-sulfur containing proteins [17]. Several iron-sulfur containing proteins had elevated abundances at both the transcript and protein levels within *D. vulgaris* biofilms. In addition, increasing amounts of cysteine are toxic to *D. vulgaris* planktonic cells, and growth effects are observed above 1 mM (data not shown). Whether the increase in enzymes involved with cysteine biosynthesis is due to a demand for sulfur acquisition and/or a means to help regulate local sulfide concentrations is yet to be determined.

**Figure 8:**
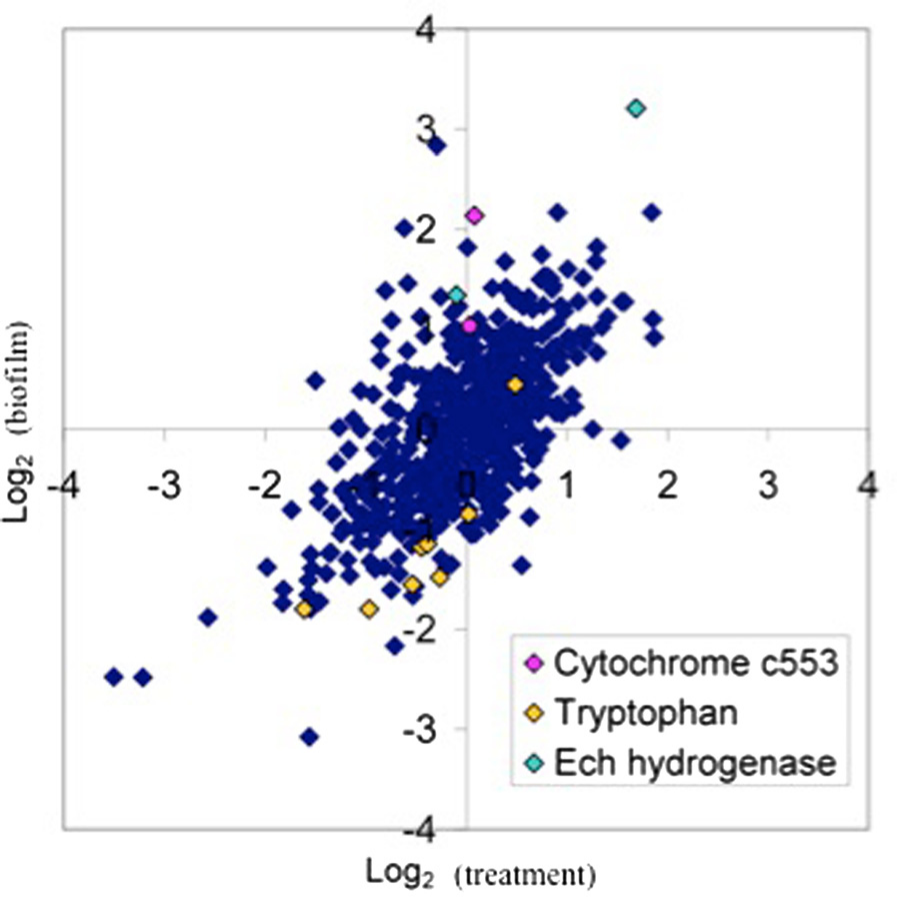
**Graphical representation of the biofilm to planktonic comparison via log**_**2**_**transcript ratios.** As an example, Ech hydrogenase genes and cytochrome c-553 components had increased expression in biofilm cells and tryptophan biosynthesis genes were down expressed.

Genes annotated for enzymes involved in tryptophan biosynthesis were down-expressed within biofilm cells (8 of the 12 genes in the operon were significantly down-expressed) (Figure  8). Two enzymes that contribute to aromatic amino acid and quinone synthesis, 3-octaprenyl-4-hydroxybenzoate carboxy-lyase (DVU3307) and type II 3-dehydroquinate dehydratase (*aroQ* DVU1665) were also down-expressed. Coinciding with these data, seven of thirteen enzymes involved with converting phosphoenolpyruvate and erythrose 4-phophate into tryptophan had decreased protein abundances ( Additional file 3, Additional file 4, Additional file 5). In previous studies, enzymes involved in tryptophan biosynthesis were elevated in young *E. coli* biofilms but declined during biofilm maturation [45,46]. In this study, the decreased levels of tryptophan genes/proteins corresponded with the decreased abundances in enzymes involved with converting tryptophan to indole, which is thought to inhibit biofilm formation in *E. coli*[46,47]. Although *D. vulgaris* has an annotated tryptophanase, expression did not change significantly within the biofilms. The effect and/or role of indole on *D. vulgaris* biofilm formation and maturation are not known; however, hydroxyindole increased the formation of *D. vulgaris* batch biofilms 2-fold (Ramsay and Fields, unpublished results).

## Conclusions

Our study compared multiple *D. vulgaris* transcriptomes and proteomes from different growth phases in the same defined sulfate medium with the same carbon and energy source (lactate). The data indicated that the *D. vulgaris* biofilm growth mode was distinct when compared to both exponential- and stationary-phases in the planktonic state. The biofilm transcriptome did have a cluster of genes (n = 70) with similar expression patterns to stages of stationary-phase that included putative proteins involved in DNA modification, protein turnover, signal transduction, and phage genes, but over half of the genes were annotated as hypothetical or conserved hypothetical proteins. The role of the hypothetical proteins (proteins of unknown function) are not known but likely play a role in the distinct physiological state of the biofilm [48].

In comparison, most of the genes that had similar expression values between biofilm and exponential-phase cells displayed a trend of down-expression (*e.g.*, phage genes) or no significant change. In addition, the transcript levels for 15 rRNAs were more similar to exponential-cells as were metabolic indicator assays. These results indicated that the biofilm growth-phase was a distinct mode of growth that was not analogous to stationary-phase and this result contradicts previous studies with aerobic microorganisms.

Gene expression responses in the biofilm cells included the down-expression of an operon that encoded *por* (pyruvate:ferredoxin oxidoreductase), acetate kinase, a possible lactate dehydrogenase, a phosphotransacetylase, and *ech*/Ech. Interestingly, preliminary results with a Δ*ech*A mutant indicated a deficiency in biofilm formation (Sabalowsky, Wall and Fields, unpublished results). Also related to carbon flow, mRNA for three of the four subunits (*oor*ABD) of a separate PFO were up-expressed in the biofilm cells (Figure  6 and 7). The putative *por* gene encodes a large (1215 aa), multi-domain protein whereas the *oor* genes encode putative subunits of a multimeric protein [49]. Both *por* and *oor* are predicted to catalyze decarboxylation of pyruvate to acetyl-CoA, but the altered levels may result from different regulatory features of the different PFOs (*i.e.*, multimeric vs. mutlidomain) or different kinetic characteristics.

The respective increase and decrease of presumptive electron transfer systems suggested that electron flow is altered in biofilms compared to both exponential- and stationary-phase cells. The different patterns of gene expression for modules in electron transfer systems may reflect differences in rates of nutrient acquisition and incorporation. These results indicate that *D. vulgaris* biofilm has an altered metabolism not a lower metabolism. With respect to hydrogen, a recent study showed that biofilm cells grown on an electrode up-expressed *hyn*-1 and *hyd*, [NiFe] and [Fe] hydrogenases, respectively, as well as *hmc* genes [50]. In comparison, *D. vulgaris* biofilms on glass slides did not up-express the same hydrogenases nor the *hmc* genes when grown as biofilms with sulfate and lactate.

Future work is needed to discern protein interactions that control metabolic flux and cell behavior through particular nodes in order to elucidate the relationships between genotype and phenotype in the context of distinct cellular states, including young and mature biofilms. While the expression of other systems is certainly involved in biofilm physiology, the presented results indicated that sulfate-reducing biofilm cells use a unique combination of genes and proteins to establish distinct energy flow and cell associations different from sulfate-reducing planktonic cells. Similar developmental states have been described in *P. aeruginosa* and *Candida albicans* biofilms [18,51]. This study provides insights into the formation, growth, and development of anaerobic, sulfate-reducing biofilms, and the improved understanding of the genetic control for SRB biofilms will have implications for biotechnology, the environment, and human health.

## Methods

### Bacterial strains and growth conditions

Cultures of *Desulfovibrio vulgaris* ATCC 29579 were acquired from Dr. T.C. Hazen (Lawrence Berkeley National Laboratory). Three CDC reactors that contained LS4D defined medium [29] were used to propagate biofilms, and the CDC reactors (continuous culture conditions) were maintained at 30°C, continuously stirred at 125 rpm, and nitrogen was sparged into the head-space at a rate of 0.85 kPa/min to ensure anaerobic conditions. Growth was initiated with an exponential-phase culture and cells were incubated for 24-h in a batch-mode. After the 24-h incubation, eight modified coupon holders were inserted into each CDC reactor that held glass slides and fresh medium was pumped into the reactor from an anaerobic reservoir (D = 0.084 h^-1^). Biofilms were allowed to form for 70-h and the reactor monitored for cell, lactate, acetate, and sulfate levels. At the end of the 70-h biofilm growth period, coupons were removed from each reactor and scrapped in order to collect the biofilm biomass. Biofilm growth in reactors showed that under these conditions, steady-state biofilm growth was reached within 70-h with consistant protein and carbohydrate levels (Figure  1).

### Sample collection for transcripts and proteins

Three coupons from each reactor were pooled together and scraped into ice-cold phosphate buffered saline (1 ml PBS; pH 7.2) to represent three biological replicates for transcriptomic analysis. Samples were kept on ice and were centrifuged immediately after scraping at 5,000 x g for 6 min. The PBS supernatant was removed after centrifugation and pellets were immediately frozen in liquid nitrogen. Concurrently, planktonic cells (60 ml) were collected from each reactor and immediately chilled as described previously [29] for transcriptomic analysis. The chilled samples were pelleted via centrifugation (4,000 x g; 6 min; 4°C). The supernatant from these samples was removed and pellets were immediately frozen in liquid nitrogen. All pelleted samples were stored at -70°C.

The remaining five coupons from each reactor were rinsed briefly with ice-cold 50 mM HEPES and pooled in ice-cold 500 mM TEAB 4 M urea solution (1 ml). The sample was sonicated for 10 min on ice and pelleted via centrifugation (30 min at 10,000 x g at 4°C). Concurrently, planktonic-phase culture (33 ml from each reactor, V_T_ = 100 ml) was collected and pelleted via centrifugation (4,000 x g; 10 min; 4°C). The pellets were rinsed in 50 mM HEPES (1 ml) by vortexing and pelleted again via centrifugation (5,000 x g; 8 min; 4°C). The pellets were resuspended in 500 mM TEAB 4 M urea (1 ml) and sonicated on ice for 10 min. After sonication, samples were pelleted via centrifugation (10,000 x g, 30 min; 4°C) and supernatants were saved. A separate planktonic sample grown under batch conditions in LS4D was also collected and sonicated as described above. All samples were stored at -70°C.

### Chemical analysis

Protein levels were measured via the Lowry Method [52] with bovine serum albumin (Pierce Biochemicals) as the standard. Lactate, acetate, and sulfate concentrations were determined by ion chromatography (Metrohm-Peak) with a Metrosep organic acid column and a Metrosep Anion Supp 5 column as described previously [29]. All measurements were done in duplicate.

### Oligonucleotide probe design and microarray construction

DNA microarrays that included 3,482 of the 3,531 annotated protein-encoding sequences of the *D. vulgaris* genome were constructed with 70-mer oligonucleotide duplicate probes as previously described [28,29,53]. Following examination of the entire probe set using the oligonucleotide probe design criteria [52], 3,471 (97.1%) specific oligonucleotide probes were obtained, and 103 probes (2.9%) were nonspecific. In addition, 10 oligonucleotides for 10 human genes and 10 oligonucleotides for 10 *Arabidopsis* genes were used with the *D. vulgaris* genome for positive (with mRNA spiked) or negative (without mRNA spiked) controls. Each slide contained duplicates of each oligonucleotide probe. Six concentrations of genomic DNA were spotted (eight duplicates on a single slide) as positive controls.

### Total RNA extraction, purification, and labeling

RNA was isolated, transcribed and labeled as described previously [29,53,54]. Briefly, total cellular RNA was extracted using the TRIzol reagent (Invitrogen, Carlsbad, CA) and was purified with an RNeasy mini kit (QIAGEN Valencia, CA) with an on-column DNase treatment. cDNA probes were generated by reverse transcription (RTase) of 10 μg purified RNA with random hexamers (Invitrogen) labeled with Cy3-dUTP or Cy5-dUTP (Amersham Biosciences, Piscataway, NJ). RNA was removed by NaOH treatment after labeling and cDNA was immediately purified with a QIAGEN PCR mini kit. Two samples of each total RNA preparation were labeled, one with Cy3-dUTP and the other with Cy5-dUTP, for microarray hybridization.

### Microarray hybridization, washing, and scanning

For determination of the overall hybridization signals, the sensitivity, and the number of genes detected, a genomic DNA or RNA sample labeled with a single dye was used as previously described [28,29,53,54]. Hybridization was performed using a slide for each biological replicate.

### Microarray image quantification and data analysis

To determine the signal fluorescence intensity for each spot, 16-bit TIFF scanned images were analyzed with ImaGene v6.0 (Biodiscovery, Marina Del Rey, CA) to quantify the spot signal, spot quality, and background fluorescent intensities. Empty spots, poor spots, and negative spots (*e.g.*, spots without deposited probe) were flagged according to the instruction of the software and removed in the subsequent analysis as previously described [28,29,53,54]. The data files were subjected to Lowess intensity-based normalization and were analyzed further with the MicrobesOnline (microbesonline.org) workbench as previously described with a statistical model that incorporated both per-gene variance (*z* values) and operon structure (OpWise) to compute the posterior probability that the expression level of each gene changed in the direction indicated by the mean value [55,56]. Data for members of operons without a consistent signal for replicates were not used and genes with log_2_ ratios of | *z* | > 2.0 were considered significant. The microarray expression data will be publicly available within the microbesonline workbench (microbesonline.org) as well as GEO (GSE35883).

### Protein preparation, labeling, and analysis

For the planktonic cell sample, 35-mL aliquots of planktonic cells were harvested from three separate bioreactors in separate falcon tubes. The resulting cell pellets were pooled into 1 mL of lysis buffer (500 mM triethylammonium bicarbonate with 4 M urea). To harvest biofilm, the glass slides were rinsed gently with 50 mM phosphate buffered saline, pH 7.2, before being scraped off the glass slides directly into 1 mL of lysis buffer. To provide a non-biofilm associated control, a separate 100 mL *D. vulgaris* aliquot was grown in a serum bottle at 30°C for 30 hours, reaching a maximum OD_600_ of 0.86. The sample was pelleted serially into a single 50 mL falcon tube. The pellet was washed gently in 1 mL of 1 M HEPES buffer, pH 7.2 and the sample was pelleted again. The final pellet was resuspended in 1 mL of lysis buffer. The three samples (biofilm, planktonic, and batch) were lysed by pulsed sonication on ice for 10 minutes and the samples were clarified by centrifugation at 10,000 × *g* for 30 min. Samples were shipped on dry ice overnight. iTRAQ labeling was carried out as previously described [57]. Briefly, samples were iTRAQ labeled and pooled as per manufacturers instructions (Applied Biosystems, Framingham, MA, USA). The labeled samples were acidified (pH 3) and fractionated via strong cation exchange (Ultimate HPLC, Famos Autosampler, Dionex-LC Packings, Sunnyvale, CA, USA) using a PolyLC Polysulfoethyl A column (4.6 mm × 100 mm) and a three step 0-100% gradient in buffer B (800 mM KCl, 25% ACN, and 0.1% FA). Fractions were collected at a flow rate of 700 μl/min on the basis of the UV trace at 214 nm. Several fractions were pooled post-collection to yield a total of 20 fractions. Each fraction was desalted (C18 MacroSpin Columns (Nest Group, Southborough, MA), dried using a vacuum centrifuge, reconstituted in 86 μL 0.1% FA and subjected to reverse phase separation, (Ultimate HPLC, Famos Autosampler, Dionex-LC Packings, Sunnyvale, CA) using a PepMap100 column (75 μm × 15 cm). A flow rate of 200 nL/min with buffers 2% ACN, 0.1% FA (A) and 80% ACN, 0.1% FA (B) was used at a gradient of 0-30% B in 120 min, 30-100% B in 5 minutes, 100% B for 10 minutes, 100-0% B in 5 minutes, and 0% B for 20 minutes. Elutions from the reverse phase column were labeled as follows: tag_114_, biofilm; tag_115_, batch culture; tag_116_, biofilm replicate; tag_117_, planktonic cells from bioreactor. Because tag_114_ and tag_116_ were technical replicates, the reported ratios are the average of log_2_ (114/115) and log_2_ (116/115). The internal error was defined as the value at which 95% of all proteins had no deviation from each other, where the deviation was the absolute value (0.28) of the difference between log_2_ (114/115) and log_2_ (116/115).

### Extracellular protein analysis

To examine the protein content of the extracellular matrix of the biofilm via scraping the glass slides from a single bioreactor into 1.5 mL of chilled 50 mM HEPES buffer (pH 7.2). The resuspended samples were pipetted up and down through a 12-gauge needle for ten repetitions to break apart the extracellular matrix, and then the sample was filtered through a 0.22-μm polyethersulfone (PES) filter to remove cells and other extracellular matrix material. The remaining soluble fraction was concentrated to approximately 300 μL using a Millipore centricon concentrator. Due to the dilute nature of the extracellular fraction, the proteins were separated on an SDS-PAGE gel, as previous in-solution digestions did not yield protein identifications. Sample extracted from 30 biofilm slides was loaded into 3 lanes on a 12-well, 4-12% NuPage Bis-Tris gels (Invitrogen, Carlsbad, CA) and run in MOPS buffer for 1 hour. The gel was stained with Coomassie Brilliant Blue, and destained with 45% methanol and 10% acetic acid solution overnight. Bands were excised from the gel across all three lanes and were subjected to an in-gel digestion protocol. Prepared samples were split into three equal portions, one of which loaded onto a reverse-phase column as previously described [57]. Reverse-phase separation was completed as described except that the gradient was run as follows: 0-30% B in 30 min, 30-100% B in 5 minutes, 100% B for 10 minutes, 100-0% B in 5 minutes, and 0% B for 20 minutes. Data analysis proceeded as previously described [57].

### Transcriptome comparisons

Microarray data sets for different times were analyzed using average linkage hierarchical cluster analysis with Euclidean distance matrices and were visualized with TreeView as previously described [28,29,37]. If there were expression data for a gene for all sample times and the differential expression of log_2_ (ratio) was ≥2.0 or ≤ -2.0 at one or more times, the gene was used for clustering analysis (ratio of the biofilm gene expression versus different planktonic time points from a batch culture). Gene expression with a log_2_ (ratio) of ≥2.0 was considered up-expression, and gene expression with a log_2_ (ratio) of ≤ -2.0 was considered down-expression. The same data set was used for principal components analysis (PCA). The correlation matrix of multiple transcriptomes was generated with a centered Pearson correlation using MicrobesOnline functional genomics analysis (microbesonline.org). The data sets for growth with the different substrates will be described in separate manuscripts, but all growth was done in the same defined medium as used for biofilm growth in this study.

### Quantitative PCR

Biofilm and reactor planktonic cells were grown and harvested as described above for quantitative PCR (qPCR). Four biofilm coupons and 20 mL of reactor planktonic cells were collected at 35 and 70 h post coupon insertion and nutrient flow. RNA was extracted and purified as described above except that in addition to the on-column DNase treatment a second DNase treatment using Turbo DNase (Ambion) was done following the manufactures instruction. cDNA was prepared by reverse transcription with appropriate controls as previously described [29]. Briefly, 10 μg total RNA was mixed with 6 μg of random primers (Invitrogen) and was incubated at 70°C for 10 min followed by a cooling on ice for approximately 1 min. After cooling, 1 μl of reverse transcriptase (Invitrogen), 4 μl of 5X reverse transcription buffer, 2 μl of 0.1 M dithiothreitol, 1 μl of RNase-Out inhibitor (Invitrogen), and 1 μl of a solution containing each deoxynucleoside triphosphate at a concentration of 10 mM were added to each tube. The tubes were incubated at 42°C for 2 h, 70°C for 15 min, and then cooled on ice. A 2.5-μl sample of cDNA (4 ng/μl) was used for qPCR.

The primers used for qPCR were diluted to a concentration of 12.5 μM (Table  1). Each qPCR mixture (final volume, 25 μl) contained 12.5 μl of SYBR Green Master Mix (Applied Biosystems), 0.7 μl of 12.5 μM solution of the forward primer, 0.7 μl of a 12.5 μM solution of the reverse primer, 2.5 μl of cDNA template, and 8.6 μl of nuclease-free water. The real-time PCR was carried out with a Smart Cycler II (Cepheid, Sunnyvale, CA) using the following conditions: one cycle of 95°C for 10 min and 45 cycles of 95°C for 15 s, 55°C for 30 s, and 60°C for 30 s. A serial dilution of genomic DNA was used to generate a standard curve. A negative control that did not contain RTase was included for all samples. Real-time PCR analysis was used to determine the levels of expression of seven genes. The results from the microarray and real-time PCR analysis revealed a high degree of correlation (*R* = 0.82). Similar results were observed in previous studies that used similar microarray procedures and techniques [28,29,37,53].

### CTC Staining

Metabolic activity was measured with the redox dye 5-cyano-2,3-ditolyl tetrazolium chloride (CTC) as previously described [58]. Briefly, biomass was collected aseptically under anaerobic conditions for exponential planktonic, stationary planktonic, and biofilm cells. Samples were pelleted via centrifugation (2,000 x g at room temperature) and washed 2 times with anoxic PBS buffer (10 mM NaPO_4_, 138 mM NaCl, 2.7 mM KCl, pH 7.4). After the final wash and centrifugation, cell pellets were suspended in 2 ml of anoxic PBS. A total volume of 2.5 ml was used for the assay. For each phase of growth, three separate dilutions were tested: 1.2, 6, and 12-fold. Each diluted sample received 1.9 ml of a 6.5 mM stock solution of CTC in PBS (final concentration approximately 5 mM CTC). Each dilution was done in triplicate.

CTC-treated samples were incubated at 37°C for 4 h with gentle agitation and kept in the dark. Samples were collected on a 25 mm diameter white polycarbonate filter with a 0.22 μm pore size. The filter was then placed in a vial containing 2.5 ml 95% EtOH and agitated for 12 h at room temperature in the dark. After 12 h, the EtOH was filtered through a 0.22 μm pore size filter and the solution absorbance was measured (450 nm). The amount of CTC-formazan within the solution was calculated using a molar extinction coefficient of 1.63 X 10^4^ M^-1^ cm^-1^ and the amount calculated was normalized to protein.

### Formate Dehydrogenase activity

Formate dehydrogenase activity was measured for biofilm and reactor planktonic cells using a methyl viologen reduction assay as described previously [59]. Briefly, biofilm biomass was collected by scraping 4 slides from the CDC reactor into Tris HCl buffer (50 mM Tris-HCl pH 8.0) within an anaerobic chamber. Suspended biofilm biomass was placed in a N_2_ gassed Hungate tube and pelleted via centrifugation at 2,000 x g at 4°C for 10 min. Planktonic biomass (10 ml) was collected from the CDC reactor and placed in a N_2_ gassed Hungate tube and pelleted as described. The collection tubes were pressurized with N_2_ and pellets were stored at -70°C until processed. Biofilm and planktonic pellets were suspended in 2 ml of Tris HCl buffer and pulse sonicated for 10 min. Supernatants were cleared by centrifugation at 9,400 x g for 30 min at 4°C followed by bubbling with N_2_ for 30 min to regenerate anoxic conditions. Supernatants were stored in N_2_ gassed Hungate tubes at -20°C until activity was measured (within 12 h after sonication).

Activity assays were done in a N_2_ flushed cuvette (10 min) that was sealed with a septum and components were added via a N_2_ flushed syringe. Initially, 0.6 ml of anoxic H_2_0, 0.1 ml of 500 mM Tris HCl pH 8.0, 0.1 ml of 1 M β mercaptoethanol, and 0.05 ml of 100 mM methyl viologen were incubated for 1 min at room temperature in the cuvette. Next, 0.05 ml of crude sample was added and the mixture was incubated for 5 min at room temperature. Last, 0.1 ml of 1 M sodium formate was added to start the reaction and the OD_578_ was measured immediately after a quick inversion to mix the components. Readings were taken every 5 s for a total of 8 min. Activity was determined using the molar extinction coefficient for methyl viologen at OD_578_ = 9.8 mM^-1^ cm ^-1^. Protein concentration of the crude extract was determined by the Bradford assay. Reduction of methyl viologen was minimal during initial incubation periods.

## Competing interests

The authors declare that they have no competing interest.

## Authors’ contributions

MEC carried out growth, biomass harvest, and activity assays, participated in array/protein data analysis, and drafted the manuscript. ZH carried out microarray hybridizations and data analysis. AMR carried out proteomic detection and data analysis. JMP and APA participated in data analysis and manuscript preparation. AM, JZ, JDK, and MWF participated in study design, study coordination, data analysis/interpretation, and manuscript preparation. All authors read and approved the final manuscript.

## Supplementary Material

Additional file 1 Confocal image of *D. vulgaris* biofilm stained with acridine orange. Click here for file

Additional file 2 Comparison of biofilm transcriptomic and proteomic data when both samples were normalized to batch, exponential-phase planktonic cells. Genes of interest are highlighted. Click here for file

Additional file 3 Whole genome transcript expression data for biofilm cells compared to planktonic, batch cells. Click here for file

Additional file 4 Whole genome transcript expression data for biofilm cells compared to planktonic, reactor cells. Click here for file

Additional file 5 Protein expression ratios between biofilm to batch, planktonic cells; biofilm to reactor, planktonic cells; or reactor, planktonic to batch, planktonic cells. Click here for file

Additional file 6 Expression and z values for 15 ribosomal protein genes. Expo1 and Expo2 represent exponential-phase cells and Stat1 through Stat4 represent stationary-phase cells. Blue and red colors denote level of expression for genes with a significant z score for up- and down-expression, respectively. Click here for file

Additional file 7 Analysis of enriched extracellular proteins from biofilms. Click here for file
