# Supplementary figures and images for: Transcriptomic and proteomic analyses of Desulfovibrio vulgaris biofilms: Carbon and energy flow contribute to the distinct biofilm growth state
Source: BMC Genomics. 2012 Apr 16;13:138. doi: 10.1186/1471-2164-13-138 (PMC3431258; doi:10.1186/1471-2164-13-138)

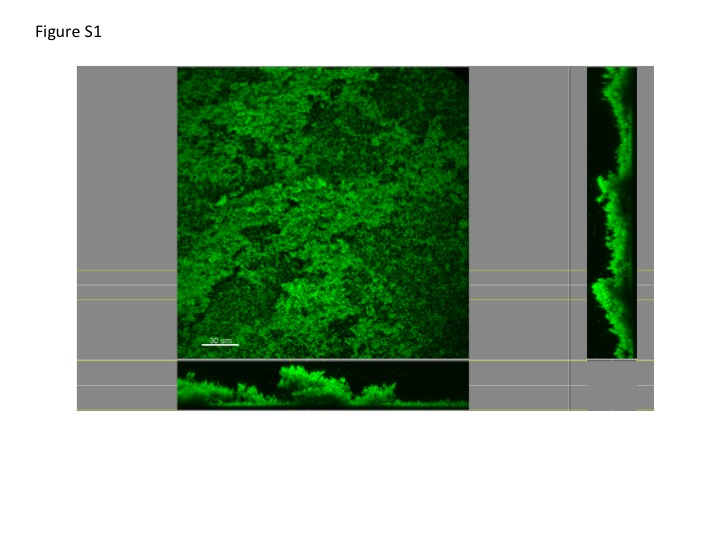

Supplement: Additional file 1 — Confocal image of D. vulgaris biofilm stained with acridine orange. [file 1471-2164-13-138-S1.jpeg]

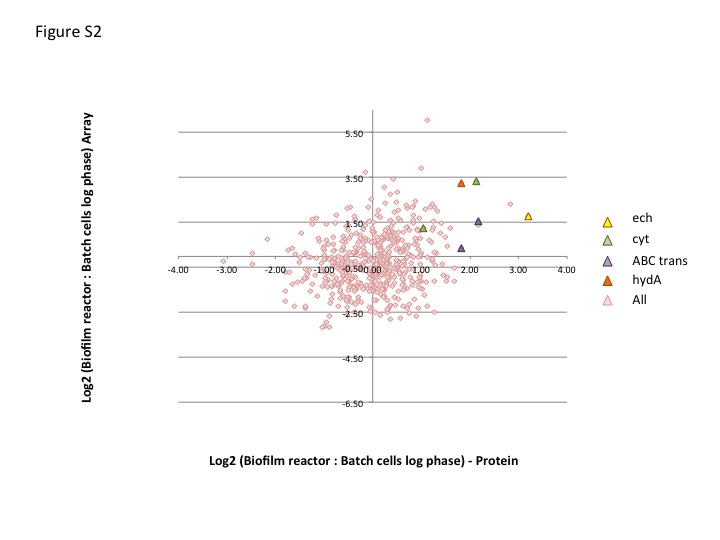

Supplement: Additional file 2 — Comparison of biofilm transcriptomic and proteomic data when both samples were normalized to batch, exponential-phase planktonic cells. Genes of interest are highlighted. [file 1471-2164-13-138-S2.jpeg]
