# Supplementary material for: Transcriptomic and proteomic analyses of Desulfovibrio vulgaris biofilms: Carbon and energy flow contribute to the distinct biofilm growth state
Source: BMC Genomics. 2012 Apr 16;13:138. doi: 10.1186/1471-2164-13-138 (PMC3431258; doi:10.1186/1471-2164-13-138)
Supplement: Additional file 3 — Whole genome transcript expression data for biofilm cells compared to planktonic, batch cells. [file 1471-2164-13-138-S3.pdf]

Additional file 3. Whole genome transcript expression data for biofilm cells compared to planktonic, batch cells.

Locus Id	Locus Tag	GI#	Mean Log Ratio	z score
VIMSS206065	DVU0646	46579060	-0.609446	-0.902932
VIMSS206066	DVU0647	46579061	0.27352	0.327413
VIMSS206067	DVU0648	46579062	-0.0246748	-0.0301761
VIMSS206069	DVU0649	46579063	0.179496	0.254675
VIMSS206070	DVU0650	46579064	-0.0206848	-0.0146579
VIMSS206071	DVU0651	46579065	-1.2427	-1.63214
VIMSS206072	DVU0652	46579066	0.664438	0.887133
VIMSS206074	DVU0653	46579067	-0.248998	-0.407924
VIMSS206075	DVU0654	46579068	-0.212501	-0.348404
VIMSS206076	DVU0655	46579069	0.317065	0.527664
VIMSS206078	DVU0656	46579070	0.372877	0.633853
VIMSS206079	DVU0657	46579071	0.319002	0.546644
VIMSS206081	DVU0659	46579073	0.68799	1.26189
VIMSS206083	DVU0660	46579074	0.287079	0.439353
VIMSS206084	DVU0661	46579075	-0.0553812	-0.0852273
VIMSS206085	DVU0662	46579076	0.0747512	0.126154
VIMSS206086	DVU0663	46579077	1.59064	3.02861
VIMSS206087	DVU0664	46579078	0.363469	0.621251
VIMSS206088	DVU0665	46579079	0.536706	0.919464
VIMSS206089	DVU0666	46579080	-0.10088	-0.162769
VIMSS206090	DVU0667	46579081	-0.684536	-1.17476
VIMSS206091	DVU0668	46579082	-0.384483	-0.544104
VIMSS206092	DVU0669	46579083	-1.17824	-2.26902
VIMSS206093	DVU0670	46579084	-0.784028	-1.1468
VIMSS206094	DVU0671	46579085	-2.19247	-3.47453
VIMSS206095	DVU0672	46579086	-1.72199	-1.63583
VIMSS206098	DVU0674	46579088	0.18294	0.331779
VIMSS206100	DVU0676	46579090	1.05607	1.66069
VIMSS206101	DVU0677	46579091	0.907193	1.63671
VIMSS206103	DVU0679	46579093	-0.473916	-0.616658
VIMSS206104	DVU0680	46579094	-0.0272281	-0.0364521
VIMSS206105	DVU0681	46579095	1.22131	1.78504
VIMSS206106			-0.00670685	-0.0112323
VIMSS206107	DVU0683	46579097	0.612396	1.12504
VIMSS206108			1.01194	1.60153
VIMSS206109	DVU0685	46579099	0.544305	0.889837
VIMSS206110	DVU0686	46579100	0.762989	1.14136
VIMSS206111	DVU0687	46579101	0.317092	0.51969
VIMSS206112	DVU0688	46579102	-0.531845	-0.743432
VIMSS206113	DVU0689	46579103	-0.237736	-0.396983
VIMSS206114	DVU0690	46579104	-0.252787	-0.413254
VIMSS206115	DVU0691	46579105	0.23303	0.336476
VIMSS206116	DVU0692	46579106	0.139512	0.243456

VIMSS206117	DVU0693	46579107	-0.944452	-1.35974
VIMSS206118	DVU0694	46579108	-0.926777	-1.15681
VIMSS206121	DVU0697	46579111	0.944853	1.60817
VIMSS206122	DVU0698	46579112	-0.230896	-0.383033
VIMSS206124	DVU0699		0.263389	0.4358
VIMSS206125	DVU0700	46579113	0.518434	0.739552
VIMSS206126	DVU0701	46579114	1.50243	2.63535
VIMSS206127	DVU0702	46579115	1.04865	1.44134
VIMSS206128	DVU0703	46579116	-0.86161	-1.26151
VIMSS206129	DVU0704	46579117	-0.918684	-1.69756
VIMSS206130	DVU0706	46579119	-0.292937	-0.470127
VIMSS206131	DVU0705	46579118	0.766116	0.905274
VIMSS206132	DVU0707	46579120	0.252623	0.472271
VIMSS206135	DVU0710	46579123	0.150977	0.102361
VIMSS206136	DVU0711	46579124	0.590649	0.857744
VIMSS206137	DVU0712	46579125	2.01488	3.3365
VIMSS206138	DVU0713	46579126	0.261848	0.35852
VIMSS206139	DVU0714	46579127	0.215198	0.317172
VIMSS206140	DVU0715	46579128	-0.776514	-1.03143
VIMSS206141	DVU0716	46579129	-0.794704	-1.26012
VIMSS206142	DVU0717	46579130	0.105959	0.145414
VIMSS206145	DVU0720	46579133	0.264866	0.403104
VIMSS206146			1.31923	1.91308
VIMSS206147	DVU0722	46579135	1.00861	0.984116
VIMSS206149	DVU0723	46579136	-0.631829	-0.914996
VIMSS206150	DVU0724	46579137	-1.30074	-2.22792
VIMSS206151	DVU0725	46579138	1.05737	1.62268
VIMSS206152	DVU0726	46579139	-0.677884	-1.07742
VIMSS206153	DVU0727	46579140	-0.170157	-0.264855
VIMSS206154	DVU0728	46579141	0.542163	0.659468
VIMSS206155	DVU0729	46579142	1.60774	2.95388
VIMSS206156	DVU0730	46579143	0.151391	0.268219
VIMSS206157	DVU0731	46579144	0.580263	0.684529
VIMSS206158	DVU0732	46579145	-1.04182	-1.47992
VIMSS206159	DVU0733	46579146	0.331601	0.479131
VIMSS206160	DVU0734	46579147	-0.861656	-1.30533
VIMSS206161	DVU0735	46579148	0.225296	0.320706
VIMSS206162	DVU0736	46579149	0.771916	1.10384
VIMSS206163	DVU0737	46579150	-0.0971069	-0.136156
VIMSS206164	DVU0738	46579151	1.2993	2.23188
VIMSS206165	DVU0739	46579152	1.38251	1.68657
VIMSS206166	DVU0740	46579153	-0.642447	-0.833605
VIMSS206167	DVU0741	46579154	1.35021	2.41461
VIMSS206168	DVU0742	46579155	1.50597	2.28199
VIMSS206169	DVU0743	46579156	0.221871	0.295969
VIMSS206170	DVU0744	46579157	0.355459	0.604653
VIMSS206171	DVU0745	46579158	-0.757596	-0.944974
VIMSS206172	DVU0746	46579159	-1.02236	-1.22895
VIMSS206173	DVU0747	46579160	-0.0858771	-0.108851

VIMSS206175	DVU0749	46579162	-0.305531	-0.326241
VIMSS206176	DVU0750	46579163	0.614523	0.895703
VIMSS206177	DVU0751	46579164	0.685117	0.796821
VIMSS206178	DVU0752	46579165	0.370237	0.580734
VIMSS206179	DVU0753	46579166	-0.332793	-0.443324
VIMSS206180	DVU0754	46579167	1.74166	3.04567
VIMSS206181	DVU0755	46579168	1.45771	2.04807
VIMSS206182	DVU0756	46579169	0.627007	0.902691
VIMSS206183	DVU0757	46579170	-0.547132	-0.684512
VIMSS206184	DVU0758	46579171	1.95466	2.62168
VIMSS206185	DVU0759	46579172	-0.0177318	-0.0275085
VIMSS206186	DVU0760	46579173	0.389518	0.52504
VIMSS206187	DVU0761	46579174	-0.194029	-0.25549
VIMSS206188	DVU0762	46579175	0.79557	1.371
VIMSS206189	DVU0763	46579176	-1.44312	-1.67582
VIMSS206190			-2.80845	-4.67068
VIMSS206191	DVU0765	46579178	-0.303991	-0.368745
VIMSS206193	DVU0766	46579179	1.45543	1.94696
VIMSS206194	DVU0767	46579180	-0.735283	-1.17024
VIMSS206195	DVU0768	46579181	-0.465395	-0.561554
VIMSS206196	DVU0769	46579182	-0.603308	-0.915367
VIMSS206197	DVU0770	46579183	-0.351869	-0.36147
VIMSS206198	DVU0771	46579184	-0.306474	-0.429395
VIMSS206199	DVU0772	46579185	0.601079	0.564306
VIMSS206200	DVU0773	46579186	2.69773	3.95357
VIMSS206201	DVU0774	46579187	-1.1071	-1.26503
VIMSS206202	DVU0775	46579188	-0.735086	-0.885375
VIMSS206203	DVU0776	46579189	-1.18624	-1.16966
VIMSS206204	DVU0777	46579190	-0.694746	-0.708675
VIMSS206205	DVU0778	46579191	-1.89234	-2.51924
VIMSS206206	DVU0779	46579192	-1.425	-1.83323
VIMSS206207	DVU0780	46579193	-0.853696	-1.32082
VIMSS206211	DVU0784	46579197	-1.10505	-1.36996
VIMSS206212	DVU0785	46579198	-0.24528	-0.35504
VIMSS206213	DVU0786	46579199	-0.0304676	-0.0386782
VIMSS206214	DVU0787	46579200	-0.0842764	-0.120614
VIMSS206215	DVU0788	46579201	-1.37201	-1.56925
VIMSS206216	DVU0789	46579202	-0.0972556	-0.16489
VIMSS206217	DVU0790	46579203	-1.16269	-1.5621
VIMSS206218	DVU0791	46579204	-0.267287	-0.437328
VIMSS206219	DVU0792	46579205	0.0596027	0.0735075
VIMSS206220	DVU0793	46579206	-0.65308	-1.03711
VIMSS206221	DVU0794	46579207	-1.2071	-1.50234
VIMSS206222	DVU0795	46579208	-1.82364	-2.13889
VIMSS206223	DVU0796	46579209	-1.24103	-1.67549
VIMSS206224	DVU0798	46579211	-0.748718	-0.822841
VIMSS206225	DVU0799	46579212	2.31848	3.77833
VIMSS206227	DVU0801	46579214	0.116062	0.125608
VIMSS206228	DVU0802	46579215	-1.82659	-1.97061

VIMSS206229	DVU0803	46579216	0.609673	0.752367
VIMSS206230	DVU0804	46579217	1.12086	1.02128
VIMSS206231	DVU0805	46579218	0.0636254	0.0856305
VIMSS206232	DVU0806	46579219	0.658636	1.15646
VIMSS206233	DVU0807	46579220	-0.671519	-0.892995
VIMSS206234	DVU0808	46579221	-0.301631	-0.46479
VIMSS206235	DVU0809	46579222	-0.459329	-0.688513
VIMSS206236	DVU0810	46579223	0.187541	0.324391
VIMSS206238	DVU0811	46579224	0.739273	1.0953
VIMSS206239	DVU0812	46579225	0.704672	1.03015
VIMSS206240	DVU0813	46579226	1.09979	1.81845
VIMSS206241	DVU0814	304569687	0.0155227	0.0202612
VIMSS206242	DVU0815	46579228	0.589533	1.03207
VIMSS206243	DVU0816	46579229	0.233209	0.30801
VIMSS206244	DVU0817	46579230	-0.943748	-1.71256
VIMSS206245	DVU0818	46579231	-0.860724	-1.10511
VIMSS206246	DVU0819	46579232	1.06786	1.86959
VIMSS206248	DVU0821	46579234	0.161516	0.236405
VIMSS206249	DVU0822	46579235	0.1719	0.222171
VIMSS206250	DVU0823	46579236	-0.329644	-0.474319
VIMSS206252	DVU0825	46579238	-0.0939562	-0.153821
VIMSS206253	DVU0826	46579239	-0.356341	-0.392303
VIMSS206254	DVU0827	46579240	-0.792206	-1.44355
VIMSS206256	DVU0828	46579241	-0.313215	-0.361911
VIMSS206257	DVU0829	46579242	0.158725	0.275347
VIMSS206258	DVU0830	46579243	-0.348639	-0.438165
VIMSS206259	DVU0831	46579244	0.266183	0.410346
VIMSS206260	DVU0832	46579245	-0.508164	-0.699507
VIMSS206261	DVU0834	46579247	-0.326458	-0.41891
VIMSS206262	DVU0835	46579248	-1.39349	-1.93051
VIMSS206263	DVU0836	46579249	-1.21172	-1.87778
VIMSS206264	DVU0837	46579250	-0.23522	-0.318993
VIMSS206265	DVU0838	46579251	-0.34101	-0.557237
VIMSS206266	DVU0839	46579252	-0.914385	-1.5167
VIMSS206267	DVU0840	46579253	-1.53364	-2.49507
VIMSS206268	DVU0841	46579254	-0.130066	-0.206239
VIMSS206269	DVU0842	46579255	-0.92994	-1.49278
VIMSS206270	DVU0843	304569688	-0.0697755	-0.102502
VIMSS206272	DVU0846	46579259	0.0241966	0.0330458
VIMSS206274	DVU0847	46579260	-0.632561	-0.770207
VIMSS206275	DVU0848	46579261	-1.2589	-1.64089
VIMSS206276	DVU0849	46579262	-1.28556	-1.90243
VIMSS206277	DVU0850	46579263	-1.44009	-1.88692
VIMSS206279	DVU0852	46579265	-0.522312	-0.50059
VIMSS206280			0.525213	0.871916
VIMSS206281	DVU0854	46579267	-0.454207	-0.659092
VIMSS206282			-0.329631	-0.495064
VIMSS206283	DVU0856	46579269	-1.22948	-1.73506
VIMSS206285	DVU0857	46579270	0.0148237	0.0226189

VIMSS206286	DVU0858	46579271	0.839631	0.93837
VIMSS206287			0.842313	1.14809
VIMSS206289	DVU0861	46579274	0.175063	0.200633
VIMSS206290	DVU0862	46579275	0.613193	0.882854
VIMSS206291	DVU0863	46579276	-0.310013	-0.534978
VIMSS206292	DVU0864	46579277	-0.139785	-0.212937
VIMSS206293	DVU0865	46579278	0.077914	0.127061
VIMSS206294	DVU0866	46579279	-0.432507	-0.740564
VIMSS206295	DVU0867	46579280	-0.351718	-0.53768
VIMSS206296	DVU0868	46579281	0.135293	0.219019
VIMSS206297	DVU0869	46579282	-0.197364	-0.299939
VIMSS206298	DVU0870	46579283	0.391621	0.6872
VIMSS206299	DVU0871	46579284	-1.33596	-2.33274
VIMSS206300	DVU0872	46579285	0.785238	1.17475
VIMSS206301	DVU0873	46579286	-2.25894	-3.15543
VIMSS206302	DVU0874	46579287	-1.47142	-2.0978
VIMSS206303	DVU0875	46579288	0.352333	0.43806
VIMSS206304	DVU0876	46579289	0.775969	1.22498
VIMSS206306	DVU0878	46579291	2.3757	3.41734
VIMSS206308			-1.84919	-2.71731
VIMSS206309	DVU0881	46579294	1.38238	2.04187
VIMSS206310	DVU0882	46579295	1.48955	2.3764
VIMSS206311	DVU0883	46579296	1.1272	2.00722
VIMSS206312	DVU0884	46579297	0.704822	1.00631
VIMSS206313	DVU0885	46579298	-0.656385	-0.962527
VIMSS206314	DVU0886	46579299	-0.352874	-0.491024
VIMSS206316	DVU0888	46579301	1.01246	1.5677
VIMSS206318	DVU0890	46579303	-0.601212	-0.936668
VIMSS206319	DVU0891	46579304	-0.970654	-1.57077
VIMSS206320	DVU0892	46579305	0.867531	1.24002
VIMSS206321	DVU0893	46579306	-0.207813	-0.387266
VIMSS206323	DVU0895	46579308	-0.291703	-0.454624
VIMSS206324	DVU0896	46579309	0.507495	0.813426
VIMSS206325	DVU0897	46579310	0.160893	0.229791
VIMSS206327	DVU0899	46579312	0.0610005	0.100641
VIMSS206328	DVU0900	46579313	-0.197235	-0.278044
VIMSS206329	DVU0901	46579314	-0.0110612	-0.0129965
VIMSS206330	DVU0902	46579315	-0.328203	-0.459656
VIMSS206331	DVU0903	46579316	0.527891	0.799313
VIMSS206332	DVU0904	46579317	0.271065	0.292704
VIMSS206333	DVU0905	46579318	-0.365005	-0.514757
VIMSS206334	DVU0906	46579319	0.268302	0.310752
VIMSS206335	DVU0907	46579320	-0.109433	-0.151364
VIMSS206336	DVU0908	46579321	-1.17489	-1.58471
VIMSS206337	DVU0909	46579322	0.621726	0.890696
VIMSS206338	DVU0910	46579323	1.23168	2.09443
VIMSS206339	DVU0911	46579324	-1.98314	-3.07417
VIMSS206340	DVU0912	46579325	0.363147	0.630309
VIMSS206341	DVU0913	46579326	0.0523159	0.0882077

VIMSS206343	DVU0914	46579327	-0.260172	-0.336229
VIMSS206344	DVU0915	46579328	0.767206	1.19831
VIMSS206345	DVU0916	46579329	0.13648	0.223548
VIMSS206347	DVU0917	46579330	1.01669	1.59251
VIMSS206348	DVU0918	46579331	0.33205	0.552099
VIMSS206349	DVU0919	46579332	-0.176128	-0.319044
VIMSS206350			0.364344	0.438434
VIMSS206351	DVU0921	46579334	0.424479	0.398307
VIMSS206352	DVU0922	46579335	0.324977	0.375169
VIMSS206353	DVU0923	46579336	-0.750702	-1.05715
VIMSS206355	DVU0924	46579337	-0.447008	-0.539418
VIMSS206356	DVU0925	46579338	-0.221111	-0.307725
VIMSS206357	DVU0926	46579339	-0.9151	-1.10367
VIMSS206358	DVU0927	46579340	-1.42935	-2.10217
VIMSS206359	DVU0928	46579341	-1.74224	-2.9964
VIMSS206360	DVU0929	46579342	-0.704538	-0.840002
VIMSS206361	DVU0930	46579343	-0.226817	-0.320839
VIMSS206362	DVU0931	46579344	-0.371858	-0.555918
VIMSS206363	DVU0932	46579345	0.557458	0.912092
VIMSS206364	DVU0933	46579346	0.20601	0.291105
VIMSS206365	DVU0934	46579347	-0.236323	-0.298316
VIMSS206366	DVU0935	46579348	1.62106	2.24016
VIMSS206367			-1.56616	-2.05629
VIMSS206368	DVU0937	46579350	0.223364	0.289939
VIMSS206369	DVU0938	46579351	1.54589	2.29339
VIMSS206370	DVU0939	46579352	1.24694	1.96879
VIMSS206371	DVU0940	46579353	-0.252335	-0.275658
VIMSS206373	DVU0941	46579354	0.0181882	0.0302183
VIMSS206374	DVU0942	46579355	0.732327	0.990614
VIMSS206375			0.425083	0.496023
VIMSS206376	DVU0944	46579357	0.723928	0.934407
VIMSS206377	DVU0945	46579358	0.889057	1.27517
VIMSS206378	DVU0946	46579359	0.23339	0.394192
VIMSS206379	DVU0947	46579360	-0.383927	-0.486249
VIMSS206380	DVU0948	46579361	-0.617781	-0.628359
VIMSS206381	DVU0949	46579362	-1.17373	-1.21868
VIMSS206383			-0.867772	-1.15878
VIMSS206384	DVU0952	46579365	-0.0670207	-0.112797
VIMSS206385	DVU0953	46579366	-1.5038	-1.7696
VIMSS206386	DVU0954	46579367	-0.501532	-0.585525
VIMSS206387	DVU0955	46579368	0.587751	0.879576
VIMSS206388	DVU0956	46579369	-1.00674	-1.53544
VIMSS206389	DVU0957	46579370	-1.57265	-2.02306
VIMSS206390	DVU0958	46579371	-1.23631	-1.93627
VIMSS206391	DVU0959	46579372	-2.27103	-2.75062
VIMSS206393	DVU0961	46579374	0.986643	1.34702
VIMSS206395	DVU0963	46579376	0.76733	1.14047
VIMSS206398	DVU0966	46579379	-0.537221	-0.945275
VIMSS206399	DVU0967	46579380	-0.691927	-1.02163

VIMSS206400	DVU0968	46579381	-0.371171	-0.525444
VIMSS206401	DVU0969	46579382	0.673948	0.89813
VIMSS206402	DVU0970	46579383	0.470889	0.667233
VIMSS206403	DVU0971	304569689	1.27482	1.68244
VIMSS206404	DVU0972	46579385	-0.13965	-0.150438
VIMSS206405	DVU0973	46579386	-0.247136	-0.24925
VIMSS206406	DVU0974	46579387	0.357593	0.577152
VIMSS206407	DVU0975	46579388	0.214675	0.283378
VIMSS206408	DVU0976	46579389	1.28134	1.89324
VIMSS206410	DVU0978	46579391	-0.021141	-0.0325617
VIMSS206411	DVU0979	46579392	1.81109	2.46229
VIMSS206412	DVU0980	46579393	1.29242	1.89949
VIMSS206413	DVU0981	46579394	1.22258	1.49941
VIMSS206414	DVU0982	46579395	0.513401	0.826537
VIMSS206415	DVU0983	46579396	0.698357	1.01987
VIMSS206416	DVU0984	46579397	0.49043	0.843354
VIMSS206418	DVU0987	46579400	1.30137	1.8788
VIMSS206420	DVU0988	46579401	-0.108792	-0.169847
VIMSS206421			0.340162	0.483964
VIMSS206422	DVU0990	46579403	-0.678579	-1.0324
VIMSS206423	DVU0991	46579404	-0.914599	-1.20512
VIMSS206424	DVU0992	46579405	0.816741	1.22167
VIMSS206425	DVU0993	46579406	-0.402661	-0.55196
VIMSS206426	DVU0994	46579407	-0.168035	-0.216565
VIMSS206427	DVU0995	46579408	1.81975	2.88441
VIMSS206428	DVU0996	46579409	0.400778	0.482733
VIMSS206429	DVU0997	46579410	0.364843	0.526808
VIMSS206430	DVU0998	46579411	0.356501	0.541754
VIMSS206433	DVU0999	46579412	-0.0166109	-0.0179037
VIMSS206434	DVU1000	46579413	-0.14656	-0.172405
VIMSS206435	DVU1001	46579414	0.179364	0.257522
VIMSS206436	DVU1002	46579415	0.399391	0.546049
VIMSS206437	DVU1003	46579416	0.378256	0.591762
VIMSS206438	DVU1004	46579417	1.51319	1.87859
VIMSS206439	DVU1005	46579418	0.902085	1.60559
VIMSS206440	DVU1006	46579419	0.355966	0.49571
VIMSS206441	DVU1007	46579420	0.521348	0.668326
VIMSS206442	DVU1008	46579421	-0.358256	-0.45836
VIMSS206443	DVU1009	46579422	-0.329659	-0.521092
VIMSS206446	DVU1012	46579425	1.55175	2.11465
VIMSS206447	DVU1013	46579426	2.03527	3.25398
VIMSS206451	DVU1017	46579430	-0.309239	-0.399761
VIMSS206452	DVU1018	304569690	-0.051561	-0.0621318
VIMSS206453	DVU1019	46579432	-0.485747	-0.735945
VIMSS206454	DVU1020	46579433	0.025646	0.0276472
VIMSS206455	DVU1021	46579434	-0.990003	-1.32269
VIMSS206456	DVU1022	46579435	-1.45453	-1.91134
VIMSS206458	DVU1024	46579437	-0.404317	-0.47613
VIMSS206459	DVU1025	46579438	-0.316662	-0.466937

VIMSS206460	DVU1026	46579439	-0.0544503	-0.0570638
VIMSS206461	DVU1027	46579440	0.322843	0.46251
VIMSS206462	DVU1028	46579441	-0.0430197	-0.0697221
VIMSS206463	DVU1029	46579442	-2.97339	-5.3232
VIMSS206465	DVU1030	46579443	0.852488	1.43024
VIMSS206466	DVU1032	46579445	0.235315	0.237735
VIMSS206467	DVU1033	46579446	0.96912	1.7023
VIMSS206468	DVU1034	46579447	-0.0302064	-0.0382695
VIMSS206469	DVU1035	304569691	2.31188	4.17586
VIMSS206470	DVU1036	46579449	-0.0603905	-0.0607181
VIMSS206471	DVU1037	46579450	0.445938	0.782211
VIMSS206472	DVU1038	46579451	-1.08976	-1.80698
VIMSS206473	DVU1039	46579452	-0.68513	-1.2258
VIMSS206474	DVU1040	304569692	-0.490891	-0.884601
VIMSS206475	DVU1041	46579454	-0.387745	-0.639409
VIMSS206476	DVU1042	46579455	-0.977095	-1.5071
VIMSS206477	DVU1043	46579456	-0.599234	-0.958642
VIMSS206478	DVU1044	46579457	-0.31998	-0.2102
VIMSS206479	DVU1045	46579458	-0.0590766	-0.0942439
VIMSS206480	DVU1046	46579459	-0.00640225	-0.00902818
VIMSS206481	DVU1047	46579460	0.200514	0.350163
VIMSS206482	DVU1048	46579461	-0.329581	-0.48822
VIMSS206483	DVU1049	46579462	-0.0376929	-0.0580564
VIMSS206484	DVU1050	46579463	0.828683	1.10812
VIMSS206485	DVU1051	46579464	0.40049	0.69237
VIMSS206488	DVU1054	46579467	-0.766799	-1.14975
VIMSS206489	DVU1055	46579468	-0.855913	-1.16655
VIMSS206490	DVU1056	46579469	-0.568826	-0.830255
VIMSS206491	DVU1057	46579470	0.750296	0.871431
VIMSS206492	DVU1058	46579471	0.778091	1.03002
VIMSS206494	DVU1060	46579473	-1.25606	-1.74415
VIMSS206495	DVU1061	46579474	-0.0731012	-0.110299
VIMSS206496	DVU1062	46579475	-0.559792	-0.957112
VIMSS206497			-0.0957536	-0.145498
VIMSS206498	DVU1064	46579477	-0.251939	-0.433332
VIMSS206499	DVU1065	46579478	-1.54506	-2.72201
VIMSS206500	DVU1066	46579479	-0.67326	-1.14364
VIMSS206501	DVU1067	46579480	0.0522557	0.0960638
VIMSS206502	DVU1068	46579481	0.992762	1.07086
VIMSS206503	DVU1069	46579482	-0.98192	-1.3623
VIMSS206505	DVU1070	304569693	0.0714137	0.107893
VIMSS206506	DVU1071	46579484	-0.334935	-0.465622
VIMSS206507	DVU1072	46579485	0.207857	0.309731
VIMSS206508	DVU1073	46579486	2.25294	3.87907
VIMSS206509	DVU1074	46579487	-2.20572	-3.09515
VIMSS206510	DVU1075	46579488	-0.916176	-1.6233
VIMSS206511	DVU1076	46579489	-0.750861	-1.15816
VIMSS206512	DVU1077	46579490	-1.46544	-2.48822

VIMSS206514	DVU1079	46579492	-0.235371	-0.283987
VIMSS206515	DVU1080	46579493	1.40182	1.94918
VIMSS206516	DVU1081	46579494	-1.84806	-2.93632
VIMSS206517	DVU1082	46579495	-1.37951	-2.26467
VIMSS206518	DVU1083	46579496	1.67032	2.88431
VIMSS206519	DVU1084	46579497	0.0967903	0.143984
VIMSS206520	DVU1085	46579498	-0.286975	-0.43015
VIMSS206521			0.752799	1.33024
VIMSS206522	DVU1087	46579500	1.66851	2.21374
VIMSS206523	DVU1088	46579501	1.19262	1.77123
VIMSS206524	DVU1089	46579502	-0.975461	-1.42165
VIMSS206525	DVU1090	46579503	-1.01633	-1.68707
VIMSS206526	DVU1091	46579504	-0.345127	-0.506644
VIMSS206527	DVU1092	46579505	-0.88677	-1.36641
VIMSS206528	DVU1093	46579506	1.18057	2.05205
VIMSS206529	DVU1094	46579507	-1.1099	-1.89576
VIMSS206530	DVU1095	46579508	-2.00114	-2.27717
VIMSS206531	DVU1096	46579509	-0.699936	-1.07683
VIMSS206532	DVU1097	46579510	0.126576	0.212341
VIMSS206533	DVU1098	46579511	0.897053	1.58162
VIMSS206534	DVU1099	46579512	-0.0847221	-0.122085
VIMSS206535	DVU1100	46579513	-0.751219	-0.866237
VIMSS206538	DVU1103	46579516	0.87989	1.36207
VIMSS206539	DVU1104	46579517	0.649673	0.805323
VIMSS206540	DVU1105	46579518	0.511376	0.647726
VIMSS206541	DVU1106	46579519	2.37947	3.52529
VIMSS206542	DVU1107	46579520	-0.0470161	-0.0531719
VIMSS206544	DVU1108	46579521	-0.902006	-1.29843
VIMSS206545	DVU1109	46579522	-0.858432	-0.912635
VIMSS206546	DVU1110	46579523	-1.92626	-2.47688
VIMSS206547	DVU1111	46579524	-1.39712	-2.02545
VIMSS206549	DVU1113	46579526	-0.915893	-1.14832
VIMSS206550	DVU1114	46579527	-0.0828933	-0.0985856
VIMSS206551	DVU1115	46579528	1.99506	2.92971
VIMSS206552	DVU1116	46579529	1.21314	1.46405
VIMSS206553	DVU1117	46579530	0.263287	0.406268
VIMSS206554	DVU1118	46579531	1.25709	1.39235
VIMSS206556	DVU1120	46579533	0.401979	0.474723
VIMSS206557	DVU1121	46579534	1.7283	3.03426
VIMSS206558	DVU1122	46579535	0.528304	0.620536
VIMSS206559	DVU1123	46579536	2.8057	3.11366
VIMSS206560	DVU1125	46579538	1.8283	2.97536
VIMSS206561	DVU1124	46579537	0.818125	0.930257
VIMSS206562	DVU1126	46579539	-0.125451	-0.112823
VIMSS206564	DVU1128	46579541	0.674253	0.844169
VIMSS206565	DVU1129	46579542	0.566242	0.417133
VIMSS206566	DVU1130	46579543	1.78697	2.24758
VIMSS206567	DVU1131	46579544	0.564902	0.578472
VIMSS206568	DVU1132	46579545	2.11593	3.1243

VIMSS206569	DVU1133	46579546	0.939144	1.15318
VIMSS206570	DVU1134	46579547	0.698619	1.10721
VIMSS206571	DVU1135	46579548	1.50251	1.83404
VIMSS206572	DVU1136	46579549	1.16432	2.04567
VIMSS206573	DVU1137	46579550	1.01362	1.15784
VIMSS206574	DVU1138	46579551	1.02955	1.4057
VIMSS206575	DVU1139	46579552	1.51717	1.66158
VIMSS206576			1.18622	1.72098
VIMSS206577	DVU1141	46579554	0.670857	0.805133
VIMSS206578	DVU1142	46579555	1.63608	2.04089
VIMSS206579	DVU1143	46579556	3.053	3.6076
VIMSS206580	DVU1144	46579557	-0.335976	-0.576598
VIMSS206582	DVU1145	46579558	-0.0245743	-0.032128
VIMSS206585	DVU1147	46579559	-1.99833	-2.17867
VIMSS206589	DVU1151	46579562	-0.166563	-0.170416
VIMSS206591	DVU1153	46579564	0.281753	0.3799
VIMSS206592	DVU1154	46579565	0.215471	0.315949
VIMSS206593	DVU1155	46579566	-0.651917	-0.824979
VIMSS206595	DVU1157	46579568	-0.996981	-0.969725
VIMSS206596	DVU1158	46579569	0.768292	0.7543
VIMSS206597	DVU1159	46579570	-0.776315	-0.973814
VIMSS206598	DVU1160	46579571	-0.367156	-0.549277
VIMSS206599	DVU1161	46579572	0.0817956	0.0936644
VIMSS206600	DVU1162	46579573	0.153186	0.17375
VIMSS206601	DVU1163	46579574	0.0842294	0.110219
VIMSS206602	DVU1164	46579575	0.655326	0.982518
VIMSS206603	DVU1165	46579576	0.477926	0.680299
VIMSS206604	DVU1166	46579577	-1.90488	-2.54526
VIMSS206608	DVU1168	46579579	0.226183	0.33791
VIMSS206609	DVU1169	46579580	0.644029	0.973822
VIMSS206610	DVU1170	46579581	-2.74499	-4.58299
VIMSS206613	DVU1173	46579584	-0.346826	-0.508113
VIMSS206614	DVU1174	46579585	0.321654	0.545738
VIMSS206615			-0.105571	-0.136589
VIMSS206616	DVU1176	46579587	-1.15005	-1.54906
VIMSS206617	DVU1177	46579588	-1.34452	-1.44724
VIMSS206619	DVU1179	46579590	1.97422	2.39756
VIMSS206620	DVU1180	46579591	0.589153	0.953101
VIMSS206621	DVU1181	46579592	-0.318038	-0.381295
VIMSS206622	DVU1182	46579593	0.134294	0.243351
VIMSS206624	DVU1185	46579596	-0.525036	-0.887375
VIMSS206625	DVU1186	46579597	-0.893418	-1.23403
VIMSS206626	DVU1187	46579598	-0.70583	-1.12548
VIMSS206627	DVU1188	46579599	0.437213	0.680081
VIMSS206628	DVU1189	46579600	0.348116	0.557309
VIMSS206629	DVU1190	46579601	1.11275	1.73139
VIMSS206630	DVU1191	46579602	-0.538719	-0.848457
VIMSS206631	DVU1192	46579603	1.05081	1.40808
VIMSS206632	DVU1193	46579604	-0.0116167	-0.0204954

VIMSS206633			-0.341868	-0.543102
VIMSS206634	DVU1195	46579606	-0.952578	-1.35314
VIMSS206635	DVU1196	46579607	-0.685464	-0.9815
VIMSS206636	DVU1197	46579608	-1.046	-1.67239
VIMSS206637	DVU1198	46579609	-1.2387	-1.81306
VIMSS206638	DVU1199	46579610	-2.10672	-3.28
VIMSS206639	DVU1200	46579611	-0.201561	-0.208685
VIMSS206640	DVU1201	46579612	-0.115133	-0.21195
VIMSS206641	DVU1202	46579613	-0.83048	-1.1539
VIMSS206642	DVU1203	46579614	-0.966248	-1.48618
VIMSS206643	DVU1204	46579615	-2.31603	-2.13256
VIMSS206644	DVU1205	46579616	-1.73031	-2.47899
VIMSS206645			-1.59539	-2.13364
VIMSS206646	DVU1207	46579618	-2.21916	-3.47775
VIMSS206648	DVU1209	46579620	-0.748683	-1.14257
VIMSS206650	DVU1211	46579622	0.475172	0.506907
VIMSS206651	DVU1212	46579623	0.626285	0.78245
VIMSS206652	DVU1213	46579624	-0.390521	-0.391179
VIMSS206653			-0.0853644	-0.129531
VIMSS206654			-0.595437	-0.78194
VIMSS206656	DVU1217	46579628	-0.38907	-0.567162
VIMSS206657	DVU1218	46579629	0.41865	0.702161
VIMSS206658	DVU1219	46579630	0.629579	0.9545
VIMSS206659	DVU1220	46579631	-1.09888	-1.83067
VIMSS206660	DVU1221	46579632	-0.104827	-0.152381
VIMSS206661	DVU1222	46579633	-0.465396	-0.671821
VIMSS206662			-0.592511	-0.818658
VIMSS206663	DVU1224	46579635	-0.468371	-0.591279
VIMSS206664	DVU1226	46579637	-1.03064	-1.17905
VIMSS206665	DVU1225	46579636	-0.611078	-0.891678
VIMSS206667	DVU1228	46579639	0.294381	0.515062
VIMSS206669	DVU1230	46579641	0.077603	0.0799668
VIMSS206670	DVU1231	46579642	-0.9212	-1.32653
VIMSS206671	DVU1232	46579643	0.18386	0.303624
VIMSS206672	DVU1233	46579644	0.535203	0.600732
VIMSS206674	DVU1235	46579646	-0.227418	-0.30766
VIMSS206675	DVU1236	46579647	-1.10274	-1.50376
VIMSS206676	DVU1237	46579648	-0.0556368	-0.0811343
VIMSS206677	DVU1238	46579649	-0.300657	-0.449638
VIMSS206678	DVU1239	46579650	0.771686	1.18547
VIMSS206679	DVU1240	46579651	-0.195392	-0.269522
VIMSS206680	DVU1241	46579652	-0.326047	-0.55334
VIMSS206681	DVU1242	46579653	-0.193023	-0.334473
VIMSS206682	DVU1243	46579654	-0.325099	-0.403672
VIMSS206683	DVU1244	46579655	1.09006	1.96705
VIMSS206684	DVU1245	46579656	-1.07713	-1.90647
VIMSS206685	DVU1246	46579657	1.16395	1.56245
VIMSS206686	DVU1247	46579658	-1.33988	-2.37289
VIMSS206687	DVU1248	46579659	-0.190762	-0.319882

VIMSS206688	DVU1249	46579660	-1.10538	-1.58326
VIMSS206689	DVU1250	46579661	-0.748734	-1.0161
VIMSS206690	DVU1251	46579662	-0.878433	-1.23354
VIMSS206691	DVU1252	46579663	0.0409358	0.0581478
VIMSS206692	DVU1253	46579664	-0.229731	-0.383951
VIMSS206693	DVU1254	46579665	-0.400049	-0.552816
VIMSS206694	DVU1255	46579666	-1.00116	-1.22867
VIMSS206695			-1.09677	-1.67667
VIMSS206697	DVU1258	46579669	0.0796442	0.123466
VIMSS206699	DVU1260	46579671	-1.53718	-2.29639
VIMSS206700	DVU1261	46579672	-1.4116	-2.23104
VIMSS206701	DVU1262	46579673	0.30648	0.571319
VIMSS206702	DVU1263	46579674	0.489997	0.684817
VIMSS206703	DVU1264	46579675	-0.527281	-0.617249
VIMSS206704	DVU1265	46579676	-0.19112	-0.250515
VIMSS206705	DVU1266	46579677	0.619165	0.940643
VIMSS206706	DVU1267	46579678	0.955304	1.72064
VIMSS206708	DVU1268	46579679	1.36538	1.28221
VIMSS206709	DVU1270	46579681	-0.25074	-0.312895
VIMSS206710	DVU1271	46579682	-1.07891	-1.56832
VIMSS206711	DVU1272	46579683	-0.421228	-0.520595
VIMSS206712	DVU1273	46579684	0.312242	0.525694
VIMSS206713	DVU1274	46579685	-0.333013	-0.605953
VIMSS206714	DVU1275	46579686	-0.447667	-0.669459
VIMSS206715	DVU1276	46579687	-1.47457	-2.21721
VIMSS206716	DVU1277	46579688	-1.55804	-2.03611
VIMSS206718	DVU1278	46579689	0.315369	0.367627
VIMSS206719	DVU1279	46579690	0.890132	1.46723
VIMSS206720	DVU1280	46579691	-1.56928	-2.44453
VIMSS206721	DVU1281	46579692	-0.464571	-0.789297
VIMSS206723	DVU1282	46579693	-0.148693	-0.229289
VIMSS206724	DVU1283	46579694	0.696419	1.06649
VIMSS206725	DVU1284	46579695	-0.445325	-0.551921
VIMSS206726			-0.12705	-0.211312
VIMSS206727	DVU1286	46579697	0.374355	0.664523
VIMSS206728	DVU1287	46579698	-0.151097	-0.236038
VIMSS206729	DVU1288	46579699	-0.883106	-1.05291
VIMSS206730	DVU1289	46579700	-1.40881	-1.75252
VIMSS206732			-0.810796	-1.46488
VIMSS206733	DVU1292	46579703	0.00631183	0.00861674
VIMSS206735	DVU1294	46579705	-2.25598	-3.08733
VIMSS206736	DVU1295	46579706	-0.567136	-0.726247
VIMSS206738	DVU1297	46579708	-1.44589	-1.90151
VIMSS206739	DVU1298	46579709	-0.822767	-1.37939
VIMSS206740	DVU1299	46579710	-2.65287	-3.22633
VIMSS206742	DVU1300	46579711	-1.78527	-2.80753
VIMSS206743	DVU1301	46579712	-0.210568	-0.370447
VIMSS206744	DVU1302	46579713	-0.913887	-1.42654
VIMSS206745	DVU1303	46579714	-2.33212	-3.43923

VIMSS206746	DVU1304	46579715	-1.92682	-2.88773
VIMSS206747	DVU1305	46579716	-2.76209	-4.09074
VIMSS206748	DVU1306	46579717	-1.84138	-2.68178
VIMSS206749	DVU1307	46579718	-1.80417	-2.65214
VIMSS206750	DVU1308	46579719	-1.4382	-2.00705
VIMSS206751	DVU1309	46579720	-1.67331	-2.43594
VIMSS206752	DVU1310	46579721	-1.93094	-2.5771
VIMSS206753	DVU1311	46579722	-2.26704	-3.36529
VIMSS206754	DVU1312	46579723	-1.5004	-2.29678
VIMSS206755	DVU1313	46579724	-2.01624	-3.11961
VIMSS206756	DVU1314	46579725	-0.803753	-1.05774
VIMSS206757	DVU1315	46579726	-2.16293	-3.45808
VIMSS206758	DVU1316	46579727	-1.52825	-2.34169
VIMSS206759	DVU1317	46579728	-2.04552	-3.06232
VIMSS206760	DVU1318	46579729	-1.77124	-2.68555
VIMSS206761	DVU1319	46579730	-2.19746	-3.6571
VIMSS206762	DVU1320	46579731	-0.938172	-1.60768
VIMSS206763	DVU1321	46579732	-1.32422	-1.30442
VIMSS206764	DVU1322	46579733	-2.2589	-4.11023
VIMSS206765	DVU1323	46579734	-1.51176	-1.929
VIMSS206766	DVU1324	46579735	-0.52882	-0.945901
VIMSS206767	DVU1325	46579736	-1.26819	-1.58649
VIMSS206768	DVU1326	46579737	-1.05141	-1.57727
VIMSS206769	DVU1327	46579738	-1.98906	-2.15192
VIMSS206770	DVU1328	46579739	-2.04881	-2.99946
VIMSS206771	DVU1329	46579740	-1.80031	-2.47914
VIMSS206772	DVU1330	46579741	-1.95894	-3.25831
VIMSS206773	DVU1331	46579742	0.705076	0.912398
VIMSS206774	DVU1332	46579743	-0.315026	-0.515581
VIMSS206775	DVU1333	46579744	0.835387	1.25315
VIMSS206776	DVU1334	46579745	-2.18019	-4.04302
VIMSS206777	DVU1335	46579746	-0.348293	-0.505677
VIMSS206778	DVU1336	46579747	-1.14111	-1.86458
VIMSS206779	DVU1337	46579748	-0.117353	-0.166835
VIMSS206780	DVU1338	46579749	0.693064	0.880582
VIMSS206781	DVU1339	46579750	1.58008	2.43293
VIMSS206782	DVU1340	46579751	0.67	1.08083
VIMSS206783	DVU1341	46579752	0.149367	0.221264
VIMSS206784	DVU1342	46579753	0.87376	1.31836
VIMSS206785	DVU1343	46579754	-0.0405512	-0.0623581
VIMSS206786			-0.0343508	-0.0590911
VIMSS206787	DVU1345	46579756	-1.35545	-1.67992
VIMSS206788	DVU1346	46579757	-1.02813	-1.54272
VIMSS206789	DVU1347	46579758	-0.794365	-0.96874
VIMSS206790	DVU1348	46579759	-1.18759	-1.54854
VIMSS206791	DVU1349	46579760	-0.61196	-0.945246
VIMSS206792	DVU1350	46579761	-0.512909	-0.82187
VIMSS206793	DVU1351	46579762	-0.925676	-1.1797
VIMSS206794	DVU1352	46579763	-0.553657	-1.05987

VIMSS206795	DVU1353	46579764	-0.617623	-0.787387
VIMSS206797	DVU1355	46579766	0.188212	0.313318
VIMSS206798	DVU1356	46579767	0.0808425	0.118737
VIMSS206799	DVU1357	46579768	-0.442654	-0.595278
VIMSS206800			1.54801	2.91766
VIMSS206801			0.128969	0.160691
VIMSS206802	DVU1360	46579771	0.598447	1.02171
VIMSS206803	DVU1361	46579772	-1.03792	-1.17802
VIMSS206804	DVU1362	46579773	-0.183682	-0.289557
VIMSS206805	DVU1363	46579774	0.329771	0.431401
VIMSS206806	DVU1364	46579775	0.646218	1.22724
VIMSS206807	DVU1365	46579776	0.301546	0.410345
VIMSS206808	DVU1366	46579777	-0.142179	-0.219618
VIMSS206809	DVU1368	46579779	-0.453595	-0.665377
VIMSS206810	DVU1367	46579778	3.42097	3.42792
VIMSS206811	DVU1369	46579780	-0.139625	-0.202191
VIMSS206812	DVU1370	46579781	0.0457581	0.0710821
VIMSS206813	DVU1371	46579782	-0.865047	-1.20887
VIMSS206814	DVU1372	46579783	0.468848	0.804171
VIMSS206815	DVU1373	46579784	-0.654064	-0.844652
VIMSS206816	DVU1374	46579785	0.93086	1.56848
VIMSS206817	DVU1375	46579786	0.727941	0.988663
VIMSS206818	DVU1376	46579787	1.34561	2.35544
VIMSS206819	DVU1377	46579788	-0.248443	-0.329617
VIMSS206820	DVU1378	46579789	-1.33764	-2.02442
VIMSS206822	DVU1380	46579791	-0.169595	-0.282916
VIMSS206823	DVU1381	46579792	-1.55257	-2.01241
VIMSS206827	DVU1384	46579795	1.05539	1.45965
VIMSS206829	DVU1386	46579797	-0.727657	-0.762395
VIMSS206830	DVU1387	46579798	-0.671595	-1.16416
VIMSS206831	DVU1388	46579799	2.2806	2.90448
VIMSS206832	DVU1389	46579800	0.0626956	0.0998252
VIMSS206833	DVU1390	46579801	-0.515145	-0.696192
VIMSS206835			0.299291	0.385793
VIMSS206836			-1.13063	-1.2793
VIMSS206837	DVU1394	46579805	-2.4453	-2.94252
VIMSS206838	DVU1395	46579806	-1.85249	-2.35224
VIMSS206840	DVU1397	46579808	2.04929	3.18679
VIMSS206843	DVU1400	46579811	-0.332723	-0.434378
VIMSS206844	DVU1401	46579812	0.16864	0.257135
VIMSS206845	DVU1402	46579813	0.343027	0.479905
VIMSS206846	DVU1403	46579814	0.639087	0.882603
VIMSS206847	DVU1404	46579815	0.382461	0.505614
VIMSS206848	DVU1405	46579816	-0.14197	-0.258237
VIMSS206849	DVU1406	46579817	-0.934791	-1.41806
VIMSS206850	DVU1408	46579819	-<br>0.00595591	-<br>0.00628639
VIMSS206851	DVU1409	46579820	-0.0257172	-0.0314104
VIMSS206852	DVU1407	46579818	0.480165	0.735145

VIMSS206853	DVU1410	46579821	0.654141	0.83228
VIMSS206854	DVU1411	46579822	-2.6399	-3.82119
VIMSS206855	DVU1412	46579823	0.315298	0.41836
VIMSS206856	DVU1413	46579824	-0.969177	-1.40918
VIMSS206857	DVU1414	46579825	-0.157946	-0.204744
VIMSS206859	DVU1416	46579827	-0.0330132	-0.0295656
VIMSS206861	DVU1418	46579829	-0.16855	-0.272802
VIMSS206862	DVU1419	46579830	0.236624	0.373425
VIMSS206863	DVU1420	46579831	0.672465	0.92968
VIMSS206865	DVU1421	46579832	0.242044	0.42008
VIMSS206866	DVU1422	46579833	0.844212	1.26489
VIMSS206867	DVU1423	46579834	1.23552	2.12752
VIMSS206868	DVU1424	46579835	-0.62793	-0.954382
VIMSS206869	DVU1425	46579836	0.408285	0.735769
VIMSS206870	DVU1426	46579837	0.415394	0.535547
VIMSS206871	DVU1427	46579838	-0.557298	-1.00097
VIMSS206872	DVU1428	46579839	0.908959	1.5294
VIMSS206873	DVU1429	46579840	-0.130374	-0.123205
VIMSS206874	DVU1430	46579841	1.12218	1.94462
VIMSS206875	DVU1431	46579842	1.28666	2.19411
VIMSS206876	DVU1432	46579843	-0.169955	-0.301051
VIMSS206878	DVU1434	46579845	-0.00753034	-0.0126262
VIMSS206879	DVU1435	46579846	-0.0842199	-0.0967417
VIMSS206880	DVU1436	46579847	1.26041	2.20814
VIMSS206882	DVU1438	46579849	1.12961	1.98357
VIMSS206884	DVU1440	46579851	0.317084	0.483434
VIMSS206885	DVU1441	46579852	0.880385	1.33021
VIMSS206886	DVU1442	46579853	1.53263	2.30628
VIMSS206887	DVU1443	46579854	-0.330329	-0.47439
VIMSS206888	DVU1444	46579855	-0.905834	-1.05192
VIMSS206889	DVU1445	46579856	-0.731606	-1.05837
VIMSS206890	DVU1446	46579857	0.169219	0.184879
VIMSS206891	DVU1447	46579858	0.534175	0.854422
VIMSS206892	DVU1448	46579859	0.816811	1.28777
VIMSS206893	DVU1449	46579860	1.55967	2.82413
VIMSS206894	DVU1450	46579861	0.568515	0.94313
VIMSS206895	DVU1451	46579862	0.292478	0.47511
VIMSS206896	DVU1452	46579863	-0.0958067	-0.142486
VIMSS206897	DVU1453	46579864	-0.689406	-1.06322
VIMSS206898	DVU1454	46579865	-2.04018	-3.25646
VIMSS206899	DVU1455	46579866	-1.37143	-1.98404
VIMSS206901	DVU1457	46579868	1.09882	1.80925
VIMSS206902	DVU1458	304569698	0.857218	1.30326
VIMSS206904	DVU1459	46579870	-0.190865	-0.220067
VIMSS206905	DVU1460	46579871	0.720414	1.24812
VIMSS206906	DVU1461	46579872	0.0629285	0.100964
VIMSS206907	DVU1462	46579873	1.33433	1.81168
VIMSS206908	DVU1463	46579874	0.734408	1.15681

VIMSS206909	DVU1464	46579875	1.0405	1.38678
VIMSS206910	DVU1465	304569699	0.176436	0.209233
VIMSS206911	DVU1466	46579877	-0.148325	-0.263439
VIMSS206912	DVU1467	46579878	-0.442406	-0.664225
VIMSS206913	DVU1468	46579879	-0.0387633	-0.0620053
VIMSS206914	DVU1469	46579880	-0.372824	-0.513498
VIMSS206915	DVU1470	46579881	0.741992	1.34576
VIMSS206916	DVU1471	304569700	1.79628	2.89044
VIMSS206917	DVU1472		1.21386	2.23612
VIMSS206920	DVU1474	46579885	1.61151	2.39542
VIMSS206921	DVU1475	46579886	0.981328	1.62798
VIMSS206922	DVU1476	46579887	0.124752	0.116099
VIMSS206927	DVU1479	46579890	-0.0375413	-0.0585754
VIMSS206928	DVU1480	46579891	0.976613	1.24277
VIMSS206931	DVU1483	46579894	-0.482123	-0.657441
VIMSS206932	DVU1484	46579895	0.574456	0.894022
VIMSS206935	DVU1488	46579899	0.808023	0.879942
VIMSS206936	DVU1489	46579900	1.52991	1.89502
VIMSS206938	DVU1491	46579902	0.843694	1.33803
VIMSS206941	DVU1494	46579905	0.609446	0.715416
VIMSS206944	DVU1499	46579910	0.326734	0.31157
VIMSS206945	DVU1500	46579911	1.21402	1.57407
VIMSS206947	DVU1501	46579912	1.20955	1.6447
VIMSS206948	DVU1502	46579913	1.2388	1.43081
VIMSS206949	DVU1503	46579914	1.39289	1.5055
VIMSS206950	DVU1504	46579915	0.0615128	0.0638519
VIMSS206951	DVU1505	46579916	1.56958	1.89304
VIMSS206952	DVU1506	46579917	1.42274	1.70543
VIMSS206953	DVU1507	46579918	0.320819	0.397349
VIMSS206954	DVU1508	46579919	1.32634	1.54472
VIMSS206956	DVU1509	46579920	-1.28888	-1.98095
VIMSS206960	DVU1513	46579924	0.101442	0.0936838
VIMSS206961	DVU1514	46579925	1.03339	1.22363
VIMSS206962	DVU1515	46579926	1.13033	1.08835
VIMSS206963	DVU1516	46579927	0.267656	0.371236
VIMSS206964	DVU1517	46579928	1.62327	1.97949
VIMSS206965	DVU1518	46579929	0.230748	0.305475
VIMSS206969	DVU1520	46579931	-1.28842	-1.85258
VIMSS206970	DVU1521	46579932	0.207536	0.332907
VIMSS206971	DVU1522	46579933	-0.109448	-0.102852
VIMSS206972	DVU1523	46579934	1.1906	1.69134
VIMSS206973	DVU1524	46579935	1.48671	1.86508
VIMSS206974	DVU1525	46579936	0.0358001	0.0292303
VIMSS206975	DVU1527	46579938	0.760956	0.922742
VIMSS206976	DVU1528	46579939	1.42083	2.07607
VIMSS206977	DVU1529	46579940	0.945999	1.62269
VIMSS206978	DVU1530	46579941	-1.40269	-2.03866
VIMSS206979	DVU1531	46579942	-0.189392	-0.301106
VIMSS206980	DVU1532	46579943	0.529126	0.83727

VIMSS206981	DVU1533	46579944	-0.0422539	-0.0558755
VIMSS206982	DVU1534	46579945	2.22239	2.7023
VIMSS206983	DVU1535	46579946	1.88834	3.02961
VIMSS206984	DVU1536	46579947	0.995775	1.19427
VIMSS206985	DVU1537	46579948	-0.388177	-0.684524
VIMSS206986	DVU1538	46579949	-1.3231	-1.71468
VIMSS206988	DVU1540	46579951	-1.37563	-1.82532
VIMSS206989	DVU1541	46579952	1.44772	2.45162
VIMSS206990	DVU1542	46579953	-0.636264	-0.884936
VIMSS206991	DVU1543	46579954	0.699913	1.15366
VIMSS206992			1.11057	1.5245
VIMSS206993	DVU1545	46579956	1.4632	1.97826
VIMSS206995	DVU1547	46579958	0.527014	0.870465
VIMSS206996	DVU1548	46579959	-1.82802	-2.42666
VIMSS206997	DVU1549	46579960	1.41492	2.3672
VIMSS206998	DVU1550	46579961	0.25683	0.298427
VIMSS206999	DVU1551	46579962	-0.134354	-0.222335
VIMSS207000	DVU1552	46579963	0.809115	1.0555
VIMSS207001	DVU1553	46579964	0.469923	0.624121
VIMSS207002	DVU1554	46579965	0.948537	1.14458
VIMSS207003	DVU1555	46579966	1.90598	3.00379
VIMSS207004	DVU1556	46579967	1.86481	2.27131
VIMSS207006	DVU1558	46579969	1.84163	2.1118
VIMSS207007	DVU1559	46579970	1.22899	1.92493
VIMSS207008	DVU1560	46579971	0.665102	0.73649
VIMSS207010	DVU1561	46579972	0.968538	1.26237
VIMSS207011	DVU1562	46579973	0.479714	0.576002
VIMSS207012	DVU1563	46579974	1.3784	1.59595
VIMSS207016			-0.653593	-0.93878
VIMSS207018	DVU1568	46579979	-0.234336	-0.319296
VIMSS207019	DVU1569	46579980	0.178791	0.245697
VIMSS207020	DVU1570	46579981	0.765152	1.07166
VIMSS207021			-1.44938	-1.87289
VIMSS207022	DVU1572	46579983	1.39667	2.58474
VIMSS207023	DVU1573	46579984	-1.46272	-1.80649
VIMSS207024	DVU1574	46579985	-2.12504	-2.79848
VIMSS207025	DVU1575	46579986	-2.48725	-3.20599
VIMSS207026	DVU1576	46579987	-0.151293	-0.226659
VIMSS207028	DVU1577	46579988	0.776374	1.29726
VIMSS207029	DVU1578	46579989	0.119189	0.164631
VIMSS207030	DVU1579	46579990	-0.0146713	-0.0257481
VIMSS207031	DVU1580	46579991	-0.308505	-0.376803
VIMSS207032	DVU1581	46579992	0.155725	0.250368
VIMSS207033	DVU1582	46579993	-0.6079	-0.792921
VIMSS207034	DVU1583	46579994	-0.392868	-0.557049
VIMSS207035	DVU1584	46579995	-0.989724	-1.22433
VIMSS207036	DVU1585	46579996	-0.0213061	-0.0274001
VIMSS207037	DVU1586	46579997	-0.160518	-0.18521
VIMSS207038	DVU1587	46579998	-0.972663	-1.66746

VIMSS207040	DVU1589	46580000	-0.0419745	-0.0695262
VIMSS207041	DVU1590	46580001	0.382756	0.491921
VIMSS207042	DVU1591	46580002	1.35772	1.64541
VIMSS207043	DVU1592	46580003	0.966718	1.55324
VIMSS207044	DVU1593	46580004	1.23322	2.23442
VIMSS207045	DVU1594	46580005	1.45177	2.28097
VIMSS207046	DVU1595	304569703	0.844359	1.17045
VIMSS207047	DVU1596	46580007	1.19156	1.85619
VIMSS207048	DVU1597	46580008	1.17888	1.58811
VIMSS207050	DVU1599	46580010	0.332142	0.439278
VIMSS207051	DVU1600	46580011	0.723733	1.24786
VIMSS207052	DVU1601	46580012	1.20912	1.80144
VIMSS207054	DVU1603	46580014	1.64764	2.48379
VIMSS207055			-1.49064	-1.74165
VIMSS207056	DVU1605	46580016	0.59099	0.811976
VIMSS207057	DVU1606	46580017	0.114228	0.143275
VIMSS207058	DVU1607	46580018	0.518104	0.930912
VIMSS207059	DVU1608	46580019	-1.10975	-1.18037
VIMSS207060	DVU1609	46580020	-0.473112	-0.780743
VIMSS207061	DVU1610	46580021	-0.415333	-0.36555
VIMSS207062	DVU1611	46580022	0.465544	0.774751
VIMSS207063	DVU1612	46580023	-0.470508	-0.603358
VIMSS207064	DVU1613	304569704	-0.350254	-0.591284
VIMSS207065	DVU1614	46580025	-0.311925	-0.397103
VIMSS207067	DVU1615	46580026	-1.1727	-1.47381
VIMSS207068	DVU1617	46580028	-0.390428	-0.675687
VIMSS207069	DVU1618	46580029	-1.35986	-1.84457
VIMSS207070	DVU1619	46580030	-0.491874	-0.871842
VIMSS207071	DVU1620	46580031	1.11489	1.82873
VIMSS207072	DVU1621	46580032	-0.347509	-0.532359
VIMSS207073	DVU1622	46580033	-0.976319	-1.27871
VIMSS207074	DVU1623	46580034	-0.897088	-1.38447
VIMSS207075	DVU1624	46580035	-0.0348715	-0.0531344
VIMSS207076	DVU1625	46580036	0.0625843	0.101511
VIMSS207077	DVU1626	46580037	-0.926568	-1.68115
VIMSS207079	DVU1627	46580038	-0.702689	-1.12429
VIMSS207080	DVU1628	46580039	-0.0616097	-0.0996969
VIMSS207081	DVU1629	46580040	1.06578	1.86778
VIMSS207082	DVU1630	46580041	-0.609018	-1.0983
VIMSS207083	DVU1631	46580042	-0.571276	-0.910456
VIMSS207084	DVU1632	46580043	-0.741061	-1.18458
VIMSS207085	DVU1633	46580044	-0.855345	-1.28272
VIMSS207086	DVU1634	46580045	-0.106406	-0.174609
VIMSS207087	DVU1635	46580046	-1.63366	-2.27754
VIMSS207088	DVU1636	46580047	-0.943045	-1.22824
VIMSS207090	DVU1638	46580049	-0.996489	-1.81764
VIMSS207091	DVU1639	46580050	-0.243604	-0.417247
VIMSS207094	DVU1641	46580052	0.0449815	0.0775968
VIMSS207100	DVU1644	46580054	0.172655	0.24837

VIMSS207101	DVU1645	46580055	-1.09876	-1.82349
VIMSS207104	DVU1646	46580056	-0.700761	-1.00703
VIMSS207105	DVU1647	46580057	1.33943	2.53606
VIMSS207106	DVU1648	46580058	0.0462937	0.0809489
VIMSS207107	DVU1649	46580059	1.11334	1.97001
VIMSS207109	DVU1650	46580060	0.21196	0.350714
VIMSS207110	DVU1651	46580061	0.796006	1.29663
VIMSS207111	DVU1652	46580062	-0.17347	-0.292349
VIMSS207114	DVU1655	46580065	0.36533	0.661061
VIMSS207115	DVU1656	46580066	1.73402	2.71248
VIMSS207116	DVU1657	46580067	-0.592231	-1.06321
VIMSS207117	DVU1658	46580068	0.545604	0.794428
VIMSS207119	DVU1660	46580070	-0.251	-0.392683
VIMSS207120	DVU1661	46580071	-0.821231	-1.22164
VIMSS207121	DVU1662	46580072	-0.727749	-1.02237
VIMSS207122	DVU1663	46580073	-1.09195	-1.1998
VIMSS207123	DVU1664	46580074	-1.1006	-1.86154
VIMSS207124	DVU1665	46580075	-1.67509	-2.40641
VIMSS207125	DVU1666	46580076	-1.29027	-2.12458
VIMSS207126	DVU1667	46580077	0.394138	0.709744
VIMSS207127	DVU1668	46580078	0.149683	0.233539
VIMSS207128	DVU1669	304569705	-0.133449	-0.207911
VIMSS207129	DVU1670	46580080	-0.899401	-1.48609
VIMSS207130	DVU1671	46580081	-1.04477	-1.47129
VIMSS207131	DVU1672	46580082	-0.476125	-0.813698
VIMSS207132	DVU1673	304569706	-0.444195	-0.515209
VIMSS207133	DVU1674	46580084	0.17589	0.316694
VIMSS207134	DVU1675	46580085	1.44887	2.54598
VIMSS207135	DVU1676	46580086	-1.53192	-2.42548
VIMSS207136	DVU1677	46580087	0.189426	0.331532
VIMSS207137	DVU1678	46580088	-0.660866	-0.96127
VIMSS207138	DVU1679	46580089	-0.167502	-0.271916
VIMSS207139	DVU1680	46580090	0.108449	0.160889
VIMSS207140	DVU1681	46580091	0.422182	0.756807
VIMSS207141	DVU1682	46580092	-0.293567	-0.423354
VIMSS207142	DVU1683	46580093	-0.196527	-0.258225
VIMSS207143	DVU1684	304569707	0.661936	1.07938
VIMSS207144	DVU1685	46580095	0.672755	1.25406
VIMSS207145	DVU1686	46580096	0.443947	0.753175
VIMSS207146	DVU1687	46580097	0.0357945	0.0415412
VIMSS207147	DVU1688	46580098	-1.39108	-2.48684
VIMSS207149	DVU1690	46580100	-1.32049	-2.37847
VIMSS207151	DVU1692	46580102	0.520038	0.815426
VIMSS207152	DVU1693	46580103	-1.36579	-2.33614
VIMSS207153	DVU1694	46580104	0.543799	0.719139
VIMSS207154	DVU1695	46580105	1.38165	1.85584
VIMSS207155	DVU1696	46580106	1.19528	1.10262
VIMSS207156			1.28638	1.92181
VIMSS207157	DVU1698	46580108	1.06881	1.08366

VIMSS207159	DVU1699	46580109	-0.260228	-0.46452
VIMSS207161	DVU1701	46580111	-0.551604	-0.739105
VIMSS207163	DVU1703	46580113	0.0849996	0.143752
VIMSS207165	DVU1705	304569708	-1.27655	-2.05319
VIMSS207166	DVU1707	46580117	-0.149857	-0.210828
VIMSS207167	DVU1708	46580118	-0.303411	-0.431334
VIMSS207168	DVU1709	46580119	0.305938	0.471495
VIMSS207171			-0.659243	-1.23833
VIMSS207173	DVU1713	46580123	1.51332	1.59219
VIMSS207174	DVU1714	46580124	0.604514	0.82434
VIMSS207175	DVU1715	46580125	0.146601	0.159689
VIMSS207176	DVU1716	46580126	1.47008	1.75471
VIMSS207177	DVU1717	46580127	1.41669	2.33653
VIMSS207178	DVU1718	46580128	1.30507	1.51697
VIMSS207179	DVU1719	46580129	-0.217546	-0.204468
VIMSS207180	DVU1720	46580130	2.13451	2.15804
VIMSS207181	DVU1721	46580131	1.01399	1.37409
VIMSS207183	DVU1723	46580133	3.20381	3.03988
VIMSS207184	DVU1724	46580134	2.19338	2.25105
VIMSS207185	DVU1725	46580135	-0.284595	-0.356888
VIMSS207186	DVU1727	46580137	1.93543	1.80744
VIMSS207187	DVU1726	46580136	0.799808	1.10904
VIMSS207188	DVU1728	46580138	0.254266	0.332447
VIMSS207189	DVU1729	46580139	0.740059	1.00825
VIMSS207190	DVU1730	46580140	1.1044	1.58666
VIMSS207191	DVU1731	46580141	-0.567256	-0.544372
VIMSS207197	DVU1736	46580146	0.255096	0.252243
VIMSS207198	DVU1737	46580147	-0.435575	-0.673362
VIMSS207199	DVU1738	46580148	1.009	0.954613
VIMSS207201	DVU1740	46580150	2.4604	2.97038
VIMSS207202	DVU1741	46580151	1.23417	1.60289
VIMSS207203	DVU1742	46580152	-1.51677	-1.65811
VIMSS207204	DVU1743	46580153	0.572613	0.563869
VIMSS207205	DVU1744	46580154	-1.29946	-1.30556
VIMSS207208	DVU1746	46580156	-1.04745	-1.51327
VIMSS207213	DVU1750	46580160	-0.542419	-0.627579
VIMSS207214	DVU1751	46580161	-1.55649	-1.67407
VIMSS207216	DVU1752	46580162	0.449303	0.478399
VIMSS207217	DVU1753	46580163	0.518303	0.661912
VIMSS207218	DVU1754	46580164	0.672901	0.798291
VIMSS207221	DVU1756	46580166	-1.84746	-2.45591
VIMSS207223	DVU1758	46580168	-0.407721	-0.644425
VIMSS207224	DVU1759	46580169	-0.270632	-0.317132
VIMSS207225	DVU1760	46580170	0.808187	1.00703
VIMSS207227	DVU1762	46580172	1.07528	1.53039
VIMSS207229	DVU1764	46580174	-1.35196	-2.4262
VIMSS207230	DVU1765	46580175	-1.59366	-1.96549
VIMSS207231	DVU1766	46580176	0.761945	0.723039
VIMSS207232	DVU1767	46580177	0.209469	0.267139

VIMSS207233	DVU1768	46580178	0.263155	0.328805
VIMSS207234	DVU1769	46580179	-0.896163	-1.12159
VIMSS207235	DVU1770	46580180	0.750601	0.820842
VIMSS207236	DVU1771	46580181	0.570751	0.882544
VIMSS207237	DVU1772	46580182	0.339776	0.558114
VIMSS207239	DVU1774	46580184	0.273471	0.413266
VIMSS207240	DVU1775	46580185	-0.793752	-1.12239
VIMSS207241	DVU1776	46580186	-0.763094	-0.853011
VIMSS207242	DVU1777	46580187	-2.01732	-2.23352
VIMSS207243	DVU1778	46580188	0.881261	1.31318
VIMSS207244	DVU1779	46580189	1.27314	1.73782
VIMSS207245	DVU1780	46580190	0.943873	1.50804
VIMSS207246	DVU1781	46580191	2.21201	3.46087
VIMSS207247	DVU1782	46580192	2.26418	3.39602
VIMSS207248	DVU1783	46580193	1.80743	2.55223
VIMSS207249	DVU1784	46580194	0.209332	0.381854
VIMSS207250	DVU1785	46580195	-0.370397	-0.357909
VIMSS207251	DVU1786	46580196	1.07939	1.92212
VIMSS207252	DVU1787	46580197	-0.991714	-1.59419
VIMSS207253	DVU1788	46580198	-0.630924	-1.1029
VIMSS207254	DVU1789	46580199	-1.37643	-1.66769
VIMSS207256	DVU1791	46580201	-0.936594	-1.33114
VIMSS207257	DVU1792	161343883	-1.81553	-2.92231
VIMSS207259	DVU1794	46580204	1.99208	3.56503
VIMSS207260	DVU1795	46580205	-0.122852	-0.188861
VIMSS207262			-0.198896	-0.259621
VIMSS207263	DVU1798	46580208	0.725163	1.28659
VIMSS207264	DVU1799	46580209	-0.0133271	-0.0194051
VIMSS207266	DVU1801	46580211	0.239728	0.33664
VIMSS207267	DVU1802	46580212	1.28871	1.43874
VIMSS207268	DVU1803	46580213	0.474353	0.647917
VIMSS207269			1.559	1.79949
VIMSS207270	DVU1805	46580215	0.254923	0.361644
VIMSS207271	DVU1806	46580216	0.0708907	0.0943595
VIMSS207272	DVU1807	46580217	-0.623756	-0.640085
VIMSS207273	DVU1808	46580218	-0.659634	-0.983712
VIMSS207274	DVU1809	46580219	0.356134	0.51535
VIMSS207275	DVU1810	46580220	0.671754	0.887022
VIMSS207276	DVU1811	46580221	2.21365	2.87396
VIMSS207277	DVU1812	46580222	1.79114	2.64207
VIMSS207278	DVU1813	46580223	0.762393	1.0423
VIMSS207279	DVU1814	46580224	1.3106	2.17391
VIMSS207281			1.17994	1.75793
VIMSS207282	DVU1817	46580227	3.32953	4.02432
VIMSS207283	DVU1818	46580228	-0.668335	-1.15821
VIMSS207284	DVU1819	46580229	-1.11777	-1.88076
VIMSS207285	DVU1820	46580230	0.656457	0.885663
VIMSS207286	DVU1821	46580231	0.507004	0.568444
VIMSS207287	DVU1822	46580232	0.0697221	0.122352

VIMSS207288	DVU1823	46580233	0.21994	0.324228
VIMSS207289	DVU1824	46580234	2.12636	2.55586
VIMSS207290	DVU1825	46580235	0.176127	0.327005
VIMSS207291	DVU1826	46580236	-2.31009	-3.3142
VIMSS207292	DVU1827	46580237	-0.772429	-1.42905
VIMSS207293	DVU1828	46580238	-2.01788	-1.95023
VIMSS207295	DVU1830	46580240	0.78067	1.25836
VIMSS207297	DVU1832	46580241	-0.780159	-1.2485
VIMSS207298	DVU1833	46580242	-0.936568	-1.67253
VIMSS207299	DVU1834	304569711	0.019593	0.0301832
VIMSS207300	DVU1835		-0.282858	-0.457782
VIMSS207302	DVU1836	46580245	0.0199262	0.026064
VIMSS207303	DVU1837	46580246	-0.920914	-1.29773
VIMSS207304	DVU1838	46580247	0.379251	0.703342
VIMSS207305	DVU1839	46580248	1.17029	1.91437
VIMSS207306	DVU1840	46580249	-1.06474	-1.81063
VIMSS207307	DVU1841	46580250	-1.01256	-1.47353
VIMSS207308	DVU1842	46580251	0.163284	0.292282
VIMSS207309	DVU1843	46580252	0.0950536	0.149543
VIMSS207310	DVU1844	46580253	0.930683	1.47096
VIMSS207311	DVU1845	46580254	0.483595	0.708709
VIMSS207312	DVU1846	46580255	0.827226	1.26786
VIMSS207313	DVU1847	46580256	-0.271137	-0.434184
VIMSS207314	DVU1848	46580257	-0.982842	-1.54469
VIMSS207315	DVU1849	46580258	-0.642162	-0.944575
VIMSS207316	DVU1850	46580259	0.636433	1.05223
VIMSS207317	DVU1851	46580260	0.0555183	0.0938515
VIMSS207318	DVU1853	46580262	0.819621	1.17772
VIMSS207319	DVU1854	46580263	0.28066	0.370678
VIMSS207321	DVU1855	46580264	0.936879	1.25096
VIMSS207323	DVU1857	46580266	0.748553	1.08428
VIMSS207324	DVU1858	46580267	5.11825	7.32522
VIMSS207325	DVU1859	46580268	-0.494012	-0.578108
VIMSS207326	DVU1860	46580269	-0.595127	-1.04387
VIMSS207327	DVU1861	46580270	0.339023	0.611392
VIMSS207328	DVU1862	46580271	0.871763	1.57049
VIMSS207329	DVU1863	46580272	-0.0441485	-0.0674063
VIMSS207331	DVU1865	46580274	-0.935626	-1.36851
VIMSS207332	DVU1866	46580275	-0.112476	-0.197094
VIMSS207333	DVU1867	46580276	0.906183	1.55948
VIMSS207334	DVU1868	46580277	0.0671592	0.122988
VIMSS207335	DVU1869	46580278	0.703561	1.14118
VIMSS207336	DVU1870	46580279	0.711145	1.08303
VIMSS207339			-1.21564	-1.63563
VIMSS207340	DVU1874	46580283	0.804003	1.35829
VIMSS207341	DVU1875	46580284	0.753075	1.33021
VIMSS207342	DVU1876	46580285	0.315074	0.542162
VIMSS207343	DVU1877	46580286	0.240893	0.388049
VIMSS207347	DVU1881	46580290	-0.3988	-0.587859

VIMSS207348	DVU1882	46580291	0.0582634	0.10079
VIMSS207349	DVU1883	46580292	0.106858	0.169307
VIMSS207350	DVU1884	46580293	0.344824	0.483697
VIMSS207352	DVU1886	46580295	-0.366077	-0.650836
VIMSS207353	DVU1887	46580296	-0.605855	-1.05032
VIMSS207354	DVU1888	46580297	0.52216	0.780476
VIMSS207355	DVU1889	46580298	-0.963158	-1.37991
VIMSS207357	DVU1890	46580299	-0.834404	-1.33993
VIMSS207358	DVU1891	46580300	0.678987	1.2889
VIMSS207359	DVU1892	46580301	1.28655	2.26923
VIMSS207360	DVU1893	46580302	-1.06813	-1.50832
VIMSS207361			0.474891	0.714159
VIMSS207363	DVU1895	46580304	-0.101208	-0.146305
VIMSS207364	DVU1896	46580305	-2.80788	-3.59966
VIMSS207366	DVU1898	46580307	-0.694292	-1.23026
VIMSS207367	DVU1899	46580308	1.5977	2.0364
VIMSS207368	DVU1900	46580309	0.172821	0.305488
VIMSS207369			0.647166	1.14224
VIMSS207370	DVU1902	46580311	-0.474623	-0.609916
VIMSS207371	DVU1903	46580312	-1.21374	-1.5019
VIMSS207372	DVU1904	46580313	0.179534	0.249924
VIMSS207378	DVU1909	46580318	0.851394	1.33958
VIMSS207379	DVU1910	46580319	1.15302	1.67177
VIMSS207380	DVU1911	46580320	-0.474618	-0.729898
VIMSS207382	DVU1913	46580322	-0.892615	-1.3393
VIMSS207384	DVU1915	46580324	-0.652898	-0.974582
VIMSS207385	DVU1916	46580325	-1.25138	-1.44054
VIMSS207386	DVU1917	46580326	1.26175	1.94586
VIMSS207388	DVU1919	46580328	-0.288726	-0.440452
VIMSS207390	DVU1921	46580330	1.68218	1.937
VIMSS207391	DVU1922	46580331	1.2393	1.43251
VIMSS207392	DVU1923	46580332	0.638412	1.17738
VIMSS207393	DVU1924	46580333	1.39762	1.97312
VIMSS207394	DVU1925	46580334	1.43977	2.30482
VIMSS207395	DVU1926	46580335	0.507833	0.65828
VIMSS207397	DVU1927	46580336	-1.65562	-2.89439
VIMSS207398	DVU1928	46580337	-0.448483	-0.706611
VIMSS207399	DVU1929	46580338	-1.63553	-2.40273
VIMSS207400	DVU1930	46580339	-0.574673	-0.809647
VIMSS207401	DVU1931	46580340	-1.31887	-1.80405
VIMSS207402	DVU1932	46580341	0.519214	0.656212
VIMSS207403	DVU1933	46580342	-0.964738	-1.54653
VIMSS207404	DVU1934	46580343	-0.505714	-0.807565
VIMSS207405	DVU1935	46580344	-1.23274	-1.70186
VIMSS207406	DVU1936	46580345	-0.594847	-1.02404
VIMSS207407	DVU1937	46580346	0.476976	0.749603
VIMSS207408	DVU1938	46580347	0.139538	0.226046
VIMSS207409	DVU1939	46580348	-0.201719	-0.311745
VIMSS207410	DVU1940	46580349	0.383389	0.605651

VIMSS207411	DVU1941	46580350	-0.30347	-0.343829
VIMSS207412	DVU1942	46580351	-0.975717	-1.73801
VIMSS207413	DVU1943	46580352	0.737326	1.19529
VIMSS207414	DVU1944	46580353	2.1626	4.10894
VIMSS207415	DVU1945	46580354	1.95683	3.01577
VIMSS207416	DVU1946	46580355	1.58358	2.82504
VIMSS207417	DVU1947	46580356	0.199236	0.29872
VIMSS207418	DVU1948	46580357	-1.66796	-2.57499
VIMSS207419	DVU1949	46580358	-1.15429	-1.45892
VIMSS207420	DVU1950	46580359	-0.447256	-0.703816
VIMSS207421	DVU1951	46580360	-1.07857	-1.41513
VIMSS207422	DVU1952	46580361	-0.0910193	-0.144528
VIMSS207423	DVU1953	46580362	-0.422113	-0.590628
VIMSS207424	DVU1954	46580363	-0.0353983	-0.0545413
VIMSS207425	DVU1955	46580364	-0.594008	-0.813555
VIMSS207426	DVU1956	46580365	0.296455	0.468687
VIMSS207428	DVU1958	46580367	1.11412	1.48048
VIMSS207430	DVU1960	46580369	-0.595247	-0.794601
VIMSS207431	DVU1961	46580370	0.0528653	0.0687789
VIMSS207437	DVU1967	46580376	-0.369691	-0.519931
VIMSS207438	DVU1968	46580377	0.428574	0.426298
VIMSS207439	DVU1969	46580378	-0.468922	-0.567417
VIMSS207440	DVU1970	46580379	0.0574892	0.0682524
VIMSS207441	DVU1971	46580380	-1.22004	-1.79784
VIMSS207444	DVU1973	46580382	0.173562	0.220008
VIMSS207446			-1.64125	-2.43469
VIMSS207447	DVU1976	46580385	-1.47767	-1.93779
VIMSS207448	DVU1977	46580386	-1.38173	-2.12805
VIMSS207449	DVU1978	46580387	-0.634992	-0.985418
VIMSS207451	DVU1980	46580389	1.49642	2.71628
VIMSS207452	DVU1981	46580390	-1.50815	-1.96564
VIMSS207454	DVU1983	46580392	0.0986355	0.172121
VIMSS207455	DVU1984	46580393	0.420027	0.706057
VIMSS207456	DVU1985	46580394	-1.25496	-1.28108
VIMSS207458	DVU1986	46580395	2.18552	3.32854
VIMSS207459	DVU1987	46580396	-0.772086	-0.949028
VIMSS207460	DVU1988	46580397	-0.0746523	-0.0987065
VIMSS207463	DVU1991	46580400	0.115225	0.157248
VIMSS207464	DVU1992	46580401	0.450143	0.792395
VIMSS207465	DVU1993	46580402	1.76803	2.44746
VIMSS207467	DVU1995	46580404	-1.07441	-1.54032
VIMSS207471	DVU1999	46580408	-0.110138	-0.165551
VIMSS207472	DVU2000	46580409	-1.77656	-2.71817
VIMSS207476	DVU2003	46580411	1.00352	0.774877
VIMSS207479	DVU2006	46580414	-1.00496	-1.52625
VIMSS207480	DVU2007	46580415	-0.611319	-0.794753
VIMSS207482	DVU2009	46580417	-2.72179	-4.02032
VIMSS207483	DVU2010	46580418	-1.6209	-2.68854
VIMSS207485	DVU2012	46580420	-2.35846	-3.01315

VIMSS207486	DVU2013	46580421	1.14941	1.48998
VIMSS207487	DVU2014	46580422	1.14755	1.49684
VIMSS207489	DVU2016	46580424	-3.64955	-6.47517
VIMSS207490	DVU2017	46580425	-0.375249	-0.623221
VIMSS207492	DVU2019	46580426	-1.89697	-2.38212
VIMSS207493	DVU2020	46580427	-0.840618	-1.24637
VIMSS207494	DVU2021	46580428	-2.41108	-3.37485
VIMSS207496	DVU2023	46580430	-1.30472	-1.52169
VIMSS207497	DVU2024	46580431	-1.22632	-1.50935
VIMSS207498	DVU2025	46580432	-2.97491	-3.91445
VIMSS207499	DVU2026	46580433	-1.89964	-3.10009
VIMSS207501	DVU2028	46580435	-2.61832	-3.91603
VIMSS207505	DVU2032	46580439	-5.16044	-7.30502
VIMSS207506	DVU2033	46580440	-6.82381	-10.7965
VIMSS207507	DVU2034	46580441	-3.04477	-3.75729
VIMSS207508	DVU2035	46580442	-0.324489	-0.476405
VIMSS207509	DVU2036	46580443	3.82343	4.28634
VIMSS207510	DVU2037	46580444	-2.74568	-3.50063
VIMSS207511	DVU2038	46580445	-5.8065	-6.81687
VIMSS207512	DVU2039	46580446	-3.09196	-3.48461
VIMSS207513	DVU2040	46580447	-3.76425	-3.56377
VIMSS207514	DVU2041	46580448	-3.03902	-3.85106
VIMSS207515			-0.247495	-0.335854
VIMSS207516	DVU2043	46580450	-4.27489	-4.32315
VIMSS207517	DVU2044	46580451	-4.94354	-7.48319
VIMSS207518	DVU2045	46580452	-1.50411	-1.80152
VIMSS207519	DVU2046	46580453	-0.789352	-1.221
VIMSS207521	DVU2048	46580455	-1.32291	-2.10485
VIMSS207523	DVU2049		-2.4156	-3.21589
VIMSS207524	DVU2051	46580456	0.71023	0.957156
VIMSS207525	DVU2052	46580457	-3.58903	-4.94418
VIMSS207526	DVU2053	46580458	-2.95182	-4.16647
VIMSS207527	DVU2054	46580459	-2.43131	-3.15145
VIMSS207528	DVU2055	46580460	-0.140155	-0.224712
VIMSS207530	DVU2057	46580462	0.334512	0.449933
VIMSS207531			0.180566	0.232967
VIMSS207532	DVU2059	46580464	1.82018	2.67686
VIMSS207533	DVU2060	46580465	2.23138	3.13746
VIMSS207534	DVU2061	46580466	3.03958	3.87857
VIMSS207535	DVU2062	46580467	-0.439532	-0.576352
VIMSS207536	DVU2063	46580468	-0.377403	-0.479431
VIMSS207537	DVU2064	46580469	3.15863	5.27313
VIMSS207539	DVU2066	46580471	-3.64798	-5.82579
VIMSS207540	DVU2067	46580472	0.134403	0.154733
VIMSS207541	DVU2068	46580473	0.151472	0.180377
VIMSS207543	DVU2069	46580474	0.0904615	0.147735
VIMSS207544	DVU2070	46580475	1.8185	2.80695
VIMSS207545	DVU2071	46580476	0.413407	0.446115
VIMSS207547	DVU2073	46580478	0.356934	0.644756

VIMSS207548	DVU2074	304569714	4.07606	5.09069
VIMSS207549	DVU2075	46580480	1.34	1.97771
VIMSS207550	DVU2076	46580481	0.671829	0.821267
VIMSS207551	DVU2077	46580482	2.08243	2.69877
VIMSS207552	DVU2078	46580483	-2.9135	-3.47318
VIMSS207553			-4.85343	-7.61706
VIMSS207555	DVU2081	46580486	-1.00967	-1.00871
VIMSS207556	DVU2082	46580487	3.09117	3.90296
VIMSS207558	DVU2084	46580489	1.24821	2.23048
VIMSS207559	DVU2085	304569715	0.821756	1.34163
VIMSS207560	DVU2086	46580491	-2.63799	-3.72526
VIMSS207561	DVU2087	46580492	-6.36485	-6.40006
VIMSS207562	DVU2088	46580493	-2.47648	-3.2523
VIMSS207565	DVU2090	46580495	1.92524	2.02529
VIMSS207566	DVU2091	46580496	-3.11458	-5.01229
VIMSS207567	DVU2092	46580497	0.156864	0.194292
VIMSS207568	DVU2093	46580498	-0.981972	-1.38957
VIMSS207570	DVU2095	46580500	4.26179	4.56254
VIMSS207571	DVU2096	46580501	-3.61334	-4.76882
VIMSS207572	DVU2097	46580502	-2.2646	-3.68866
VIMSS207573	DVU2098	46580503	-3.88452	-5.47876
VIMSS207574	DVU2099	46580504	0.469003	0.501573
VIMSS207575	DVU2100	46580505	6.03432	7.80549
VIMSS207577	DVU2101	46580506	0.231477	0.364327
VIMSS207578	DVU2102	46580507	2.17174	2.88716
VIMSS207580	DVU2104	46580509	5.98646	8.79858
VIMSS207581	DVU2105	46580510	3.74077	4.47071
VIMSS207582	DVU2106	46580511	-3.39693	-3.83443
VIMSS207583			-1.88034	-1.89169
VIMSS207584	DVU2108	46580513	1.64453	2.78614
VIMSS207585	DVU2109	46580514	0.415797	0.642659
VIMSS207586	DVU2110	46580515	0.238955	0.281213
VIMSS207587	DVU2111	46580516	-0.0069468	-0.00932018
VIMSS207588	DVU2112	46580517	1.12735	2.13266
VIMSS207589	DVU2113	46580518	-1.40199	-1.54886
VIMSS207590	DVU2114	46580519	-1.51102	-2.17749
VIMSS207591	DVU2115	46580520	1.50525	2.14676
VIMSS207594	DVU2117	46580522	0.211697	0.169658
VIMSS207595	DVU2118	46580523	1.44995	1.59466
VIMSS207596	DVU2119	46580524	0.646331	0.678385
VIMSS207597	DVU2120	46580525	0.634078	0.742371
VIMSS207598	DVU2121	46580526	0.00825943	0.00882666
VIMSS207600	DVU2123	46580528	1.50052	1.85123
VIMSS207601	DVU2124	46580529	1.81969	2.55298
VIMSS207602	DVU2125	46580530	0.910713	0.974345
VIMSS207603	DVU2126	46580531	1.08877	1.03703
VIMSS207604	DVU2127	46580532	1.45913	1.33183
VIMSS207605	DVU2128	46580533	0.324996	0.508405

VIMSS207607	DVU2130	46580535	1.36176	1.78248
VIMSS207608	DVU2131	46580536	-0.225373	-0.274097
VIMSS207609	DVU2132	46580537	0.961773	1.19468
VIMSS207610	DVU2133	46580538	0.323468	0.44462
VIMSS207612	DVU2135	46580540	-0.851717	-0.889599
VIMSS207613	DVU2136	46580541	1.06775	1.57497
VIMSS207614	DVU2137	46580542	-0.337474	-0.326262
VIMSS207615	DVU2138	46580543	0.682382	1.07171
VIMSS207616	DVU2139	46580544	-0.0659247	-0.10952
VIMSS207617	DVU2140	46580545	0.901686	1.56485
VIMSS207618	DVU2141	46580546	-0.122629	-0.209984
VIMSS207619	DVU2142	46580547	-0.372504	-0.605664
VIMSS207621	DVU2144	46580549	-0.364759	-0.474582
VIMSS207623	DVU2145	46580550	-0.64009	-0.734702
VIMSS207625	DVU2146	46580551	-0.159682	-0.179349
VIMSS207626	DVU2147	46580552	0.509746	0.609734
VIMSS207627	DVU2148	46580553	0.34859	0.514156
VIMSS207628	DVU2149	46580554	-0.806348	-1.00805
VIMSS207629	DVU2150	46580555	-1.91042	-2.62905
VIMSS207630	DVU2151	46580556	-0.645991	-0.700703
VIMSS207631	DVU2152	46580557	0.392043	0.563145
VIMSS207633	DVU2154	46580559	0.903759	0.963608
VIMSS207634	DVU2155	46580560	2.21606	2.83135
VIMSS207635	DVU2156	46580561	1.08641	1.20684
VIMSS207636	DVU2157	46580562	1.42794	2.14153
VIMSS207640	DVU2160	46580565	1.44183	2.01275
VIMSS207644	DVU2164	46580569	1.77953	2.85882
VIMSS207645	DVU2165	46580570	1.64547	1.71074
VIMSS207646	DVU2166	46580571	0.830068	1.04174
VIMSS207648	DVU2168	46580573	-0.271013	-0.289113
VIMSS207651	DVU2171	46580576	1.35627	1.1855
VIMSS207652	DVU2172	46580577	1.16586	1.02642
VIMSS207653	DVU2173	46580578	-0.0198471	-0.0180879
VIMSS207656	DVU2175	46580580	-2.40331	-3.06474
VIMSS207658	DVU2176	46580581	-0.472131	-0.586017
VIMSS207659	DVU2177	46580582	-0.840506	-1.1784
VIMSS207663	DVU2180	46580585	0.689058	0.851743
VIMSS207664	DVU2181	46580586	0.53582	0.761959
VIMSS207668	DVU2184	46580589	0.129669	0.140368
VIMSS207669	DVU2185	46580590	-0.482573	-0.509895
VIMSS207671	DVU2187	46580592	0.388063	0.366071
VIMSS207672	DVU2188	46580593	0.173722	0.280384
VIMSS207673	DVU2189	46580594	0.00149008	0.00237036
VIMSS207674	DVU2190	46580595	0.833864	1.13595
VIMSS207675	DVU2191	46580596	1.25625	1.70765
VIMSS207676	DVU2192	46580597	1.21684	1.48561
VIMSS207677	DVU2193	46580598	0.28062	0.392835
VIMSS207678	DVU2194	46580599	2.46329	2.66912
VIMSS207679	DVU2195	46580600	1.7929	2.85332

VIMSS207680	DVU2196	46580601	0.423774	0.489285
VIMSS207681	DVU2197	46580602	0.558224	0.732709
VIMSS207682	DVU2198	46580603	-0.809659	-1.09929
VIMSS207684	DVU2200	46580605	-0.897173	-1.43119
VIMSS207686	DVU2202	46580607	1.42598	1.8561
VIMSS207687	DVU2203	46580608	-1.15201	-1.52714
VIMSS207688	DVU2204	46580609	0.573929	0.754289
VIMSS207689	DVU2205	46580610	-2.26879	-2.00896
VIMSS207690	DVU2206	46580611	-0.505416	-0.659022
VIMSS207692	DVU2208	46580613	-0.622543	-0.873809
VIMSS207693	DVU2209	46580614	-0.829934	-1.21246
VIMSS207694	DVU2210	46580615	-0.971334	-1.54229
VIMSS207695	DVU2211	46580616	0.278139	0.427985
VIMSS207696	DVU2212	46580617	1.04109	1.87128
VIMSS207697	DVU2213	46580618	-0.152949	-0.219839
VIMSS207698	DVU2214	46580619	-0.448272	-0.525626
VIMSS207699	DVU2215	46580620	-1.42937	-2.47885
VIMSS207700	DVU2216	46580621	-0.115296	-0.214233
VIMSS207701	DVU2217	46580622	-0.333927	-0.379749
VIMSS207702	DVU2218	46580623	0.890862	1.07543
VIMSS207704	DVU2220	46580625	-1.12878	-1.94456
VIMSS207705	DVU2221	46580626	-0.786478	-1.32423
VIMSS207706	DVU2222	46580627	-1.10275	-1.47522
VIMSS207707	DVU2223	46580628	-1.75196	-2.86468
VIMSS207708	DVU2224	46580629	-0.161544	-0.204419
VIMSS207709	DVU2225	46580630	-0.86019	-1.29486
VIMSS207710	DVU2226	46580631	-1.6679	-2.38042
VIMSS207711	DVU2227	46580632	0.198324	0.317768
VIMSS207712	DVU2228	46580633	0.79248	1.39497
VIMSS207713	DVU2229	46580634	0.772469	1.15908
VIMSS207714	DVU2230	46580635	0.014971	0.0215923
VIMSS207715	DVU2231	46580636	-1.36192	-2.24673
VIMSS207716	DVU2232	46580637	1.62386	2.61481
VIMSS207717	DVU2233	46580638	0.353897	0.600477
VIMSS207718	DVU2234	46580639	-0.163567	-0.214661
VIMSS207719	DVU2235	46580640	0.461705	0.901683
VIMSS207720	DVU2236	46580641	0.759894	1.44159
VIMSS207721	DVU2237	46580642	0.0265498	0.0380348
VIMSS207722	DVU2238	46580643	0.735704	1.22473
VIMSS207723	DVU2239	46580644	1.25579	1.53448
VIMSS207724	DVU2240	46580645	0.451872	0.854435
VIMSS207725	DVU2241	46580646	-1.25846	-1.74631
VIMSS207726	DVU2242	46580647	1.23095	2.00704
VIMSS207727	DVU2243	46580648	-0.533625	-0.738554
VIMSS207728	DVU2244	46580649	1.69704	3.01507
VIMSS207729	DVU2245	46580650	1.35295	2.11158
VIMSS207731	DVU2246	46580651	-0.578347	-0.934408
VIMSS207732	DVU2247	46580652	1.80297	1.468
VIMSS207735	DVU2250	46580655	-0.118853	-0.163023

VIMSS207736	DVU2251	46580656	-0.0154691	-0.02732
VIMSS207737	DVU2252	46580657	-1.18029	-1.39329
VIMSS207738	DVU2253	46580658	-0.592196	-0.561252
VIMSS207739	DVU2254	46580659	-0.66179	-1.08988
VIMSS207740	DVU2255	46580660	0.0868558	0.150541
VIMSS207741	DVU2256	46580661	0.433947	0.745804
VIMSS207742	DVU2257	46580662	-2.84263	-3.46563
VIMSS207743	DVU2258	46580663	-0.972104	-1.75139
VIMSS207744	DVU2259	46580664	-1.94732	-2.59912
VIMSS207745	DVU2260	46580665	-0.874433	-1.37247
VIMSS207746	DVU2261	46580666	-0.870284	-1.43718
VIMSS207748	DVU2263	46580668	-0.904184	-1.39418
VIMSS207749	DVU2264	46580669	0.00201797	0.00350168
VIMSS207752	DVU2267	46580672	-0.953501	-1.40508
VIMSS207753	DVU2268	46580673	1.21395	1.88715
VIMSS207754	DVU2269	46580674	-0.528518	-0.828872
VIMSS207755	DVU2270	46580675	-0.149899	-0.244159
VIMSS207756	DVU2271	46580676	0.415828	0.495723
VIMSS207757	DVU2272	46580677	0.416949	0.566278
VIMSS207759	DVU2274	46580679	-1.20019	-1.78706
VIMSS207760	DVU2275	46580680	-1.68734	-2.34503
VIMSS207761	DVU2276	46580681	-0.783371	-0.77168
VIMSS207762	DVU2277	46580682	-0.282065	-0.398483
VIMSS207763	DVU2278	46580683	1.43357	1.77846
VIMSS207765	DVU2279	46580684	-1.99319	-2.69935
VIMSS207766	DVU2280	46580685	-1.19415	-1.60186
VIMSS207767	DVU2281	46580686	1.69432	2.36381
VIMSS207768	DVU2282	46580687	0.993101	1.50302
VIMSS207769	DVU2283	46580688	2.56798	3.44967
VIMSS207770	DVU2284	46580689	-0.602315	-1.01997
VIMSS207771	DVU2285	46580690	-3.14846	-4.08882
VIMSS207772	DVU2286	46580691	-4.29717	-7.59023
VIMSS207775	DVU2288	46580693	-3.67906	-4.57511
VIMSS207776	DVU2289	46580694	-3.00242	-4.41625
VIMSS207777	DVU2290	304569716	-3.12489	-5.17495
VIMSS207778	DVU2291	46580696	-3.12795	-4.45281
VIMSS207779	DVU2292	46580697	-1.97458	-3.31529
VIMSS207780	DVU2293	46580698	-1.98468	-3.35959
VIMSS207781	DVU2294	46580699	-1.52123	-2.06546
VIMSS207782	DVU2295	46580700	-0.12671	-0.175051
VIMSS207783	DVU2296	46580701	-0.457928	-0.728993
VIMSS207784	DVU2297	46580702	1.61465	2.55037
VIMSS207785	DVU2298	46580703	0.899527	1.63717
VIMSS207786	DVU2299	46580704	0.787492	1.25443
VIMSS207787	DVU2300	46580705	-0.807637	-0.638731
VIMSS207788	DVU2301	46580706	-1.26414	-1.32284
VIMSS207789	DVU2302	46580707	1.28234	1.6188
VIMSS207790	DVU2303	46580708	0.284382	0.520452
VIMSS207792	DVU2305	46580710	-1.89172	-2.3079

VIMSS207793	DVU2306	46580711	-1.54325	-1.80138
VIMSS207794	DVU2307	46580712	1.19747	1.95547
VIMSS207795	DVU2308	46580713	0.969134	1.50016
VIMSS207796	DVU2309	46580714	1.68034	3.11078
VIMSS207797	DVU2310	46580715	-0.434516	-0.498125
VIMSS207799	DVU2312	46580717	1.09305	1.52386
VIMSS207800	DVU2313	46580718	0.787365	1.22827
VIMSS207802	DVU2315	46580720	-0.103435	-0.176456
VIMSS207803	DVU2316	46580721	-0.843005	-0.956282
VIMSS207804	DVU2317	46580722	-0.134804	-0.172637
VIMSS207805	DVU2318	46580723	-1.1007	-1.02349
VIMSS207806			0.661279	0.954362
VIMSS207807	DVU2320	46580725	-0.0789811	-0.134307
VIMSS207809	DVU2322	46580727	0.0830494	0.117314
VIMSS207810	DVU2323	46580728	0.0120877	0.0201371
VIMSS207811	DVU2324	46580729	1.07602	1.39003
VIMSS207812	DVU2325	46580730	0.479876	0.739173
VIMSS207813	DVU2326	46580731	-0.511473	-0.680625
VIMSS207815	DVU2328	46580733	-1.05748	-1.46007
VIMSS207816	DVU2329	46580734	-0.234018	-0.396224
VIMSS207817	DVU2330	46580735	-0.0400634	-0.063411
VIMSS207818	DVU2331	46580736	-0.223589	-0.388565
VIMSS207819	DVU2332	46580737	-0.430508	-0.549446
VIMSS207820	DVU2333	46580738	-1.08531	-1.47031
VIMSS207822	DVU2335	46580740	-1.46087	-2.15297
VIMSS207823	DVU2336	46580741	0.78531	0.911215
VIMSS207824	DVU2337	46580742	0.155918	0.25534
VIMSS207825	DVU2338	46580743	0.593599	0.871939
VIMSS207826	DVU2339	46580744	-0.700268	-1.21656
VIMSS207827	DVU2340	46580745	0.147232	0.189595
VIMSS207828	DVU2341	46580746	0.67446	1.11689
VIMSS207829	DVU2342	46580747	1.56458	2.18181
VIMSS207830	DVU2343	46580748	0.113966	0.166909
VIMSS207833	DVU2345	46580750	3.40065	4.73432
VIMSS207835	DVU2347	46580752	0.155696	0.258359
VIMSS207836			-0.382197	-0.530139
VIMSS207837	DVU2349	46580754	2.46434	3.90338
VIMSS207838	DVU2350	46580755	0.404434	0.591595
VIMSS207839	DVU2351	46580756	0.834314	1.33244
VIMSS207840	DVU2352	46580757	0.467366	0.746988
VIMSS207841			0.757916	1.17827
VIMSS207842	DVU2354	46580759	0.0602012	0.101457
VIMSS207843	DVU2355	46580760	-0.83723	-1.29261
VIMSS207844	DVU2356	46580761	0.518236	0.626203
VIMSS207845	DVU2357	304569717	1.68424	2.56697
VIMSS207847	DVU2359	46580764	0.754245	1.08481
VIMSS207848	DVU2360	46580765	0.720501	1.0846
VIMSS207850	DVU2363	46580768	0.0659596	0.111561
VIMSS207851	DVU2362	46580767	-0.0692306	-0.084884

VIMSS207852	DVU2364	46580769	-1.2391	-1.93861
VIMSS207853	DVU2365	46580770	-0.678401	-0.809815
VIMSS207855	DVU2367	46580772	-1.23518	-1.68179
VIMSS207856	DVU2368	46580773	-0.258796	-0.448755
VIMSS207857	DVU2369	46580774	-0.227776	-0.329254
VIMSS207858	DVU2370	46580775	-0.107092	-0.167638
VIMSS207859	DVU2371	46580776	-0.706188	-1.0463
VIMSS207860	DVU2372	46580777	0.972086	1.4368
VIMSS207861			-1.30727	-2.30062
VIMSS207862	DVU2374	46580779	-1.38122	-2.21237
VIMSS207863	DVU2375	46580780	-1.14436	-1.73343
VIMSS207864			-2.20322	-3.25285
VIMSS207865	DVU2377	46580782	-1.16708	-1.69216
VIMSS207866	DVU2378	46580783	-0.224632	-0.21908
VIMSS207867	DVU2379	46580784	0.55855	0.818166
VIMSS207868	DVU2380	46580785	0.826914	1.02686
VIMSS207869	DVU2381	46580786	-1.0395	-1.2705
VIMSS207870	DVU2382	46580787	-0.0305908	-0.0314438
VIMSS207871	DVU2383	46580788	-0.58208	-0.676089
VIMSS207872	DVU2384	46580789	2.59973	4.36881
VIMSS207873	DVU2385	46580790	-0.19501	-0.196074
VIMSS207874	DVU2386	46580791	0.542285	0.888383
VIMSS207875	DVU2387	46580792	2.67844	2.89534
VIMSS207876	DVU2388	46580793	0.39753	0.562157
VIMSS207877	DVU2389	46580794	-0.310496	-0.419194
VIMSS207879	DVU2390	46580795	0.378505	0.584685
VIMSS207881	DVU2391	46580796	-1.97037	-1.40556
VIMSS207883	DVU2392	46580797	-0.129254	-0.22117
VIMSS207885	DVU2394	46580799	0.596131	0.857418
VIMSS207886	DVU2395	304569720	1.45445	2.24839
VIMSS207887	DVU2396	46580801	0.669995	1.1318
VIMSS207888	DVU2397	46580802	2.58466	3.92572
VIMSS207889	DVU2398	46580803	2.19868	3.32882
VIMSS207890	DVU2399	304569721	2.18538	3.30732
VIMSS207891	DVU2400	46580805	0.840699	1.28036
VIMSS207892	DVU2401	46580806	2.15765	3.39038
VIMSS207893	DVU2402	46580807	1.64843	2.76698
VIMSS207894	DVU2403	46580808	1.57325	2.37838
VIMSS207895	DVU2404	46580809	1.90847	3.5234
VIMSS207898	DVU2407	46580812	-1.05719	-1.22915
VIMSS207899	DVU2408	46580813	0.880319	1.12217
VIMSS207900	DVU2409	46580814	0.077186	0.122144
VIMSS207901			2.45278	3.11767
VIMSS207902	DVU2411	46580816	1.55415	2.32247
VIMSS207903			-0.376435	-0.543872
VIMSS207904	DVU2413	46580818	-0.203241	-0.343142
VIMSS207905	DVU2414	46580819	1.20391	1.42976
VIMSS207907	DVU2416	46580821	1.25991	1.93942
VIMSS207908	DVU2417	46580822	0.940536	1.57579

VIMSS207909	DVU2418	46580823	0.0870414	0.112373
VIMSS207910	DVU2419	46580824	0.954852	1.39998
VIMSS207911	DVU2420	46580825	0.605346	1.12594
VIMSS207912	DVU2421	46580826	2.03841	2.98893
VIMSS207913	DVU2422	46580827	2.17857	3.54771
VIMSS207915	DVU2423	46580828	2.1496	3.54799
VIMSS207916	DVU2424	46580829	2.01803	3.39354
VIMSS207917	DVU2425	46580830	-0.262267	-0.442668
VIMSS207919	DVU2427	46580832	2.2222	3.44024
VIMSS207920	DVU2428	46580833	-0.907493	-1.51678
VIMSS207921	DVU2429	46580834	-0.573652	-0.846203
VIMSS207923	DVU2431	46580836	-0.405947	-0.589331
VIMSS207924	DVU2432	46580837	0.690435	1.07174
VIMSS207926	DVU2434	46580839	-2.46094	-2.8374
VIMSS207927			0.611631	0.954989
VIMSS207928	DVU2436	46580841	-0.781495	-1.06555
VIMSS207929	DVU2437	46580842	-0.10909	-0.126528
VIMSS207932	DVU2439	46580843	1.82042	2.81979
VIMSS207933	DVU2440	46580844	0.0540948	0.0758305
VIMSS207934	DVU2441	46580845	1.33386	1.96999
VIMSS207935	DVU2442	46580846	2.39753	3.64101
VIMSS207936	DVU2443	46580847	0.485463	0.681933
VIMSS207937	DVU2444	46580848	3.4523	5.12585
VIMSS207939	DVU2446	46580850	1.0107	1.71752
VIMSS207940	DVU2447	46580851	0.574503	0.8594
VIMSS207941	DVU2448	46580852	-0.613545	-0.908666
VIMSS207942	DVU2449	46580853	0.288958	0.457401
VIMSS207943	DVU2450	46580854	1.64086	2.62454
VIMSS207944	DVU2451	46580855	0.596451	0.812724
VIMSS207946	DVU2454	46580858	-0.351541	-0.273796
VIMSS207948	DVU2455	46580859	0.637627	0.833372
VIMSS207953	DVU2459	304569723	1.09213	1.5622
VIMSS207954	DVU2460	46580864	-0.318016	-0.369212
VIMSS207955	DVU2461	46580865	-0.302065	-0.325127
VIMSS207956	DVU2462	46580866	-0.374229	-0.402916
VIMSS207957	DVU2463	46580867	-0.390844	-0.559456
VIMSS207958	DVU2464	46580868	-0.141438	-0.222693
VIMSS207960	DVU2466	46580870	-0.748317	-0.886525
VIMSS207961	DVU2467	46580871	0.450609	0.786276
VIMSS207963	DVU2468	46580872	1.33003	2.01432
VIMSS207964	DVU2470	46580874	1.38656	2.40488
VIMSS207967	DVU2472	46580876	1.14776	1.56481
VIMSS207968	DVU2473	46580877	0.479687	0.67463
VIMSS207969	DVU2474	46580878	0.690994	1.17725
VIMSS207970	DVU2475	46580879	0.209655	0.342588
VIMSS207971	DVU2476	46580880	1.77556	3.12973
VIMSS207972	DVU2477	46580881	0.0898145	0.119624
VIMSS207973	DVU2478	46580882	-0.362924	-0.437528
VIMSS207974	DVU2479	46580883	0.279037	0.315929

VIMSS207976	DVU2481	46580885	1.48956	2.38426
VIMSS207980	DVU2483	46580887	1.23538	1.89396
VIMSS207981	DVU2484	46580888	2.063	3.33617
VIMSS207982	DVU2485	46580889	1.78129	2.70161
VIMSS207983	DVU2486	46580890	0.56996	0.724932
VIMSS207984	DVU2487	46580891	0.559142	0.902647
VIMSS207986			0.765194	1.1211
VIMSS207987	DVU2490	46580894	0.963217	1.75337
VIMSS207988	DVU2491	46580895	0.278176	0.423453
VIMSS207989			0.362425	0.496766
VIMSS207990			-1.13406	-1.72582
VIMSS207991	DVU2494	46580898	1.64668	2.22749
VIMSS207992	DVU2495	46580899	0.419653	0.770227
VIMSS207993	DVU2496	46580900	0.837427	1.49899
VIMSS207994	DVU2497	46580901	1.82929	3.44779
VIMSS207995	DVU2498	46580902	0.213868	0.323859
VIMSS207997	DVU2500	46580904	-0.822669	-1.43973
VIMSS207998	DVU2501	46580905	-1.2276	-1.73895
VIMSS207999	DVU2502	46580906	-0.395633	-0.547984
VIMSS208000	DVU2503	46580907	-1.0801	-1.63903
VIMSS208001	DVU2504	46580908	0.211023	0.31867
VIMSS208002	DVU2505	46580909	0.135413	0.160741
VIMSS208003	DVU2506	46580910	-1.0035	-1.43341
VIMSS208004	DVU2507	46580911	-0.389888	-0.514712
VIMSS208005	DVU2508	46580912	-0.176479	-0.274349
VIMSS208006	DVU2509	46580913	-0.0592723	-0.104039
VIMSS208007	DVU2510	46580914	-0.415968	-0.502084
VIMSS208008	DVU2511	46580915	-0.682866	-0.982651
VIMSS208009	DVU2512	46580916	-0.128577	-0.235606
VIMSS208010	DVU2513	46580917	-0.327766	-0.541531
VIMSS208011	DVU2514	46580918	-0.00586853	-0.00789057
VIMSS208012	DVU2515	46580919	1.08516	1.43609
VIMSS208013	DVU2516	46580920	1.00308	1.65669
VIMSS208014	DVU2517	46580921	0.0931898	0.106087
VIMSS208015	DVU2518	46580922	-1.4209	-1.92807
VIMSS208016	DVU2519	46580923	-1.14136	-1.38484
VIMSS208018	DVU2521	46580925	-1.12464	-1.47325
VIMSS208019	DVU2522	46580926	-1.25051	-2.16649
VIMSS208020	DVU2523	46580927	1.27449	1.98282
VIMSS208021	DVU2524	46580928	0.933189	1.06609
VIMSS208022	DVU2525	46580929	-0.483384	-0.571925
VIMSS208023	DVU2526	46580930	0.906162	1.10608
VIMSS208024	DVU2527	46580931	-2.45583	-3.59661
VIMSS208025	DVU2528	46580932	-1.71616	-1.78964
VIMSS208026	DVU2529	46580933	0.346957	0.504965
VIMSS208027	DVU2530	46580934	-0.287617	-0.407438
VIMSS208028	DVU2531	46580935	-0.314162	-0.439145
VIMSS208029	DVU2532	46580936	-0.646842	-1.12182

VIMSS208030	DVU2533	46580937	-1.18963	-1.88001
VIMSS208031	DVU2534	46580938	-1.04269	-1.32265
VIMSS208032	DVU2535	46580939	-1.43099	-1.96191
VIMSS208033	DVU2536	46580940	-0.96441	-1.66662
VIMSS208034	DVU2537	46580941	-1.262	-1.55779
VIMSS208035	DVU2538	46580942	-0.916064	-1.21615
VIMSS208037	DVU2540	46580944	0.114337	0.160244
VIMSS208038	DVU2541	46580945	-0.411239	-0.410261
VIMSS208040	DVU2543	304569724	2.28828	2.62287
VIMSS208041	DVU2544	46580948	1.02638	1.47693
VIMSS208042	DVU2545	46580949	-0.0688427	-0.0917998
VIMSS208043	DVU2546	304569725	0.690714	1.19726
VIMSS208044	DVU2547	46580951	2.10482	3.05035
VIMSS208045	DVU2548	46580952	1.21537	1.82379
VIMSS208046	DVU2549	46580953	0.284986	0.427787
VIMSS208048			0.993531	1.73901
VIMSS208049	DVU2552	46580956	-0.78934	-1.28492
VIMSS208050	DVU2553	46580957	-0.839413	-1.2986
VIMSS208051	DVU2554	46580958	0.12372	0.232065
VIMSS208052	DVU2555	46580959	-0.609882	-0.970309
VIMSS208053	DVU2556	46580960	2.03698	3.1633
VIMSS208054	DVU2557	46580961	1.80073	2.72599
VIMSS208055	DVU2558	46580962	-3.07338	-4.1694
VIMSS208056	DVU2559	46580963	0.51847	0.780769
VIMSS208057	DVU2560	46580964	-0.200662	-0.222434
VIMSS208058	DVU2561	46580965	0.0240153	0.0252258
VIMSS208059	DVU2562	46580966	-0.735979	-0.810908
VIMSS208060	DVU2563	46580967	0.342042	0.345943
VIMSS208061	DVU2564	46580968	1.08019	1.22176
VIMSS208062	DVU2565	46580969	-1.38171	-1.40954
VIMSS208063	DVU2566	46580970	-1.72135	-2.06725
VIMSS208064	DVU2567	46580971	-1.04318	-1.10385
VIMSS208065	DVU2568	46580972	-0.496981	-0.572571
VIMSS208066	DVU2569	46580973	-0.524001	-0.932413
VIMSS208067	DVU2570	46580974	-0.861166	-1.15836
VIMSS208068	DVU2571	46580975	3.18362	3.97
VIMSS208069	DVU2572	46580976	2.75132	3.08257
VIMSS208070	DVU2573	46580977	4.11037	3.64813
VIMSS208071	DVU2574	46580978	3.68627	3.05435
VIMSS208072	DVU2575	46580979	-0.745979	-1.03547
VIMSS208073	DVU2576	46580980	-0.505978	-0.598733
VIMSS208074	DVU2577	46580981	-0.417107	-0.537427
VIMSS208077	DVU2579	46580983	-0.855845	-1.25105
VIMSS208078	DVU2580	46580984	-0.12789	-0.172411
VIMSS208079	DVU2581	46580985	0.485491	0.559579
VIMSS208080	DVU2582	46580986	-1.56333	-1.70589
VIMSS208081	DVU2583	46580987	0.587151	1.03465
VIMSS208082	DVU2584	46580988	-1.15257	-1.41237
VIMSS208083	DVU2585	46580989	1.50764	2.26682

VIMSS208085	DVU2586	46580990	-0.77387	-0.884324
VIMSS208086	DVU2587	46580991	0.581584	0.800005
VIMSS208087	DVU2588	46580992	0.700787	0.996268
VIMSS208089	DVU2590	46580994	0.783636	1.32709
VIMSS208090	DVU2591	46580995	0.554888	0.974169
VIMSS208091	DVU2592	46580996	0.767931	0.764307
VIMSS208094	DVU2595	46580999	1.24337	1.47791
VIMSS208096	DVU2596	46581000	2.50175	2.86477
VIMSS208097	DVU2598	46581002	0.942075	1.11185
VIMSS208099	DVU2600	46581004	1.62357	2.11758
VIMSS208102	DVU2603	46581007	0.480281	0.677156
VIMSS208103	DVU2604	46581008	0.250787	0.391357
VIMSS208104	DVU2605	46581009	0.929536	1.39762
VIMSS208105	DVU2606	46581010	0.40232	0.53736
VIMSS208106	DVU2607	46581011	1.91592	3.61345
VIMSS208108	DVU2609	46581013	0.16527	0.263784
VIMSS208109	DVU2610	46581014	-0.905164	-0.843002
VIMSS208111	DVU2612	46581016	0.813048	1.40052
VIMSS208112	DVU2613	46581017	0.330522	0.532259
VIMSS208114	DVU2615	46581019	2.26757	3.53162
VIMSS208115	DVU2616	46581020	1.30042	1.51535
VIMSS208116	DVU2617	46581021	-0.182841	-0.267825
VIMSS208118	DVU2619	46581023	0.461762	0.611369
VIMSS208119	DVU2620	46581024	0.525393	0.718276
VIMSS208120	DVU2621	46581025	-0.723197	-1.0355
VIMSS208121	DVU2622	46581026	1.23081	1.95643
VIMSS208122	DVU2623	46581027	0.934147	1.33243
VIMSS208123	DVU2624	46581028	0.539634	0.734121
VIMSS208124	DVU2625	46581029	0.753685	1.1429
VIMSS208125	DVU2626	46581030	1.23939	1.58346
VIMSS208127	DVU2628	46581032	0.0337694	0.0533863
VIMSS208128	DVU2629	46581033	-0.115603	-0.151548
VIMSS208129	DVU2630	46581034	1.30533	2.06483
VIMSS208130	DVU2631	46581035	0.768622	1.05227
VIMSS208132			-2.78716	-3.19388
VIMSS208133	DVU2634	46581038	0.795698	0.908644
VIMSS208134	DVU2635	46581039	0.370007	0.508391
VIMSS208137	DVU2638	46581042	0.605595	0.784511
VIMSS208138	DVU2639	46581043	-2.15299	-3.37478
VIMSS208140	DVU2641	46581045	0.165361	0.251865
VIMSS208142	DVU2643	46581047	0.290067	0.420011
VIMSS208143	DVU2644	46581048	-0.0193757	-0.0218287
VIMSS208144	DVU2645	46581049	0.456855	0.695572
VIMSS208145	DVU2646	46581050	0.364412	0.539974
VIMSS208146	DVU2647	46581051	1.27968	1.61483
VIMSS208147	DVU2648	46581052	-1.44357	-2.23638
VIMSS208148	DVU2649	46581053	-1.07808	-1.17673
VIMSS208149	DVU2650	46581054	-3.53608	-6.49219
VIMSS208150	DVU2651	46581055	-0.811896	-0.739466

VIMSS208151	DVU2652	46581056	0.189321	0.327823
VIMSS208154	DVU2655	46581059	0.0367694	0.0616181
VIMSS208156	DVU2657	46581061	1.33874	2.26777
VIMSS208157	DVU2658	46581062	0.0119343	0.0173162
VIMSS208158	DVU2659	46581063	0.675473	1.12865
VIMSS208160	DVU2661	46581065	0.662174	1.1216
VIMSS208162	DVU2663	46581067	1.27994	1.51795
VIMSS208164	DVU2665	46581069	-0.164245	-0.184501
VIMSS208165	DVU2666	46581070	0.119479	0.16237
VIMSS208166	DVU2667	46581071	0.535324	0.60427
VIMSS208167	DVU2668	46581072	-0.347472	-0.518126
VIMSS208168	DVU2669	46581073	-0.0470498	-0.0768522
VIMSS208169	DVU2670	46581074	0.36209	0.53256
VIMSS208170	DVU2671	46581075	-1.35211	-2.25495
VIMSS208172	DVU2673	46581077	-0.339985	-0.518002
VIMSS208173	DVU2674	46581078	-0.0196573	-0.0316064
VIMSS208174	DVU2675	46581079	1.78605	2.90268
VIMSS208175	DVU2676	46581080	1.44771	2.58898
VIMSS208176	DVU2677	46581081	0.00952604	0.0146142
VIMSS208177	DVU2678	46581082	-0.951495	-1.51017
VIMSS208178	DVU2679	46581083	1.30088	2.35781
VIMSS208179	DVU2680	46581084	0.00809728	0.00749384
VIMSS208181	DVU2682	46581086	-1.90935	-2.04985
VIMSS208183	DVU2683	46581087	-0.715371	-1.04857
VIMSS208184			1.18424	2.03058
VIMSS208186	DVU2686	46581090	-0.512265	-0.924689
VIMSS208188			0.803179	1.15802
VIMSS208189	DVU2688	46581092	0.76273	1.12634
VIMSS208190	DVU2689	46581093	0.724688	0.856733
VIMSS208191	DVU2690	46581094	1.43871	1.53147
VIMSS208192	DVU2691	46581095	0.892528	0.861739
VIMSS208193	DVU2692	46581096	1.54863	1.36888
VIMSS208194	DVU2693	46581097	-1.08168	-1.08962
VIMSS208195	DVU2694	46581098	-0.0625324	-0.0926501
VIMSS208197	DVU2696	46581100	-0.562047	-0.800459
VIMSS208198	DVU2697	46581101	-0.57591	-0.944024
VIMSS208199	DVU2698	46581102	0.265774	0.389131
VIMSS208200	DVU2699	46581103	1.54996	2.77894
VIMSS208201	DVU2700	46581104	-0.746862	-0.772092
VIMSS208202	DVU2701	46581105	2.21142	3.24235
VIMSS208203	DVU2702	46581106	0.645811	0.911282
VIMSS208204	DVU2703	46581107	0.448105	0.490672
VIMSS208205	DVU2704	46581108	2.30866	4.17395
VIMSS208206	DVU2705	46581109	0.983234	1.09377
VIMSS208207	DVU2706	46581110	2.57907	3.30824
VIMSS208208	DVU2707	46581111	0.113066	0.184995
VIMSS208209	DVU2708	46581112	0.196771	0.266384
VIMSS208215	DVU2712	46581116	1.02831	1.53627
VIMSS208217	DVU2714	46581118	-0.174322	-0.234858

VIMSS208218	DVU2715	46581119	1.26423	2.1161
VIMSS208219	DVU2716	46581120	0.2903	0.316623
VIMSS208220	DVU2717	46581121	0.0372806	0.0396532
VIMSS208222	DVU2719	46581123	-0.302831	-0.354869
VIMSS208223	DVU2720	46581124	1.48177	2.08119
VIMSS208225	DVU2721	46581125	0.830244	1.0727
VIMSS208226	DVU2722	46581126	0.782784	1.21855
VIMSS208227	DVU2723	46581127	0.403816	0.535668
VIMSS208228	DVU2724	46581128	1.24725	1.3406
VIMSS208229			-0.432826	-0.545557
VIMSS208232	DVU2727	46581131	-0.840528	-1.0131
VIMSS208233	DVU2728	46581132	0.609814	0.954653
VIMSS208234	DVU2729	46581133	0.546022	0.594421
VIMSS208235	DVU2730	46581134	0.216521	0.265499
VIMSS208236	DVU2731	46581135	0.0922705	0.120075
VIMSS208237	DVU2732	46581136	0.829638	1.50756
VIMSS208238	DVU2733	46581137	0.853613	0.954546
VIMSS208241	DVU2735	46581139	-0.125304	-0.174173
VIMSS208242	DVU2736	46581140	1.63268	2.07482
VIMSS208243	DVU2737	46581141	-0.186504	-0.265276
VIMSS208244	DVU2738	46581142	0.572314	0.992533
VIMSS208245			1.49348	2.24919
VIMSS208247	DVU2741	46581145	0.775044	1.267
VIMSS208248	DVU2742	46581146	0.947794	1.18384
VIMSS208249	DVU2743	46581147	-1.38765	-2.27534
VIMSS208250	DVU2744	46581148	0.502542	0.805768
VIMSS208252	DVU2746	46581150	1.11563	1.6402
VIMSS208253	DVU2747	46581151	0.506523	0.650143
VIMSS208254	DVU2748	46581152	-0.0618309	-0.0947781
VIMSS208255	DVU2749	46581153	0.1152	0.138733
VIMSS208256	DVU2750	46581154	-0.419816	-0.760163
VIMSS208257	DVU2751	46581155	-0.951996	-1.5486
VIMSS208258	DVU2752	46581156	0.188731	0.296683
VIMSS208259	DVU2753	46581157	0.359993	0.558578
VIMSS208261	DVU2755	46581159	1.45625	2.04598
VIMSS208262	DVU2756	46581160	0.122741	0.172669
VIMSS208263	DVU2757	46581161	0.434258	0.762121
VIMSS208264	DVU2758	46581162	1.25169	1.04237
VIMSS208266	DVU2760	46581164	1.40856	1.57312
VIMSS208267	DVU2761	46581165	0.622529	0.781288
VIMSS208268	DVU2762	46581166	0.764762	1.15525
VIMSS208269	DVU2764	46581168	0.122897	0.207054
VIMSS208270			0.588117	0.955611
VIMSS208271	DVU2765	46581169	0.254588	0.351807
VIMSS208272	DVU2766	46581170	0.140601	0.186805
VIMSS208273	DVU2767	46581171	0.743474	0.881983
VIMSS208274	DVU2768	46581172	0.567666	0.866499
VIMSS208275	DVU2769	46581173	0.360041	0.397868
VIMSS208276	DVU2770	46581174	1.85005	3.00146

VIMSS208277	DVU2771	46581175	1.10923	1.85061
VIMSS208278	DVU2772	46581176	-0.724257	-1.26586
VIMSS208279	DVU2773	46581177	-0.0424016	-0.0546456
VIMSS208280	DVU2774	46581178	0.789244	1.39471
VIMSS208281	DVU2775	46581179	-0.334344	-0.388947
VIMSS208282	DVU2776	46581180	2.36369	3.18627
VIMSS208285	DVU2779	46581183	-0.104738	-0.13016
VIMSS208286	DVU2780	46581184	0.688799	0.856466
VIMSS208287	DVU2781	46581185	1.38111	2.06703
VIMSS208289	DVU2783	46581187	0.446481	0.778977
VIMSS208290	DVU2784	46581188	0.635646	0.982087
VIMSS208291	DVU2785	46581189	-0.467212	-0.673799
VIMSS208293	DVU2787	46581191	1.38201	1.80655
VIMSS208294	DVU2788	46581192	2.89708	3.38083
VIMSS208295	DVU2789	46581193	0.207377	0.315714
VIMSS208296	DVU2790	46581194	0.147279	0.200184
VIMSS208298	DVU2792	46581196	1.97094	3.37241
VIMSS208299	DVU2793	46581197	2.60755	4.59305
VIMSS208300	DVU2794	46581198	2.70024	4.50957
VIMSS208301	DVU2795	46581199	1.71358	2.6763
VIMSS208302	DVU2796	46581200	2.51161	3.95249
VIMSS208303	DVU2797	46581201	1.4234	2.54991
VIMSS208304	DVU2798	46581202	2.94467	5.07444
VIMSS208305	DVU2799	46581203	0.702636	1.22877
VIMSS208306	DVU2800	46581204	1.25717	2.19445
VIMSS208309			-0.331141	-0.597196
VIMSS208310	DVU2802	46581206	0.50512	0.660647
VIMSS208311	DVU2803	46581207	1.54449	2.82129
VIMSS208313	DVU2805	304569730	1.00656	1.11722
VIMSS208314	DVU2806	46581210	0.961981	1.39064
VIMSS208315	DVU2807	46581211	0.0605563	0.0558716
VIMSS208317	DVU2809	46581213	0.210695	0.335957
VIMSS208318	DVU2810	304569731	-0.511534	-0.537731
VIMSS208319	DVU2811	46581215	0.254137	0.345515
VIMSS208324	DVU2815	46581219	0.129066	0.180366
VIMSS208325	DVU2816	46581220	-0.906771	-1.08698
VIMSS208326	DVU2818	46581222	-1.29358	-2.26053
VIMSS208327	DVU2817	46581221	0.152043	0.217101
VIMSS208328	DVU2819	46581223	0.19684	0.319419
VIMSS208330	DVU2821	46581225	-0.277707	-0.269029
VIMSS208331	DVU2822	46581226	-0.795934	-1.15677
VIMSS208332	DVU2823	46581227	-1.84548	-2.84616
VIMSS208333	DVU2824	46581228	-0.9879	-1.4175
VIMSS208334	DVU2825	46581229	0.457872	0.575772
VIMSS208336	DVU2827	46581231	0.265754	0.465806
VIMSS208338	DVU2829	46581233	-0.0952158	-0.141873
VIMSS208339	DVU2830	46581234	0.848818	1.137
VIMSS208340	DVU2831	46581235	0.708741	1.23499
VIMSS208341	DVU2832	46581236	0.834944	0.946958

VIMSS208342	DVU2833	46581237	0.784706	1.04988
VIMSS208344	DVU2835	46581239	-0.720887	-1.10765
VIMSS208345	DVU2836	46581240	0.286894	0.476673
VIMSS208346	DVU2837	46581241	-0.741636	-1.34135
VIMSS208347	DVU2838	46581242	-1.30126	-1.95733
VIMSS208348	DVU2839	46581243	-0.802241	-1.30055
VIMSS208350	DVU2841	46581245	-0.383014	-0.553166
VIMSS208351	DVU2842	46581246	-0.196734	-0.351835
VIMSS208352	DVU2843	46581247	-1.01772	-1.57788
VIMSS208353	DVU2844	46581248	-1.06112	-1.4932
VIMSS208354	DVU2845	46581249	0.467033	0.793798
VIMSS208355	DVU2846	46581250	0.801249	1.0529
VIMSS208356	DVU2847	46581251	0.554042	0.657811
VIMSS208358	DVU2849	46581253	0.454445	0.600132
VIMSS208359	DVU2850	46581254	0.371387	0.50775
VIMSS208360	DVU2851	46581255	0.392834	0.641737
VIMSS208361	DVU2852	46581256	0.769203	0.922745
VIMSS208362	DVU2853	46581257	0.676716	0.739537
VIMSS208363	DVU2854	46581258	1.10234	1.51962
VIMSS208364	DVU2855	46581259	1.11164	1.65004
VIMSS208365	DVU2856	46581260	1.27957	1.00167
VIMSS208366	DVU2858	46581262	-0.076897	-0.140135
VIMSS208367	DVU2857	46581261	-0.343877	-0.311982
VIMSS208368	DVU2859	46581263	-0.498977	-0.587287
VIMSS208369	DVU2860	46581264	1.22991	1.83133
VIMSS208370	DVU2861	46581265	0.686731	0.990987
VIMSS208371	DVU2862	46581266	-0.409152	-0.600479
VIMSS208372	DVU2863	46581267	-0.742355	-1.01698
VIMSS208373	DVU2864	46581268	0.31943	0.547839
VIMSS208374	DVU2865	46581269	2.72321	3.58662
VIMSS208375	DVU2866	46581270	-0.823803	-1.26988
VIMSS208376	DVU2867	46581271	-1.12237	-1.54647
VIMSS208377	DVU2868	46581272	1.26248	1.63097
VIMSS208378	DVU2869	46581273	-0.483315	-0.721952
VIMSS208379	DVU2870	46581274	-0.714153	-1.00764
VIMSS208380	DVU2871	46581275	-1.36502	-1.8442
VIMSS208381	DVU2872	46581276	-1.2794	-2.048
VIMSS208382	DVU2873	46581277	0.848082	1.2438
VIMSS208383	DVU2874	46581278	2.39814	2.84578
VIMSS208384	DVU2875	46581279	0.0958339	0.0958666
VIMSS208385	DVU2876	46581280	1.11442	1.69544
VIMSS208386	DVU2877	46581281	-0.376596	-0.472808
VIMSS208387	DVU2878	46581282	0.614995	0.965577
VIMSS208388	DVU2879	46581283	2.86617	1.4314
VIMSS208389	DVU2880	46581284	-1.03351	-1.07315
VIMSS208390	DVU2881	46581285	0.714001	0.789488
VIMSS208391	DVU2882	46581286	-1.60546	-2.40112
VIMSS208392	DVU2883	46581287	0.554705	1.04165
VIMSS208393	DVU2884	46581288	-0.765038	-1.3489

VIMSS208394	DVU2885	46581289	0.397182	0.516218
VIMSS208395	DVU2886	46581290	-0.106835	-0.147386
VIMSS208396	DVU2887	46581291	-0.116898	-0.16171
VIMSS208397	DVU2888	46581292	-0.189437	-0.280788
VIMSS208398	DVU2889	46581293	-0.277395	-0.432039
VIMSS208399			0.181789	0.21577
VIMSS208400	DVU2891	46581295	-0.190087	-0.263613
VIMSS208401	DVU2892	304569732	-0.529022	-0.884418
VIMSS208402	DVU2893	46581297	0.233666	0.399565
VIMSS208403	DVU2894	46581298	1.12872	1.92383
VIMSS208404	DVU2895	46581299	1.41335	2.18913
VIMSS208405	DVU2896	46581300	0.540306	0.947935
VIMSS208406	DVU2897	46581301	1.73912	2.9341
VIMSS208407	DVU2898	46581302	1.05931	1.64065
VIMSS208408	DVU2899	46581303	0.169638	0.228181
VIMSS208409	DVU2900	46581304	0.248452	0.259059
VIMSS208411	DVU2901	46581305	0.229495	0.295117
VIMSS208412	DVU2902	46581306	-0.01456	-0.0210141
VIMSS208413	DVU2903	46581307	-0.479246	-0.694383
VIMSS208415	DVU2905	46581309	0.635545	0.815039
VIMSS208416	DVU2906	46581310	0.969553	1.02897
VIMSS208417	DVU2907	46581311	1.1857	1.3877
VIMSS208418	DVU2908	46581312	-0.420215	-0.493004
VIMSS208419	DVU2909	46581313	0.578425	0.741531
VIMSS208420	DVU2910	46581314	-2.82234	-2.94504
VIMSS208421	DVU2911	46581315	-0.234242	-0.307917
VIMSS208422	DVU2912	46581316	-1.44734	-2.06042
VIMSS208423	DVU2913	46581317	-1.89853	-3.43179
VIMSS208424	DVU2914	46581318	-1.31707	-1.72014
VIMSS208425	DVU2915	46581319	1.6677	2.53305
VIMSS208426	DVU2916	46581320	-0.938888	-1.18591
VIMSS208427	DVU2917	46581321	-2.70865	-3.589
VIMSS208428	DVU2918	46581322	-0.0645988	-0.079417
VIMSS208430	DVU2920	46581324	-1.20224	-1.11636
VIMSS208431	DVU2921	46581325	-1.86536	-2.52238
VIMSS208432	DVU2922	46581326	-3.08132	-4.0789
VIMSS208433	DVU2923	46581327	-2.4705	-3.5947
VIMSS208434	DVU2924	46581328	-2.32561	-2.65159
VIMSS208435	DVU2925	46581329	-2.48945	-3.38947
VIMSS208436	DVU2926	46581330	-1.95455	-2.19003
VIMSS208437	DVU2927	46581331	-2.66831	-3.82851
VIMSS208438	DVU2928	46581332	-0.310421	-0.503859
VIMSS208439	DVU2929	46581333	-0.802808	-1.3532
VIMSS208441	DVU2931	46581335	-0.362107	-0.560039
VIMSS208442	DVU2932	46581336	0.658675	0.900439
VIMSS208443	DVU2933	46581337	-0.15103	-0.218309
VIMSS208444	DVU2934	46581338	-0.10878	-0.146948
VIMSS208445	DVU2935	46581339	1.37488	2.18482
VIMSS208447	DVU2937	46581341	1.18714	1.92132

VIMSS208448	DVU2938	46581342	0.807774	1.30603
VIMSS208449	DVU2939	46581343	0.0459894	0.0516102
VIMSS208450			1.27635	1.72478
VIMSS208451	DVU2941	46581345	-0.896981	-1.13815
VIMSS208452	DVU2942	46581346	-0.879207	-1.34102
VIMSS208453	DVU2943	46581347	0.314225	0.467083
VIMSS208454	DVU2944	46581348	0.455453	0.563997
VIMSS208455	DVU2945	46581349	-0.224406	-0.362987
VIMSS208456	DVU2946	46581350	-0.424222	-0.625407
VIMSS208458	DVU2948	46581352	0.415448	0.568585
VIMSS208459	DVU2949	46581353	-1.56707	-1.86569
VIMSS208462	DVU2951	46581354	0.231006	0.433481
VIMSS208463	DVU2952	46581355	0.929357	1.19747
VIMSS208464	DVU2953	46581356	-0.915611	-1.13402
VIMSS208465	DVU2954	46581357	0.134378	0.14233
VIMSS208467	DVU2956	46581359	-0.299494	-0.43188
VIMSS208470	DVU2957	46581360	-3.64861	-3.33911
VIMSS208471	DVU2958	46581361	-1.8658	-2.55993
VIMSS208472	DVU2959	46581362	-2.6838	-3.23277
VIMSS208473	DVU2960	46581363	-0.910455	-1.37738
VIMSS208474	DVU2961	46581364	-0.10608	-0.160197
VIMSS208475	DVU2962	46581365	-0.304478	-0.437595
VIMSS208476	DVU2963	46581366	-0.738466	-0.959012
VIMSS208477	DVU2964	46581367	-0.673649	-0.809688
VIMSS208478	DVU2965	46581368	0.503602	0.784009
VIMSS208479	DVU2966	46581369	-0.508756	-0.736107
VIMSS208480	DVU2967	46581370	0.191677	0.291314
VIMSS208481	DVU2968	46581371	1.62921	1.88001
VIMSS208482	DVU2969	46581372	1.18284	1.82678
VIMSS208483	DVU2970	46581373	0.967028	1.44655
VIMSS208484	DVU2971	46581374	0.0913573	0.149367
VIMSS208486	DVU2973	46581376	1.47342	2.71957
VIMSS208487	DVU2974	46581377	0.608174	0.99661
VIMSS208488	DVU2975	46581378	2.52493	3.20361
VIMSS208489	DVU2976	46581379	0.807955	1.27692
VIMSS208492	DVU2979	46581382	0.258356	0.434136
VIMSS208493	DVU2980	46581383	0.312539	0.457664
VIMSS208494	DVU2981	46581384	0.140764	0.180511
VIMSS208495	DVU2982	46581385	-0.522011	-0.706565
VIMSS208496	DVU2983	46581386	-0.172725	-0.295016
VIMSS208497	DVU2984	46581387	0.289899	0.441513
VIMSS208498	DVU2985	46581388	0.298871	0.490008
VIMSS208499	DVU2986	46581389	1.24007	1.62429
VIMSS208500	DVU2987	46581390	1.31784	1.67309
VIMSS208501	DVU2988	46581391	0.308087	0.4578
VIMSS208502	DVU2989	46581392	0.412656	0.533606
VIMSS208503	DVU2990	46581393	0.612172	0.988785
VIMSS208504			-0.347617	-0.547772
VIMSS208505	DVU2992	46581395	0.555082	0.735797

VIMSS208506	DVU2993	46581396	0.439928	0.575332
VIMSS208508	DVU2995	46581398	0.788836	1.10653
VIMSS208509	DVU2996	46581399	0.175685	0.255354
VIMSS208510	DVU2997	46581400	0.959043	1.13091
VIMSS208512	DVU2999	46581402	0.440977	0.65577
VIMSS208513	DVU3000	46581403	0.6483	0.808343
VIMSS208514	DVU3001	46581404	0.419553	0.628053
VIMSS208515	DVU3002	46581405	0.0616121	0.10346
VIMSS208516	DVU3003	46581406	0.822047	1.39423
VIMSS208517			0.37089	0.546223
VIMSS208518	DVU3005	46581408	-0.110362	-0.174281
VIMSS208519	DVU3006	46581409	-0.900015	-1.40872
VIMSS208520	DVU3007	46581410	1.43543	1.54343
VIMSS208521	DVU3008	46581411	-1.1564	-1.71841
VIMSS208522	DVU3009	46581412	0.695746	1.08351
VIMSS208523	DVU3010	46581413	0.857717	1.27727
VIMSS208524	DVU3011	46581414	0.950079	1.49279
VIMSS208525	DVU3012	46581415	1.7254	2.66453
VIMSS208526	DVU3013	46581416	0.589199	0.991559
VIMSS208527	DVU3014	46581417	0.984841	1.19883
VIMSS208528	DVU3015	46581418	0.700974	0.914757
VIMSS208529	DVU3016	46581419	0.289039	0.438058
VIMSS208530			0.0643556	0.0920231
VIMSS208531	DVU3018	46581421	0.755491	1.19169
VIMSS208532	DVU3019	46581422	0.698529	1.12818
VIMSS208533	DVU3020	46581423	0.89269	1.32615
VIMSS208534	DVU3021	46581424	1.10025	1.47153
VIMSS208536	DVU3022	46581425	0.146539	0.22388
VIMSS208537	DVU3023	46581426	-0.960924	-1.15854
VIMSS208538	DVU3024	46581427	-2.21157	-2.4716
VIMSS208539	DVU3025	46581428	-3.08681	-3.63512
VIMSS208540	DVU3026	46581429	1.67823	2.44832
VIMSS208541	DVU3027	46581430	-2.0643	-2.60142
VIMSS208542	DVU3028	46581431	-0.461076	-0.787914
VIMSS208543	DVU3029	46581432	-0.683723	-1.23421
VIMSS208544	DVU3030	46581433	-2.61806	-3.67187
VIMSS208545	DVU3031	46581434	-1.91816	-2.32525
VIMSS208546	DVU3032	46581435	-2.60121	-3.81583
VIMSS208547	DVU3033	46581436	-2.41814	-3.68931
VIMSS208550	DVU3035	304569734	-0.250789	-0.432924
VIMSS208551	DVU3036	46581439	1.05904	1.67212
VIMSS208552	DVU3037	46581440	0.809237	1.35654
VIMSS208554	DVU3039	46581442	-0.0495616	-0.0729288
VIMSS208556	DVU3041	46581444	1.24269	2.09128
VIMSS208557	DVU3042	46581445	1.69579	2.87807
VIMSS208561	DVU3046	46581449	-0.182037	-0.219202
VIMSS208563	DVU3048	46581451	0.0188745	0.0249328
VIMSS208564	DVU3049	46581452	0.342125	0.607145
VIMSS208565	DVU3050	46581453	0.217902	0.30669

VIMSS208566	DVU3051	46581454	-0.0728985	-0.101364
VIMSS208567	DVU3052	46581455	0.560112	0.625489
VIMSS208568	DVU3053	46581456	-0.787177	-1.32986
VIMSS208569	DVU3054	46581457	1.95629	2.72978
VIMSS208570	DVU3055	46581458	-0.435233	-0.508256
VIMSS208571	DVU3056	46581459	-0.591066	-0.952422
VIMSS208572	DVU3057	46581460	1.63348	2.26067
VIMSS208573	DVU3058	46581461	0.637685	0.787893
VIMSS208574	DVU3059	46581462	-0.0524799	-0.0846957
VIMSS208576	DVU3061	46581464	0.794623	1.26065
VIMSS208577	DVU3062	46581465	1.94046	2.67711
VIMSS208578	DVU3063	46581466	1.48938	1.77745
VIMSS208579	DVU3064	46581467	-0.223652	-0.307353
VIMSS208580	DVU3065	46581468	-0.393678	-0.71229
VIMSS208581	DVU3066	46581469	-0.7241	-1.23724
VIMSS208582			0.798713	1.12961
VIMSS208584	DVU3068	46581471	0.720255	1.25062
VIMSS208586	DVU3070	46581473	-1.51142	-1.96162
VIMSS208587	DVU3071	46581474	-0.971228	-1.65041
VIMSS208588	DVU3072	46581475	-0.282507	-0.321278
VIMSS208590	DVU3074	46581477	0.100714	0.123509
VIMSS208592	DVU3076	46581479	1.94289	3.5592
VIMSS208593	DVU3077	46581480	2.41457	4.49129
VIMSS208595	DVU3079	46581482	0.49184	0.762709
VIMSS208596	DVU3080	46581483	-0.537036	-0.892349
VIMSS208597	DVU3081	46581484	-3.00135	-3.13932
VIMSS208598	DVU3082	46581485	0.0930177	0.152972
VIMSS208600	DVU3084	46581487	-0.593394	-1.04543
VIMSS208601	DVU3085	46581488	0.798224	1.35657
VIMSS208602	DVU3086	46581489	-0.270532	-0.315336
VIMSS208603	DVU3087	46581490	0.491537	0.542015
VIMSS208604	DVU3088	46581491	-0.390722	-0.578484
VIMSS208605	DVU3089	46581492	-1.009	-1.44892
VIMSS208606	DVU3090	46581493	-1.84798	-3.10206
VIMSS208607	DVU3091	46581494	0.69697	0.921625
VIMSS208609	DVU3092	46581495	-1.05674	-1.58813
VIMSS208610	DVU3093	46581496	1.57232	2.22533
VIMSS208611	DVU3094	46581497	0.533645	0.668331
VIMSS208612	DVU3095	46581498	0.146497	0.119023
VIMSS208614	DVU3097	46581500	0.0865615	0.14995
VIMSS208615	DVU3098	46581501	0.303153	0.391722
VIMSS208616	DVU3099	46581502	-1.0958	-1.62166
VIMSS208617	DVU3100	46581503	-0.702934	-1.07126
VIMSS208618	DVU3101	46581504	-1.10244	-1.69705
VIMSS208619	DVU3102	46581505	-0.364975	-0.436599
VIMSS208620	DVU3103	46581506	-2.28588	-2.65723
VIMSS208621	DVU3104	46581507	1.08926	1.66845
VIMSS208624			0.273402	0.304123
VIMSS208625	DVU3107	46581510	1.77582	1.96019

VIMSS208626	DVU3108	46581511	-0.601243	-0.680629
VIMSS208627	DVU3109	46581512	-0.811791	-1.02797
VIMSS208628	DVU3110	46581513	0.28101	0.276387
VIMSS208629			0.143719	0.204336
VIMSS208630	DVU3112	46581515	0.547899	1.01206
VIMSS208631	DVU3113	46581516	-0.866713	-1.5525
VIMSS208632	DVU3114	46581517	0.105079	0.19235
VIMSS208635	DVU3117	46581520	2.07856	3.57414
VIMSS208637	DVU3119	46581522	-0.636058	-0.948601
VIMSS208639	DVU3121	46581524	0.548153	0.727319
VIMSS208640	DVU3122	46581525	-0.117422	-0.106245
VIMSS208641	DVU3123	46581526	0.217991	0.219061
VIMSS208643	DVU3125	46581528	0.513823	0.875116
VIMSS208645	DVU3126		1.06813	1.33259
VIMSS208646	DVU3127	46581529	0.872243	1.21314
VIMSS208647	DVU3128	46581530	1.24118	2.13288
VIMSS208648	DVU3129	46581531	0.425163	0.596437
VIMSS208649	DVU3130	46581532	-0.616993	-0.521273
VIMSS208650	DVU3131	46581533	2.1512	2.96775
VIMSS208651	DVU3132	46581534	1.68086	2.7023
VIMSS208652	DVU3133	46581535	2.6814	3.36896
VIMSS208653	DVU3134	46581536	2.33725	3.7049
VIMSS208655	DVU3136	46581538	0.948021	1.54887
VIMSS208656	DVU3137	46581539	1.30132	1.62692
VIMSS208659	DVU3140	46581542	-0.372742	-0.495924
VIMSS208661	DVU3142	46581544	1.67919	2.45303
VIMSS208662	DVU3143	46581545	1.26036	1.68062
VIMSS208663	DVU3144	46581546	1.24783	1.55222
VIMSS208664	DVU3145	46581547	1.1606	1.47451
VIMSS208665	DVU3146	46581548	0.666124	1.18324
VIMSS208667	DVU3148	46581550	1.13454	1.5907
VIMSS208668	DVU3149	46581551	0.48603	0.719076
VIMSS208669	DVU3150	46581552	-0.301147	-0.548065
VIMSS208670	DVU3151	46581553	0.192794	0.212333
VIMSS208672	DVU3153	46581555	0.692731	0.943233
VIMSS208673	DVU3154	46581556	0.0377189	0.0602825
VIMSS208674	DVU3155	46581557	0.676703	1.00493
VIMSS208676	DVU3157	46581559	0.397592	0.570814
VIMSS208677	DVU3158	46581560	1.57774	2.65533
VIMSS208681	DVU3161	46581563	-0.467804	-0.471939
VIMSS208683	DVU3163	46581565	1.31139	2.15866
VIMSS208684	DVU3164	46581566	0.385279	0.698593
VIMSS208685	DVU3165	46581567	-0.249483	-0.396146
VIMSS208686	DVU3166	46581568	0.747307	0.81704
VIMSS208688	DVU3168	46581570	-1.03999	-1.74288
VIMSS208689	DVU3169	46581571	0.936352	1.61202
VIMSS208691	DVU3170	46581572	0.713083	0.907607
VIMSS208693			-0.0376459	-0.0620572
VIMSS208694	DVU3173	46581575	-0.739804	-1.2163

VIMSS208695	DVU3174	46581576	-0.0227128	-0.0405092
VIMSS208696	DVU3175	46581577	-1.53133	-2.35646
VIMSS208697	DVU3176	46581578	-0.233619	-0.402086
VIMSS208698	DVU3177	46581579	0.0369229	0.0617954
VIMSS208699			0.241505	0.424797
VIMSS208700	DVU3179	46581581	0.172622	0.287056
VIMSS208701	DVU3180	46581582	0.314716	0.433955
VIMSS208702	DVU3181	304569735	-0.691691	-1.00827
VIMSS208703	DVU3182	46581584	1.95426	2.58218
VIMSS208705	DVU3184	46581586	-0.686644	-1.07396
VIMSS208707	DVU3186	46581588	0.667024	0.961756
VIMSS208708	DVU3187	46581589	0.715507	1.09551
VIMSS208709	DVU3188	46581590	0.607856	0.745721
VIMSS208710	DVU3189	46581591	-0.882964	-1.5705
VIMSS208711	DVU3190	46581592	-0.368754	-0.609601
VIMSS208712	DVU3191	46581593	-0.842282	-1.20048
VIMSS208713	DVU3192	46581594	-0.100895	-0.161579
VIMSS208714			0.0732173	0.128171
VIMSS208715	DVU3194	46581596	-0.271349	-0.34324
VIMSS208716	DVU3195	46581597	0.62589	0.822033
VIMSS208717			0.599361	0.875531
VIMSS208718	DVU3197	46581599	0.803924	1.49818
VIMSS208719	DVU3198	46581600	-0.661931	-1.03295
VIMSS208720	DVU3199	46581601	-1.43935	-2.06549
VIMSS208721	DVU3200	46581602	-0.545839	-0.877709
VIMSS208722	DVU3201	46581603	2.12148	2.9476
VIMSS208723	DVU3202	46581604	-0.441879	-0.500185
VIMSS208724			-0.103354	-0.152566
VIMSS208725	DVU3204	46581606	-0.31612	-0.490853
VIMSS208726	DVU3205	46581607	0.105432	0.146521
VIMSS208728			-1.26819	-1.61385
VIMSS208729	DVU3207	46581609	-0.420304	-0.528089
VIMSS208730	DVU3208	46581610	-0.305348	-0.471404
VIMSS208732			-0.291606	-0.446554
VIMSS208734	DVU3212	46581614	-1.46386	-1.8373
VIMSS208735	DVU3213	46581615	0.889644	1.23198
VIMSS208736	DVU3214	46581616	1.05024	1.62722
VIMSS208737	DVU3215	46581617	0.502768	0.585807
VIMSS208738	DVU3216	46581618	0.914022	1.25067
VIMSS208739	DVU3217	46581619	1.74798	3.08061
VIMSS208740	DVU3218	46581620	0.813223	1.22442
VIMSS208741	DVU3219	304569737	0.458459	0.776486
VIMSS208742	DVU3220	46581622	0.399134	0.650428
VIMSS208743	DVU3221	46581623	0.42466	0.739118
VIMSS208744	DVU3222	46581624	1.19147	1.82528
VIMSS208745	DVU3223	46581625	0.268443	0.372062
VIMSS208746	DVU3224	46581626	-0.165474	-0.188481
VIMSS208747	DVU3225	46581627	1.22224	1.88155
VIMSS208748	DVU3226	46581628	0.476512	0.751469

VIMSS208750	DVU3228	46581630	0.29644	0.455285
VIMSS208752	DVU3230	46581632	0.0728221	0.0890478
VIMSS208754	DVU3232	46581634	-0.214567	-0.334192
VIMSS208755	DVU3233	46581635	-1.35431	-1.64961
VIMSS208757	DVU3234	46581636	0.00276702	0.00371084
VIMSS208758	DVU3235	46581637	-0.159306	-0.224099
VIMSS208759	DVU3236	46581638	-0.614116	-0.712075
VIMSS208761	DVU3238	46581640	0.0303899	0.0414012
VIMSS208762	DVU3239	46581641	-0.278162	-0.434537
VIMSS208763			-0.190114	-0.252932
VIMSS208764	DVU3241	46581643	-0.353379	-0.400357
VIMSS208765	DVU3242	46581644	1.16173	2.00308
VIMSS208766	DVU3243	46581645	-1.3	-1.70963
VIMSS208768	DVU3245	46581647	-0.329959	-0.626975
VIMSS208769	DVU3246	46581648	1.54999	2.1944
VIMSS208771	DVU3247	46581649	1.08918	1.61161
VIMSS208772	DVU3248	46581650	1.37137	1.73814
VIMSS208774			0.318892	0.373201
VIMSS208775	DVU3251	46581653	0.943414	0.947566
VIMSS208776	DVU3252	46581654	0.236616	0.332882
VIMSS208777	DVU3253	46581655	-0.337063	-0.525164
VIMSS208778	DVU3254	46581656	1.50964	2.12202
VIMSS208779	DVU3255	46581657	-0.0733212	-0.0846626
VIMSS208780	DVU3256	46581658	0.239677	0.246021
VIMSS208782	DVU3258	46581660	0.231661	0.29422
VIMSS208783	DVU3259	46581661	0.409188	0.66122
VIMSS208784			0.55689	0.626818
VIMSS208785	DVU3261	46581663	1.01446	1.43108
VIMSS208786	DVU3262	46581664	0.20377	0.245559
VIMSS208787	DVU3263	46581665	1.36445	1.81475
VIMSS208788	DVU3264	46581666	0.281191	0.390777
VIMSS208790	DVU3266	46581668	1.68349	2.74939
VIMSS208791			0.010825	0.0178686
VIMSS208792	DVU3268	46581670	0.0639599	0.0985982
VIMSS208793	DVU3269	46581671	0.668208	0.850734
VIMSS208794	DVU3270	46581672	2.52744	3.23232
VIMSS208795	DVU3271	46581673	2.20125	3.57759
VIMSS208796	DVU3272	46581674	-1.3377	-1.93915
VIMSS208797	DVU3273	46581675	-0.785856	-1.00343
VIMSS208798	DVU3274	46581676	0.350022	0.598823
VIMSS208799	DVU3275	46581677	0.0731116	0.132838
VIMSS208800	DVU3276	46581678	0.0921912	0.0796747
VIMSS208802	DVU3278	46581680	0.533984	0.803203
VIMSS208803	DVU3279	46581681	-0.909889	-1.17656
VIMSS208806	DVU3281	46581682	-0.656778	-0.891643
VIMSS208807	DVU3282	46581683	1.13345	1.53323
VIMSS208809	DVU3283	46581684	-0.0720875	-0.109874
VIMSS208810	DVU3284	46581685	0.461408	0.553721
VIMSS208811	DVU3285	46581686	-0.094293	-0.10173

VIMSS208813			-1.15948	-1.3531
VIMSS208816	DVU3289	46581690	-0.234887	-0.275999
VIMSS208817	DVU3290	46581691	-0.194659	-0.263298
VIMSS208818	DVU3291	46581692	0.441093	0.530523
VIMSS208819	DVU3292	46581693	-0.00582798	-0.0064549
VIMSS208820	DVU3293	46581694	1.45629	2.34052
VIMSS208821	DVU3294	46581695	0.816825	1.19934
VIMSS208822	DVU3295	46581696	2.62981	3.04839
VIMSS208823	DVU3296	46581697	1.67122	2.41087
VIMSS208824	DVU3297	46581698	-0.998525	-1.37856
VIMSS208825	DVU3298	46581699	0.836666	1.09287
VIMSS208826	DVU3299	46581700	0.360291	0.539922
VIMSS208827	DVU3300	46581701	1.02286	1.21615
VIMSS208828	DVU3301	46581702	0.356332	0.349069
VIMSS208829			-0.598548	-0.680818
VIMSS208830	DVU3303	46581704	1.34361	1.85308
VIMSS208832	DVU3305	46581705	0.0527269	0.0622666
VIMSS208833	DVU3306	46581706	-1.06971	-1.27827
VIMSS208834	DVU3307	46581707	-1.297	-1.75079
VIMSS208835	DVU3308	46581708	-0.409155	-0.447721
VIMSS208836	DVU3310	46581710	-2.20818	-2.45102
VIMSS208837	DVU3311	46581711	-1.31919	-1.69265
VIMSS208838	DVU3312	46581712	-0.16603	-0.177966
VIMSS208839	DVU3313	46581713	-0.699525	-0.736992
VIMSS208840	DVU3314	46581714	0.681476	0.675597
VIMSS208841	DVU3315	46581715	0.20369	0.380162
VIMSS208842	DVU3316	46581716	0.59257	0.772411
VIMSS208844	DVU3318	46581718	-0.827603	-1.17811
VIMSS208845	DVU3319	46581719	1.45436	2.56651
VIMSS208846	DVU3320	46581720	0.359665	0.421872
VIMSS208848	DVU3322	46581722	0.211503	0.203453
VIMSS208849	DVU3323	46581723	0.275588	0.418056
VIMSS208850	DVU3324	46581724	-0.486609	-0.404653
VIMSS208851	DVU3325	46581725	0.571641	0.863169
VIMSS208852	DVU3326	46581726	1.16448	1.21595
VIMSS208855	DVU3329	46581729	0.345353	0.576467
VIMSS208856	DVU3330	46581730	-0.116235	-0.157714
VIMSS208857	DVU3331	46581731	0.825145	1.03879
VIMSS208858	DVU3332	46581732	-0.378531	-0.492686
VIMSS208860	DVU3334	46581734	-0.301797	-0.348734
VIMSS208861	DVU3335	46581735	-0.229808	-0.360544
VIMSS208862	DVU3336	46581736	0.675363	0.819699
VIMSS208863	DVU3337	46581737	0.340312	0.431771
VIMSS208864	DVU3338	46581738	0.782062	1.05574
VIMSS208868	DVU3342	46581743	0.057053	0.0840912
VIMSS208869	DVU3343	46581744	-0.853118	-1.09462
VIMSS208870	DVU3344	46581745	0.518195	0.565755
VIMSS208874	DVU3347	46581748	-1.48195	-2.34232

VIMSS208875	DVU3348	304569740	-2.30449	-3.01116
VIMSS208876	DVU3349	46581750	-1.54983	-1.87275
VIMSS208877	DVU3350	46581751	-1.53737	-2.10124
VIMSS208878	DVU3351	46581752	-0.632901	-0.869015
VIMSS208879	DVU3352	46581753	0.518304	0.727764
VIMSS208882	DVU3355	46581756	2.1196	2.42468
VIMSS208883	DVU3356	46581757	0.286188	0.374057
VIMSS208884	DVU3357	46581758	-1.15507	-0.956398
VIMSS208886	DVU3359	46581760	-1.22249	-1.56349
VIMSS208887	DVU3360	46581761	0.159142	0.232119
VIMSS208888	DVU3361	46581762	-0.402236	-0.560568
VIMSS208890	DVU3363	46581764	0.504096	0.774706
VIMSS208891	DVU3364	46581765	0.877142	1.46339
VIMSS208892	DVU3365	46581766	-0.0477611	-0.0890476
VIMSS208893	DVU3366	46581767	-0.551731	-0.819444
VIMSS208894	DVU3367	46581768	-2.39893	-3.23175
VIMSS208895	DVU3368	46581769	-0.785534	-1.04701
VIMSS208896	DVU3369	46581770	0.854531	1.04459
VIMSS208898	DVU3371	46581772	0.146777	0.188215
VIMSS208899	DVU3372	46581773	0.247512	0.29571
VIMSS208900	DVU3373	46581774	-0.585124	-0.871953
VIMSS208901	DVU3374	46581775	0.881022	1.28141
VIMSS208904	DVU3376	46581777	0.0440444	0.0498087
VIMSS208907	DVU3379	46581780	-0.686824	-0.747275
VIMSS208908	DVU3380	46581781	-0.923138	-0.81638
VIMSS208909	DVU3381	46581782	1.11209	1.60581
VIMSS208910	DVU3382	46581783	0.0659279	0.0727486
VIMSS208912	DVU3384	46581785	2.17971	1.57294
VIMSS208914	DVU3386	46581787	-0.665035	-0.961636
VIMSS208915	DVU3387	46581788	1.4032	2.03444
VIMSS208916	DVU3388	46581789	-0.0133238	-0.020706
VIMSS208917	DVU3389	46581790	-1.00715	-1.7759
VIMSS208921	DVU3392	46581793	0.878811	1.40001
VIMSS208922	DVU3393	46581794	0.250382	0.398088
VIMSS208923	DVU3394	46581795	-0.384393	-0.348979
VIMSS208924	DVU3395	46581796	-1.12275	-1.91489
VIMSS208926		46578418	0.182993	0.302721
VIMSS208928	DVU0002	46578419	-0.544314	-0.806788
VIMSS208929	DVU0003	46578420	-0.669291	-1.21849
VIMSS208931	DVU0004	46578421	-0.754002	-1.15672
VIMSS208932	DVU0005	46578422	0.0907492	0.128303
VIMSS208933	DVU0006	46578423	1.43292	2.46024
VIMSS208934	DVU0007	46578424	-0.421515	-0.663131
			-	-
VIMSS208935	DVU0008	46578425	0.00431263	0.00470764
VIMSS208936	DVU0009	46578426	-0.715488	-0.793272
VIMSS208937	DVU0010	46578427	0.262201	0.285477
VIMSS208938	DVU0011	46578428	0.359179	0.597703
VIMSS208939	DVU0012	46578429	0.429008	0.771101

VIMSS208940	DVU0013	46578430	-0.116753	-0.164961
VIMSS208941	DVU0014	46578431	-3.04106	-4.23302
VIMSS208942	DVU0015	46578432	0.146251	0.219363
VIMSS208943	DVU0016	46578433	0.598283	0.796928
VIMSS208945	DVU0018	46578435	0.841005	1.36466
VIMSS208946	DVU0019	46578436	1.67943	2.54203
VIMSS208948	DVU0020	46578437	0.31533	0.471291
VIMSS208949			0.673696	1.10704
VIMSS208950	DVU0022	46578439	0.318509	0.428497
VIMSS208952	DVU0024	46578441	-1.43641	-1.66123
VIMSS208953	DVU0025	46578442	0.554055	0.704223
VIMSS208954	DVU0026	46578443	-0.335297	-0.534539
VIMSS208955	DVU0027	46578444	0.785267	1.25219
VIMSS208956	DVU0028	46578445	1.10217	1.66075
VIMSS208957	DVU0029	46578446	-0.366168	-0.604898
VIMSS208959			0.32801	0.483655
VIMSS208960	DVU0032	46578449	0.779505	1.10236
VIMSS208961	DVU0033	46578450	0.464469	0.846234
VIMSS208962	DVU0034	46578451	0.256306	0.436176
VIMSS208963	DVU0035	46578452	-0.0837912	-0.120696
VIMSS208964	DVU0036	46578453	0.0339445	0.0352102
VIMSS208965	DVU0037	46578454	0.457298	0.766258
VIMSS208966	DVU0038	46578455	-0.29492	-0.452794
VIMSS208967	DVU0039	46578456	-0.365632	-0.4151
VIMSS208968	DVU0040	46578457	-0.81374	-1.11197
VIMSS208969	DVU0041	46578458	-0.0428265	-0.0598896
VIMSS208970			0.24859	0.375881
VIMSS208971	DVU0043	46578460	0.0848999	0.142568
VIMSS208972	DVU0044	46578461	0.0258918	0.0376711
VIMSS208973	DVU0045	46578462	0.455121	0.817796
VIMSS208974	DVU0046	46578463	0.856125	1.59101
VIMSS208975	DVU0047	46578464	0.615184	1.02096
VIMSS208976	DVU0048	46578465	0.524637	0.753252
VIMSS208977	DVU0049	46578466	1.33868	1.99588
VIMSS208978	DVU0050	46578467	0.285364	0.410711
VIMSS208979			0.418345	0.611026
VIMSS208980	DVU0052	46578469	-0.45036	-0.680701
VIMSS208981			1.52294	2.12646
VIMSS208982	DVU0054	46578471	-0.0305732	-0.0438901
VIMSS208983	DVU0055	46578472	-0.184492	-0.234913
VIMSS208984	DVU0056	46578473	0.908005	1.43062
VIMSS208985	DVU0057	46578474	-0.70135	-1.1448
VIMSS208986	DVU0058	46578475	0.527378	0.87065
VIMSS208987	DVU0059	46578476	-0.539776	-0.669462
VIMSS208988	DVU0060	46578477	0.163859	0.257722
VIMSS208989	DVU0061	46578478	-0.320049	-0.577048
VIMSS208990	DVU0062	46578479	0.158363	0.250717
VIMSS208991	DVU0063	46578480	-0.0800524	-0.138908
VIMSS208992	DVU0064	46578481	-0.465994	-0.688574

VIMSS208993	DVU0065	46578482	0.924204	1.16458
VIMSS208994	DVU0066	46578483	0.22093	0.357832
VIMSS208995	DVU0067	46578484	-0.26494	-0.415521
VIMSS208996	DVU0068	46578485	1.17435	1.86004
VIMSS208997	DVU0069	46578486	1.05894	1.73356
VIMSS208998	DVU0070	46578487	0.688263	0.617204
VIMSS208999	DVU0071	46578488	0.483229	0.663668
VIMSS209000	DVU0072	46578489	0.135928	0.208156
VIMSS209001	DVU0073	46578490	-0.228832	-0.384097
VIMSS209002	DVU0074	46578491	0.908626	1.61523
VIMSS209003	DVU0075	46578492	0.505197	0.764789
VIMSS209004	DVU0076	304569677	0.368918	0.65774
VIMSS209005	DVU0077	46578494	1.17951	1.70601
VIMSS209006	DVU0078	46578495	0.482944	0.583444
VIMSS209007	DVU0079	46578496	1.05129	1.59314
VIMSS209008	DVU0080	46578497	0.492382	0.834198
VIMSS209009	DVU0081	46578498	1.5435	2.16243
VIMSS209010	DVU0082	46578499	0.434615	0.439286
VIMSS209011	DVU0083	46578500	-0.559538	-0.8536
VIMSS209012	DVU0084	46578501	-0.174719	-0.208704
VIMSS209013	DVU0085	46578502	-0.565333	-0.901575
VIMSS209016	DVU0087	46578504	-0.536531	-0.750296
VIMSS209017	DVU0088	46578505	-2.91732	-2.91599
VIMSS209018	DVU0089	46578506	-0.394807	-0.371973
VIMSS209019	DVU0090	46578507	-0.629257	-0.804561
VIMSS209020	DVU0091	46578508	0.0482186	0.0816245
VIMSS209021	DVU0092	46578509	-0.393303	-0.59793
VIMSS209022	DVU0093	46578510	-0.598342	-0.820148
VIMSS209023	DVU0094	46578511	-1.04715	-1.48105
VIMSS209024	DVU0095	46578512	-0.60851	-0.950074
VIMSS209025	DVU0096	46578513	-0.386244	-0.390915
VIMSS209026	DVU0097	46578514	-0.119778	-0.170961
VIMSS209027	DVU0098	46578515	0.169412	0.242901
VIMSS209028	DVU0099	46578516	-1.38129	-1.7491
VIMSS209029	DVU0100	46578517	-0.361103	-0.333362
VIMSS209030	DVU0101	46578518	-0.226897	-0.312044
VIMSS209031	DVU0102	46578519	0.463585	0.610795
VIMSS209032	DVU0103	46578520	-2.31724	-2.54701
VIMSS209033	DVU0104	46578521	-1.15545	-1.33234
VIMSS209035	DVU0105	46578522	0.178076	0.269382
VIMSS209036	DVU0106	46578523	0.246227	0.398249
VIMSS209037	DVU0107	46578524	0.117983	0.219454
VIMSS209038	DVU0108	46578525	1.69543	2.85187
VIMSS209039			-0.469893	-0.730959
VIMSS209040	DVU0110	46578527	-0.11193	-0.162343
VIMSS209041	DVU0111	46578528	-0.497579	-0.547291
VIMSS209042	DVU0112	46578529	-0.260072	-0.357667
VIMSS209043	DVU0113	46578530	-1.17446	-1.96534
VIMSS209044	DVU0114	46578531	-1.81562	-2.30011

VIMSS209045	DVU0115	46578532	-0.603932	-0.795936
VIMSS209046	DVU0116	46578533	-0.912287	-1.24877
VIMSS209047	DVU0117	46578534	-0.226989	-0.271982
VIMSS209048	DVU0118	46578535	1.0998	1.48492
VIMSS209049	DVU0119	46578536	0.453622	0.74225
VIMSS209050	DVU0120	46578537	0.933806	1.21256
VIMSS209051	DVU0121	46578538	1.06066	1.78145
VIMSS209052	DVU0122	46578539	0.489058	0.674733
VIMSS209053	DVU0123	46578540	-0.450735	-0.547203
VIMSS209054	DVU0124	46578541	-4.04217	-4.81348
VIMSS209055	DVU0125	46578542	0.97418	1.44052
VIMSS209056	DVU0126	46578543	-1.78493	-2.01832
VIMSS209057	DVU0127	46578544	0.451039	0.663054
VIMSS209058	DVU0128	46578545	0.852954	1.22187
VIMSS209059	DVU0129	46578546	0.214842	0.319814
VIMSS209060	DVU0130	46578547	0.785244	1.13074
VIMSS209063	DVU0132	46578549	-0.260018	-0.394837
VIMSS209065	DVU0133	46578550	-0.792102	-1.22362
VIMSS209066	DVU0134	46578551	-1.12773	-1.76568
VIMSS209067	DVU0136	46578553	-1.2026	-1.55094
VIMSS209068	DVU0135	46578552	-0.713926	-1.23012
VIMSS209070	DVU0138	46578555	2.32329	4.2551
VIMSS209071	DVU0139	46578556	-0.653832	-0.812902
VIMSS209072	DVU0140	46578557	0.782029	1.17343
VIMSS209073	DVU0141	46578558	-0.768004	-1.27941
VIMSS209074	DVU0142	46578559	-0.727455	-1.15046
VIMSS209075	DVU0143	46578560	-0.038122	-0.0578928
VIMSS209076	DVU0144	46578561	0.41414	0.587766
VIMSS209077	DVU0145	46578562	0.956483	1.6184
VIMSS209078	DVU0146	46578563	-0.80756	-1.24781
VIMSS209079	DVU0147	46578564	-0.108618	-0.142069
VIMSS209080	DVU0148	46578565	0.599576	1.0185
VIMSS209081	DVU0149	46578566	1.15125	1.71302
VIMSS209082	DVU0150	46578567	-0.55163	-0.802209
VIMSS209084	DVU0152	46578569	0.582843	1.00367
VIMSS209085	DVU0153	46578570	0.141371	0.239025
VIMSS209087	DVU0155	46578572	0.654252	0.995661
VIMSS209088	DVU0156	46578573	1.46731	2.45462
VIMSS209089	DVU0157	46578574	0.198676	0.343473
VIMSS209090	DVU0158	46578575	-0.221384	-0.263531
VIMSS209091	DVU0159	46578576	-0.790295	-1.08432
VIMSS209092	DVU0160	46578577	-0.0258381	-0.0458344
VIMSS209093	DVU0161	46578578	-1.25985	-1.6421
VIMSS209094	DVU0162	46578579	-1.38319	-2.25742
VIMSS209096	DVU0163	46578580	-1.36813	-1.59544
VIMSS209097	DVU0164	46578581	0.971413	1.38797
VIMSS209098	DVU0165	46578582	-1.68226	-2.14411
VIMSS209099	DVU0166	46578583	-1.27621	-2.0284
VIMSS209100	DVU0167	46578584	-1.18535	-1.79674

VIMSS209101	DVU0168	46578585	-0.98815	-1.62512
VIMSS209102	DVU0169	46578586	0.964086	1.04784
VIMSS209103	DVU0170	46578587	2.02252	2.57891
VIMSS209105	DVU0172	46578589	2.79087	5.24064
VIMSS209106			1.63933	3.11831
VIMSS209107	DVU0174	46578591	1.23041	1.86605
VIMSS209108	DVU0175	46578592	0.213742	0.34573
VIMSS209109	DVU0176	46578593	0.798928	1.3448
VIMSS209110	DVU0177	46578594	0.657028	0.952095
VIMSS209112	DVU0179	304569678	0.463304	0.770709
VIMSS209113	DVU0180	46578597	0.190567	0.216325
VIMSS209114	DVU0181	46578598	0.673154	0.804838
VIMSS209115	DVU0182	46578599	-0.117848	-0.191821
VIMSS209116	DVU0183	46578600	-0.508006	-0.538443
VIMSS209117	DVU0184	46578601	-1.84223	-1.96754
VIMSS209118	DVU0185	46578602	-0.0973711	-0.131227
VIMSS209119	DVU0186	46578603	1.68455	2.42052
VIMSS209120	DVU0187	46578604	0.0306443	0.0447469
VIMSS209122	DVU0189	46578606	-0.263852	-0.360572
VIMSS209123	DVU0190	46578607	-1.06797	-1.05692
VIMSS209124	DVU0191	46578608	0.475254	0.661186
VIMSS209125	DVU0192	46578609	0.099672	0.111465
VIMSS209126	DVU0193	46578610	0.183461	0.0931278
VIMSS209127	DVU0194	46578611	-0.876623	-1.11917
VIMSS209128	DVU0195	46578612	-0.210021	-0.254677
VIMSS209129			-0.446968	-0.699587
VIMSS209130	DVU0197	46578614	-0.267617	-0.396509
VIMSS209131	DVU0198	46578615	-1.06931	-1.28148
VIMSS209132	DVU0199	46578616	-0.877044	-1.43153
VIMSS209133	DVU0200	46578617	-1.15667	-2.0051
VIMSS209134	DVU0201	46578618	-0.71416	-1.18235
VIMSS209135	DVU0202	46578619	-0.987337	-1.35975
VIMSS209136	DVU0203	46578620	-1.22773	-1.74147
VIMSS209137	DVU0204	46578621	-0.474731	-0.675539
VIMSS209138	DVU0205	46578622	-0.20992	-0.268723
VIMSS209139	DVU0206	46578623	-0.25507	-0.371147
VIMSS209140	DVU0207	46578624	-0.237313	-0.330145
VIMSS209141	DVU0208	46578625	-0.806108	-1.15911
VIMSS209142	DVU0209	46578626	-0.207378	-0.334639
VIMSS209143	DVU0210	46578627	-0.959611	-1.22429
VIMSS209144	DVU0211	46578628	0.833114	1.47911
VIMSS209145	DVU0212	46578629	-0.165796	-0.255644
VIMSS209146	DVU0213	46578630	-1.26771	-1.81053
VIMSS209147	DVU0214	46578631	1.45909	1.94762
VIMSS209148	DVU0215	46578632	0.904225	1.28122
VIMSS209149	DVU0216	46578633	0.0196528	0.0248622
VIMSS209150	DVU0217	46578634	-0.421658	-0.578917
VIMSS209151	DVU0218	46578635	0.808185	1.30012
VIMSS209152	DVU0219	46578636	-0.449138	-0.697086

VIMSS209153	DVU0220	46578637	-0.30916	-0.433594
VIMSS209154	DVU0221	46578638	-0.970941	-1.38424
VIMSS209155	DVU0222	46578639	0.32138	0.443667
VIMSS209156	DVU0223	46578640	-0.878163	-1.58896
VIMSS209157	DVU0224	46578641	0.0401522	0.0654575
VIMSS209159	DVU0226	46578643	0.142447	0.229758
VIMSS209160	DVU0227	46578644	-0.654971	-0.892852
VIMSS209163	DVU0230	46578647	0.463009	0.619958
VIMSS209164	DVU0231	46578648	0.564509	0.846092
VIMSS209165	DVU0232	46578649	-1.39252	-1.66588
VIMSS209167	DVU0234	46578651	0.170263	0.245497
VIMSS209168	DVU0235	46578652	-0.189895	-0.284372
VIMSS209169	DVU0236	46578653	-0.44264	-0.473551
VIMSS209170	DVU0237	46578654	-0.491587	-0.739728
VIMSS209171	DVU0238	46578655	0.38997	0.669565
VIMSS209173			1.70614	2.25918
VIMSS209174	DVU0241	46578658	0.157059	0.266711
VIMSS209175	DVU0242	46578659	1.25448	1.8882
VIMSS209176	DVU0243	46578660	0.543686	0.799034
VIMSS209177	DVU0244	46578661	-0.103918	-0.174637
VIMSS209178	DVU0245	46578662	0.577804	0.723044
VIMSS209179	DVU0246	46578663	1.1097	1.4324
VIMSS209180	DVU0247	46578664	-0.268717	-0.447864
VIMSS209182	DVU0249	46578665	-0.378528	-0.490429
VIMSS209183	DVU0250	46578666	1.39158	2.52439
VIMSS209185	DVU0252	46578668	-0.426368	-0.779705
VIMSS209186	DVU0253	46578669	1.04217	1.65708
VIMSS209188	DVU0255	46578671	0.173829	0.297845
VIMSS209189	DVU0256	46578672	0.0831585	0.134377
VIMSS209191	DVU0257	46578673	-0.970536	-1.17204
VIMSS209192	DVU0258	46578674	-0.206239	-0.253936
VIMSS209193	DVU0259	46578675	0.451586	0.79571
VIMSS209194	DVU0260	46578676	0.332169	0.484606
VIMSS209195	DVU0261	46578677	1.11578	1.65414
VIMSS209196	DVU0262	46578678	0.215014	0.264387
VIMSS209197	DVU0263	46578679	-0.149931	-0.238161
VIMSS209198	DVU0264	46578680	0.197641	0.317675
VIMSS209199	DVU0265	46578681	0.179575	0.263327
VIMSS209200	DVU0266	304569679	1.22128	1.81762
VIMSS209203	DVU0269	46578685	-0.621846	-1.03045
VIMSS209204	DVU0270	46578686	0.471311	0.750653
VIMSS209205	DVU0271	46578687	0.525039	0.933429
VIMSS209206	DVU0272	46578688	0.606629	0.748899
VIMSS209207	DVU0273	46578689	-0.124125	-0.121059
VIMSS209208	DVU0274	46578690	-1.05286	-1.59757
VIMSS209209	DVU0275	46578691	1.48987	2.51274
VIMSS209210	DVU0276	46578692	2.55017	3.94891
VIMSS209211	DVU0277	46578693	-0.058431	-0.108042
VIMSS209212	DVU0278	46578694	0.689761	0.936168

VIMSS209213	DVU0279	46578695	-2.03795	-3.12032
VIMSS209214	DVU0280	46578696	-0.0866738	-0.140379
VIMSS209215			0.813143	1.44194
VIMSS209216	DVU0282	46578698	-0.565565	-0.996264
VIMSS209217	DVU0284	304569680	-1.08069	-1.81491
VIMSS209218			-0.073875	-0.0749713
VIMSS209219	DVU0285	46578701	-1.08979	-1.78941
VIMSS209220	DVU0286	46578702	0.108811	0.174486
VIMSS209223	DVU0289		-0.128862	-0.213656
VIMSS209224	DVU0290	46578706	0.569822	0.891006
VIMSS209225	DVU0291	46578707	0.323689	0.460343
VIMSS209226	DVU0293	46578709	-0.810056	-1.2609
VIMSS209227	DVU0292	46578708	-0.508547	-0.781325
VIMSS209228			0.850675	1.25356
VIMSS209229	DVU0295	46578711	0.491257	0.832317
VIMSS209230	DVU0296	46578712	-0.518389	-0.786775
VIMSS209231			-0.469408	-0.654842
VIMSS209232	DVU0298	46578714	0.727045	1.04708
VIMSS209233	DVU0299	46578715	0.25319	0.401106
VIMSS209234	DVU0300	46578716	0.380097	0.504345
VIMSS209236	DVU0302	46578718	0.0708721	0.121084
VIMSS209237	DVU0303	46578719	-0.531555	-0.346686
VIMSS209238	DVU0304	46578720	-1.78438	-2.10988
VIMSS209239	DVU0305	46578721	1.59392	2.33318
VIMSS209240	DVU0306	46578722	-0.0512419	-0.0938025
VIMSS209241	DVU0307	46578723	-0.289785	-0.337852
VIMSS209242	DVU0308	46578724	-2.00652	-2.90508
VIMSS209243	DVU0309	46578725	-0.88921	-0.93679
VIMSS209244	DVU0310	46578726	0.03031	0.0422655
VIMSS209245	DVU0311	46578727	0.65052	1.0753
VIMSS209246	DVU0312	46578728	0.0746287	0.11627
VIMSS209247	DVU0313	46578729	0.203382	0.325762
VIMSS209248	DVU0314	46578730	0.475141	0.76338
VIMSS209249	DVU0315	46578731	0.20761	0.262505
VIMSS209250	DVU0316	46578732	0.435314	0.650165
VIMSS209252	DVU0318	46578734	-0.977981	-1.46264
VIMSS209253	DVU0319	46578735	-1.30693	-1.72147
VIMSS209254	DVU0320	46578736	0.487938	0.476568
VIMSS209255	DVU0321	46578737	0.457546	0.797053
VIMSS209256	DVU0322	46578738	-0.661525	-0.820384
VIMSS209257	DVU0323	46578739	-1.33055	-1.64615
VIMSS209259	DVU0325	46578741	-0.269883	-0.324746
VIMSS209260	DVU0326	46578742	0.00894479	0.0134704
VIMSS209261	DVU0327	46578743	0.495621	0.788806
VIMSS209262	DVU0328	46578744	0.390937	0.711402
VIMSS209264	DVU0330	46578746	1.21578	1.48446
VIMSS209265	DVU0331	46578747	0.825661	0.929997
VIMSS209267	DVU0333	46578749	-0.246655	-0.413575
VIMSS209268	DVU0334	46578750	-0.305569	-0.442068

VIMSS209269	DVU0335	46578751	-1.05301	-1.32145
VIMSS209270	DVU0336	46578752	-0.624087	-0.950997
VIMSS209271	DVU0337	46578753	-0.0562908	-0.0802194
VIMSS209272	DVU0339	46578755	-0.543493	-0.977972
VIMSS209273	DVU0338	46578754	-0.46564	-0.780951
VIMSS209274	DVU0340	46578756	0.0963538	0.15854
VIMSS209275	DVU0341	46578757	-0.247323	-0.38155
VIMSS209276	DVU0342	46578758	0.0629097	0.102624
VIMSS209277	DVU0343	46578759	0.254877	0.35293
VIMSS209278			0.800598	1.20382
VIMSS209280	DVU0346	46578762	-0.729082	-0.783327
VIMSS209281	DVU0347	46578763	0.328875	0.448158
VIMSS209282	DVU0348	46578764	-0.598625	-0.945809
VIMSS209283	DVU0349	46578765	-0.629007	-0.759506
VIMSS209284	DVU0350	46578766	-0.465779	-0.622471
VIMSS209285	DVU0351	46578767	0.575561	0.781101
VIMSS209286	DVU0352	46578768	-0.545796	-0.893883
VIMSS209287	DVU0353	46578769	-0.123029	-0.189003
VIMSS209288			1.33715	2.16479
VIMSS209289	DVU0355	46578771	1.03095	1.80323
VIMSS209290	DVU0356	46578772	0.283996	0.464136
VIMSS209291	DVU0357	46578773	0.346539	0.496518
VIMSS209292	DVU0358	46578774	0.135205	0.166679
VIMSS209293	DVU0359	46578775	-0.691493	-0.837867
VIMSS209294	DVU0360	46578776	1.54251	2.09197
VIMSS209295	DVU0361	46578777	1.59416	2.31172
VIMSS209296	DVU0362	46578778	-0.910199	-1.03285
VIMSS209297			-0.743303	-0.58622
VIMSS209298	DVU0364	46578780	0.48101	0.864996
VIMSS209299	DVU0365	46578781	1.32839	1.53512
VIMSS209301	DVU0367	46578783	0.138855	0.171768
VIMSS209302	DVU0368	46578784	-1.88272	-2.29423
VIMSS209304	DVU0369	46578785	0.0805803	0.0641183
VIMSS209305	DVU0370	46578786	-1.12617	-1.20742
VIMSS209306	DVU0371	46578787	-1.92753	-2.61654
VIMSS209307	DVU0372	46578788	-2.61993	-2.65149
VIMSS209308	DVU0373	46578789	0.217243	0.283701
VIMSS209309	DVU0374	46578790	-0.504646	-0.633005
VIMSS209310	DVU0375	46578791	1.47167	2.46977
VIMSS209311	DVU0376	46578792	0.0670453	0.0745031
VIMSS209312	DVU0377	46578793	0.204032	0.291041
VIMSS209313	DVU0378	46578794	-1.89303	-1.79656
VIMSS209314	DVU0379	46578795	-0.126091	-0.203227
VIMSS209315	DVU0380	46578796	0.195955	0.257708
VIMSS209317	DVU0381	46578797	-2.52639	-1.70582
VIMSS209318	DVU0382	46578798	1.23679	0.599939
VIMSS209319	DVU0383	46578799	0.540322	0.769722
VIMSS209320	DVU0384	46578800	-1.14864	-1.7128
VIMSS209322	DVU0386	46578802	-0.192128	-0.262063

VIMSS209323	DVU0387	46578803	-0.717996	-0.626881
VIMSS209324	DVU0388	46578804	-1.29088	-2.0671
VIMSS209325	DVU0389	46578805	-1.06446	-1.57339
VIMSS209326	DVU0390	46578806	-0.837452	-1.23658
VIMSS209327	DVU0391	46578807	-0.0155477	-0.0188716
VIMSS209328	DVU0392	46578808	0.0309483	0.0438058
VIMSS209329			-0.432172	-0.578527
VIMSS209330	DVU0394	46578810	-1.28135	-1.70063
VIMSS209331	DVU0395	46578811	0.208404	0.2877
VIMSS209332	DVU0396	46578812	-0.726853	-1.25529
VIMSS209333	DVU0397	46578813	-0.618848	-0.809555
VIMSS209334	DVU0398	46578814	-1.24096	-1.92857
VIMSS209335	DVU0399	46578815	-2.12374	-2.71001
VIMSS209336	DVU0400	46578816	-1.49285	-1.62316
VIMSS209338	DVU0402	46578818	-0.100639	-0.106776
VIMSS209339	DVU0403	46578819	-0.576846	-0.663828
VIMSS209340	DVU0404	46578820	-1.07941	-1.32261
VIMSS209341	DVU0405	46578821	-0.809617	-1.00963
VIMSS209342	DVU0406	46578822	-1.53101	-1.92488
VIMSS209344	DVU0408	46578824	0.266703	0.49426
VIMSS209345	DVU0409	46578825	-0.283701	-0.363604
VIMSS209346	DVU0410	46578826	-0.778556	-1.14099
VIMSS209347	DVU0411	46578827	2.18022	3.13972
VIMSS209348	DVU0412	46578828	0.305094	0.518927
VIMSS209349	DVU0413	46578829	1.10186	1.75847
VIMSS209350	DVU0414	46578830	-1.22111	-1.71274
VIMSS209351	DVU0415	46578831	-1.33428	-1.8924
VIMSS209352	DVU0416	46578832	0.895597	1.38504
VIMSS209353	DVU0417	46578833	-1.0087	-1.71689
VIMSS209354	DVU0418	46578834	-0.16895	-0.267995
VIMSS209355	DVU0419	46578835	2.06105	3.29159
VIMSS209356	DVU0420	46578836	0.659247	0.690537
VIMSS209358	DVU0422	46578838	0.498281	0.506578
VIMSS209359	DVU0423	46578839	2.86358	4.95298
VIMSS209360	DVU0424	46578840	-0.0412713	-0.0557086
VIMSS209361	DVU0425	46578841	0.805623	0.971775
VIMSS209364	DVU0428	46578844	-0.623307	-0.990041
VIMSS209365	DVU0429	46578845	1.77725	2.54013
VIMSS209367	DVU0431	46578847	2.44361	2.62286
VIMSS209368	DVU0432	46578848	3.15518	4.27305
VIMSS209369	DVU0433	46578849	2.79888	3.3626
VIMSS209370	DVU0434	46578850	1.90586	2.98123
VIMSS209372			1.17443	1.45736
VIMSS209373	DVU0437	46578853	0.770793	1.17008
VIMSS209374	DVU0438	46578854	0.595763	0.973753
VIMSS209375	DVU0439	46578855	-0.115872	-0.171012
VIMSS209376	DVU0440	46578856	0.270995	0.317375
VIMSS209377	DVU0441	46578857	1.29965	2.02347
VIMSS209378	DVU0442	46578858	0.57976	0.61468

VIMSS209379	DVU0443	46578859	2.88303	4.59608
VIMSS209380	DVU0444	46578860	1.1978	1.57731
VIMSS209381	DVU0445	46578861	-0.0526134	-0.0673782
VIMSS209382	DVU0446	46578862	-0.497008	-0.461386
VIMSS209383	DVU0447	46578863	0.109206	0.118518
VIMSS209384	DVU0448	46578864	0.617961	1.1345
VIMSS209386	DVU0450	46578866	0.266311	0.442154
VIMSS209387	DVU0451	46578867	0.293559	0.418348
VIMSS209388			-0.528452	-0.767219
VIMSS209389	DVU0453	304569684	1.33874	2.0234
VIMSS209390	DVU0454	46578870	0.389397	0.686996
VIMSS209391	DVU0455	46578871	0.84562	1.23445
VIMSS209392	DVU0456	46578872	0.559475	0.931603
VIMSS209393	DVU0457	46578873	-0.0086564	-0.0115253
VIMSS209394	DVU0458	46578874	-1.33099	-1.27185
VIMSS209395	DVU0459	46578875	-2.17968	-2.94289
VIMSS209396	DVU0460	46578876	-1.17713	-1.87599
VIMSS209397	DVU0461	46578877	-1.31441	-2.26739
VIMSS209398	DVU0462	46578878	-0.713342	-1.14153
VIMSS209399	DVU0463	46578879	-0.0627859	-0.112138
VIMSS209400	DVU0464	46578880	-0.586938	-0.923396
VIMSS209401	DVU0465	46578881	-1.16153	-1.53877
VIMSS209402	DVU0466	46578882	-0.9568	-1.24173
VIMSS209403	DVU0467	46578883	-0.011699	-0.0156225
VIMSS209404	DVU0468	46578884	-1.00078	-1.46329
VIMSS209405	DVU0469	46578885	-0.95664	-0.948975
VIMSS209406	DVU0470	46578886	-1.54854	-2.37529
VIMSS209407	DVU0471	46578887	-0.846149	-1.34717
VIMSS209411	DVU0475	46578891	-0.903374	-1.19873
VIMSS209413	DVU0477	46578893	-0.360247	-0.532062
VIMSS209414	DVU0478	46578894	1.24535	1.57297
VIMSS209415	DVU0479	46578895	0.416855	0.48864
VIMSS209416	DVU0480	46578896	-0.0802579	-0.128729
VIMSS209417	DVU0481	46578897	-0.205887	-0.376566
VIMSS209418	DVU0482	46578898	2.27439	3.73699
VIMSS209420	DVU0484	46578900	1.5236	1.95989
VIMSS209421	DVU0485	46578901	-0.403024	-0.632544
VIMSS209423	DVU0487	46578903	-0.137802	-0.215806
VIMSS209425	DVU0489	46578905	-0.0230254	-0.0383345
VIMSS209428	DVU0491	46578906	0.641198	0.925172
VIMSS209429	DVU0492	46578907	0.505479	0.795748
VIMSS209430	DVU0493	46578908	-0.126376	-0.189846
VIMSS209431	DVU0494	46578909	-0.192898	-0.269505
VIMSS209432	DVU0495	46578910	0.120296	0.197379
VIMSS209433	DVU0496	46578911	0.597985	0.934831
VIMSS209434	DVU0497	46578912	1.06184	0.998703
VIMSS209435	DVU0498	46578913	0.612168	0.89918
VIMSS209436	DVU0499	46578914	0.530134	0.711066
VIMSS209437	DVU0500	46578915	0.375545	0.59441

VIMSS209438	DVU0501	46578916	-1.3567	-2.22618
VIMSS209439			-0.920672	-1.3415
VIMSS209440	DVU0503	46578918	-1.76274	-1.77477
VIMSS209441	DVU0504	46578919	-2.5202	-3.53144
VIMSS209442	DVU0505	46578920	0.489753	0.75829
VIMSS209443	DVU0506	46578921	-0.341303	-0.516686
VIMSS209445	DVU0507	46578922	-1.04761	-1.1579
VIMSS209446	DVU0508	46578923	-1.09852	-1.34019
VIMSS209448	DVU0510	46578925	-1.55983	-1.76809
VIMSS209449	DVU0511	46578926	-1.17334	-1.9746
VIMSS209450	DVU0512	46578927	-0.431769	-0.60809
VIMSS209451	DVU0513	46578928	0.0018208	0.00313372
VIMSS209452	DVU0514	46578929	0.0751847	0.112959
VIMSS209453	DVU0515	46578930	1.27002	2.19135
VIMSS209455	DVU0517	46578932	1.17785	1.74778
VIMSS209456	DVU0518	46578933	-0.522191	-0.969487
VIMSS209457	DVU0519	46578934	-0.662674	-1.08573
VIMSS209458	DVU0520	46578935	-0.368876	-0.430248
VIMSS209459	DVU0521	46578936	0.451311	0.642412
VIMSS209461	DVU0522	46578937	-0.217666	-0.311325
VIMSS209463	DVU0524	46578939	1.17684	2.15441
VIMSS209465	DVU0526	46578941	0.546098	0.664339
VIMSS209466	DVU0527	46578942	1.17715	1.62192
VIMSS209467	DVU0528	46578943	0.855846	0.951804
VIMSS209468	DVU0529	46578944	0.0686882	0.0928487
VIMSS209469	DVU0530	46578945	-0.480248	-0.560664
VIMSS209470	DVU0531	46578946	0.483665	0.638655
VIMSS209471	DVU0532	46578947	0.0691403	0.0786479
VIMSS209472	DVU0533	46578948	-0.0886638	-0.107591
VIMSS209473	DVU0534	46578949	-1.85246	-1.61747
VIMSS209475	DVU0536	46578951	-0.396062	-0.504854
VIMSS209477	DVU0538	46578953	0.168136	0.215696
VIMSS209478	DVU0539	46578954	0.144691	0.207011
VIMSS209479	DVU0540	46578955	0.756342	1.07806
VIMSS209481	DVU0542	46578957	-0.171897	-0.219655
VIMSS209482	DVU0543	46578958	1.00609	1.01186
VIMSS209483	DVU0544	46578959	-0.755318	-0.9011
VIMSS209484	DVU0545	46578960	0.738299	1.01105
VIMSS209486	DVU0547	46578962	-0.368168	-0.545808
VIMSS209487	DVU0548	46578963	-1.17532	-1.58386
VIMSS209488	DVU0549	46578964	-1.06503	-1.56343
VIMSS209489	DVU0550	46578965	-0.751495	-1.03842
VIMSS209490	DVU0551	46578966	-0.577659	-0.966907
VIMSS209496	DVU0555	46578970	-2.94414	-3.3658
VIMSS209497	DVU0556	46578971	-1.43686	-2.07252
VIMSS209498	DVU0557		-1.70229	-2.88618
VIMSS209502	DVU0561	46578975	-2.10314	-3.24371
VIMSS209503	DVU0562	46578976	-2.82595	-4.92421
VIMSS209504	DVU0563	46578977	-2.32882	-4.02127

VIMSS209506	DVU0565	46578979	2.13584	3.63755
VIMSS209507	DVU0566	46578980	1.28322	2.36093
VIMSS209508	DVU0567	46578981	-0.0782076	-0.112129
VIMSS209510	DVU0568	46578982	0.213681	0.253055
VIMSS209511	DVU0569	46578983	-0.260625	-0.394757
VIMSS209512	DVU0570	46578984	0.323875	0.389592
VIMSS209513	DVU0571	46578985	0.972989	1.51923
VIMSS209514	DVU0572	46578986	0.906854	1.57679
VIMSS209516	DVU0573	46578987	1.33988	2.37548
VIMSS209518	DVU0575	46578989	0.680045	1.07265
VIMSS209519	DVU0576	46578990	2.32675	4.23962
VIMSS209520	DVU0577	46578991	1.52068	2.15748
VIMSS209522	DVU0579	46578993	0.851825	1.18946
VIMSS209523	DVU0580	46578994	2.35159	4.33405
VIMSS209524	DVU0581	46578995	1.31933	2.01895
VIMSS209525	DVU0582	46578996	1.63245	2.95679
VIMSS209527			1.21466	2.1796
VIMSS209528	DVU0585	46578999	-0.898105	-0.971712
VIMSS209529	DVU0586	46579000	4.81649	6.99623
VIMSS209532	DVU0588	46579002	3.25426	4.99522
VIMSS209534			0.139865	0.144659
VIMSS209535	DVU0591	46579005	1.34486	2.49686
VIMSS209536	DVU0592	46579006	1.33598	2.20024
			-	-
VIMSS209537	DVU0593	46579007	0.00591326	0.00640109
VIMSS209538	DVU0594	46579008	1.20023	2.04243
VIMSS209539	DVU0595	46579009	1.56499	2.9848
VIMSS209540	DVU0596	46579010	1.03031	1.64562
VIMSS209541	DVU0597	46579011	0.716246	1.16199
VIMSS209542	DVU0598	46579012	3.48868	3.48879
VIMSS209543	DVU0599	46579013	2.72416	3.67995
VIMSS209544	DVU0600	46579014	0.878375	1.07215
VIMSS209545	DVU0601	46579015	0.799001	1.13699
VIMSS209546	DVU0602	46579016	1.17404	2.17716
VIMSS209547	DVU0603	46579017	1.32562	2.02087
VIMSS209549	DVU0605	46579019	0.501461	0.397251
VIMSS209550	DVU0606	46579020	-0.2314	-0.371515
VIMSS209552	DVU0608	46579022	2.00618	3.33535
VIMSS209555	DVU0611	46579025	0.81797	1.13992
VIMSS209556			2.12393	3.2519
VIMSS209561	DVU0616	46579030	-0.0452346	-0.0633194
VIMSS209562	DVU0617	46579031	0.136729	0.180166
VIMSS209564	DVU0618	46579032	-0.991094	-0.999748
VIMSS209565	DVU0619	46579033	0.346627	0.504639
VIMSS209566	DVU0620	46579034	1.16881	1.77158
VIMSS209567	DVU0621	46579035	0.258788	0.303018
VIMSS209568	DVU0622	46579036	0.0794962	0.12865
VIMSS209570	DVU0624	46579038	0.957427	1.58377
VIMSS209571	DVU0625	46579039	1.61936	2.6137

VIMSS209572	DVU0626	46579040	0.686729	1.22305
VIMSS209573	DVU0627	46579041	0.466841	0.677698
VIMSS209574	DVU0628	46579042	1.42701	2.42069
VIMSS209575	DVU0629	46579043	0.331006	0.462359
VIMSS209576	DVU0630	46579044	0.774912	1.32807
VIMSS209577	DVU0631	46579045	0.77963	1.32761
VIMSS209578	DVU0632	46579046	-0.252131	-0.398949
VIMSS209579	DVU0633	46579047	0.473183	0.726284
VIMSS209580	DVU0634	46579048	1.50762	2.64924
VIMSS209582	DVU0636	46579050	1.53542	2.32269
VIMSS209583	DVU0637	46579051	-0.268111	-0.356849
VIMSS209584	DVU0638	46579052	1.17171	1.99409
VIMSS209585	DVU0639	46579053	0.622861	1.05852
VIMSS209587	DVU0641	46579055	0.595338	0.941918
VIMSS209588	DVU0642	46579056	0.161099	0.270048
VIMSS209589			-0.00770574	-0.0105225
VIMSS209592	DVUA0132	46562130	0.808531	1.52578
VIMSS209594	DVUA0131	46562131	2.84543	4.19403
VIMSS209607	DVUA0116	46562143	0.124789	0.146809
VIMSS209609	DVUA0114	46562145	0.397409	0.460871
VIMSS209612	DVUA0111	46562148	0.0770927	0.103363
VIMSS209624	DVUA0097	46562160	1.92004	2.60679
VIMSS209629	DVUA0091	46562165	3.91191	3.6569
VIMSS209643	DVUA0076	46562176	-0.336367	-0.470852
VIMSS209644	DVUA0075	46562177	-1.84563	-2.39555
VIMSS209646	DVUA0074	46562178	-0.816927	-0.544087
VIMSS209647	DVUA0073	46562179	-0.134253	-0.199113
VIMSS209650	DVUA0070	46562182	0.215179	0.232973
VIMSS209652	DVUA0069	46562183	1.05319	1.43482
VIMSS209686	DVUA0036	46562211	0.690487	0.98728
VIMSS209692	DVUA0030	46562217	3.06491	3.01353
VIMSS209694	DVUA0028	46562219	2.20566	2.35395
VIMSS209700	DVUA0023	46562224	1.48393	1.76625
VIMSS209704	DVUA0020	46562227	-1.12801	-1.09844
VIMSS209705	DVUA0019	46562228	0.854639	0.892537
VIMSS209717	DVUA0006	46562240	0.136286	0.187412
VIMSS209719	DVUA0003	46562242	1.01804	0.953485
VIMSS209727	DVUA0147	46562249	0.26261	0.246496
VIMSS209731	DVUA0141	46562253	2.15499	1.83314
VIMSS209737	DVUA0135	46562258	0.602653	1.0307
VIMSS408305	DVU1112	46579525	0.713655	0.980037
VIMSS408306	DVU1127	46579540	2.22894	2.25566
VIMSS408312	DVU1290	46579701	1.02006	1.48172
VIMSS408317			0.0415176	0.0392431
VIMSS408318			-0.218626	-0.212028
VIMSS408321	DVU1485	46579896	-0.372566	-0.552609
VIMSS408322	DVU1486	46579897	1.11015	1.54663
VIMSS408323	DVU1487	46579898	1.8093	1.97986

VIMSS408326	DVU1495	46579906	1.514	1.60594
VIMSS408327	DVU1496	46579907	2.43616	2.43033
VIMSS408328	DVU1497	46579908	0.705951	0.751227
VIMSS408329	DVU1510	46579921	-0.119161	-0.196473
VIMSS408330	DVU1511	46579922	0.993157	1.13121
VIMSS408331			-0.798825	-1.20034
VIMSS408352	DVU2174	46580579	-2.49893	-4.07739
