# Supplementary material for: Transcriptomic and proteomic analyses of Desulfovibrio vulgaris biofilms: Carbon and energy flow contribute to the distinct biofilm growth state
Source: BMC Genomics. 2012 Apr 16;13:138. doi: 10.1186/1471-2164-13-138 (PMC3431258; doi:10.1186/1471-2164-13-138)
Supplement: Additional file 4 — Whole genome transcript expression data for biofilm cells compared to planktonic, reactor cells. [file 1471-2164-13-138-S4.pdf]

Systematic_Name	LocusID	Log_Ratio Biofilm vs. Reactor Planktonic	Z_Score
DVU0646	206065	-0.815643	-1.4852
DVU0647	206066	-0.339293	-0.499856
DVU0648	206067	-0.878017	-1.29022
DVU0649	206069	-0.356352	-0.607528
DVU0650	206070	-0.605423	-0.902032
DVU0651	206071	-0.850364	-1.35787
DVU0652	206072	0.747784	1.27723
DVU0653	206074	-0.521212	-1.00515
DVU0654	206075	-0.21437	-0.396491
DVU0655	206076	0.0406761	0.0757331
VIMSS206077	206077	-0.0750986	-0.138052
DVU0656	206078	0.00374359	0.00709161
DVU0657	206079	-0.68162	-1.25067
DVU0659	206081	0.391295	0.762787
VIMSS206082	206082	0.18909	0.306542
DVU0660	206083	-0.118134	-0.218163
DVU0661	206084	0.330414	0.59695
DVU0662	206085	0.226507	0.431604
DVU0663	206086	0.967311	1.8331
DVU0664	206087	0.0294189	0.0540912
DVU0665	206088	1.19737	2.18045
DVU0666	206089	-0.0199644	-0.033141
DVU0667	206090	-0.103349	-0.18833
DVU0668	206091	-0.431293	-0.652047
DVU0669	206092	-0.226359	-0.431413
DVU0670	206093	0.63229	0
DVU0671	206094	-1.76641	-3.14671
DVU0672	206095	1.91506	2.12813
DVU0674	206098	0.169817	0.31904
DVU0676	206100	0.621267	1.15128
DVU0677	206101	0.776917	1.43082
VIMSS206102	206102	-0.0288718	-0.0480486
DVU0679	206103	0.00585334	0.00876795
DVU0680	206104	-0.409953	-0.664233
DVU0681	206105	0.355897	0.6129
DVU0682	206106	-0.398573	-0.69868
DVU0683	206107	0.858155	1.61964
DVU0684	206108	0.324922	0.590276
DVU0685	206109	0.531883	0.972099
DVU0686	206110	0.973273	1.63356
DVU0687	206111	0.226682	0.398462
DVU0688	206112	0.135176	0.223756
DVU0689	206113	0.201506	0.364417
DVU0690	206114	-0.226207	-0.434333
DVU0691	206115	0.326493	0.559478
DVU0692	206116	-0.827795	-1.59697
DVU0693	206117	-0.796819	-1.37573
DVU0694	206118	-0.899687	-1.3084
VIMSS206119	206119	-0.838219	-1.17167
VIMSS206120	206120	-0.021033	-0.0363483
DVU0697	206121	0.360628	0.689685
DVU0698	206122	-0.188784	-0.348297
VIMSS206123	206123	-0.341994	-0.652312
DVU0699	206124	-0.330912	-0.60606
DVU0700	206125	0.655068	1.16469
DVU0701	206126	0.634307	1.21106
DVU0702	206127	-0.132072	-0.220817
DVU0703	206128	-0.776221	-1.24827
DVU0704	206129	-0.499602	-0.969887
DVU0706	206130	-0.130018	-0.197469
DVU0705	206131	-0.112797	-0.156004
DVU0707	206132	0.341608	0.656252
VIMSS206133	206133	0.334404	0.489319
VIMSS206134	206134	0.0696518	0.119006
DVU0710	206135	0.522376	0.518665
DVU0711	206136	-0.608922	-1.08729
DVU0712	206137	0.794407	1.30832
DVU0713	206138	-0.716273	-1.32759
DVU0714	206139	-0.434145	-0.801706
DVU0715	206140	-0.412372	-0.690565
DVU0716	206141	-1.66459	-2.90893

DVU0717	206142	0.318955	0.524578
DVU0720	206145	0.950652	1.72088
DVU0721	206146	0.399829	0.696165
DVU0722	206147	0.164665	0.259069
VIMSS206148	206148	-0.916297	-1.48195
DVU0723	206149	-1.06543	-1.81051
DVU0724	206150	-1.70762	-3.07516
DVU0725	206151	1.17929	2.31665
DVU0726	206152	-0.566464	-1.01318
DVU0727	206153	0.382828	0.687776
DVU0728	206154	0.799191	1.34854
DVU0729	206155	2.07207	4.05212
DVU0730	206156	-0.324077	-0.588656
DVU0731	206157	0.477004	0.791412
DVU0732	206158	-1.15264	-1.98032
DVU0733	206159	-0.286542	-0.514758
DVU0734	206160	-0.824422	-1.42075
DVU0735	206161	0.142492	0.252923
DVU0736	206162	-0.127026	-0.221575
DVU0737	206163	-0.00115679	-0.00213216
DVU0738	206164	0.474212	0.892045
DVU0739	206165	0.529251	0.848123
DVU0740	206166	-0.0858672	-0.147809
DVU0741	206167	0.668166	1.29811
DVU0742	206168	0.792122	1.42288
DVU0743	206169	-0.192355	-0.346861
DVU0744	206170	-0.335254	-0.621187
DVU0745	206171	-0.410128	-0.741695
DVU0746	206172	-1.43836	-2.26623
DVU0747	206173	-0.464286	-0.756149
DVU0749	206175	0.0814519	0.152055
DVU0750	206176	0.0418466	0.0661956
DVU0751	206177	0.386693	0.750794
DVU0752	206178	0.00402871	0.00718712
DVU0753	206179	0.0443022	0.0844714
DVU0754	206180	1.00883	1.87218
DVU0755	206181	1.03919	1.85966
DVU0756	206182	0.244151	0.395259
DVU0757	206183	-0.237013	-0.411316
DVU0758	206184	0.304976	0.490835
DVU0759	206185	0.147104	0.252558
DVU0760	206186	-0.231424	-0.424361
DVU0761	206187	-0.0136245	-0.0263837
DVU0762	206188	0.060876	0.11936
DVU0763	206189	0.0518783	0.0956952
DVU0764	206190	-2.15381	-3.86314
DVU0765	206191	-0.432193	-0.803731
VIMSS206192	206192	1.49067	2.47938
DVU0766	206193	0.949009	1.82856
DVU0767	206194	-1.01948	-1.83883
DVU0768	206195	-0.565916	-1.03044
DVU0769	206196	-0.688381	-1.13566
DVU0770	206197	0.382114	0.614883
DVU0771	206198	0.167704	0.268032
DVU0772	206199	0.820398	1.19457
DVU0773	206200	2.05169	3.74908
DVU0774	206201	-0.992573	-1.63033
DVU0775	206202	-0.833528	-1.20988
DVU0776	206203	-0.645054	-0.83247
DVU0777	206204	-1.13269	-1.4052
DVU0778	206205	-0.31641	-0.48609
DVU0779	206206	-0.492227	-0.754511
DVU0780	206207	-1.50457	-2.43569
VIMSS206208	206208	-0.988684	-1.24567
VIMSS206209	206209	-0.883753	-1.266
VIMSS206210	206210	-0.869222	-1.23109
DVU0784	206211	-0.670883	-1.17864
DVU0785	206212	-0.454962	-0.850147
DVU0786	206213	-0.397119	-0.693663
DVU0787	206214	-0.337848	-0.647907
DVU0788	206215	-0.776838	-1.43736
DVU0789	206216	-0.0598656	-0.109225

DVU0790	206217	0.181738	0
DVU0791	206218	0.459802	0.868742
DVU0792	206219	-0.355687	-0.638196
DVU0793	206220	-1.38327	-2.51976
DVU0794	206221	-0.467365	-0.857693
DVU0795	206222	-1.04549	-1.43058
DVU0796	206223	-0.535761	-0.991246
DVU0798	206224	-0.427656	-0.545864
VIMSS206226	206226	0.0341473	0.0473444
DVU0801	206227	0.311801	0.52526
DVU0802	206228	-0.0577485	-0.101839
DVU0803	206229	0.227256	0.417378
DVU0804	206230	-0.07262	-0.107289
DVU0805	206231	0.22252	0.417436
DVU0806	206232	-0.0580421	-0.110225
DVU0807	206233	-0.653702	-1.19349
DVU0808	206234	-0.978549	-1.66462
DVU0809	206235	-0.467881	-0.837003
DVU0810	206236	-0.236951	-0.436318
VIMSS206237	206237	-0.213011	-0.267478
DVU0811	206238	0.452366	0.822348
DVU0812	206239	0.12903	0.230732
DVU0813	206240	0.356389	0.638829
DVU0814	206241	-0.122242	-0.228853
DVU0815	206242	0.29615	0.559459
DVU0816	206243	-0.316578	-0.507882
DVU0817	206244	-0.271689	-0.513158
DVU0818	206245	-0.404202	-0.691871
DVU0819	206246	1.14567	2.05699
VIMSS206247	206247	-0.398641	-0.448539
DVU0821	206248	0.733819	1.24582
DVU0822	206249	0.600095	1.08003
DVU0823	206250	-0.28959	-0.463848
VIMSS206251	206251	0.59048	0.791886
DVU0825	206252	0.0910175	0.170098
DVU0826	206253	-0.440455	-0.704457
DVU0827	206254	-1.36036	-2.62967
DVU0828	206256	-0.00574312	-0.00908451
DVU0829	206257	-0.215244	-0.405224
DVU0830	206258	-0.537921	-1.02618
DVU0831	206259	-0.229616	-0.437018
DVU0832	206260	-0.5321	-1.03089
DVU0834	206261	-0.810832	-1.08743
DVU0835	206262	-0.735994	-1.31231
DVU0836	206263	-1.20755	-2.16747
DVU0837	206264	0.488742	0.933319
DVU0838	206265	0.793012	1.42781
DVU0839	206266	0.151122	0.282529
DVU0840	206267	-0.701535	-1.34707
DVU0841	206268	-0.974301	-1.79735
DVU0842	206269	-0.28111	-0.505329
DVU0843	206270	-0.112522	-0.197588
VIMSS206271	206271	0.140536	0
VIMSS206273	206273	-0.858517	-1.20558
DVU0847	206274	-0.855404	-1.19108
DVU0848	206275	-0.904976	-1.41703
DVU0849	206276	-1.21909	-1.9254
DVU0850	206277	-1.19249	-1.71392
DVU0852	206279	-0.245868	-0.278944
DVU0853	206280	0.705076	1.28708
DVU0854	206281	-0.287783	-0.539115
DVU0855	206282	-0.177053	-0.331441
DVU0856	206283	-0.76483	-1.41264
VIMSS206284	206284	0.434415	0.77617
DVU0857	206285	0.218498	0.403906
DVU0858	206286	0.549865	0.820499
DVU0859	206287	0.190553	0.31475
VIMSS206288	206288	-0.0853759	-0.129448
DVU0861	206289	-0.0224845	-0.0338379
DVU0862	206290	-0.0341508	-0.0552106
DVU0863	206291	0.0728798	0.131508
DVU0864	206292	-0.949691	-1.71113

DVU0865	206293	-0.641723	-1.16217
DVU0866	206294	-0.0495209	-0.0935959
DVU0867	206295	-0.695866	-1.22513
DVU0868	206296	0.00691825	0.0119219
DVU0869	206297	-0.742624	-1.36389
DVU0870	206298	0.420647	0.821621
DVU0871	206299	-0.607484	-1.15535
DVU0872	206300	-0.350979	-0.655154
DVU0873	206301	-1.29004	-2.02344
DVU0874	206302	-1.47371	-2.49877
DVU0875	206303	-0.0271406	-0.0484355
DVU0876	206304	-0.0457078	-0.0862308
VIMSS206305	206305	-0.5244	-0.643197
DVU0878	206306	1.2897	2.34808
VIMSS206307	206307	-0.624262	-1.10739
DVU0880	206308	-0.850443	-1.50706
DVU0881	206309	0.825075	1.50819
DVU0882	206310	0.484096	0.793249
DVU0883	206311	0.789586	1.4981
DVU0884	206312	0.667222	1.12987
DVU0885	206313	-0.929228	-1.75403
DVU0886	206314	-0.755685	-1.31723
DVU0888	206316	0.26939	0.467291
DVU0890	206318	-0.8919	-1.59162
DVU0891	206319	-1.11683	-1.90378
DVU0892	206320	0.599028	1.02851
DVU0893	206321	0.500385	0.974045
DVU0895	206323	-1.05686	-1.95398
DVU0896	206324	-1.01644	-1.88983
DVU0897	206325	0.164787	0.275399
DVU0899	206327	-0.00389033	-0.00758116
DVU0900	206328	-0.334396	-0.600354
DVU0901	206329	-0.352066	-0.485196
DVU0902	206330	-0.0962643	-0.178126
DVU0903	206331	0.165237	0.295648
DVU0904	206332	-0.492608	-0.688259
DVU0905	206333	-0.161541	-0.270261
DVU0906	206334	0.0236068	0.0339055
DVU0907	206335	-0.0804063	-0.148145
DVU0908	206336	-0.38329	-0.687257
DVU0909	206337	0.657458	0
DVU0910	206338	0.228411	0.439294
DVU0911	206339	-0.697423	-1.22817
DVU0912	206340	0.277782	0.528328
DVU0913	206341	0.721255	1.3252
VIMSS206342	206342	0.109838	0.173112
DVU0914	206343	0.110284	0.195323
DVU0915	206344	-0.0242343	-0.0435925
DVU0916	206345	-0.111184	-0.213444
VIMSS206346	206346	-0.0736335	-0.14383
DVU0918	206348	-0.247913	-0.446488
DVU0919	206349	0.209315	0.391743
DVU0920	206350	-0.00590538	-0.00993368
DVU0921	206351	0.0960505	0.119687
DVU0922	206352	-0.172856	-0.303646
DVU0923	206353	-0.320506	-0.481779
DVU0924	206355	-0.774086	-1.3494
DVU0925	206356	-0.449352	-0.778876
DVU0926	206357	-0.226899	-0.359072
DVU0927	206358	-1.26556	-2.25267
DVU0928	206359	-1.00742	-1.81696
DVU0929	206360	-0.111682	-0.190229
DVU0930	206361	-0.250851	-0.42795
DVU0931	206362	-0.545575	-1.0216
DVU0932	206363	-0.555999	-1.02073
DVU0933	206364	-0.270991	-0.456535
DVU0934	206365	-0.0720692	-0.111909
DVU0935	206366	1.14242	1.94608
DVU0936	206367	-0.803848	-1.44273
DVU0937	206368	-0.161459	-0.25168
DVU0938	206369	1.33543	2.42456
DVU0939	206370	1.83106	3.56998

DVU0940	206371	0.486211	0.868672
VIMSS206372	206372	0.471096	0.778503
DVU0941	206373	0.818457	1.5193
DVU0942	206374	0.290348	0.532773
DVU0943	206375	1.29947	1.85512
DVU0944	206376	1.46603	2.39974
DVU0945	206377	0.592544	1.09149
DVU0946	206378	-0.0416895	-0.0803055
DVU0947	206379	-0.818195	-1.3811
DVU0948	206380	0.0681599	0.0966022
DVU0949	206381	-0.249576	-0.351844
VIMSS206382	206382	-1.12394	-1.35701
DVU0951	206383	-0.108968	-0.209072
DVU0952	206384	-0.878793	-1.6079
DVU0953	206385	-0.816945	-1.45663
DVU0954	206386	-0.914004	0
DVU0955	206387	0.890158	1.57258
DVU0956	206388	-1.42045	-2.47868
DVU0957	206389	-0.876082	-1.5555
DVU0958	206390	-1.24758	-2.26137
DVU0959	206391	-0.989818	-1.58642
VIMSS206392	206392	-0.532583	-0.960489
DVU0961	206393	0.76853	1.43477
VIMSS206394	206394	-0.503187	-0.64344
DVU0963	206395	0.832886	1.53177
DVU0966	206398	-1.26784	-2.19941
DVU0967	206399	-1.36597	-2.42725
DVU0968	206400	-1.09463	-1.8524
DVU0969	206401	0.104228	0.186728
DVU0970	206402	0.0398646	0.0672401
DVU0971	206403	0.111033	0.203557
DVU0972	206404	-0.23456	-0.366118
DVU0973	206405	-1.13437	-1.88324
DVU0974	206406	0.314997	0.561252
DVU0975	206407	0.0396178	0.0727337
DVU0976	206408	0.00244318	0.00425403
VIMSS206409	206409	0.234423	0.282973
DVU0978	206410	0.0625967	0.101031
DVU0979	206411	1.11751	2.17235
DVU0980	206412	0.727556	1.35486
DVU0981	206413	0.617978	1.06828
DVU0982	206414	-0.164454	-0.297972
DVU0983	206415	0.0149001	0.0280125
DVU0984	206416	0.149085	0.271679
VIMSS206417	206417	-0.157313	-0.256065
DVU0987	206418	0.933039	1.63739
VIMSS206419	206419	0.688245	1.15488
DVU0988	206420	-0.0477501	-0.0878722
DVU0989	206421	0.0866699	0.164235
DVU0990	206422	-1.07335	-1.94538
DVU0991	206423	-0.785773	-1.39915
DVU0992	206424	0.428975	0.762688
DVU0993	206425	0.227565	0.419491
DVU0994	206426	-0.639783	-1.11112
DVU0995	206427	0.281781	0.534134
DVU0996	206428	0.0633564	0.11117
DVU0997	206429	-0.490982	-0.941392
DVU0998	206430	-0.505489	-0.927975
VIMSS206432	206432	-0.944866	-1.33569
DVU0999	206433	0.584111	0.985764
DVU1000	206434	-0.00343483	-0.00628373
DVU1001	206435	-0.345171	-0.582604
DVU1002	206436	0.122606	0.239862
DVU1003	206437	-0.336966	-0.589984
DVU1004	206438	0.721272	1.35245
DVU1005	206439	0.144866	0.280731
DVU1006	206440	0.179464	0.335855
DVU1007	206441	0.338818	0.589085
DVU1008	206442	-0.587851	-1.08729
DVU1009	206443	-0.73375	-1.33365
VIMSS206444	206444	-0.902988	-1.4825
VIMSS206445	206445	0.0293076	0.0482225

DVU1012	206446	0.877148	1.5816
DVU1013	206447	1.22974	2.35412
VIMSS206448	206448	0.667827	0.702604
VIMSS206449	206449	0.937248	0
VIMSS206450	206450	0.532791	0.847612
DVU1017	206451	-0.107291	-0.193171
DVU1018	206452	0.188575	0.356858
DVU1019	206453	-0.395413	-0.699546
DVU1020	206454	0.38078	0.690749
DVU1021	206455	-1.02208	-1.82775
DVU1022	206456	-0.923138	-1.74859
DVU1024	206458	-0.598169	-1.12796
DVU1025	206459	-0.934822	-1.68905
DVU1026	206460	-0.66374	-1.09294
DVU1027	206461	-0.43494	-0.687443
DVU1028	206462	0.106881	0.197846
DVU1029	206463	-1.58231	-2.92669
VIMSS206464	206464	-0.298944	-0.416514
DVU1030	206465	0.5176	0.95929
DVU1032	206466	-0.209166	-0.23898
DVU1033	206467	0.5167	0.995804
DVU1034	206468	0.327965	0.485339
DVU1035	206469	-0.232022	-0.352728
DVU1036	206470	-0.161641	-0.196711
DVU1037	206471	0.311951	0.612522
DVU1038	206472	-0.846322	-1.57017
DVU1039	206473	-0.931674	-1.75125
DVU1040	206474	-0.496137	-0.920792
DVU1041	206475	-0.708663	-1.31058
DVU1042	206476	-1.29971	-2.12853
DVU1043	206477	-0.725447	-1.32507
DVU1044	206478	0.291648	0.236824
DVU1045	206479	-1.76236	-3.23236
DVU1046	206480	-1.23474	-1.99326
DVU1047	206481	-1.22087	-2.37587
DVU1048	206482	-0.866224	-1.58037
DVU1049	206483	-0.833907	-1.48888
DVU1050	206484	-1.10239	-2.02321
DVU1051	206485	-0.565971	-1.00078
DVU1054	206488	-0.356558	-0.674327
DVU1055	206489	-1.17521	-1.81381
DVU1056	206490	-0.392959	-0.67743
DVU1057	206491	0.0475662	0.0751324
DVU1058	206492	0.908914	1.44042
DVU1060	206494	-1.2074	-2.14267
DVU1061	206495	-0.96525	-1.75103
DVU1062	206496	-1.67468	-3.08236
DVU1063	206497	-0.570901	0
DVU1064	206498	-0.417117	-0.756564
DVU1065	206499	-1.32703	-2.47244
DVU1066	206500	-0.303797	-0.560186
DVU1067	206501	-0.416961	-0.793815
DVU1068	206502	1.02658	1.68464
DVU1069	206503	-1.10592	-1.80464
DVU1070	206505	-0.0677045	-0.1043
DVU1071	206506	-0.239769	-0.411622
DVU1072	206507	0.673941	1.15246
DVU1073	206508	0.981221	1.78414
DVU1074	206509	-1.88242	-3.22897
DVU1075	206510	-0.989363	-1.83842
DVU1076	206511	-0.965264	-1.73326
DVU1077	206512	-1.29289	-2.42102
DVU1079	206514	-0.224669	-0.332061
DVU1080	206515	0.557271	0.842999
DVU1081	206516	-1.35332	-2.32858
DVU1082	206517	-1.06102	-1.91001
DVU1083	206518	0.981472	1.76156
DVU1084	206519	-0.000567832	-0.000936122
DVU1085	206520	-0.632559	-1.22956
DVU1086	206521	0.915499	1.71118
DVU1087	206522	-0.159673	-0.268003
DVU1088	206523	0.114258	0.211658

DVU1089	206524	-1.58695	-2.7223
DVU1090	206525	-0.142456	-0.249002
DVU1091	206526	-0.354379	-0.626667
DVU1092	206527	-0.947373	-1.65452
DVU1093	206528	0.192426	0.368239
DVU1094	206529	-0.753994	-1.43316
DVU1095	206530	-1.27139	-1.99972
DVU1096	206531	-0.179932	-0.30457
DVU1097	206532	0.0883209	0.166206
DVU1098	206533	0.473096	0.868511
DVU1099	206534	-0.895637	-1.52547
DVU1100	206535	-0.656204	-1.10382
VIMSS206536	206536	-0.0778238	-0.0978051
DVU1103	206538	-0.00969479	-0.0161529
DVU1104	206539	-0.0466117	-0.0706192
DVU1105	206540	0.240894	0.370596
DVU1106	206541	0.46603	0.736377
DVU1107	206542	0.273255	0.371852
DVU1108	206544	-0.775609	-1.2853
DVU1109	206545	-2.45943	-3.56429
DVU1110	206546	-0.765195	-1.18547
DVU1111	206547	-0.0937963	-0.145478
DVU1113	206549	-0.325996	-0.540459
DVU1114	206550	-0.0202534	-0.0297434
DVU1115	206551	0.952643	1.52458
DVU1116	206552	0.246538	0.341919
DVU1117	206553	-0.0671616	-0.119815
DVU1118	206554	0.40001	0.576889
DVU1120	206556	-0.25969	-0.34076
DVU1121	206557	1.04536	1.92006
DVU1122	206558	-0.181706	0
DVU1123	206559	0.73899	0.878606
DVU1125	206560	0.678069	1.05256
DVU1124	206561	0.291463	0.384004
DVU1126	206562	-0.239399	-0.325095
DVU1128	206564	-0.0177346	-0.0258758
DVU1129	206565	-0.231648	-0.237192
DVU1130	206566	0.266132	0.397113
DVU1131	206567	-0.365173	-0.500128
DVU1132	206568	0.427273	0.597882
DVU1133	206569	-0.0258483	-0.0427444
DVU1134	206570	0.629269	1.04845
DVU1135	206571	0.745866	1.20778
DVU1136	206572	0.303565	0.549141
DVU1137	206573	-0.0615824	-0.0833233
DVU1138	206574	0.235392	0.377817
DVU1139	206575	0.0859221	0.119878
DVU1140	206576	0.170413	0.276795
DVU1141	206577	0.763452	1.20272
DVU1142	206578	1.14504	1.67668
DVU1143	206579	1.67896	0
DVU1144	206580	0.580744	1.05762
DVU1145	206582	0.0970179	0.175444
VIMSS206583	206583	0.171154	0.281348
DVU1147	206585	-0.363875	-0.495448
VIMSS206586	206586	-0.376341	-0.618294
VIMSS206587	206587	-0.0911467	-0.142749
VIMSS206588	206588	0.371805	0.506377
DVU1151	206589	0.0850234	0.110316
DVU1153	206591	-0.240609	-0.409513
DVU1154	206592	0.427771	0.739683
DVU1155	206593	-0.100589	-0.164523
DVU1157	206595	-0.476832	-0.727285
DVU1158	206596	-0.351586	-0.482577
DVU1159	206597	0.182609	0.333637
DVU1160	206598	0.0878749	0.147988
DVU1161	206599	0.47126	0.592978
DVU1162	206600	-0.106133	-0.144808
DVU1163	206601	-0.247254	-0.429086
DVU1164	206602	-0.0649069	-0.0980517
DVU1165	206603	0.352048	0.610877
DVU1166	206604	-0.928108	-1.40802

VIMSS206605	206605	-0.370431	-0.411528
VIMSS206606	206606	0.644186	1.09056
VIMSS206607	206607	0.836468	0.718837
DVU1168	206608	0.0397943	0.067872
DVU1169	206609	-0.788774	-1.42804
DVU1170	206610	-1.73807	-2.92567
VIMSS206611	206611	-0.193169	-0.328431
DVU1173	206613	-0.0671457	-0.120564
DVU1174	206614	0.12359	0.222421
DVU1175	206615	0.00271932	0.00506896
DVU1176	206616	-1.03414	-1.55875
DVU1177	206617	0.0300956	0.0555522
VIMSS206618	206618	-0.318412	-0.367727
DVU1179	206619	0.390071	0.647338
DVU1180	206620	0.469703	0.887024
DVU1181	206621	0.554922	0.988818
DVU1182	206622	-0.295082	-0.560474
VIMSS206623	206623	-0.644232	-1.10716
DVU1185	206624	-0.386454	-0.692176
DVU1186	206625	-0.121767	-0.228648
DVU1187	206626	-0.303157	-0.549052
DVU1188	206627	0.0743812	0.128389
DVU1189	206628	0.0116612	0.0218883
DVU1190	206629	0.336381	0.63375
DVU1191	206630	-0.678945	-1.24433
DVU1192	206631	0.119398	0
DVU1193	206632	0.155208	0.298301
DVU1194	206633	-0.493403	-0.908399
DVU1195	206634	-1.53907	-2.52108
DVU1196	206635	-0.552922	-0.979262
DVU1197	206636	-0.13051	-0.224184
DVU1198	206637	-0.617207	0
DVU1199	206638	-0.739136	-1.29943
DVU1200	206639	-0.443451	-0.76641
DVU1201	206640	-0.253568	-0.487094
DVU1202	206641	-0.723203	-1.24794
DVU1203	206642	-0.862971	-1.51494
DVU1204	206643	-1.33402	-1.80848
DVU1205	206644	-1.96999	-3.23865
DVU1206	206645	-1.79464	-2.91309
DVU1207	206646	-0.932991	-1.65998
DVU1209	206648	-0.77248	-1.31006
DVU1211	206650	-0.163579	-0.17175
DVU1212	206651	1.43758	2.04248
DVU1213	206652	-1.05803	-1.49327
DVU1214	206653	-0.389874	0
DVU1215	206654	-0.712739	-1.21898
VIMSS206655	206655	-0.900843	0
DVU1217	206656	-0.919742	-1.59803
DVU1218	206657	0.172034	0.309142
DVU1219	206658	-0.386935	-0.719248
DVU1220	206659	-1.23745	0
DVU1221	206660	-0.406216	-0.695264
DVU1222	206661	-0.855004	-1.59913
DVU1223	206662	-0.608853	-1.02742
DVU1224	206663	-0.536135	-0.953022
DVU1226	206664	-0.241278	-0.432832
DVU1225	206665	-0.419257	-0.753287
VIMSS206666	206666	-0.151693	-0.253895
DVU1228	206667	-1.11981	-1.85197
VIMSS206668	206668	0.329042	0.418548
DVU1230	206669	-0.0849473	-0.114114
DVU1231	206670	0.0515521	0.0925291
DVU1232	206671	1.67564	2.99343
DVU1233	206672	0.0900125	0.137654
DVU1235	206674	0.00117477	0.00189184
DVU1236	206675	-1.23418	-1.98387
DVU1237	206676	-1.14001	-1.82925
DVU1238	206677	-1.04455	-1.6877
DVU1239	206678	-0.200746	-0.330545
DVU1240	206679	-0.507742	-0.847945
DVU1241	206680	0.907263	1.59854

DVU1242	206681	-0.464979	-0.880937
DVU1243	206682	-0.203076	0
DVU1244	206683	0.7559	1.43822
DVU1245	206684	-1.17789	-2.28111
DVU1246	206685	0.00382908	0.00731715
DVU1247	206686	-0.627139	-1.16628
DVU1248	206687	-0.74897	-1.36667
DVU1249	206688	-1.11195	-1.8261
DVU1250	206689	0.0250049	0.0466399
DVU1251	206690	-0.690039	-1.16782
DVU1252	206691	-0.245258	-0.431969
DVU1253	206692	-0.0324979	-0.0571662
DVU1254	206693	-1.07739	-1.97559
DVU1255	206694	-0.218662	-0.355049
DVU1256	206695	-0.546749	-1.02024
DVU1258	206697	0.00954065	0.0170979
VIMSS206698	206698	0.493381	0.390247
DVU1260	206699	-2.14153	-3.76181
DVU1261	206700	-0.861097	0
DVU1262	206701	0.223948	0.425234
DVU1263	206702	0.447496	0.847899
DVU1264	206703	-0.790697	-1.17296
DVU1265	206704	-0.098925	-0.148877
DVU1266	206705	0.662382	1.23582
DVU1267	206706	0.0751937	0.140507
VIMSS206707	206707	-0.176511	-0.284557
DVU1268	206708	0.375869	0.424297
DVU1270	206709	-0.602988	-0.971418
DVU1271	206710	-0.907157	-1.61618
DVU1272	206711	-0.806076	-1.39179
DVU1273	206712	-0.0186807	-0.0347153
DVU1274	206713	-0.997402	-1.85308
DVU1275	206714	-0.748181	-1.33744
DVU1276	206715	-1.04489	-1.85538
DVU1277	206716	-1.1713	-1.97093
VIMSS206717	206717	0.576011	1.08069
DVU1278	206718	0.0805374	0.133728
DVU1279	206719	0.167297	0.300386
DVU1280	206720	-0.171145	-0.327738
DVU1281	206721	-0.0468969	-0.082234
DVU1282	206723	-0.0976239	0
DVU1283	206724	1.20141	2.18484
DVU1284	206725	-1.16308	-1.75716
DVU1285	206726	0.0242767	0.0438015
DVU1286	206727	-0.276808	-0.53377
DVU1287	206728	-0.00303471	-0.00519826
DVU1288	206729	-0.794433	-1.22947
DVU1289	206730	-0.476512	-0.688002
DVU1291	206732	0.443526	0.824604
DVU1292	206733	0.0841514	0.132475
DVU1294	206735	-0.206946	-0.380539
VIMSS206737	206737	-0.496892	-0.714189
DVU1297	206738	-0.6916	-0.967665
DVU1298	206739	-0.369787	-0.687623
DVU1299	206740	-1.68775	-2.57718
VIMSS206741	206741	-1.00214	-1.26751
DVU1300	206742	-0.869123	-1.58024
DVU1301	206743	-0.196173	-0.373361
DVU1302	206744	-0.223099	-0.423297
DVU1303	206745	-0.747946	-1.20444
DVU1304	206746	-0.627864	-1.12998
DVU1305	206747	-0.601658	-0.995666
DVU1306	206748	-0.652911	-1.12484
DVU1307	206749	-0.438006	-0.774662
DVU1308	206750	-0.67811	-1.11771
DVU1309	206751	-0.45139	-0.762917
DVU1310	206752	-0.89292	-1.53112
DVU1311	206753	-0.696565	-1.15892
DVU1312	206754	-0.708354	-1.23663
DVU1313	206755	-0.586419	-1.05932
DVU1314	206756	-0.0241505	-0.0411886
DVU1315	206757	-0.0739841	-0.125171

DVU1316	206758	-0.919631	-1.65725
DVU1317	206759	-0.647232	-1.16267
DVU1318	206760	-0.936749	-1.65982
DVU1319	206761	-0.619998	-1.11182
DVU1320	206762	-0.0640647	-0.120071
DVU1321	206763	-0.392285	-0.655095
DVU1322	206764	-0.47512	-0.872892
DVU1323	206765	0.114086	0.185381
DVU1324	206766	-0.0882195	-0.160689
DVU1325	206767	-0.461476	-0.871303
DVU1326	206768	-0.894236	-1.57369
DVU1327	206769	-0.919241	-1.41431
DVU1328	206770	-1.17351	-1.99177
DVU1329	206771	-0.852921	-1.5557
DVU1330	206772	-1.08756	-2.01433
DVU1331	206773	0.430968	0.778717
DVU1332	206774	0.0306721	0.0573842
DVU1333	206775	0.761012	1.39291
DVU1334	206776	-0.897007	-1.71442
DVU1335	206777	0.325803	0.601978
DVU1336	206778	0.0297311	0.0551659
DVU1337	206779	0.230431	0.434533
DVU1338	206780	0.30293	0.543264
DVU1339	206781	0.937149	1.69515
DVU1340	206782	0.72561	1.27782
DVU1341	206783	0.704902	1.36109
DVU1342	206784	0.927127	1.69097
DVU1343	206785	0.897767	1.71312
DVU1344	206786	-0.234976	-0.45466
DVU1345	206787	-0.302391	-0.563791
DVU1346	206788	-1.12352	-2.06903
DVU1347	206789	-1.24883	-2.18495
DVU1348	206790	-1.39822	-2.41373
DVU1349	206791	-0.379442	-0.664971
DVU1350	206792	-1.0433	-1.8716
DVU1351	206793	-0.797208	-1.28177
DVU1352	206794	-0.362234	-0.672895
DVU1353	206795	-0.606999	-0.990756
VIMSS206796	206796	-1.05102	-1.58256
DVU1355	206797	-0.0254322	-0.0473338
DVU1356	206798	-0.16047	-0.267077
DVU1357	206799	-0.757669	-1.33489
DVU1358	206800	-0.0355561	-0.0663997
DVU1359	206801	0.289306	0.531694
DVU1360	206802	0.292595	0.530441
DVU1361	206803	-0.835155	-1.26297
DVU1362	206804	-0.646077	-1.1577
DVU1363	206805	0.418067	0.796919
DVU1364	206806	0.290959	0.535977
DVU1365	206807	0.0720086	0.125723
DVU1366	206808	-0.251974	-0.45655
DVU1368	206809	0.0924995	0.173823
DVU1367	206810	-0.764025	-0.789334
DVU1369	206811	-0.259935	-0.490896
DVU1370	206812	-0.556214	-0.99259
DVU1371	206813	-0.386874	-0.713621
DVU1372	206814	0.50891	0.984167
DVU1373	206815	0.0518258	0.100113
DVU1374	206816	0.777488	1.4356
DVU1375	206817	1.1147	2.06144
DVU1376	206818	0.0984312	0.184632
DVU1377	206819	0.164111	0.301723
VIMSS206821	206821	0.183972	0.240973
DVU1380	206822	0.784856	1.40415
DVU1381	206823	-0.415302	-0.675205
VIMSS206826	206826	-0.769054	-1.30388
DVU1384	206827	0.116612	0.218245
VIMSS206828	206828	-0.413086	-0.606107
DVU1386	206829	-0.288439	-0.438903
DVU1387	206830	-0.205921	-0.370117
DVU1388	206831	1.21355	2.19982
DVU1389	206832	0.0909541	0.158948

DVU1390	206833	-0.337063	-0.599672
VIMSS206834	206834	-0.34486	-0.546547
DVU1392	206835	0.560565	0.981344
DVU1393	206836	-1.11522	-1.95564
DVU1394	206837	-0.340994	-0.56099
DVU1395	206838	-0.395734	-0.623168
VIMSS206839	206839	1.17017	2.13451
DVU1397	206840	1.65098	3.03698
DVU1400	206843	0.485153	0.888959
DVU1401	206844	-0.0256527	-0.0449863
DVU1402	206845	0.433914	0.736504
DVU1403	206846	0.0929352	0.160208
DVU1404	206847	0.469426	0.723868
DVU1405	206848	0.313982	0.56876
DVU1406	206849	-1.11364	-1.9921
DVU1408	206850	0.514887	0.798746
DVU1409	206851	0.350173	0.640387
DVU1407	206852	-0.303223	-0.585513
DVU1410	206853	0.504194	0.947088
DVU1411	206854	-2.3116	-3.7338
DVU1412	206855	0.448353	0.868637
DVU1413	206856	0.243687	0.461124
DVU1414	206857	0.181272	0.2815
VIMSS206858	206858	-0.526089	-0.753333
DVU1416	206859	-0.181874	-0.237483
VIMSS206860	206860	0.421726	0.493636
DVU1418	206861	-0.30278	-0.528604
DVU1419	206862	0.331883	0.580062
DVU1420	206863	0.882811	1.67282
VIMSS206864	206864	0.989267	1.7601
DVU1421	206865	0.148289	0
DVU1422	206866	-0.110529	-0.199871
DVU1423	206867	0.306834	0.579981
DVU1424	206868	-0.720633	-1.25837
DVU1425	206869	-0.285336	-0.534519
DVU1426	206870	-0.164889	-0.258804
DVU1427	206871	0.107813	0.199117
DVU1428	206872	0.552268	1.03086
DVU1429	206873	-0.338444	0
DVU1430	206874	0.79683	1.47893
DVU1431	206875	1.1121	2.08386
DVU1432	206876	0.0287143	0.052337
VIMSS206877	206877	0.571623	0.961975
DVU1434	206878	0.324014	0.602611
DVU1435	206879	-0.236714	-0.41984
DVU1436	206880	0.0884428	0.16643
VIMSS206881	206881	0.684303	1.12073
DVU1438	206882	1.04476	1.98809
VIMSS206883	206883	0.476009	0.848536
DVU1440	206884	0.496357	0.91662
DVU1441	206885	0.545529	0.927536
DVU1442	206886	1.34297	2.36486
DVU1443	206887	-0.123063	-0.155301
DVU1444	206888	0.508788	0.598402
DVU1445	206889	0.217236	0.323438
DVU1446	206890	0.512775	0.704058
DVU1447	206891	-0.408426	-0.73509
DVU1448	206892	0.0894104	0.167537
DVU1449	206893	0.416208	0.785024
DVU1450	206894	0.643875	1.24804
DVU1451	206895	-0.105505	-0.202194
DVU1452	206896	0.0311999	0.0552601
DVU1453	206897	-0.182846	-0.338512
DVU1454	206898	-0.0723468	-0.130656
DVU1455	206899	-0.543547	-0.949885
DVU1457	206901	1.06652	2.00114
DVU1458	206902	0.269299	0.485734
DVU1459	206904	-0.860286	-1.42073
DVU1460	206905	-0.050443	-0.097027
DVU1461	206906	-0.349857	-0.649284
DVU1462	206907	0.933806	1.74922
DVU1463	206908	0.804767	1.50148

DVU1464	206909	0.0722307	0.123501
DVU1465	206910	0.336366	0.60328
DVU1466	206911	-0.178846	-0.331815
DVU1467	206912	-0.460574	-0.853302
DVU1468	206913	0.321462	0.585785
DVU1469	206914	-0.731867	-1.20878
DVU1470	206915	-0.336835	-0.629015
DVU1471	206916	1.16747	2.15428
DVU1472	206917	1.1534	2.14644
VIMSS206918	206918	1.23251	1.67643
VIMSS206919	206919	2.28755	4.1947
DVU1474	206920	2.08344	3.24836
DVU1475	206921	0.447851	0.780808
DVU1476	206922	0.135556	0.163203
DVU1479	206927	-0.0968812	-0.156516
DVU1480	206928	0.375933	0.698377
DVU1483	206931	-0.144676	-0.223808
DVU1484	206932	-0.130285	-0.223693
DVU1488	206935	0.464921	0.712871
DVU1489	206936	1.17788	1.5928
DVU1491	206938	1.66489	2.79202
DVU1494	206941	0.775647	1.19447
DVU1499	206944	0.789256	0.984594
DVU1500	206945	0.960926	1.37205
DVU1501	206947	0.741545	1.03795
DVU1502	206948	0.945184	1.2286
DVU1503	206949	0.929509	1.35494
DVU1504	206950	0.476483	0.699682
DVU1505	206951	0.764007	0.930964
DVU1506	206952	0.72753	1.07798
DVU1507	206953	-0.633438	-1.03899
DVU1508	206954	-0.395548	-0.606054
DVU1509	206956	-0.269882	-0.491002
VIMSS206959	206959	-0.165279	-0.239696
DVU1513	206960	0.685233	0.79644
DVU1514	206961	0.463175	0.597688
DVU1515	206962	0.769191	1.01328
DVU1516	206963	0.524269	0.815027
DVU1517	206964	1.24817	0
DVU1518	206965	1.70418	0
DVU1520	206969	0.453215	0.710074
DVU1521	206970	0.547983	0.936539
DVU1522	206971	0.22468	0.32156
DVU1523	206972	1.17748	1.40465
DVU1524	206973	1.07284	1.71114
DVU1525	206974	1.17806	1.55795
DVU1527	206975	1.08763	1.30731
DVU1528	206976	0.625454	1.18129
DVU1529	206977	0.630407	1.21005
DVU1530	206978	-0.372549	-0.600399
DVU1531	206979	-0.0801207	-0.152608
DVU1532	206980	0.254858	0.449282
DVU1533	206981	-0.782652	-1.13754
DVU1534	206982	1.71281	2.59414
DVU1535	206983	1.77225	3.02299
DVU1536	206984	1.14735	2.02862
DVU1537	206985	-0.691646	-1.29399
DVU1538	206986	-0.754932	-1.4345
DVU1540	206988	-0.946186	-1.65946
DVU1541	206989	0.932242	1.76364
DVU1542	206990	0.288337	0.433788
DVU1543	206991	0.538531	0.849381
DVU1544	206992	0.353938	0.680111
DVU1545	206993	-0.00264287	-0.00441505
VIMSS206994	206994	-0.124171	-0.19978
DVU1547	206995	0.65077	1.22882
DVU1548	206996	-0.761715	-1.25742
DVU1549	206997	0.778277	1.37611
DVU1550	206998	0.364899	0
DVU1551	206999	-0.524695	-0.906399
DVU1552	207000	0.554047	0.820224
DVU1553	207001	0.268719	0.457203

DVU1554	207002	1.00859	1.60731
DVU1555	207003	1.13931	1.79474
DVU1556	207004	0.983566	1.38673
DVU1558	207006	1.0079	1.48417
DVU1559	207007	0.618199	1.07405
DVU1560	207008	0.567694	0.696537
DVU1561	207010	1.54051	0
DVU1562	207011	0.275552	0.490254
DVU1563	207012	1.13514	1.46708
VIMSS207013	207013	1.05828	1.19561
VIMSS207014	207014	1.32512	2.31241
VIMSS207015	207015	0.778853	0
DVU1566	207016	0.31532	0.531365
DVU1568	207018	0.892732	1.54365
DVU1569	207019	0.178602	0.304194
DVU1570	207020	0.688779	1.17883
DVU1571	207021	-0.557511	-0.990231
DVU1572	207022	0.996375	1.91391
DVU1573	207023	-0.633365	-1.10289
DVU1574	207024	-1.64743	-2.53933
DVU1575	207025	-1.22838	-2.175
DVU1576	207026	-1.57062	-2.93974
VIMSS207027	207027	0.599598	0.808844
DVU1577	207028	0.302335	0.553863
DVU1578	207029	0.615014	0.996794
DVU1579	207030	0.29753	0.563389
DVU1580	207031	-0.0540293	-0.102563
DVU1581	207032	-0.14462	-0.269779
DVU1582	207033	0.196763	0.379963
DVU1583	207034	-0.410303	-0.674025
DVU1584	207035	-0.784585	-1.37732
DVU1585	207036	-0.121701	-0.191006
DVU1586	207037	0.223296	0.410718
DVU1587	207038	-0.952125	-1.8418
DVU1589	207040	0.538245	0.930633
DVU1590	207041	0.38453	0.711889
DVU1591	207042	0.283074	0.445572
DVU1592	207043	1.029	1.89617
DVU1593	207044	1.2635	0
DVU1594	207045	1.0784	0
DVU1595	207046	0.799836	1.49707
DVU1596	207047	0.803545	1.43003
DVU1597	207048	0.503804	0.780425
DVU1599	207050	0.202457	0.342951
DVU1600	207051	0.641808	1.12924
DVU1601	207052	0.577479	0.966111
DVU1603	207054	1.00282	1.59518
DVU1604	207055	-0.465149	0
DVU1605	207056	0.0146668	0.0221713
DVU1606	207057	0.26415	0.475989
DVU1607	207058	-0.542738	-1.02676
DVU1608	207059	-0.871684	-1.6114
DVU1609	207060	-0.616574	-1.12932
DVU1610	207061	-0.0690371	0
DVU1611	207062	-0.255833	-0.461998
DVU1612	207063	0.781088	1.50624
DVU1613	207064	-0.281337	-0.539292
DVU1614	207065	-0.582169	-1.07911
VIMSS207066	207066	-0.123063	-0.228887
DVU1615	207067	-0.713432	-1.22317
DVU1617	207068	-0.768124	-1.45044
DVU1618	207069	-0.433273	-0.757413
DVU1619	207070	-0.499371	-0.904946
DVU1620	207071	1.00314	0
DVU1621	207072	-0.28229	-0.516668
DVU1622	207073	-1.08225	-1.92226
DVU1623	207074	0.230481	0.417851
DVU1624	207075	0.516278	0.818083
DVU1625	207076	-0.0519408	-0.0974001
DVU1626	207077	-0.753345	-1.39954
VIMSS207078	207078	0.18076	0.226012
DVU1627	207079	-0.400878	-0.770326

DVU1628	207080	-0.0877458	-0.162157
DVU1629	207081	0.952536	1.76508
DVU1630	207082	0.283244	0.501176
DVU1631	207083	-0.966126	-1.8502
DVU1632	207084	-0.832331	-1.52258
DVU1633	207085	-0.0882228	-0.162509
DVU1634	207086	0.491684	0.924451
DVU1635	207087	-1.01763	-1.87223
DVU1636	207088	-1.5381	-2.58822
DVU1638	207090	0.847242	1.53398
DVU1639	207091	-0.253132	-0.45179
DVU1641	207094	-0.0442548	-0.0742667
VIMSS207099	207099	-0.0819145	-0.109916
DVU1644	207100	0.100887	0.16395
DVU1645	207101	-0.956081	-1.73846
DVU1646	207104	0.200279	0.31335
DVU1647	207105	0.569174	1.09251
DVU1648	207106	0.195439	0.366998
DVU1649	207107	0.192643	0.371017
VIMSS207108	207108	-0.02705	-0.0395084
DVU1650	207109	-0.448515	-0.800703
DVU1651	207110	-0.195194	-0.333013
DVU1652	207111	-0.188308	-0.34718
DVU1655	207114	0.14393	0.274675
DVU1656	207115	0.812745	1.48477
DVU1657	207116	0.758043	1.4038
DVU1658	207117	0.331976	0.61802
VIMSS207118	207118	0.498985	0.832914
DVU1660	207119	-0.197875	-0.361066
DVU1661	207120	-0.793855	-1.36477
DVU1662	207121	-0.875602	-1.58068
DVU1663	207122	-1.36112	-2.23709
DVU1664	207123	-0.492688	-0.898332
DVU1665	207124	-1.35903	-2.39933
DVU1666	207125	-1.84233	-3.3676
DVU1667	207126	-0.0227455	-0.0425
DVU1668	207127	-0.64841	-1.10757
DVU1669	207128	-0.605852	-1.07413
DVU1670	207129	-0.00333195	-0.00569834
DVU1671	207130	-1.01228	-1.71859
DVU1672	207131	-0.103958	-0.193127
DVU1673	207132	-0.992032	-1.72906
DVU1674	207133	0.399231	0.749764
DVU1675	207134	1.19101	1.90188
DVU1676	207135	-1.10414	-1.9271
DVU1677	207136	0.196242	0.348245
DVU1678	207137	-0.656552	-1.14538
DVU1679	207138	0.382239	0.682946
DVU1680	207139	-0.204177	-0.386454
DVU1681	207140	0.297155	0.57122
DVU1682	207141	0.396822	0.749656
DVU1683	207142	-0.173048	-0.272051
DVU1684	207143	-0.210263	-0.393551
DVU1685	207144	0.761003	1.45533
DVU1686	207145	0.577071	1.127
DVU1687	207146	0.0820199	0.124895
DVU1688	207147	-0.414351	-0.758526
VIMSS207148	207148	-0.624084	-0.969105
DVU1690	207149	-1.46027	-2.6654
VIMSS207150	207150	-0.240055	-0.28671
DVU1692	207151	0.230673	0.434754
DVU1693	207152	-0.00984106	-0.0183863
DVU1694	207153	-0.236199	-0.389694
DVU1695	207154	0.217684	0.338088
DVU1696	207155	0.129811	0.139534
DVU1697	207156	0.491124	0.686424
DVU1698	207157	0.65619	0.998174
DVU1699	207159	0.210566	0
DVU1701	207161	-0.0754342	-0.109761
DVU1703	207163	0.392874	0.720766
VIMSS207164	207164	-0.496851	-0.860205
DVU1705	207165	-0.10098	-0.168886

DVU1707	207166	1.46446	0
DVU1708	207167	0.291301	0.47829
DVU1709	207168	0.424131	0.712488
DVU1711	207171	0.0940982	0.166814
DVU1713	207173	0.447741	0.604185
DVU1714	207174	0.621338	0.863572
DVU1715	207175	0.329479	0.397968
DVU1716	207176	0.348531	0.457428
DVU1717	207177	0.393327	0.56549
DVU1718	207178	0.116204	0.136753
DVU1719	207179	0.491131	0.635792
DVU1720	207180	0.453142	0.4312
DVU1721	207181	0.46897	0.612359
DVU1723	207183	0.856317	0
DVU1724	207184	1.67901	1.61009
DVU1725	207185	0.463277	0.70287
DVU1727	207186	0.741015	0.921247
DVU1726	207187	0.0147382	0.0211301
DVU1728	207188	-0.20289	-0.332599
DVU1729	207189	0.642496	1.17797
DVU1730	207190	0.74804	1.24009
DVU1731	207191	0.882866	0
VIMSS207192	207192	0.865956	0.765797
VIMSS207194	207194	0.741062	1.23248
VIMSS207195	207195	0.727248	1.1649
DVU1736	207197	0.323012	0.491472
DVU1737	207198	0.309492	0.511097
DVU1738	207199	0.602524	0.827727
VIMSS207200	207200	2.32492	2.38071
DVU1740	207201	1.40772	0
DVU1741	207202	0.404523	0.561674
DVU1742	207203	0.274974	0.490284
DVU1743	207204	-0.0396458	-0.0608782
DVU1744	207205	-0.283927	-0.394282
DVU1746	207208	0.218344	0.391019
VIMSS207210	207210	1.36529	1.14141
VIMSS207212	207212	0.348944	0.507191
DVU1750	207213	-1.45158	-2.39294
DVU1751	207214	-1.92533	0
VIMSS207215	207215	-0.894553	-1.58421
DVU1752	207216	0.329045	0.473611
DVU1753	207217	-0.0112066	-0.0157318
DVU1754	207218	0.546976	0.785024
VIMSS207219	207219	0.732065	1.16566
VIMSS207220	207220	0.331461	0.556468
DVU1756	207221	-0.435663	-0.763412
DVU1758	207223	0.496496	0.87832
DVU1759	207224	0.266157	0.429961
DVU1760	207225	0.0421426	0.0700581
DVU1762	207227	0.253282	0.43621
VIMSS207228	207228	0.468432	0.703006
DVU1764	207229	-0.643207	-1.20424
DVU1765	207230	-0.25901	-0.381417
DVU1766	207231	0.531044	0.671972
DVU1767	207232	0.261777	0.429274
DVU1768	207233	-0.0302915	-0.0413031
DVU1769	207234	0.156722	0.26307
DVU1770	207235	1.28178	1.92384
DVU1771	207236	0.0700676	0.127193
DVU1772	207237	-0.4803	-0.86452
VIMSS207238	207238	0.188147	0.336207
DVU1774	207239	0.103226	0.19059
DVU1775	207240	-0.948658	-1.73414
DVU1776	207241	-0.543678	-0.852575
DVU1777	207242	-0.543307	-1.00603
DVU1778	207243	-0.0560584	-0.105029
DVU1779	207244	1.02628	1.66996
DVU1780	207245	1.30593	2.39888
DVU1781	207246	1.35539	2.59676
DVU1782	207247	1.84339	3.57882
DVU1783	207248	1.66971	3.08918
DVU1784	207249	0.0203108	0.0388707

DVU1785	207250	0.412959	0.660558
DVU1786	207251	0.494487	0.92193
DVU1787	207252	-0.364235	-0.677741
DVU1788	207253	-0.334885	-0.653436
DVU1789	207254	-0.371821	-0.576983
DVU1791	207256	-0.530571	-0.982637
DVU1792	207257	-0.731383	-1.36449
VIMSS207258	207258	-0.218576	-0.407947
DVU1794	207259	-0.3909	-0.735397
DVU1795	207260	0.592257	1.10859
DVU1797	207262	-0.278097	-0.498469
DVU1798	207263	-0.286048	-0.538537
DVU1799	207264	0.526158	0.955143
VIMSS207265	207265	0.777859	1.18146
DVU1801	207266	-0.280611	0
DVU1802	207267	-0.0506386	-0.0847764
DVU1803	207268	0.180495	0.319726
DVU1804	207269	0.883098	0
DVU1805	207270	0.553181	1.01402
DVU1806	207271	-0.266903	-0.451843
DVU1807	207272	-0.184946	-0.266931
DVU1808	207273	-0.454743	-0.781449
DVU1809	207274	0.736371	1.40425
DVU1810	207275	0.891901	1.53295
DVU1811	207276	1.05106	1.90557
DVU1812	207277	1.01675	1.82505
DVU1813	207278	1.54554	2.68304
DVU1814	207279	1.44808	2.75412
DVU1816	207281	1.18843	2.24835
DVU1818	207283	-0.916346	-1.69229
DVU1819	207284	-1.04757	-1.99767
DVU1820	207285	-0.592083	-1.00416
DVU1821	207286	-0.768686	0
DVU1822	207287	0.318108	0.558652
DVU1823	207288	0.0705199	0.118551
DVU1824	207289	1.27764	2.01286
DVU1825	207290	0.191933	0
DVU1826	207291	-2.03953	-3.60914
DVU1827	207292	-0.440219	0
DVU1828	207293	-1.68658	-2.55628
DVU1830	207295	0.207368	0.391043
DVU1832	207297	0.389025	0.683192
DVU1833	207298	-0.490902	-0.91364
DVU1834	207299	0.940813	1.57412
DVU1835	207300	-0.0679536	0
VIMSS207301	207301	-0.128283	-0.246053
DVU1836	207302	0.388238	0
DVU1837	207303	-0.961306	-1.52216
DVU1838	207304	0.690784	1.3157
DVU1839	207305	1.49503	2.87822
DVU1840	207306	-0.899233	0
DVU1841	207307	-1.00439	-1.6747
DVU1842	207308	-0.19547	-0.376919
DVU1843	207309	-0.797461	-1.37504
DVU1844	207310	0.400612	0
DVU1845	207311	0.626493	1.11801
DVU1846	207312	0.908884	1.53818
DVU1847	207313	-0.00694308	-0.0122133
DVU1848	207314	-0.460321	0
DVU1849	207315	-0.945265	-1.80559
DVU1850	207316	0.211722	0
DVU1851	207317	-0.406288	-0.728542
DVU1853	207318	1.42349	0
DVU1854	207319	0.655023	0.894039
VIMSS207320	207320	0.497363	0.47085
DVU1855	207321	0.82576	1.26672
VIMSS207322	207322	-0.446077	0
DVU1857	207323	0.963671	1.75359
DVU1859	207325	0.908143	1.634
DVU1860	207326	-0.574933	-1.03346
DVU1861	207327	1.16098	2.18659
DVU1862	207328	1.4374	2.49674

DVU1863	207329	-0.356012	-0.618717
DVU1865	207331	-0.0990729	-0.17056
DVU1866	207332	-0.0575891	-0.104006
DVU1867	207333	0.655768	1.23721
DVU1868	207334	0.47725	0.899841
DVU1869	207335	0.670669	1.2199
DVU1870	207336	0.016762	0
VIMSS207338	207338	0.362237	0
DVU1873	207339	-1.23813	-2.04358
DVU1874	207340	0.657955	1.24946
DVU1875	207341	0.539904	1.03372
DVU1876	207342	0.599881	1.11335
DVU1877	207343	0.0856135	0.151607
DVU1881	207347	0.404264	0.73998
DVU1882	207348	-0.424178	0
DVU1883	207349	-0.15704	-0.281799
DVU1884	207350	0.596938	0.921793
DVU1886	207352	-0.134267	0
DVU1887	207353	-0.518126	-0.963314
DVU1888	207354	-0.0496634	0
DVU1889	207355	-0.817011	-1.51711
VIMSS207356	207356	0.307026	0
DVU1890	207357	-0.243604	0
DVU1891	207358	-0.00613507	0
DVU1892	207359	0.619035	1.13329
DVU1894	207361	1.45209	2.56421
DVU1895	207363	0.278132	0.459257
DVU1896	207364	-0.827294	-1.28694
DVU1898	207366	-0.49731	-0.919288
DVU1899	207367	0.99224	1.47264
DVU1900	207368	-0.511253	-0.963715
DVU1901	207369	0.398251	0.775817
DVU1902	207370	0.0140098	0.0220435
DVU1903	207371	-0.14639	-0.257715
DVU1904	207372	0.787845	1.45486
VIMSS207374	207374	-0.11656	-0.184431
VIMSS207375	207375	-0.551529	-0.626043
DVU1909	207378	1.11937	0
DVU1910	207379	-0.598692	0
DVU1911	207380	-0.713006	-1.17856
DVU1913	207382	-0.417773	0
DVU1915	207384	0.305219	0.568388
DVU1916	207385	0.28738	0.488526
DVU1919	207388	-0.124069	-0.21919
VIMSS207389	207389	0.457122	0.568082
DVU1921	207390	1.36431	1.84685
DVU1922	207391	1.25137	2.07483
DVU1923	207392	0.345742	0
DVU1924	207393	0.688965	0
DVU1925	207394	0.516799	0.931397
DVU1926	207395	0.0674586	0.126511
VIMSS207396	207396	-0.0935527	-0.155227
DVU1927	207397	-1.46084	0
DVU1928	207398	-0.711363	-1.29252
DVU1929	207399	-1.69789	0
DVU1930	207400	-0.316451	0
DVU1931	207401	-0.156973	-0.273885
DVU1932	207402	-0.151707	-0.24255
DVU1933	207403	-0.507349	0
DVU1934	207404	-0.468614	-0.83146
DVU1935	207405	-1.1981	0
DVU1936	207406	-0.479908	-0.888495
DVU1937	207407	0.0439636	0.0807414
DVU1938	207408	0.0466677	0.0849965
DVU1939	207409	0.229975	0.432412
DVU1940	207410	0.405247	0.693474
DVU1941	207411	0.417188	0.676308
DVU1942	207412	-0.74801	-1.39554
DVU1943	207413	-0.663462	-1.24882
DVU1944	207414	1.94459	0
DVU1945	207415	1.81456	0
DVU1946	207416	1.86441	3.54678

DVU1947	207417	1.40514	0
DVU1948	207418	0.349587	0.593849
DVU1949	207419	-0.924958	0
DVU1950	207420	-0.353548	-0.661178
DVU1951	207421	-0.573707	0
DVU1952	207422	-0.374197	-0.645427
DVU1953	207423	-0.35645	-0.698207
DVU1954	207424	-0.910104	-1.57204
DVU1955	207425	-0.518275	0
DVU1956	207426	-0.0843848	-0.154489
VIMSS207427	207427	0.0415721	0.0749692
DVU1958	207428	1.37587	2.35216
DVU1960	207430	-0.482363	-0.726164
DVU1961	207431	-0.0315086	-0.0536318
VIMSS207433	207433	-0.476095	-0.581894
VIMSS207435	207435	0.520234	0
VIMSS207436	207436	0.673925	0
DVU1967	207437	0.673757	0
DVU1968	207438	0.31482	0.411716
DVU1969	207439	-0.310415	0
DVU1970	207440	0.600134	0.79017
DVU1971	207441	-1.02089	0
DVU1975	207446	-0.611449	0
DVU1976	207447	-1.52026	-2.37809
DVU1977	207448	-0.6772	-1.19239
DVU1978	207449	-0.920154	-1.66049
DVU1980	207451	0.59284	1.146
DVU1981	207452	-0.485038	0
DVU1983	207454	0.1917	0
DVU1984	207455	0.547342	1.03938
DVU1985	207456	-0.694365	-1.16195
DVU1986	207458	1.16891	0
DVU1987	207459	-0.481401	-0.756561
DVU1988	207460	-0.57334	-0.901387
VIMSS207461	207461	0.00114991	0.00160212
DVU1991	207463	0.492161	0
DVU1992	207464	-0.279842	-0.480902
DVU1993	207465	0.596015	1.11762
VIMSS207466	207466	1.24772	1.83915
DVU1995	207467	0.627827	0
DVU2000	207472	-0.276837	-0.472546
VIMSS207475	207475	1.66233	2.30763
DVU2003	207476	0.426805	0.610933
DVU2006	207479	-1.03901	0
DVU2007	207480	-0.688593	-1.0096
DVU2008	207481	2.48741	0
DVU2009	207482	-0.554908	-0.929003
DVU2010	207483	-0.987982	-1.81139
DVU2012	207485	1.53843	2.12608
DVU2013	207486	1.19601	2.09174
DVU2014	207487	1.32114	2.42196
VIMSS207488	207488	-0.260332	-0.401231
DVU2016	207489	-0.36759	-0.668983
DVU2017	207490	0.0104023	0
VIMSS207491	207491	-0.804636	-1.38555
DVU2019	207492	-0.390804	-0.636815
DVU2020	207493	-0.356772	-0.593493
DVU2021	207494	0.173989	0.325131
VIMSS207495	207495	-0.0751699	-0.122053
DVU2023	207496	-0.513143	-0.843322
DVU2024	207497	0.0705343	0.116829
DVU2025	207498	-1.019	-1.69074
DVU2026	207499	0.87248	1.52614
VIMSS207500	207500	0.807936	0.701108
DVU2028	207501	0.0430466	0.0565312
VIMSS207504	207504	0.554603	1.04852
DVU2032	207505	0.00621251	0.00869122
DVU2033	207506	0.129059	0.143863
DVU2034	207507	1.47422	1.24556
DVU2035	207508	1.48273	2.58638
DVU2036	207509	2.12559	3.34196
DVU2037	207510	0.361952	0.499513

DVU2038	207511	-0.222191	-0.212942
DVU2039	207512	-0.139822	-0.201794
DVU2040	207513	-0.950716	-1.21578
DVU2041	207514	-0.11095	-0.127929
DVU2042	207515	0.131825	0.247617
DVU2043	207516	-0.523371	-0.490916
DVU2044	207517	-0.155331	-0.212295
DVU2045	207518	0.0375338	0.053919
DVU2046	207519	0.462704	0.690893
VIMSS207520	207520	0.515581	0.429759
DVU2048	207521	0.856761	1.5654
DVU2049	207523	1.1198	2.15053
DVU2051	207524	-0.106055	-0.170029
DVU2052	207525	-0.81065	-0.971353
DVU2053	207526	-0.795683	-1.52003
DVU2054	207527	-0.556728	-0.964007
DVU2055	207528	-0.485654	-0.911737
VIMSS207529	207529	-0.354727	-0.616593
DVU2057	207530	-0.691474	-1.21957
DVU2058	207531	-0.198014	-0.298042
DVU2059	207532	-0.855104	-1.45644
DVU2060	207533	-0.131476	-0.220479
DVU2061	207534	0.302142	0.530708
DVU2062	207535	0.0308959	0.0506854
DVU2063	207536	-0.647071	-1.08732
DVU2064	207537	-0.0584057	-0.107015
VIMSS207538	207538	-0.418369	-0.633213
DVU2066	207539	-0.638037	-1.12771
DVU2067	207540	-0.313516	-0.56461
DVU2068	207541	0.359325	0.698074
DVU2069	207543	-0.417547	-0.752231
DVU2070	207544	0.0738744	0.1432
DVU2071	207545	-0.949975	-1.72967
DVU2073	207547	1.24007	2.36873
DVU2074	207548	0.0907271	0.173106
DVU2075	207549	0.656148	1.2256
DVU2076	207550	0.397765	0.758946
DVU2077	207551	0.516309	0.997063
DVU2078	207552	0.391688	0.732865
DVU2079	207553	0.504907	0.905471
VIMSS207554	207554	-0.126717	-0.200807
DVU2081	207555	0.256709	0.303061
DVU2082	207556	0.362864	0.577282
DVU2083	207557	-0.884464	-1.70963
DVU2084	207558	-0.0239437	-0.0457803
DVU2085	207559	-1.26964	-2.36031
DVU2086	207560	0.886134	1.46711
DVU2087	207561	0.131935	0.130799
DVU2088	207562	-0.0844267	-0.140555
VIMSS207563	207563	1.07166	0.924267
VIMSS207564	207564	0.680913	0.677414
DVU2090	207565	0.653982	0.811842
DVU2091	207566	-1.47778	-2.7199
DVU2092	207567	-1.7781	-3.00848
DVU2093	207568	-2.64533	-4.43728
DVU2096	207571	-0.737603	-1.12132
DVU2097	207572	1.0985	1.34815
DVU2098	207573	-0.542308	-0.852521
DVU2099	207574	0.178264	0.256103
DVU2100	207575	3.17125	5.52104
VIMSS207576	207576	0.214707	0.407768
DVU2101	207577	0.449891	0.768128
DVU2102	207578	0.420865	0.783767
DVU2104	207580	0.992991	1.78989
DVU2105	207581	2.06183	0
DVU2106	207582	0.0227084	0.0313277
DVU2107	207583	0.0519032	0.0399227
DVU2108	207584	0.391925	0.700151
DVU2109	207585	0.485907	0
DVU2110	207586	0.133618	0.192724
DVU2111	207587	-0.225555	-0.369616
DVU2112	207588	0.740867	1.40334

DVU2113	207589	-0.962531	-1.23333
DVU2114	207590	1.01808	1.96987
DVU2115	207591	1.44179	2.33331
DVU2116	207593	1.16349	1.87346
DVU2117	207594	-0.0491383	-0.0568173
DVU2118	207595	1.04525	1.67273
DVU2119	207596	0.347103	0.478785
DVU2120	207597	0.514974	0.655303
DVU2121	207598	0.414787	0.542105
DVU2123	207600	-0.135294	0
DVU2124	207601	2.36335	2.34479
DVU2125	207602	0.859555	0
DVU2126	207603	0.4371	0.626433
DVU2127	207604	0.702607	0.889545
DVU2128	207605	0.0910335	0.165441
DVU2130	207607	0.154465	0.284318
DVU2131	207608	-0.723398	0
DVU2132	207609	0.0573631	0.0876632
DVU2133	207610	0.537634	0
DVU2135	207612	0.298314	0.340409
DVU2136	207613	0.686921	0
DVU2138	207615	0.318721	0.613285
DVU2140	207617	0.103072	0.201579
DVU2142	207619	-0.40547	-0.743609
DVU2144	207621	0.0562211	0.097525
DVU2145	207623	-0.299849	-0.520824
DVU2146	207625	-0.462267	-0.788503
DVU2147	207626	-0.0585496	0
DVU2148	207627	0.582714	1.02034
DVU2149	207628	-0.134772	0
DVU2150	207629	-1.10695	-1.91207
DVU2151	207630	-0.199157	0
DVU2152	207631	0.493494	0.847274
DVU2154	207633	0.28261	0.364616
DVU2155	207634	1.03845	0
DVU2156	207635	0.549658	0
DVU2157	207636	0.556381	0.876491
DVU2160	207640	0.195824	0
VIMSS207643	207643	0.579163	0.889999
DVU2165	207645	0.92865	0
DVU2166	207646	0.359384	0.521647
DVU2168	207648	-0.272372	-0.369223
DVU2171	207651	0.710419	0.985802
DVU2172	207652	0.528903	0.649947
DVU2173	207653	0.699089	0.675077
DVU2175	207656	-0.601597	0
DVU2176	207658	-0.170839	-0.283349
DVU2177	207659	-0.616274	-1.10951
DVU2180	207663	-0.47714	-0.71837
DVU2181	207664	0.0279313	0.0432533
VIMSS207666	207666	0.483756	0
DVU2184	207668	-0.446411	-0.660325
DVU2185	207669	-0.259305	0
DVU2187	207671	-0.138934	-0.186049
DVU2188	207672	-0.113162	0
DVU2189	207673	0.71803	0
DVU2190	207674	0.711925	1.07403
DVU2191	207675	0.635524	0.872173
DVU2192	207676	0.83699	1.16342
DVU2194	207678	1.33704	1.75342
DVU2195	207679	0.872513	1.53739
DVU2196	207680	0.721028	0.914493
DVU2197	207681	0.529473	0.811818
DVU2198	207682	0.669783	0
VIMSS207683	207683	0.139104	0.165731
DVU2200	207684	-0.717697	-1.23804
DVU2202	207686	0.505588	0.854357
DVU2203	207687	-1.55934	-2.57214
DVU2204	207688	-0.321961	-0.553571
DVU2205	207689	-1.4247	0
DVU2206	207690	-1.63681	-2.49317
DVU2208	207692	-0.301938	-0.505777

DVU2209	207693	-0.261332	0
DVU2210	207694	-1.28554	-2.29365
DVU2211	207695	0.344129	0.550843
DVU2212	207696	0.868536	1.63665
DVU2213	207697	0.865771	1.21699
DVU2214	207698	-0.230225	-0.312441
DVU2216	207700	-0.162781	-0.314092
DVU2217	207701	0.417829	0.557229
DVU2218	207702	0.705527	1.00318
VIMSS207703	207703	-0.226492	-0.318
DVU2220	207704	-1.08851	-1.83928
DVU2221	207705	0.269975	0.478838
DVU2222	207706	-1.56382	-2.61022
DVU2223	207707	-0.987161	-1.83214
DVU2224	207708	0.0203469	0.0327922
DVU2225	207709	-0.773245	-1.33843
DVU2226	207710	-0.758903	-1.30052
DVU2227	207711	-0.0525685	-0.0976817
DVU2228	207712	0.837282	1.51143
DVU2229	207713	0.277843	0.476864
DVU2230	207714	-0.595947	-1.06056
DVU2231	207715	-1.0785	-1.96703
DVU2232	207716	0.859802	1.66564
DVU2233	207717	-0.153358	-0.287816
DVU2234	207718	-0.384814	-0.627561
DVU2235	207719	-0.236621	-0.458555
DVU2236	207720	0.1184	0.221718
DVU2237	207721	-0.487821	-0.785342
DVU2238	207722	0.0717246	0.135253
DVU2239	207723	1.04354	1.67467
DVU2240	207724	-0.227529	-0.434554
DVU2241	207725	0.155595	0.282485
DVU2242	207726	0.110307	0.170781
DVU2243	207727	0.210941	0.396592
DVU2244	207728	0.24247	0.462923
DVU2245	207729	0.400788	0.743089
DVU2246	207731	-0.760842	-1.39689
DVU2247	207732	0.630425	0.692221
VIMSS207733	207733	-0.129093	-0.170124
VIMSS207734	207734	1.14855	1.44596
DVU2250	207735	-0.318706	-0.590931
DVU2251	207736	1.16946	2.19717
DVU2252	207737	-0.636342	-0.82687
DVU2253	207738	0.913126	0.854926
DVU2254	207739	-0.307606	-0.565712
DVU2255	207740	-0.408374	-0.801139
DVU2256	207741	-0.306183	-0.565314
DVU2257	207742	-1.62371	-2.58476
DVU2258	207743	-1.23082	-2.27126
DVU2259	207744	-1.03406	-1.6744
DVU2260	207745	-0.74095	-1.29775
DVU2261	207746	0.00575671	0.0109514
VIMSS207747	207747	0.799487	0.953577
DVU2263	207748	-0.011324	-0.0203367
DVU2264	207749	0.649006	1.22642
VIMSS207750	207750	-1.13838	-2.02151
VIMSS207751	207751	1.06745	0
DVU2267	207752	-0.105433	-0.188401
DVU2268	207753	0.565074	0.983833
DVU2269	207754	-0.189585	-0.335383
DVU2270	207755	0.150122	0.279896
DVU2271	207756	0.634179	0.909261
DVU2272	207757	0.258302	0.389842
VIMSS207758	207758	0.21405	0.276235
DVU2274	207759	-0.252151	-0.375216
DVU2275	207760	-1.35113	-2.13979
DVU2276	207761	-0.843565	-1.33438
DVU2277	207762	0.43556	0.65342
DVU2278	207763	1.5936	0
VIMSS207764	207764	-0.084307	-0.139234
DVU2279	207765	0.177858	0.236244
DVU2280	207766	-0.563175	-0.937873

DVU2281	207767	1.53107	2.5697
DVU2282	207768	0.975919	1.70742
DVU2283	207769	0.837689	1.34478
DVU2284	207770	-0.153828	-0.283958
DVU2285	207771	-3.53335	-5.22275
DVU2286	207772	-3.2099	-6.0347
DVU2287	207774	-3.25154	-4.38343
DVU2288	207775	-3.14202	-4.58788
DVU2289	207776	-2.86677	-4.30687
DVU2290	207777	-2.90167	-4.8817
DVU2291	207778	-2.54954	-3.84877
DVU2292	207779	-1.99832	-3.7528
DVU2293	207780	-1.68813	-2.92856
DVU2294	207781	-0.752491	-1.18447
DVU2295	207782	0.26197	0.46937
DVU2296	207783	0.411996	0.68147
DVU2297	207784	0.670921	1.22291
DVU2298	207785	0.0955069	0.181531
DVU2299	207786	0.327469	0.611007
DVU2300	207787	-0.286704	-0.426311
DVU2301	207788	-0.000119138	-0.000150224
DVU2302	207789	0.752223	1.35767
DVU2303	207790	0.211366	0.394872
VIMSS207791	207791	0.648528	1.14156
DVU2305	207792	-1.09767	-1.75339
DVU2306	207793	-1.19783	-2.1455
DVU2307	207794	-0.154666	-0.280549
DVU2308	207795	-0.00619537	-0.0118141
DVU2309	207796	1.95843	3.68461
DVU2310	207797	-0.941608	-1.42963
VIMSS207798	207798	-0.254673	-0.234707
DVU2312	207799	0.192965	0.360004
DVU2313	207800	0.442379	0.784715
VIMSS207801	207801	0.0945669	0.137875
DVU2315	207802	-0.331025	-0.631637
DVU2316	207803	-0.329511	-0.501576
DVU2317	207804	-0.250888	-0.396591
DVU2318	207805	-1.74453	-3.27502
DVU2319	207806	-0.0696786	-0.117168
DVU2320	207807	-0.730417	-1.3545
VIMSS207808	207808	-0.57202	-0.891203
DVU2322	207809	0.125153	0.220543
DVU2323	207810	-1.04286	-1.85841
DVU2324	207811	0.528583	0.817567
DVU2325	207812	0.835081	1.34929
DVU2326	207813	-0.0230751	0
VIMSS207814	207814	0.12203	0.147022
DVU2328	207815	-0.0984949	-0.184449
DVU2329	207816	-0.583916	-1.12785
DVU2330	207817	-0.167414	-0.316028
DVU2331	207818	-0.0126965	-0.0230905
DVU2332	207819	-0.0156479	-0.0300877
DVU2333	207820	-1.00567	-1.7531
VIMSS207821	207821	0.481708	0.786282
DVU2335	207822	-2.04106	-3.56338
DVU2336	207823	-0.229765	-0.37269
DVU2337	207824	0.0619598	0.110888
DVU2338	207825	0.480913	0.902277
DVU2339	207826	-0.474305	-0.881651
DVU2340	207827	-0.215364	-0.355999
DVU2341	207828	0.885943	1.5805
DVU2342	207829	1.28147	2.3469
DVU2343	207830	0.529677	0.855298
VIMSS207832	207832	0.832881	1.06726
DVU2345	207833	2.49907	4.36768
VIMSS207834	207834	1.46346	1.74571
DVU2347	207835	-0.354737	-0.647864
DVU2348	207836	-0.687702	-1.1044
DVU2349	207837	1.64248	2.94484
DVU2350	207838	0.059979	0.0971783
DVU2351	207839	0.787492	1.41258
DVU2352	207840	0.0609192	0.110164

DVU2353	207841	0.28806	0.493366
DVU2354	207842	0.086627	0.166344
DVU2355	207843	-0.70682	-1.24382
DVU2356	207844	-0.363461	-0.671707
DVU2357	207845	1.02514	1.89593
VIMSS207846	207846	-0.338877	-0.585083
DVU2359	207847	0.285147	0.471448
DVU2360	207848	1.0024	1.9226
VIMSS207849	207849	0.405708	0.751321
DVU2363	207850	0.522518	0.936532
DVU2362	207851	0.787808	1.34333
DVU2364	207852	-1.4669	-2.35725
DVU2365	207853	-0.635248	-1.02128
VIMSS207854	207854	1.54657	2.91423
DVU2367	207855	-0.873975	-1.58505
DVU2368	207856	-0.773037	-1.50236
DVU2369	207857	-0.652909	-1.1485
DVU2370	207858	-0.840117	-1.47816
DVU2371	207859	-0.61371	-1.10342
DVU2372	207860	-0.149277	-0.268802
DVU2373	207861	-1.7746	0
DVU2374	207862	-2.07673	-4.01263
DVU2375	207863	-1.46141	-2.64044
DVU2376	207864	-1.74139	-3.08672
DVU2377	207865	-0.123787	-0.201338
DVU2378	207866	0.764159	1.16071
DVU2379	207867	0.767779	1.43646
DVU2380	207868	0.283254	0.434466
DVU2381	207869	-0.122401	-0.174742
DVU2382	207870	0.178067	0.257743
DVU2383	207871	-0.236993	-0.351688
DVU2384	207872	1.86419	3.40967
DVU2385	207873	1.95923	3.59427
DVU2386	207874	0.445462	0.767295
DVU2387	207875	2.0254	3.7965
DVU2388	207876	-0.369433	-0.688574
DVU2389	207877	0.38366	0.647974
DVU2390	207879	0.662311	1.15713
DVU2391	207881	1.13438	1.04773
VIMSS207882	207882	1.39638	2.17722
DVU2392	207883	0.914533	1.73166
VIMSS207884	207884	0.507439	0.855274
DVU2394	207885	0.536322	0.980949
DVU2395	207886	0.916751	1.79349
DVU2396	207887	0.0997483	0.182356
DVU2397	207888	1.03704	1.97423
DVU2398	207889	1.57813	2.96194
DVU2399	207890	0.408753	0.795032
DVU2400	207891	0.780627	1.40906
DVU2401	207892	1.03707	1.81641
DVU2402	207893	1.27668	2.33696
DVU2403	207894	1.0811	1.924
DVU2404	207895	1.36287	2.59785
DVU2407	207898	0.711889	1.09988
DVU2408	207899	1.14521	1.94922
DVU2409	207900	0.458439	0.897959
DVU2410	207901	1.79921	3.0609
DVU2411	207902	0.79407	1.49898
DVU2412	207903	-0.716616	-1.22436
DVU2413	207904	-0.413539	-0.78519
DVU2414	207905	0.873975	1.55572
DVU2416	207907	0.648919	1.26554
DVU2417	207908	0.119388	0.225309
DVU2418	207909	0.484324	0.755452
DVU2419	207910	0.00621645	0.0100503
DVU2420	207911	-0.4735	-0.930697
DVU2421	207912	1.15717	2.25274
DVU2422	207913	1.33733	2.44097
VIMSS207914	207914	1.04595	1.47848
DVU2423	207915	1.38997	2.56071
DVU2424	207916	0.582506	1.07486
DVU2425	207917	-0.477848	-0.85019

VIMSS207918	207918	0.629271	0.790462
DVU2427	207919	0.550555	1.02622
DVU2428	207920	0.0621366	0.113253
DVU2429	207921	0.443898	0.747707
DVU2431	207923	0.418034	0.710238
DVU2432	207924	0.779651	1.27324
VIMSS207925	207925	-1.27621	-1.6292
DVU2434	207926	-0.68586	-1.0001
DVU2435	207927	0.000174724	0.000326197
DVU2436	207928	-0.762182	-1.38189
DVU2437	207929	0.073477	0.127772
VIMSS207931	207931	-0.321538	-0.591988
DVU2439	207932	1.15847	2.21161
DVU2440	207933	-0.159637	-0.24128
DVU2441	207934	1.57668	2.98462
DVU2442	207935	1.24969	2.1579
DVU2443	207936	-0.306117	-0.488365
DVU2444	207937	0.229603	0.362208
VIMSS207938	207938	-0.0771412	-0.127049
DVU2446	207939	0.989332	1.912
DVU2447	207940	0.356783	0.621833
DVU2448	207941	-0.555956	-0.955869
DVU2449	207942	-0.995462	-1.81108
DVU2450	207943	0.308849	0.535221
VIMSS207945	207945	-1.16876	-1.62193
DVU2454	207946	0.352881	0.554636
VIMSS207947	207947	0.744293	1.01606
DVU2455	207948	1.14709	2.16176
VIMSS207951	207951	0.90033	0.958051
VIMSS207952	207952	1.25993	2.066
DVU2459	207953	1.43225	2.64217
DVU2460	207954	0.588979	1.06247
DVU2461	207955	0.26629	0.496939
DVU2462	207956	-0.383159	-0.600731
DVU2463	207957	-0.604712	-1.08921
DVU2464	207958	-0.324833	-0.598387
VIMSS207959	207959	1.24199	1.85965
DVU2466	207960	-0.4459	-0.679437
DVU2467	207961	0.511476	0.956763
VIMSS207962	207962	0.918363	1.34222
DVU2468	207963	-0.29772	-0.53175
DVU2470	207964	0.410601	0.753909
DVU2471	207966	-0.108795	-0.200354
DVU2472	207967	0.850693	1.47704
DVU2473	207968	0.413187	0.689083
DVU2474	207969	0.00214006	0.00387235
DVU2475	207970	0.226323	0.427828
DVU2476	207971	1.33333	2.42872
DVU2477	207972	0.294559	0.457621
DVU2478	207973	0.671379	1.02478
DVU2479	207974	-0.104151	-0.154498
VIMSS207975	207975	0.947535	1.32775
DVU2481	207976	1.30175	2.39429
VIMSS207977	207977	0.774801	1.23489
DVU2482	207978	0.963914	1.72734
DVU2483	207980	0.801096	1.32372
DVU2484	207981	1.34301	2.49459
DVU2485	207982	1.17672	2.11686
DVU2486	207983	1.00927	1.76002
DVU2487	207984	0.570433	1.03479
VIMSS207985	207985	-0.250129	-0.402355
DVU2489	207986	0.63276	1.03231
DVU2490	207987	0.0546903	0.100189
DVU2491	207988	-0.345272	-0.647403
DVU2492	207989	-0.541568	-0.862438
DVU2493	207990	-1.18372	-2.05256
DVU2494	207991	0.682859	1.25794
DVU2495	207992	0.730063	1.36782
DVU2496	207993	-0.22401	-0.429827
DVU2497	207994	1.23426	2.27635
DVU2498	207995	1.01769	1.8555
DVU2500	207997	-0.64589	-1.20024

DVU2501	207998	-0.981157	-1.71483
DVU2502	207999	-0.64621	-1.06848
DVU2503	208000	-0.814383	-1.47998
DVU2504	208001	-0.990951	-1.7984
DVU2505	208002	-0.865673	-1.45378
DVU2506	208003	-1.2158	-1.88612
DVU2507	208004	-0.61709	-1.03849
DVU2508	208005	-0.489736	-0.964556
DVU2509	208006	-0.143423	-0.270911
DVU2510	208007	-0.20062	-0.305872
DVU2511	208008	0.17179	0.336956
DVU2512	208009	0.174516	0.32425
DVU2513	208010	0.262411	0.497037
DVU2514	208011	0.159581	0.289603
DVU2515	208012	0.501357	0.918673
DVU2516	208013	0.489183	0.93193
DVU2517	208014	0.84366	1.32226
DVU2518	208015	-1.28473	-2.00505
DVU2519	208016	-1.08732	-1.69254
VIMSS208017	208017	-0.771098	-1.30626
DVU2521	208018	-0.0846741	-0.150362
DVU2522	208019	-0.546559	-1.06547
DVU2523	208020	0.718936	1.35616
DVU2524	208021	1.1953	1.84084
DVU2525	208022	0.294887	0.492056
DVU2526	208023	0.600402	0.987523
DVU2527	208024	-0.295818	-0.449891
DVU2528	208025	-0.1074	-0.138974
DVU2529	208026	0.628261	0.984903
DVU2530	208027	-0.683336	-1.08745
DVU2531	208028	-0.00188016	-0.00301466
DVU2532	208029	0.312536	0.599561
DVU2533	208030	-0.541783	-0.956243
DVU2534	208031	-0.725192	-1.3839
DVU2535	208032	-0.695344	-1.16059
DVU2536	208033	-0.469691	-0.829143
DVU2537	208034	-0.307231	-0.551597
DVU2538	208035	-0.409163	-0.603396
DVU2540	208037	0.159077	0.234089
DVU2541	208038	0.202608	0.32355
VIMSS208039	208039	0.218537	0.2121
DVU2543	208040	2.27167	3.32877
DVU2544	208041	0.813166	1.4708
DVU2545	208042	0.305944	0.569552
DVU2546	208043	0.38562	0.694738
DVU2547	208044	0.819675	1.52752
DVU2548	208045	1.26056	2.28734
DVU2549	208046	0.0553295	0
DVU2551	208048	0.953666	1.82655
DVU2552	208049	-1.33837	-2.46383
DVU2553	208050	-0.676526	-1.22857
DVU2554	208051	-0.259597	-0.491908
DVU2555	208052	-1.32942	-2.5031
DVU2556	208053	1.31549	2.44216
DVU2557	208054	0.435022	0.739228
DVU2558	208055	-1.28272	-2.07328
DVU2559	208056	-0.101053	-0.136042
DVU2560	208057	-0.409962	-0.561951
DVU2561	208058	0.343645	0.504705
DVU2562	208059	-0.541511	-0.903775
DVU2563	208060	0.0560499	0.0683996
DVU2564	208061	1.5232	2.15125
DVU2565	208062	-0.494666	-0.664828
DVU2566	208063	-1.03569	-1.63957
DVU2567	208064	-0.785498	-1.14139
DVU2568	208065	-0.336616	-0.616991
DVU2569	208066	0.0612214	0.112557
DVU2570	208067	0.155015	0.280854
DVU2571	208068	0.67314	1.06321
DVU2572	208069	1.39688	1.93273
DVU2573	208070	2.30313	2.5652
DVU2574	208071	1.8475	2.13152

DVU2575	208072	-0.413608	-0.748323
DVU2576	208073	-0.120913	-0.211226
DVU2577	208074	0.326747	0.518805
DVU2578	208075	1.06322	0
DVU2579	208077	-0.178758	-0.321436
DVU2580	208078	-0.515161	-0.852065
DVU2581	208079	-0.348156	-0.616935
DVU2582	208080	-0.760517	-1.18293
DVU2583	208081	0.565851	0
DVU2584	208082	-0.762427	-1.09677
DVU2585	208083	1.31971	2.49979
DVU2586	208085	-1.05275	-1.59023
DVU2587	208086	0.179694	0.314458
DVU2588	208087	0.799872	1.39543
VIMSS208088	208088	0.0105295	0.0150832
DVU2590	208089	0.43995	0.82441
DVU2591	208090	-0.282796	-0.529251
DVU2592	208091	-0.0867549	-0.108828
VIMSS208092	208092	-0.0788362	-0.13674
VIMSS208093	208093	-0.0305945	-0.0536017
DVU2595	208094	0.514338	0.573024
VIMSS208095	208095	0.644177	0.803738
DVU2596	208096	0.874005	1.01696
DVU2598	208097	0.264623	0
DVU2600	208099	0.59274	0
DVU2603	208102	0.522112	0.755575
DVU2604	208103	-0.176335	0
DVU2605	208104	0.516779	0.883361
DVU2606	208105	0.196664	0
DVU2607	208106	0.128995	0.252268
DVU2609	208108	0.239146	0.411375
DVU2610	208109	-0.598623	0
DVU2612	208111	-0.459438	-0.857718
DVU2613	208112	-0.0954266	-0.177114
DVU2615	208114	1.76688	3.32736
DVU2616	208115	0.629921	0
DVU2617	208116	-0.500168	-0.940607
DVU2619	208118	-0.272721	-0.43069
DVU2620	208119	-0.556729	0
DVU2621	208120	-0.905035	-1.58265
DVU2622	208121	1.23947	0
DVU2623	208122	0.38282	0.563606
DVU2624	208123	0.275952	0.359855
DVU2625	208124	0.340923	0.575642
DVU2626	208125	1.83836	2.70532
DVU2628	208127	-0.103572	0
DVU2629	208128	0.504364	0.804605
DVU2630	208129	0.612071	0
DVU2631	208130	0.767662	1.15573
VIMSS208131	208131	0.321505	0.418487
DVU2633	208132	-0.948352	-1.53545
DVU2634	208133	0.284963	0.514513
DVU2635	208134	0.441041	0.783211
DVU2638	208137	-0.164236	-0.275596
DVU2639	208138	-0.39181	-0.7139
DVU2641	208140	0.0820593	0.151352
DVU2643	208142	0.506923	0.884849
DVU2644	208143	0.195024	0.28104
DVU2645	208144	0.232396	0.341871
DVU2646	208145	0.1896	0
DVU2647	208146	0.986162	1.42094
DVU2649	208148	-0.0897852	-0.117527
DVU2651	208150	0.469231	0.388647
DVU2652	208151	-0.594727	-1.09595
VIMSS208152	208152	-0.544562	-0.838523
VIMSS208153	208153	-0.191968	-0.23292
DVU2655	208154	0.736259	1.31776
VIMSS208155	208155	-0.341015	0
DVU2657	208156	1.16008	2.14738
DVU2658	208157	0.455541	0
DVU2659	208158	-0.180785	-0.328835
VIMSS208159	208159	-0.669647	0

DVU2661	208160	0.579139	1.06153
DVU2663	208162	1.31207	1.85223
DVU2665	208164	1.08851	1.65089
DVU2666	208165	2.18659	0
DVU2667	208166	0.664722	0.873182
DVU2668	208167	0.364056	0
DVU2669	208168	0.286372	0.497932
DVU2670	208169	0.26313	0.483227
DVU2671	208170	-0.992627	-1.91421
DVU2673	208172	0.0194126	0.0331058
DVU2674	208173	-1.00941	-1.79523
DVU2675	208174	2.19048	3.93804
DVU2676	208175	1.53584	2.89113
DVU2677	208176	-0.259338	-0.472588
DVU2678	208177	-0.574322	-1.03886
DVU2679	208178	0.590893	0.983963
DVU2680	208179	0.569731	0.903307
VIMSS208180	208180	0.74334	1.14356
DVU2682	208181	-1.1575	0
DVU2683	208183	-1.31982	-2.31724
DVU2684	208184	1.32184	0
VIMSS208185	208185	1.04583	1.57084
DVU2686	208186	0.419648	0
DVU2687	208188	1.47615	2.59908
DVU2688	208189	0.296423	0
DVU2689	208190	0.731199	1.10038
DVU2690	208191	1.1906	0
DVU2691	208192	0.985893	1.45213
DVU2693	208194	-0.153554	0
DVU2694	208195	-0.102459	-0.144944
DVU2696	208197	0.616608	1.06979
DVU2698	208199	0.588111	0.632376
DVU2700	208201	-0.186979	-0.311005
DVU2701	208202	0.0292502	0.0464424
DVU2702	208203	0.175036	0.314243
DVU2703	208204	0.472269	0.637054
DVU2704	208205	0.844835	0
DVU2705	208206	-0.109987	-0.173378
DVU2706	208207	1.97912	0
DVU2707	208208	-0.52716	0
DVU2708	208209	0.65398	1.00875
VIMSS208210	208210	-1.57433	0
DVU2712	208215	0.192973	0.366324
DVU2714	208217	-0.0715776	-0.100364
DVU2715	208218	0.81165	0
DVU2716	208219	-0.257685	-0.352642
DVU2717	208220	-0.74287	0
VIMSS208221	208221	1.09634	1.5445
DVU2719	208222	0.172816	0
DVU2720	208223	0.574127	0.890274
DVU2721	208225	0.724339	1.20892
DVU2722	208226	0.260986	0.398524
DVU2723	208227	0.00945777	0.0164799
DVU2724	208228	1.36447	1.53264
VIMSS208231	208231	-0.50075	-0.892709
DVU2727	208232	-0.637713	-0.996312
DVU2728	208233	-0.192549	-0.33909
DVU2729	208234	0.497553	0.616687
DVU2730	208235	-0.256067	-0.446129
DVU2731	208236	-0.112878	-0.182467
DVU2732	208237	0.0222455	0
DVU2733	208238	-0.130444	-0.166717
VIMSS208240	208240	0.624476	1.18586
DVU2735	208241	0.0295774	0
DVU2736	208242	0.400108	0
DVU2737	208243	-0.176948	0
DVU2738	208244	0.177384	0.33261
DVU2739	208245	1.03572	0
DVU2741	208247	0.537627	0
DVU2742	208248	0.306378	0.452175
DVU2743	208249	-0.257179	0
DVU2744	208250	0.472152	0.853305

VIMSS208251	208251	-1.39962	0
DVU2746	208252	0.689399	1.18624
DVU2747	208253	0.408958	0.782284
DVU2748	208254	-0.92293	-1.63637
DVU2749	208255	-0.273716	0
DVU2750	208256	-0.897579	0
DVU2751	208257	-0.0941322	-0.143646
DVU2752	208258	0.0022549	0.00414201
DVU2753	208259	0.388534	0.638619
DVU2755	208261	0.613483	0
DVU2756	208262	-0.165264	-0.266577
DVU2757	208263	0.0981697	0
DVU2758	208264	0.863998	0
VIMSS208265	208265	-0.0647133	0
DVU2760	208266	0.654369	0.951502
DVU2761	208267	0.89078	1.46949
DVU2762	208268	0.376046	0.664997
DVU2764	208269	0.0624022	0
DVU2763	208270	0.235361	0.419555
DVU2765	208271	0.279703	0
DVU2766	208272	-0.552824	-0.892619
DVU2767	208273	0.441647	0
DVU2768	208274	0.144063	0.221825
DVU2769	208275	0.67091	1.19366
DVU2771	208277	0.235714	0
DVU2772	208278	-0.0889344	-0.158596
DVU2773	208279	0.159161	0
DVU2774	208280	0.53809	0
DVU2775	208281	0.0551512	0
DVU2776	208282	-1.09483	-1.72164
DVU2779	208285	-0.051076	-0.099144
DVU2780	208286	-0.0975857	-0.152558
DVU2781	208287	0.957664	0
VIMSS208288	208288	0.750713	1.22935
DVU2783	208289	-0.0673654	0
DVU2784	208290	-0.0782858	0
DVU2787	208293	0.500391	0
DVU2788	208294	0.827786	1.227
DVU2789	208295	0.798743	0
DVU2790	208296	0.721332	1.10988
DVU2792	208298	1.31676	2.23275
DVU2793	208299	1.53497	0
DVU2794	208300	1.34701	2.45381
DVU2795	208301	0.894722	0
DVU2796	208302	1.3547	2.37701
DVU2797	208303	1.01192	0
DVU2798	208304	1.53489	3.00994
DVU2799	208305	-1.10795	0
DVU2800	208306	-0.913705	-1.61922
DVU2801	208309	-0.274336	0
DVU2802	208310	0.190842	0
DVU2805	208313	-0.91805	-1.11251
DVU2806	208314	-1.21713	-1.6419
DVU2807	208315	-1.92797	-2.01046
DVU2809	208317	-0.17704	0
DVU2810	208318	-0.523921	-0.645055
DVU2811	208319	0.51903	0.696887
DVU2812	208320	0.3861	0.506451
VIMSS208322	208322	2.2557	0
VIMSS208323	208323	0.323945	0.419833
DVU2815	208324	-0.0372718	-0.0570291
DVU2816	208325	0.125149	0.211094
DVU2817	208327	0.385451	0.612657
DVU2819	208328	-0.0418458	0
DVU2821	208330	-0.659895	-0.928116
DVU2822	208331	-0.61972	-1.07777
DVU2824	208333	-0.910476	-1.46425
DVU2825	208334	0.0789957	0.138439
VIMSS208335	208335	0.301473	0.447674
DVU2827	208336	0.657546	0
DVU2829	208338	-0.588652	0
DVU2830	208339	0.152953	0.247636

DVU2831	208340	0.19451	0.345527
DVU2832	208341	0.256009	0.402528
DVU2833	208342	0.854936	0
VIMSS208343	208343	-0.266591	-0.426476
DVU2835	208344	-0.402419	-0.700366
DVU2836	208345	0.113313	0.199083
DVU2837	208346	-0.522373	0
DVU2838	208347	-1.90171	-3.47029
DVU2839	208348	-0.194584	0
DVU2842	208351	-0.195426	-0.37045
DVU2844	208353	-0.111511	-0.190309
DVU2846	208355	0.617569	1.02671
DVU2847	208356	-0.0896082	-0.131582
DVU2849	208358	0.0775694	0.118565
DVU2850	208359	-0.722801	-1.05625
DVU2851	208360	-0.737032	-1.22804
DVU2852	208361	-0.535149	-0.874012
DVU2853	208362	-0.724563	-1.17987
DVU2854	208363	0.200187	0.307761
DVU2855	208364	0.21333	0.31566
DVU2858	208366	-1.38034	-2.35443
DVU2857	208367	-0.979384	-1.06294
DVU2859	208368	-1.43722	-1.86353
DVU2860	208369	0.0463489	0.0640188
DVU2861	208370	-0.0052573	-0.00811938
DVU2862	208371	-0.987207	-1.60605
DVU2863	208372	-1.83529	-2.921
DVU2864	208373	-0.33824	-0.592297
DVU2865	208374	0.711872	0.934941
DVU2866	208375	-1.24566	-2.07357
DVU2867	208376	-1.53654	-2.72674
DVU2868	208377	0.617383	0.893442
DVU2869	208378	-1.22199	-1.81683
DVU2870	208379	-1.45835	-2.23729
DVU2871	208380	-1.02987	-1.7859
DVU2872	208381	-0.864998	0
DVU2873	208382	-0.13468	0
DVU2874	208383	1.15726	0
DVU2875	208384	1.16954	1.24306
DVU2876	208385	-0.122695	-0.225194
DVU2877	208386	0.0515098	0.0680475
DVU2878	208387	-0.0449967	0
DVU2879	208388	1.86134	1.46079
DVU2880	208389	0.196658	0.252985
DVU2881	208390	1.1093	1.32628
DVU2882	208391	-0.551821	-0.944505
DVU2883	208392	-0.0863181	-0.163482
DVU2884	208393	-0.780948	0
DVU2885	208394	-0.15903	-0.251233
DVU2886	208395	-0.781403	-1.39914
DVU2887	208396	-0.241637	-0.401192
DVU2888	208397	-1.16097	0
DVU2889	208398	-0.67485	-1.21072
DVU2890	208399	-0.361813	0
DVU2891	208400	-0.733471	-1.12364
DVU2892	208401	-0.840591	0
DVU2893	208402	0.530962	0.983346
DVU2894	208403	-0.0226674	0
DVU2895	208404	0.344251	0.593199
DVU2896	208405	-0.178744	-0.330282
DVU2897	208406	0.868996	1.63991
DVU2898	208407	0.436184	0.790278
DVU2899	208408	-0.208983	-0.372319
DVU2900	208409	-0.924194	0
DVU2901	208411	-0.877852	-1.32628
DVU2902	208412	-0.298391	0
DVU2903	208413	-0.673374	-1.18199
DVU2905	208415	-0.0812094	-0.120017
DVU2906	208416	0.0451101	0.0666827
DVU2907	208417	1.37741	1.8795
DVU2908	208418	-0.804851	-1.38438
DVU2909	208419	0.0854061	0.131519

DVU2910	208420	-1.05877	-1.77592
DVU2911	208421	0.190838	0.335253
DVU2913	208423	-1.45325	-2.72646
DVU2914	208424	-0.77736	0
DVU2915	208425	1.07322	0
DVU2916	208426	-1.28229	0
DVU2917	208427	-0.71045	-1.16708
DVU2918	208428	0.596523	0.84115
VIMSS208429	208429	1.00827	1.83565
DVU2920	208430	-1.8945	0
DVU2921	208431	-0.816086	-1.38165
DVU2922	208432	-1.85746	-2.63372
DVU2923	208433	-2.15727	-3.41659
DVU2924	208434	-1.31598	-1.82411
DVU2925	208435	-1.35116	-2.23855
DVU2927	208437	-2.06633	-3.28917
DVU2928	208438	0.0780073	0
DVU2929	208439	-0.400823	-0.7665
DVU2931	208441	-0.214611	-0.385677
DVU2932	208442	0.277813	0.469554
DVU2933	208443	-0.324591	-0.571969
DVU2934	208444	-0.0379104	0
DVU2935	208445	0.641225	0
VIMSS208446	208446	0.482359	0.738501
DVU2937	208447	0.705303	1.28899
DVU2938	208448	0.85896	0
DVU2939	208449	-0.0207231	-0.0355886
DVU2940	208450	-0.937312	-1.61646
DVU2941	208451	-0.542416	-0.927357
DVU2942	208452	-0.718446	0
DVU2943	208453	-0.135395	-0.22636
DVU2944	208454	-0.777226	-1.2436
DVU2945	208455	-0.739053	-1.29055
DVU2946	208456	-0.17731	-0.343186
DVU2949	208459	0.329206	0.485987
VIMSS208461	208461	0.950263	1.73975
DVU2951	208462	0.118303	0
DVU2952	208463	0.518231	0.860029
DVU2953	208464	0.000253736	0.000355493
DVU2954	208465	0.00327102	0
VIMSS208466	208466	0.0296761	0
DVU2956	208467	0.474342	0.866419
VIMSS208468	208468	1.28866	0
DVU2957	208470	0.297615	0.274423
DVU2958	208471	0.658395	0.909662
DVU2959	208472	-0.217074	-0.326533
DVU2961	208474	0.709441	1.12996
DVU2962	208475	0.49303	0
DVU2963	208476	-0.196177	-0.325022
DVU2964	208477	0.745121	1.07882
DVU2965	208478	0.990562	1.65436
DVU2966	208479	0.697796	0
DVU2967	208480	-0.270157	-0.473166
DVU2969	208482	0.881595	1.47158
DVU2970	208483	0.963287	0
DVU2971	208484	-0.0641641	-0.116447
DVU2973	208486	1.87932	3.68583
DVU2974	208487	1.10923	1.9643
DVU2975	208488	1.59501	2.41286
DVU2976	208489	0.857648	0
VIMSS208490	208490	1.04786	1.89529
DVU2979	208492	-0.706744	-1.28762
DVU2981	208494	-0.256741	0
DVU2982	208495	-0.218564	-0.3409
DVU2983	208496	-0.570746	-1.01495
DVU2984	208497	0.117959	0
DVU2985	208498	-0.159962	-0.305447
DVU2986	208499	0.580623	0
DVU2987	208500	0.413283	0.625323
DVU2988	208501	0.862826	1.46949
DVU2989	208502	0.0806952	0.141773
DVU2990	208503	0.600604	1.11596

DVU2991	208504	0.295623	0
DVU2992	208505	0.146105	0.261881
DVU2993	208506	-0.263696	-0.47893
DVU2995	208508	-0.117179	-0.216472
DVU2996	208509	-0.276021	0
DVU2997	208510	-0.530701	-0.788561
DVU2999	208512	-0.0475255	-0.0842833
DVU3001	208514	0.0952525	0.162316
DVU3002	208515	0.157436	0
DVU3003	208516	0.199658	0.368614
DVU3004	208517	0.0668133	0.108789
DVU3005	208518	-0.473539	-0.871383
DVU3006	208519	-0.718707	0
DVU3007	208520	0.368073	0.497277
DVU3008	208521	-0.727445	-1.30945
DVU3009	208522	0.158239	0.277674
DVU3010	208523	0.0794073	0.122825
DVU3011	208524	-0.720833	-1.316
DVU3012	208525	0.386741	0.652366
DVU3013	208526	-0.163545	-0.277993
DVU3014	208527	-0.00698292	-0.0116652
DVU3015	208528	-0.169253	-0.285528
DVU3016	208529	-0.508227	-0.912121
DVU3017	208530	0.136737	0.249248
DVU3018	208531	0.351495	0.661145
DVU3019	208532	0.240375	0.436046
DVU3020	208533	0.415112	0
DVU3021	208534	-0.0716023	-0.125372
VIMSS208535	208535	-1.08825	-1.99576
DVU3022	208536	-0.174719	-0.321984
DVU3023	208537	-0.728068	-1.13536
DVU3024	208538	-2.29613	-2.93171
DVU3025	208539	-2.8855	-3.89265
DVU3026	208540	-0.703238	-1.06009
DVU3027	208541	-2.74789	-3.86358
DVU3028	208542	-1.16344	-2.10364
DVU3030	208544	-2.83577	-4.48692
DVU3031	208545	-2.588	-3.66786
DVU3032	208546	-2.76675	-4.60607
DVU3033	208547	-2.26771	-3.59327
VIMSS208549	208549	0.739596	1.13537
DVU3035	208550	-0.741201	-1.32637
DVU3036	208551	0.713693	1.1954
DVU3037	208552	0.653822	1.16521
VIMSS208553	208553	0.565919	0.860514
DVU3039	208554	0.0676616	0.119992
VIMSS208555	208555	0.260628	0.404065
DVU3041	208556	0.650205	1.21206
DVU3042	208557	0.959546	1.76954
VIMSS208559	208559	0.87108	0
DVU3046	208561	-0.312092	-0.560527
DVU3048	208563	-0.132677	-0.199514
DVU3049	208564	0.145007	0.274462
DVU3050	208565	-0.414057	-0.637086
DVU3051	208566	-0.676346	-1.04472
DVU3052	208567	-0.129028	0
DVU3053	208568	-0.838915	-1.5399
DVU3054	208569	-0.104637	-0.173007
DVU3055	208570	-0.899387	-1.16917
DVU3056	208571	-1.19275	-2.17772
DVU3057	208572	0.796817	1.35636
DVU3058	208573	-0.0141826	-0.0222497
DVU3059	208574	-0.446427	-0.792234
VIMSS208575	208575	-0.111566	-0.194412
DVU3061	208576	-0.336094	-0.559816
DVU3062	208577	1.0654	1.86389
DVU3063	208578	0.882303	1.13956
DVU3064	208579	0.087009	0.141934
DVU3065	208580	-0.435661	0
DVU3066	208581	-0.212564	0
DVU3067	208582	0.950471	1.74166
DVU3068	208584	1.31247	2.37432

DVU3070	208586	-1.71357	-2.72693
DVU3071	208587	-1.24932	-2.3391
DVU3072	208588	0.0667645	0.100706
DVU3074	208590	-0.69047	-1.08222
VIMSS208591	208591	-0.0213919	-0.0252748
DVU3076	208592	1.58762	2.99734
DVU3077	208593	1.99107	3.74498
VIMSS208594	208594	0.432346	0
DVU3079	208595	0.722415	1.21271
DVU3080	208596	0.128611	0.225891
DVU3081	208597	-0.660003	-1.20321
DVU3082	208598	-0.331834	-0.612702
VIMSS208599	208599	0.0731582	0.11222
DVU3084	208600	-0.424529	-0.792639
DVU3085	208601	0.213595	0.395905
DVU3086	208602	-0.977796	-1.40219
DVU3087	208603	-0.0233187	-0.0324707
DVU3088	208604	-0.377626	-0.674968
DVU3089	208605	-0.435339	-0.785362
DVU3090	208606	-1.50813	-2.82808
DVU3091	208607	0.425163	0.770401
VIMSS208608	208608	0.0466656	0.0791409
DVU3092	208609	-0.222937	-0.349613
DVU3093	208610	0.849633	1.38249
DVU3094	208611	-0.696758	-1.26196
DVU3095	208612	0.145935	0.228974
VIMSS208613	208613	0.0407567	0.0690628
DVU3097	208614	0.557349	1.07749
DVU3098	208615	0.545824	0.843059
DVU3099	208616	-0.823767	0
DVU3100	208617	-0.244893	-0.431714
DVU3101	208618	-0.743576	0
DVU3102	208619	-0.449817	-0.63352
DVU3103	208620	-1.91874	0
DVU3104	208621	-0.854375	-1.54237
VIMSS208623	208623	0.676335	0.463438
DVU3106	208624	0.111986	0.202467
DVU3107	208625	0.893026	1.50516
DVU3108	208626	-0.140228	-0.232206
DVU3109	208627	-0.212866	-0.341336
DVU3110	208628	0.249606	0.343736
DVU3111	208629	-0.115055	-0.195357
DVU3112	208630	0.499887	0
DVU3113	208631	-0.668016	-1.29867
VIMSS208633	208633	-0.229519	-0.304162
DVU3119	208637	-0.390221	-0.710431
VIMSS208638	208638	0.25096	0
DVU3121	208639	-0.508455	-0.785976
DVU3122	208640	1.12476	1.13658
DVU3123	208641	0.0790533	0.103438
DVU3125	208643	0.57447	1.12169
VIMSS208644	208644	0.0817692	0.149684
DVU3126	208645	0.550197	0.827115
DVU3127	208646	0.56478	0
DVU3128	208647	0.234996	0.424217
DVU3129	208648	0.776212	0
DVU3130	208649	0.567834	0.652596
DVU3131	208650	2.16354	0
DVU3132	208651	1.54994	2.53637
DVU3133	208652	3.22439	5.30956
DVU3134	208653	2.38222	3.69766
DVU3136	208655	0.951804	1.76957
VIMSS208657	208657	-0.229607	-0.337847
DVU3140	208659	-0.355174	-0.649867
DVU3142	208661	0.891162	1.49704
DVU3143	208662	0.545518	0.710254
DVU3144	208663	1.46205	2.15841
DVU3145	208664	2.01021	2.51437
DVU3146	208665	0.0852757	0.151479
DVU3148	208667	0.408015	0.66716
DVU3149	208668	0.012354	0.0223053
DVU3150	208669	-0.443806	-0.834493

DVU3151	208670	0.126681	0
DVU3153	208672	0.452557	0
DVU3154	208673	-0.396076	-0.684215
DVU3155	208674	0.872818	1.53517
DVU3157	208676	-0.0185143	-0.0328201
DVU3158	208677	0.0794029	0.145643
VIMSS208680	208680	0.0945032	0.154552
DVU3161	208681	-1.47658	0
DVU3163	208683	0.357325	0
DVU3164	208684	-0.0729134	-0.139399
DVU3165	208685	-0.12247	0
DVU3166	208686	0.27627	0.405632
DVU3168	208688	-1.38978	-2.59394
DVU3169	208689	0.247545	0.419723
VIMSS208690	208690	-0.540147	-0.756807
DVU3170	208691	0.51844	0.696183
DVU3172	208693	0.202358	0.358588
DVU3173	208694	-0.152481	-0.302804
DVU3174	208695	-1.21117	-2.34941
DVU3175	208696	0.120142	0
DVU3176	208697	-0.340507	-0.626728
DVU3177	208698	-0.102958	-0.19924
DVU3178	208699	-1.11222	-2.04411
DVU3179	208700	-0.233432	0
DVU3180	208701	0.180641	0.321212
DVU3181	208702	-0.526472	0
DVU3182	208703	0.832377	1.5501
DVU3184	208705	-0.0861705	-0.155073
DVU3186	208707	-0.825961	-1.49795
DVU3187	208708	0.617363	0
DVU3188	208709	-0.0375485	-0.0547536
DVU3189	208710	0.163869	0
DVU3190	208711	-0.921218	-1.61682
DVU3191	208712	-0.598184	0
DVU3192	208713	-0.784373	-1.41978
DVU3193	208714	-0.253388	0
DVU3194	208715	-0.869789	-1.3077
DVU3195	208716	0.190885	0.316885
DVU3196	208717	0.332904	0.566868
DVU3198	208719	-0.869515	-1.53128
DVU3199	208720	-1.10704	-1.78451
DVU3200	208721	-1.13943	-2.11898
DVU3201	208722	-0.0819427	0
DVU3202	208723	-0.587681	-0.923848
DVU3203	208724	-0.331935	-0.627643
DVU3204	208725	-0.75514	-1.37074
DVU3205	208726	-0.0951306	-0.161166
DVU3206	208728	-1.0109	-1.87986
DVU3207	208729	0.178062	0.32952
DVU3208	208730	-0.577995	-1.07759
VIMSS208731	208731	0.590137	0
DVU3210	208732	-0.681442	-1.19229
VIMSS208733	208733	1.25575	1.42165
DVU3212	208734	-2.00542	0
DVU3213	208735	0.512503	0.910902
DVU3214	208736	0.541434	0.893882
DVU3215	208737	0.433558	0.830524
DVU3216	208738	0.4053	0.778224
DVU3217	208739	1.00727	1.96106
DVU3218	208740	0.46834	0.910205
DVU3219	208741	0.545273	1.05451
DVU3220	208742	0.0323151	0.0578733
DVU3221	208743	-0.148143	-0.285545
DVU3222	208744	0.492343	0.931786
DVU3223	208745	0.0175794	0.0277867
DVU3224	208746	-0.406306	-0.614201
DVU3225	208747	0.260994	0.477274
DVU3226	208748	-0.258017	-0.475377
DVU3228	208750	0.652768	1.1294
DVU3230	208752	0.372888	0
DVU3232	208754	-0.560483	0
DVU3233	208755	-0.360606	-0.616253

DVU3234	208757	-0.291801	-0.485722
DVU3236	208759	-0.498024	-0.724461
DVU3238	208761	-0.312406	-0.55425
DVU3239	208762	-1.1955	0
DVU3240	208763	0.275122	0.463828
DVU3241	208764	-0.115704	-0.164164
DVU3242	208765	-0.665483	-1.23579
DVU3243	208766	-0.647371	-0.987014
VIMSS208767	208767	-0.0530653	-0.0952936
DVU3245	208768	-0.699579	0
DVU3246	208769	0.984864	1.82296
DVU3247	208771	0.698187	1.17886
DVU3248	208772	0.819539	1.32243
DVU3249	208773	0.493065	0.809724
DVU3250	208774	-0.0271732	-0.0499566
DVU3251	208775	1.06872	1.6171
DVU3252	208776	0.0817759	0.144924
DVU3253	208777	-0.365838	-0.659552
DVU3254	208778	0.508202	0
DVU3255	208779	-0.957822	-1.57269
DVU3256	208780	-0.0722666	-0.12012
DVU3258	208782	-0.219904	-0.337693
DVU3259	208783	0.172055	0
DVU3260	208784	0.975591	1.06469
DVU3261	208785	0.762588	1.34848
DVU3262	208786	0.16893	0.303106
DVU3263	208787	0.79302	1.53152
DVU3264	208788	0.17944	0.313956
DVU3266	208790	0.755581	0
DVU3267	208791	-0.236875	-0.436788
DVU3268	208792	-0.123515	-0.203103
DVU3269	208793	0.542975	0.924028
DVU3270	208794	0.0484199	0.0725008
DVU3271	208795	-0.0385763	-0.0700463
DVU3272	208796	-0.657661	-1.20796
DVU3273	208797	-0.87913	-1.27983
DVU3274	208798	-1.3517	-2.48949
DVU3275	208799	-0.803603	-1.503
DVU3276	208800	-0.766161	-1.17644
DVU3278	208802	0.617727	1.03826
DVU3279	208803	-0.196254	-0.349024
VIMSS208804	208804	-0.62255	-1.04154
DVU3281	208806	-0.734226	-1.31017
DVU3282	208807	0.596646	1.09267
DVU3283	208809	-0.381326	-0.690044
DVU3284	208810	0.526516	0.820943
DVU3285	208811	0.855411	1.05214
DVU3287	208813	-1.10011	0
VIMSS208814	208814	-0.386303	-0.378756
VIMSS208815	208815	0.4318	0.576273
DVU3289	208816	-0.194444	-0.327947
DVU3290	208817	0.232824	0.395918
DVU3291	208818	0.439418	0.701513
DVU3292	208819	-0.246135	-0.402757
DVU3293	208820	0.352952	0.632377
DVU3294	208821	-0.0757274	-0.125934
DVU3295	208822	1.06815	1.64905
DVU3296	208823	0.707784	1.28932
DVU3297	208824	-1.32049	-2.09216
DVU3298	208825	-0.129101	-0.214335
DVU3299	208826	-0.117719	-0.209273
DVU3300	208827	0.741528	0
DVU3301	208828	0.825025	1.18663
DVU3303	208830	0.715895	1.04236
VIMSS208831	208831	0.583091	0
DVU3305	208832	-0.0794543	-0.124067
DVU3306	208833	-0.109854	0
DVU3307	208834	-1.89223	-3.32283
DVU3308	208835	-1.49081	0
DVU3310	208836	-2.13374	-3.28062
DVU3311	208837	-0.374578	0
DVU3312	208838	0.610735	0.802134

DVU3313	208839	-0.199525	-0.301515
DVU3314	208840	0.68627	0.943796
DVU3315	208841	-0.426407	0
DVU3316	208842	-0.34392	-0.61092
VIMSS208843	208843	-0.0359796	-0.054309
DVU3318	208844	-0.154583	-0.248377
DVU3319	208845	0.552677	0
DVU3320	208846	-0.328771	-0.551717
DVU3322	208848	0.0732009	0.115508
DVU3323	208849	-0.0848674	0
DVU3324	208850	-0.091296	-0.0966905
DVU3325	208851	-0.277114	-0.437234
DVU3326	208852	0.188791	0.286951
VIMSS208854	208854	-0.974553	-1.69404
DVU3329	208855	0.203216	0.342448
DVU3330	208856	-0.236181	-0.427857
DVU3331	208857	0.359917	0.561168
DVU3332	208858	0.0148882	0.0246881
DVU3334	208860	-0.165238	-0.269409
DVU3335	208861	-0.117822	-0.224275
DVU3336	208862	0.314755	0.506687
DVU3337	208863	0.0373518	0
DVU3338	208864	0.578059	0.902816
VIMSS208866	208866	1.10033	1.55033
VIMSS208867	208867	-0.0394229	-0.0634971
DVU3342	208868	0.521649	0.952414
DVU3343	208869	0.186182	0
DVU3344	208870	0.949828	1.41378
VIMSS208873	208873	1.372	1.72184
DVU3347	208874	-1.47446	-2.58546
DVU3348	208875	-1.4997	-2.42434
DVU3349	208876	-1.16687	0
DVU3350	208877	-0.994366	-1.88548
DVU3351	208878	-0.24241	-0.456881
DVU3352	208879	0.646868	1.19603
DVU3355	208882	1.61875	0
DVU3356	208883	-0.0860061	-0.155674
DVU3357	208884	-0.415003	0
DVU3359	208886	0.398852	0.719204
DVU3360	208887	0.23578	0.426657
DVU3361	208888	-0.403606	-0.652084
DVU3363	208890	0.0912994	0.16998
DVU3364	208891	0.78587	1.49086
DVU3365	208892	-0.173159	0
DVU3366	208893	-0.463841	-0.79132
DVU3367	208894	-1.68314	-2.59933
DVU3368	208895	-1.03305	-1.72053
DVU3369	208896	0.267892	0
VIMSS208897	208897	0.797849	1.2515
DVU3371	208898	0.387034	0.620556
DVU3372	208899	0.680711	0.915868
DVU3373	208900	-0.531129	-0.879936
DVU3374	208901	0.197068	0.342571
DVU3376	208904	0.475134	0.753138
DVU3380	208908	0.583345	0.599904
DVU3381	208909	0.448343	0.789066
DVU3382	208910	0.336157	0.544711
VIMSS208911	208911	0.731296	0.53397
DVU3384	208912	0.649218	1.07651
VIMSS208913	208913	-0.0107123	-0.0157469
DVU3386	208914	-0.46256	-0.757466
DVU3387	208915	0.868308	1.38582
DVU3388	208916	0.264581	0.464659
DVU3389	208917	-0.773141	-1.415
VIMSS208918	208918	-0.0318804	-0.0541832
VIMSS208919	208919	-0.232713	-0.269555
VIMSS208920	208920	-0.626492	-1.10281
DVU3392	208921	0.808232	1.37028
DVU3393	208922	0.137271	0.232621
DVU3394	208923	0.626465	0.697216
DVU3395	208924	-1.34027	-2.42724
VIMSS208925	208925	-0.240774	-0.433156

DVU0001	208926	-0.51345	-0.927761
VIMSS208927	208927	-0.613687	-1.08301
DVU0002	208928	-0.312468	-0.520794
DVU0003	208929	-0.212177	-0.403086
VIMSS208930	208930	-0.387327	-0.662839
DVU0004	208931	-0.989554	-1.66678
DVU0005	208932	-0.273117	-0.434694
DVU0006	208933	1.19033	2.13413
DVU0007	208934	-0.0527053	-0.0912681
DVU0008	208935	1.10977	1.30263
DVU0009	208936	-1.07897	-1.80255
DVU0010	208937	-0.158517	-0.21147
DVU0011	208938	0.219552	0.399895
DVU0012	208939	-0.32015	-0.601011
DVU0013	208940	-0.18339	-0.296512
DVU0014	208941	-2.5462	-3.92437
DVU0015	208942	0.0435523	0.0737281
DVU0016	208943	0.821415	0.996288
VIMSS208944	208944	-0.0371095	-0.0539102
DVU0018	208945	0.4619	0.800706
DVU0019	208946	2.18509	3.40518
VIMSS208947	208947	0.946721	1.0278
DVU0020	208948	-0.131974	-0.21709
DVU0021	208949	-0.134176	-0.251336
DVU0022	208950	0.313246	0.452765
VIMSS208951	208951	0.193509	0.287068
DVU0024	208952	-1.39948	-1.94721
DVU0025	208953	0.0104439	0.0173963
DVU0026	208954	-0.60746	-1.08744
DVU0027	208955	-0.459246	-0.860686
DVU0028	208956	0.682906	1.28453
DVU0029	208957	-0.103442	-0.180839
DVU0031	208959	0.212187	0.346272
DVU0032	208960	0.55745	0.996912
DVU0033	208961	0.0471218	0.0914735
DVU0034	208962	-0.331759	-0.620961
DVU0035	208963	-0.385012	-0.633898
DVU0036	208964	-1.72318	-2.57487
DVU0037	208965	0.246506	0.448289
DVU0038	208966	-0.393518	-0.647294
DVU0039	208967	-0.161813	-0.20476
DVU0040	208968	-0.839336	-1.36971
DVU0041	208969	-0.929814	-1.53097
DVU0042	208970	0.0709214	0.123341
DVU0043	208971	0.93782	1.68252
DVU0044	208972	0.340916	0.581743
DVU0045	208973	0.0813687	0.153265
DVU0046	208974	0.200221	0.3854
DVU0047	208975	-0.297478	-0.519401
DVU0048	208976	0.016429	0.0290253
DVU0049	208977	0.282671	0.531384
DVU0050	208978	0.116975	0.196526
DVU0051	208979	0.278506	0.478965
DVU0052	208980	-0.143544	-0.249052
DVU0053	208981	0.861114	1.52363
DVU0054	208982	-0.658922	-1.07726
DVU0055	208983	-0.258811	-0.393597
DVU0056	208984	0.0943128	0.169425
DVU0057	208985	0.0201004	0.0366993
DVU0058	208986	0.270125	0.48023
DVU0059	208987	-0.349575	-0.585805
DVU0060	208988	-0.178976	-0.310081
DVU0061	208989	-0.881766	-1.60934
DVU0062	208990	-0.343531	-0.653934
DVU0063	208991	-0.241409	-0.460314
DVU0064	208992	-1.40954	-2.30833
DVU0065	208993	-0.146933	-0.222908
DVU0066	208994	-0.241505	-0.428748
DVU0067	208995	-0.073772	-0.134909
DVU0068	208996	0.451334	0.797797
DVU0069	208997	0.302633	0.512515
DVU0070	208998	0.257754	0.279179

DVU0071	208999	-0.278241	-0.439217
DVU0072	209000	-0.334871	-0.584842
DVU0073	209001	-0.0522444	-0.0987465
DVU0074	209002	-0.458945	-0.885264
DVU0075	209003	-0.0980985	-0.169076
DVU0076	209004	-0.235638	-0.445313
DVU0077	209005	0.433975	0.767705
DVU0078	209006	0.73033	1.17701
DVU0079	209007	0.253926	0.452939
DVU0080	209008	-0.0293651	-0.0524351
DVU0081	209009	0.90401	1.50146
DVU0082	209010	0.229788	0.375308
DVU0083	209011	0.395269	0.733113
DVU0084	209012	-0.30633	-0.503355
DVU0085	209013	-1.13846	-2.04397
VIMSS209014	209014	1.19702	1.50197
DVU0087	209016	-0.396615	-0.725929
DVU0088	209017	-0.545421	-0.752465
DVU0089	209018	0.344946	0.520941
DVU0090	209019	-0.118258	-0.20085
DVU0091	209020	-0.0437735	-0.0822636
DVU0092	209021	0.105345	0.195143
DVU0093	209022	-0.467288	-0.752911
DVU0094	209023	-1.16961	-2.02042
DVU0095	209024	-0.720084	-1.2804
DVU0096	209025	-1.07007	-1.4294
DVU0097	209026	-1.36927	-2.51943
DVU0098	209027	-0.36172	-0.640183
DVU0099	209028	-0.288064	-0.507035
DVU0100	209029	-0.154307	-0.16446
DVU0101	209030	-0.504223	-0.76296
DVU0102	209031	-0.352487	-0.403198
DVU0103	209032	-0.860873	-1.1167
DVU0104	209033	-0.24498	-0.376807
DVU0105	209035	-0.39305	-0.697995
DVU0106	209036	-0.323662	-0.618412
DVU0107	209037	-0.273636	-0.527938
DVU0108	209038	0.230014	0.372137
DVU0109	209039	0.66109	1.07275
DVU0110	209040	-0.428567	-0.748014
DVU0112	209042	-0.421489	-0.781801
DVU0113	209043	-1.54461	-2.71771
DVU0114	209044	-1.46626	-2.59528
DVU0115	209045	-0.150052	-0.233442
DVU0116	209046	-0.752415	-1.23008
DVU0117	209047	-1.21172	-1.67393
DVU0118	209048	0.552734	1.03008
DVU0119	209049	-0.216601	-0.374463
DVU0120	209050	-0.329666	-0.580135
DVU0121	209051	-0.0943912	-0.175212
DVU0122	209052	1.62554	2.84946
DVU0123	209053	0.2338	0.347711
DVU0124	209054	-0.245465	-0.400641
DVU0125	209055	0.506043	0.881245
DVU0126	209056	0.0369379	0.0518633
DVU0127	209057	-0.366134	-0.681412
DVU0128	209058	0.147095	0.26516
DVU0129	209059	-0.149483	-0.277056
DVU0130	209060	0.165474	0.274309
VIMSS209062	209062	-1.02154	-1.41035
DVU0132	209063	-1.95486	-3.35016
VIMSS209064	209064	-1.16313	-1.72927
DVU0133	209065	-1.93243	-3.40118
DVU0134	209066	-0.449262	-0.795532
DVU0136	209067	-1.26486	-2.19478
DVU0135	209068	-0.538713	-1.04358
VIMSS209069	209069	0.990369	1.46075
DVU0138	209070	1.57333	3.02588
DVU0139	209071	0.0999821	0.177866
DVU0140	209072	0.497444	0.899598
DVU0141	209073	-0.695188	-1.31178
DVU0142	209074	-0.709346	-1.23269

DVU0143	209075	-0.794931	-1.38842
DVU0144	209076	-0.333312	-0.614676
DVU0145	209077	0.259896	0.473576
DVU0146	209078	-0.0277826	-0.0479559
DVU0147	209079	0.0772874	0.139397
DVU0148	209080	0.250878	0.46437
DVU0149	209081	0.591388	1.12113
DVU0150	209082	-0.33951	-0.545288
DVU0152	209084	0.637771	1.1927
DVU0153	209085	-0.146118	-0.26928
VIMSS209086	209086	-0.813414	-1.03507
DVU0155	209087	0.36128	0.600444
DVU0156	209088	-0.0570041	-0.1095
DVU0157	209089	-0.480641	-0.927727
DVU0158	209090	-0.26454	-0.473245
DVU0159	209091	-0.507538	-0.908519
DVU0160	209092	-0.388267	-0.733778
DVU0161	209093	-0.846374	-1.41046
DVU0162	209094	-0.901497	-1.61817
DVU0163	209096	0.337111	0.562665
DVU0164	209097	0.632407	1.17515
DVU0165	209098	-1.76085	-2.92052
DVU0166	209099	-1.16745	-2.12226
DVU0167	209100	-1.01426	-1.69399
DVU0168	209101	-0.988825	-1.82296
DVU0169	209102	-0.0707804	-0.0950586
DVU0170	209103	2.42638	4.38486
DVU0172	209105	2.21812	4.24333
DVU0173	209106	1.49846	0
DVU0174	209107	1.2096	2.15278
DVU0175	209108	-0.206331	0
DVU0176	209109	-0.15347	-0.286858
DVU0177	209110	0.144592	0.2466
VIMSS209111	209111	-0.410879	-0.652011
DVU0179	209112	-0.16845	-0.327951
DVU0180	209113	-0.264038	-0.410671
DVU0181	209114	0.32168	0.46931
DVU0182	209115	-0.0861832	-0.155623
DVU0183	209116	-0.296012	-0.472113
DVU0184	209117	0.18811	0.302196
DVU0185	209118	0.133495	0.183251
DVU0186	209119	1.62096	2.17233
DVU0187	209120	-1.00392	-1.76253
VIMSS209121	209121	-0.336036	-0.579214
DVU0189	209122	-1.2403	-2.00534
DVU0190	209123	-0.87241	-1.15828
DVU0191	209124	-0.228601	-0.350447
DVU0192	209125	-0.624553	-1.02193
DVU0193	209126	0.239277	0.161829
DVU0194	209127	-0.988932	-1.60707
DVU0195	209128	-0.137728	-0.208717
DVU0196	209129	-0.678425	-1.27386
DVU0197	209130	-0.31647	-0.537614
DVU0198	209131	-1.21417	-1.67423
DVU0199	209132	-1.61287	-2.7132
DVU0200	209133	-1.68102	-2.93428
DVU0201	209134	-1.14159	-1.92706
DVU0202	209135	-1.30078	-2.24459
DVU0203	209136	-1.85294	-2.98276
DVU0204	209137	-1.22716	-1.88474
DVU0205	209138	-0.905095	-1.30508
DVU0206	209139	-0.965022	-1.53051
DVU0207	209140	-0.828622	-1.31578
DVU0208	209141	-1.01069	-1.61795
DVU0209	209142	-1.32368	-2.13491
DVU0210	209143	-0.956818	-1.55633
DVU0211	209144	-0.0726252	-0.116725
DVU0212	209145	-0.307064	-0.504394
DVU0213	209146	0.197909	0.3096
DVU0214	209147	0.0320581	0.0515216
DVU0215	209148	0.203849	0.346351
DVU0216	209149	-0.880691	-1.38623

DVU0217	209150	-1.06223	-1.86197
DVU0218	209151	-0.346065	-0.619425
DVU0219	209152	-1.05748	-1.81312
DVU0220	209153	0.0857369	0.16884
DVU0221	209154	-1.33422	-2.30998
DVU0222	209155	-0.0990636	-0.158524
DVU0223	209156	-0.0938567	0
DVU0224	209157	0.245332	0.441286
VIMSS209158	209158	1.95684	1.85988
DVU0226	209159	-0.353987	-0.612912
DVU0227	209160	-0.498222	-0.939797
VIMSS209161	209161	-0.664158	-1.03295
DVU0230	209163	0.0690306	0.117504
DVU0231	209164	-0.18344	-0.294587
DVU0232	209165	-0.600989	-0.971114
VIMSS209166	209166	2.31438	2.63575
DVU0234	209167	0.241029	0.44611
DVU0235	209168	-0.337077	-0.612876
DVU0236	209169	-0.913576	-1.3434
DVU0237	209170	-0.517032	-0.851379
DVU0238	209171	1.17765	2.20892
VIMSS209172	209172	1.18043	2.06051
DVU0240	209173	0.813205	1.45162
DVU0241	209174	0.347753	0.6494
DVU0242	209175	-0.26309	-0.499622
DVU0243	209176	-0.381903	-0.660139
DVU0244	209177	-0.0873867	-0.165504
DVU0245	209178	-0.156072	-0.256193
DVU0246	209179	0.0968803	0.164585
DVU0247	209180	0.145722	0.276787
VIMSS209181	209181	-0.495088	-0.902435
DVU0249	209182	0.589929	0.944845
DVU0250	209183	1.006	1.91366
DVU0252	209185	-0.722023	-1.35313
DVU0253	209186	0.458244	0.852659
VIMSS209187	209187	0.150797	0.248785
DVU0255	209188	0.254224	0.483751
DVU0256	209189	0.0953388	0.173695
DVU0257	209191	-0.0205682	-0.0324441
DVU0258	209192	-0.445351	-0.784139
DVU0259	209193	0.0731008	0.137331
DVU0260	209194	0.539055	0.954063
DVU0261	209195	0.504562	0
DVU0262	209196	0.0644612	0.107618
DVU0263	209197	0.364027	0.632869
DVU0264	209198	-0.0320729	-0.0599916
DVU0265	209199	0.524149	0.946112
DVU0266	209200	0.327805	0.603876
VIMSS209201	209201	0.923945	1.70832
VIMSS209202	209202	0.715751	1.18033
DVU0269	209203	0.342377	0.659143
DVU0270	209204	-0.148444	-0.275131
DVU0271	209205	0.113312	0.216514
DVU0272	209206	-0.700559	-1.20348
DVU0273	209207	0.348925	0.486981
DVU0274	209208	-1.26161	-2.11044
DVU0275	209209	0.786639	1.49594
DVU0276	209210	1.57992	2.90511
DVU0277	209211	0.586738	1.06554
DVU0278	209212	0.404406	0.620315
DVU0279	209213	-1.45391	-2.77324
DVU0280	209214	-0.958492	-1.70551
DVU0281	209215	0.53306	0.975993
DVU0282	209216	-0.69717	-1.32369
DVU0284	209217	-0.869352	-1.52505
DVU0283	209218	0.205047	0.25142
DVU0285	209219	-1.11345	-2.0873
DVU0286	209220	-0.121597	-0.209316
VIMSS209221	209221	-0.576735	-0.762783
VIMSS209222	209222	-0.98003	-1.62925
DVU0289	209223	-0.0385356	-0.0738151
DVU0290	209224	0.0478652	0.0846586

DVU0291	209225	-0.771174	-1.33494
DVU0293	209226	-0.538243	-0.960308
DVU0292	209227	-0.427883	-0.775806
DVU0294	209228	-0.0100837	-0.0161411
DVU0295	209229	-0.173713	-0.316464
DVU0296	209230	-1.16176	-1.99066
DVU0297	209231	-0.723559	-1.3844
DVU0298	209232	0.635154	1.0368
DVU0299	209233	-0.796004	-1.42018
DVU0300	209234	-0.368881	-0.542445
DVU0302	209236	0.365531	0.642381
DVU0303	209237	0.513581	0.779988
DVU0304	209238	-1.07503	-1.64496
DVU0305	209239	0.580717	0
DVU0306	209240	0.411832	0.757625
DVU0307	209241	-0.453639	-0.57726
DVU0308	209242	-0.657693	-1.24354
DVU0309	209243	-0.796361	-1.1168
DVU0310	209244	0.00460754	0.00802188
DVU0311	209245	-0.512545	-0.909215
DVU0312	209246	-0.0531319	-0.098328
DVU0313	209247	-0.0228267	-0.0413463
DVU0314	209248	0.47402	0.849104
DVU0315	209249	0.12851	0.19041
DVU0316	209250	0.613898	1.00526
VIMSS209251	209251	-0.685869	-0.82631
DVU0318	209252	-0.232291	-0.338398
DVU0319	209253	-0.779393	-1.40123
DVU0320	209254	0.463974	0.51568
DVU0321	209255	-0.000429841	-0.000814821
DVU0322	209256	-0.0968673	-0.160074
DVU0323	209257	-0.693761	-1.20411
VIMSS209258	209258	0.0165221	0.0321723
DVU0325	209259	-0.106393	-0.159072
DVU0326	209260	-0.486607	-0.934172
DVU0327	209261	-0.139295	-0.250189
DVU0328	209262	-0.258608	-0.4988
VIMSS209263	209263	-0.300533	-0.42695
DVU0330	209264	1.40211	2.42998
DVU0331	209265	0.92089	1.62519
VIMSS209266	209266	-0.499617	-0.864967
DVU0333	209267	-0.155745	-0.276701
DVU0334	209268	-0.363524	-0.628722
DVU0335	209269	-1.19196	-2.05003
DVU0336	209270	-1.09439	-1.98748
DVU0337	209271	-1.04247	-1.66433
DVU0339	209272	-0.485554	-0.92579
DVU0338	209273	-0.744524	-1.30897
DVU0340	209274	-0.0163094	-0.0303445
DVU0341	209275	-0.120372	-0.2094
DVU0342	209276	-0.112122	-0.212085
DVU0343	209277	-0.733488	-1.14324
DVU0344	209278	-0.861249	-1.48663
VIMSS209279	209279	-0.661388	-0.910084
DVU0346	209280	-1.11709	0
DVU0347	209281	-0.544434	-0.859809
DVU0348	209282	-1.00385	-1.83907
DVU0349	209283	-0.220674	-0.336662
DVU0350	209284	-0.744888	-1.21133
DVU0351	209285	-0.0920629	-0.147313
DVU0352	209286	-0.508543	-0.896924
DVU0353	209287	-0.417183	-0.697554
DVU0354	209288	1.31304	2.48324
DVU0355	209289	0.992271	1.54366
DVU0356	209290	-0.157826	-0.293903
DVU0357	209291	-0.118297	-0.214574
DVU0358	209292	-0.379092	-0.52066
DVU0359	209293	-0.387444	-0.661979
DVU0360	209294	0.935814	1.43618
DVU0361	209295	0.263599	0.424644
DVU0362	209296	-0.277328	-0.435696
DVU0363	209297	-1.43603	-2.74353

DVU0364	209298	0.160998	0.292513
DVU0365	209299	0.149217	0
DVU0367	209301	0.420594	0.692292
DVU0368	209302	-0.263398	-0.325131
VIMSS209303	209303	0.764168	0.756437
DVU0369	209304	2.14659	2.39215
DVU0370	209305	-0.286986	-0.394699
DVU0371	209306	-0.546867	-0.922983
DVU0372	209307	-1.02556	-1.10088
DVU0373	209308	0.301806	0.425214
DVU0374	209309	-0.107976	-0.160633
DVU0375	209310	0.599743	0.906049
DVU0376	209311	0.0841317	0.123036
DVU0377	209312	0.229091	0.351202
DVU0378	209313	-0.0977572	-0.140432
DVU0379	209314	-0.255727	-0.46287
DVU0380	209315	0.450823	0.682624
VIMSS209316	209316	1.16591	1.29617
DVU0381	209317	-0.713393	-0.818586
DVU0382	209318	2.12363	1.82823
DVU0383	209319	0.585691	0.85256
DVU0384	209320	-1.35343	-2.43023
VIMSS209321	209321	-0.338501	-0.513736
DVU0386	209322	-0.862443	-1.30963
DVU0387	209323	0.0261631	0.0405963
DVU0388	209324	-1.56904	-2.9591
DVU0389	209325	-0.839717	-1.33897
DVU0390	209326	-1.53716	-2.94085
DVU0391	209327	0.467088	0.801594
DVU0392	209328	0.0252018	0.0333428
DVU0393	209329	-0.139179	-0.233583
DVU0394	209330	-0.374487	-0.679454
DVU0395	209331	-0.376377	-0.610839
DVU0396	209332	-0.373137	-0.681934
DVU0397	209333	-0.29047	-0.481019
DVU0398	209334	-0.582013	-1.0366
DVU0399	209335	-0.542529	-0.815008
DVU0400	209336	-0.350807	-0.530848
DVU0402	209338	-0.170566	0
DVU0403	209339	-0.438165	-0.65587
DVU0404	209340	-1.16937	-1.91763
DVU0405	209341	0.00156966	0.00256058
DVU0406	209342	0.00915495	0.0150262
DVU0408	209344	0.718961	1.38302
DVU0409	209345	-0.659947	-0.977177
DVU0410	209346	0.200801	0.322165
DVU0411	209347	2.71555	4.81882
DVU0412	209348	0.362588	0.665327
DVU0413	209349	0.336218	0.607809
DVU0414	209350	-1.23993	-2.1293
DVU0415	209351	-1.34284	-2.24242
DVU0416	209352	0.944875	1.562
DVU0417	209353	-0.588185	-1.05119
DVU0418	209354	0.378705	0.708573
DVU0419	209355	1.59618	0
DVU0420	209356	1.16428	1.90459
DVU0422	209358	0.681538	0
DVU0423	209359	2.13177	3.66407
DVU0424	209360	0.206835	0.348117
DVU0425	209361	-0.236807	-0.397221
VIMSS209363	209363	0.647013	0.718411
DVU0428	209364	-1.02626	-1.80952
DVU0429	209365	1.72875	2.72931
DVU0431	209367	1.53917	0
DVU0432	209368	1.48411	2.13676
DVU0434	209370	1.33527	2.23938
VIMSS209371	209371	0.789703	1.02623
DVU0436	209372	0.715507	1.12408
DVU0437	209373	0.387328	0.543562
DVU0438	209374	0.0499873	0.0902998
DVU0439	209375	-0.114871	-0.202783
DVU0440	209376	-0.198928	-0.272419

DVU0441	209377	0.396372	0.710151
DVU0442	209378	0.76289	1.15849
DVU0443	209379	2.09126	0
DVU0444	209380	0.625563	0.88388
DVU0445	209381	0.0707844	0.111833
DVU0446	209382	0.306513	0.438293
DVU0447	209383	0.414446	0.64984
DVU0448	209384	-0.142097	-0.27015
DVU0450	209386	-0.382805	-0.687312
DVU0451	209387	-1.08461	-1.82269
DVU0452	209388	0.588666	0.972578
DVU0453	209389	0.14816	0.2504
DVU0454	209390	0.0877179	0.150923
DVU0455	209391	0.580417	0.968297
DVU0456	209392	0.55889	1.00513
DVU0457	209393	0.455194	0.666017
DVU0458	209394	-1.36508	0
DVU0459	209395	-1.81644	-3.004
DVU0460	209396	-1.06158	-1.91563
DVU0461	209397	-1.50017	-2.81926
DVU0462	209398	-1.43719	-2.50784
DVU0463	209399	-1.66153	-3.21627
DVU0464	209400	-0.830087	-1.44215
DVU0465	209401	-1.76794	-2.74578
DVU0466	209402	-1.21202	-2.2643
DVU0467	209403	-0.00835127	-0.0134625
DVU0468	209404	-1.85642	-3.03167
DVU0469	209405	-0.933673	-1.28944
DVU0470	209406	-1.40487	-2.42947
DVU0471	209407	-2.44395	-4.16616
VIMSS209408	209408	-0.299429	-0.503848
VIMSS209409	209409	0.77679	1.21908
DVU0475	209411	-1.15616	-2.00924
DVU0477	209413	-0.345018	-0.571086
DVU0478	209414	0.5212	0.772478
DVU0479	209415	0.995495	1.33747
DVU0480	209416	-0.470618	-0.771646
DVU0481	209417	-0.455063	-0.878642
DVU0482	209418	1.3862	2.55807
DVU0484	209420	1.0612	1.42058
DVU0485	209421	0.622569	0.999441
DVU0487	209423	-0.725511	-1.2904
DVU0489	209425	-0.519255	-0.902359
VIMSS209427	209427	0.352913	0.681041
DVU0491	209428	0.354089	0.61439
DVU0492	209429	0.302591	0.562371
DVU0493	209430	-0.0263588	-0.0449935
DVU0494	209431	0.192648	0.280218
DVU0495	209432	-0.104879	-0.18337
DVU0496	209433	0.460052	0
DVU0497	209434	0.962722	0
DVU0498	209435	-0.510486	-0.856492
DVU0499	209436	0.332886	0.617772
DVU0500	209437	-0.848637	-1.52189
DVU0501	209438	-1.07109	-1.95311
DVU0502	209439	-1.4281	-2.4107
DVU0503	209440	-1.31211	-1.90581
DVU0504	209441	-1.36541	-2.56086
DVU0506	209443	-0.883774	-1.5514
DVU0507	209445	-0.675295	-0.981676
DVU0508	209446	-0.916087	-1.4729
VIMSS209447	209447	0.464332	0.715216
DVU0510	209448	-1.08842	0
DVU0511	209449	-1.30547	-2.40297
DVU0512	209450	0.42054	0.782439
DVU0513	209451	0.253594	0.455086
DVU0514	209452	0.184583	0.331719
DVU0515	209453	0.83182	1.54869
DVU0517	209455	0.718569	1.24266
DVU0518	209456	-0.365361	0
DVU0519	209457	0.232891	0.337942
DVU0520	209458	-0.0202005	-0.0307295

DVU0521	209459	-0.199075	-0.313363
DVU0522	209461	-0.0495265	-0.081088
DVU0524	209463	0.844468	0
DVU0526	209465	0.231647	0.364538
DVU0527	209466	0.380715	0.563375
DVU0528	209467	0.0352572	0.0537304
DVU0529	209468	-0.370404	-0.529162
DVU0530	209469	-0.793938	-1.15303
DVU0531	209470	-0.186711	-0.262863
DVU0532	209471	-0.232194	-0.249933
DVU0533	209472	-1.85823	-2.86845
DVU0534	209473	-1.26279	-1.57764
DVU0536	209475	-0.532342	0
VIMSS209476	209476	0.00326592	0.00390477
DVU0538	209477	0.803696	1.05109
DVU0539	209478	0.236767	0.387645
DVU0540	209479	0.0842344	0
VIMSS209480	209480	1.16686	1.58232
DVU0542	209481	0.745923	1.1921
DVU0543	209482	1.18898	1.87068
DVU0544	209483	0.130451	0.178702
DVU0545	209484	0.815477	1.21946
VIMSS209485	209485	-0.913718	0
DVU0547	209486	0.184905	0.325171
DVU0548	209487	-0.495444	-0.846311
DVU0549	209488	-0.558793	-0.932269
DVU0550	209489	-0.153119	-0.238644
DVU0551	209490	-0.124241	-0.226752
VIMSS209491	209491	-0.0166543	0
VIMSS209492	209492	-0.214877	-0.345012
VIMSS209493	209493	-0.289722	-0.343703
VIMSS209494	209494	-0.768749	0
DVU0555	209496	-0.98295	-1.80866
DVU0556	209497	-0.150744	-0.276205
DVU0557	209498	-0.032757	-0.0559425
VIMSS209499	209499	-1.3046	-2.21544
VIMSS209500	209500	-0.82106	-1.32166
DVU0561	209502	-0.446956	-0.685344
DVU0562	209503	-0.969718	-1.74758
DVU0563	209504	-0.28392	-0.53652
DVU0565	209506	0.963118	1.75258
DVU0566	209507	1.23833	0
DVU0567	209508	0.0855785	0.136251
DVU0568	209510	0.258519	0
DVU0569	209511	-0.302634	-0.560142
DVU0570	209512	1.38201	0
DVU0571	209513	0.903606	1.70069
DVU0572	209514	2.0481	3.5809
VIMSS209515	209515	0.161288	0.264666
DVU0573	209516	1.16158	2.07033
DVU0575	209518	-0.650938	0
DVU0576	209519	1.58255	2.93437
DVU0577	209520	1.28238	0
DVU0579	209522	1.19477	0
DVU0580	209523	1.12812	2.07686
DVU0581	209524	0.754481	1.42449
DVU0582	209525	0.556567	1.03095
DVU0584	209527	0.729064	1.33616
DVU0585	209528	-0.404993	0
DVU0586	209529	3.97089	5.73245
DVU0588	209532	3.43207	5.4136
DVU0590	209534	0.122314	0.17616
DVU0591	209535	1.02696	0
DVU0592	209536	0.591492	1.08811
DVU0593	209537	-0.782057	-1.13578
DVU0594	209538	0.314096	0.571207
DVU0595	209539	1.19405	2.26599
DVU0596	209540	0.765582	1.12263
DVU0597	209541	0.0730532	0.133726
DVU0598	209542	1.57627	2.17082
DVU0599	209543	1.18394	0
DVU0600	209544	0.299882	0.537767

DVU0601	209545	0.139132	0.23432
DVU0602	209546	1.00132	1.95767
DVU0603	209547	0.839615	1.4662
DVU0605	209549	1.03909	1.07703
DVU0606	209550	-0.705238	-1.29682
DVU0608	209552	2.01284	3.65673
DVU0611	209555	0.30738	0.480152
DVU0612	209556	1.13774	2.0216
VIMSS209559	209559	1.49918	1.32937
VIMSS209560	209560	2.73896	3.55916
DVU0616	209561	-0.104814	-0.173313
DVU0617	209562	0.108064	0
VIMSS209563	209563	0.127929	0.226933
DVU0618	209564	1.18899	1.16014
DVU0619	209565	-0.232032	-0.400026
DVU0620	209566	-0.0278948	-0.0482156
DVU0621	209567	0.114522	0.137497
DVU0622	209568	0.543943	1.02413
VIMSS209569	209569	1.76205	2.00159
DVU0624	209570	1.04923	1.931
DVU0625	209571	1.27929	0
DVU0626	209572	0.645078	1.1851
DVU0627	209573	0.226735	0.367503
DVU0628	209574	0.47784	0
DVU0629	209575	-0.082272	-0.137781
DVU0630	209576	0.0827734	0.154755
DVU0631	209577	0.746493	1.35993
DVU0632	209578	-0.28942	-0.505284
DVU0633	209579	0.220038	0.371633
DVU0634	209580	0.317176	0.554151
DVU0636	209582	0.370013	0.647953
DVU0637	209583	-0.809234	-1.24433
DVU0638	209584	0.606948	1.08137
DVU0639	209585	0.209547	0.393192
DVU0641	209587	0.0632433	0.114072
DVU0642	209588	-0.590851	-1.09149
DVU0643	209589	-0.650404	-1.16045
VIMSS209590	209590	-0.398367	-0.543791
DVUA0132	209592	0.302833	0
DVUA0131	209594	0.272918	0
DVUA0116	209607	-0.695659	0
DVUA0114	209609	0.0299206	0.037318
DVUA0111	209612	-0.173761	0
DVUA0097	209624	0.674167	0.991098
DVUA0091	209629	1.06159	1.47907
VIMSS209641	209641	-0.281882	0
DVUA0076	209643	-0.241774	0
DVUA0075	209644	-0.132409	0
VIMSS209645	209645	-0.229471	-0.415129
DVUA0074	209646	-0.877951	-1.4817
DVUA0073	209647	-0.195672	0
DVUA0070	209650	-1.15392	-2.07615
DVUA0069	209652	-0.454778	0
DVUA0036	209686	-0.62154	0
DVUA0030	209692	1.26103	1.31222
DVUA0028	209694	0.758423	0.980795
DVUA0023	209700	1.17944	0
DVUA0020	209704	-0.414745	-0.673897
DVUA0019	209705	-0.0937866	-0.117943
VIMSS209707	209707	0.700069	1.10198
DVUA0006	209717	-0.775751	-1.32884
DVUA0003	209719	0.242948	0
DVUA0147	209727	-0.603576	0
DVUA0141	209731	1.63773	0
DVUA0135	209737	-0.565544	0
DVU0030	408289	-0.40804	-0.647354
VIMSS408290	408290	-0.524913	-0.975925
DVU0251	408292	2.46154	3.69486
DVU0301	408293	-0.853227	-1.00627
DVU0483	408294	0.0799931	0.141913
DVU0583	408297	0.916446	1.65214
DVU0645	408298	-0.217857	-0.389302

DVU0673	408299	0.944001	0.796866
DVU0748	408300	0.310111	0.587268
DVU1112	408305	0.678134	1.05054
DVU1127	408306	0.615477	0.935765
DVU1156	408309	0.28252	0.460408
DVU1290	408312	0.365194	0.635504
VIMSS408313	408313	-0.876716	-1.47464
DVU1477	408317	0.805618	0.834459
DVU1478	408318	0.46983	0
DVU1481	408319	-0.553956	-1.02514
DVU1485	408321	-0.120573	-0.200838
DVU1486	408322	0.606488	0.940605
DVU1487	408323	1.26783	1.7196
VIMSS408324	408324	0.941797	1.30223
DVU1493	408325	0.87548	1.24006
DVU1495	408326	0.848195	1.08347
DVU1496	408327	1.35264	0
DVU1497	408328	0.570477	0.781656
DVU1510	408329	0.142858	0.230049
DVU1511	408330	0.777006	1.14571
DVU1519	408331	0.866408	1.28505
DVU1539	408333	-1.68244	-2.81777
VIMSS408335	408335	0.910304	0.821835
DVU1796	408339	0.400032	0.758969
DVU1918	408341	0.102033	0
DVU1964	408342	0.496435	0.897343
DVU2004	408343	0.305521	0.479479
DVU2072	408349	0.460814	0.744429
DVU2174	408352	-1.29786	-2.22184
DVU2178	408353	0.784424	1.24051
DVU2179	408354	0.761368	1.40317
VIMSS408357	408357	1.79125	2.87443
DVU2430	408358	0.146751	0.252119
DVU2499	408359	-0.306627	-0.563673
DVU2627	408360	0.553093	0.948766
DVU2840	408361	-0.116515	-0.191885
DVU3069	408362	-0.856956	-1.66591
DVU3227	408363	0.496486	0.882017
DVU3257	408365	-0.24712	-0.320699
DVUA0071	408376	-0.23538	0
