# Supplementary material for: Transcriptomic and proteomic analyses of Desulfovibrio vulgaris biofilms: Carbon and energy flow contribute to the distinct biofilm growth state
Source: BMC Genomics. 2012 Apr 16;13:138. doi: 10.1186/1471-2164-13-138 (PMC3431258; doi:10.1186/1471-2164-13-138)
Supplement: Additional file 5 — Protein expression ratios between biofilm to batch, planktonic cells; biofilm to reactor, planktonic cells; or reactor, planktonic to batch, planktonic cells. [file 1471-2164-13-138-S5.pdf]

Additional file 5. Protein expression ratios between biofilm to batch, planktonic cells; biofilm to reactor, planktonic cells; or reactor, planktonic to batch, planktonic cells**.**

BIOFILM RESULTS

DVU	Gene Name	Description	Ave Biofilm /Batch	Ave Biofilm /Planktonic	Planktonic /Batch
DVU0002	dnaN	DNA polymerase III, beta subunit	-0.83 (-0.97)	-0.53 (-0.75)	-0.35 (-0.46)
DVU0006	-	universal stress protein family	-1.32 (-1.54)	0.12 (0.11)	-1.36 (-1.95)
DVU0019	ngr	nigerythrin	0.56 (0.63)	1.21 (1.55)	-0.51 (-0.7)
DVU0024	-	conserved hypothetical protein	1.1 (1.25)	-0.38 (-0.55)	1.34 (2.02)
DVU0034	-	DSBA-like thioredoxin domain protein	0.22 (0.24)	0.02 (-0.02)	0.17 (0.31)
DVU0046	fliN	flagellar motor switch protein FliN	1.69 (1.92)	0.99 (1.26)	0.73 (1.13)
DVU0052	era	GTP-binding protein Era	-0.29 (-0.35)	-0.14 (-0.24)	-0.17 (-0.2)
DVU0056	cheV-1	chemotaxis protein CheV	-0.64 (-0.76)	-0.38 (-0.56)	-0.29 (-0.38)
DVU0060	acrA	efflux transporter, RND family, MFP subunit	-0.47 (-0.56)	-0.7 (-0.98)	0.13 (0.24)
DVU0062	-	RND efflux system, outer membrane protein, NodT family	0.2 (0.21)	0.69 (0.87)	-0.42 (-0.56)
DVU0085	trpB-1	tryptophan synthase, beta subunit	0.78 (0.88)	0.01 (-0.02)	0.7 (1.08)
DVU0095	potD-1	polyamine ABC transporter, periplasmic polyamine-binding protein	0.25 (0.27)	0.13 (0.12)	0.11 (0.21)
DVU0107	glnH	glutamine ABC transporter, periplasmic glutamine-binding protein	-1.75 (-2.03)	0.18 (0.18)	-1.81 (-2.62)
DVU0114	hisG	ATP phosphoribosyltransferase	-0.56 (-0.67)	-0.08 (-0.15)	-0.47 (-0.65)
DVU0133	-	hypothetical protein	0.75 (0.84)	0.29 (0.34)	0.44 (0.69)
DVU0136	-	hypothetical protein	-0.32 (-0.39)	-0.19 (-0.3)	-0.16 (-0.19)
DVU0138	-	response regulator	1.32 (1.5)	1.53 (1.97)	-0.07 (-0.06)
DVU0142	trpS	tryptophanyl-tRNA synthetase	-0.61 (-0.71)	-0.01 (-0.07)	-0.57 (-0.79)
DVU0143	-	conserved hypothetical protein	0.91 (1.03)	0.52 (0.64)	0.39 (0.62)
DVU0169	-	oligopeptide/dipeptide ABC transporter, periplasmic	-0.26 (-0.32)	-0.08 (-0.16)	-0.18 (-0.22)
DVU0186	-	conserved hypothetical protein	-1.27 (-1.48)	-0.79 (-1.09)	-0.54 (-0.75)
DVU0227	-	conserved hypothetical protein	0.59 (0.66)	-0.14 (-0.24)	0.66 (1.02)
DVU0237	serS	seryl-tRNA synthetase	-0.7 (-0.83)	-0.55 (-0.78)	-0.2 (-0.25)
DVU0240	-	hypothetical protein	1.01 (1.14)	1 (1.27)	0.08 (0.16)
DVU0241	-	MTH1175-like domain family protein	-0.45 (-0.53)	-1.03 (-1.42)	0.44 (0.69)
DVU0247	ntrX	response regulator	-0.18 (-0.23)	-0.09 (-0.17)	-0.11 (-0.11)
DVU0251	-	membrane protein, putative	1.21 (1.37)	2.2 (2.85)	-0.74 (-1.04)
DVU0253	-	oxidoreductase, FAD/iron-sulfur cluster-binding domain protein	-1.63 (-1.9)	-0.47 (-0.67)	-1.15 (-1.65)
DVU0259	divK	DNA-binding response regulator	0.35 (0.39)	0.29 (0.33)	0.07 (0.15)
DVU0261	-	universal stress protein family	0.01 (0)	1.23 (1.58)	-1.04 (-1.49)
DVU0262	-	hypothetical protein	-0.21 (-0.26)	-0.52 (-0.74)	0.22 (0.37)
DVU0264	tmcB	Transmembrane complex, ferredoxin, 2 [4Fe-4S]	-0.36 (-0.43)	0.58 (0.71)	-0.84 (-1.19)
DVU0266	-	hypothetical protein	-0.2 (-0.25)	0.1 (0.08)	-0.29 (-0.38)
DVU0269	-	transcriptional regulator, rrf2 protein, putative	0.27 (0.29)	-0.26 (-0.4)	0.46 (0.74)
DVU0276	-	conserved hypothetical protein	-0.05 (-0.08)	0.05 (0.02)	-0.11 (-0.12)
DVU0278	-	glyoxalase family protein	0.16 (0.17)	-0.04 (-0.1)	0.17 (0.3)
DVU0283	-	AhpF family protein/thioredoxin reductase	-0.63 (-0.74)	0.2 (0.21)	-0.78 (-1.09)
DVU0284	ppiB-1	peptidyl-prolyl cis-trans isomerase B	0.89 (1.01)	1.13 (1.44)	-0.13 (-0.14)
DVU0285	hisH	imidazole glycerol phosphate synthase, glutamine amidotransferase subunit	1 (1.14)	0.56 (0.69)	0.44 (0.69)
DVU0286	hisF	imidazoleglycerol phosphate synthase, cyclase subunit	0.1 (0.1)	0.08 (0.06)	0.01 (0.07)
DVU0296	-	peptidase, M24 family	-0.57 (-0.67)	-0.34 (-0.5)	-0.25 (-0.32)
DVU0311	-	flagellar assembly protein FliH, putative	0.44 (0.49)	0.15 (0.15)	0.26 (0.44)
DVU0318	-	TPR domain protein	-1.43 (-1.66)	-2.23 (-3)	0.54 (0.85)
DVU0319	-	NAD-dependent epimerase/dehydratase family protein	-1.12 (-1.31)	0.2 (0.21)	-1.24 (-1.78)
DVU0322	eno	enolase	0.29 (0.32)	0.4 (0.47)	-0.07 (-0.05)

DVU0323	folD	methylenetetrahydrofolate dehydrogenase/methenyltetrahydrofolate cyclohydrolase	-0.51 (-0.61)	0.53 (0.65)	-0.95 (-1.35)
DVU0325	hypD	hydrogenase expression/formation protein HypD	-0.43 (-0.52)	-0.2 (-0.32)	-0.24 (-0.3)
DVU0339	serA	D-isomer specific 2-hydroxyacid dehydrogenase family protein	-0.46 (-0.55)	-0.43 (-0.62)	-0.08 (-0.06)
DVU0341	kdsB	3-deoxy-D-manno-octulosonate cytidylyltransferase	-0.87 (-1.02)	-0.83 (-1.15)	-0.13 (-0.14)
DVU0343	-	HPCH/HPAI aldolase family protein	-0.83 (-0.98)	-0.25 (-0.38)	-0.58 (-0.8)
DVU0353	-	alcohol dehydrogenase, iron-containing	-0.67 (-0.79)	0.68 (0.85)	-1.23 (-1.76)
DVU0367	-	Ser/Thr protein phosphatase family protein	0.92 (1.04)	0.97 (1.24)	0 (0.05)
DVU0386	glnH	amino acid ABC transporter, periplasmic amino acid-binding protein	1.33 (1.51)	0.35 (0.41)	0.93 (1.42)
DVU0396	hup-1	DNA-binding protein HU	1.04 (1.18)	0.9 (1.15)	0.19 (0.33)
DVU0401	-	hypothetical protein	1 (1.13)	-0.1 (-0.18)	1.01 (1.54)
DVU0402	dsrA	dissimilatory sulfite reductase alpha subunit	0.28 (0.31)	0.3 (0.35)	0 (0.03)
DVU0403	dvsB	dissimilatory sulfite reductase beta subunit	0.21 (0.22)	1.1 (1.41)	-0.75 (-1.06)
DVU0404	dsrD	dissimilatory sulfite reductase D	1.38 (1.57)	0.2 (0.21)	1.11 (1.68)
DVU0410	-	hypothetical protein	-0.04 (-0.06)	-0.72 (-1.01)	0.56 (0.88)
DVU0414	tme	NADP-dependent malic enzyme-related protein	-0.77 (-0.9)	0.22 (0.25)	-0.93 (-1.32)
DVU0415	pepA	cytosol aminopeptidase	-0.04 (-0.07)	-1.55 (-2.1)	1.25 (1.89)
DVU0423	-	universal stress protein family	0.59 (0.67)	0.89 (1.13)	-0.2 (-0.25)
DVU0429	-	Ech hydrogenase, subunit EchF, putative	3.41 (3.9)	1.75 (2.26)	1.68 (2.53)
DVU0430	-	Ech hydrogenase, subunit EchE, putative	1.42 (1.61)	1.66 (2.15)	-0.09 (-0.09)
DVU0448	gmd	GDP-mannose 4,6-dehydratase	-0.33 (-0.39)	0.01 (-0.02)	-0.34 (-0.45)
DVU0460	-	predicted phospho-2-dehydro-3-deoxyheptonate aldolase	0.45 (0.5)	-0.08 (-0.16)	0.48 (0.76)
DVU0461	-	predicted 3-dehydroquinate synthase	-0.91 (-1.07)	-1.05 (-1.44)	0.02 (0.08)
DVU0462	-	chorismate mutase/prephenate dehydratase	-1.99 (-2.31)	-0.32 (-0.48)	-1.61 (-2.32)
DVU0463	aroA	3-phosphoshikimate 1-carboxyvinyltransferase	-1.24 (-1.44)	-0.93 (-1.28)	-0.39 (-0.52)
DVU0465	trpE	anthranilate synthase, component I	-1.72 (-1.99)	-1.29 (-1.76)	-0.53 (-0.73)
DVU0467	trpD	anthranilate phosphoribosyltransferase	-1.64 (-1.9)	-1.52 (-2.06)	-0.26 (-0.34)
DVU0470	trpB-2	tryptophan synthase, beta subunit	-1.94 (-2.25)	-1.02 (-1.4)	-0.96 (-1.37)
DVU0471	trpA	tryptophan synthase, alpha subunit	-1.25 (-1.45)	-0.86 (-1.19)	-0.44 (-0.6)
DVU0477	icd	isocitrate dehydrogenase, NADP-dependent	-0.67 (-0.79)	-0.74 (-1.03)	-0.01 (0.02)
DVU0481	rfaD	ADP-L-glycero-D-mannoheptose-6-epimerase	0.24 (0.26)	0.21 (0.23)	0.02 (0.09)
DVU0487	purE	phosphoribosylaminoimidazole carboxylase, catalytic subunit	0.11 (0.11)	-0.53 (-0.75)	0.55 (0.87)
DVU0488	purD	phosphoribosylamine--glycine ligase	0.06 (0.05)	-0.19 (-0.31)	0.22 (0.38)
DVU0489	paaK-1	phenylacetate-coenzyme A ligase	-1.39 (-1.62)	-1.26 (-1.71)	-0.25 (-0.31)
DVU0492	argC	N-acetyl-gamma-glutamyl-phosphate reductase	0.76 (0.85)	0.44 (0.54)	0.28 (0.47)
DVU0494	-	aminotransferase, class V	-3.3 (-3.82)	-1.82 (-2.46)	-1.56 (-2.24)
DVU0501	-	conserved hypothetical protein	-0.41 (-0.49)	-0.3 (-0.45)	-0.14 (-0.16)
DVU0503	pnp	polyribonucleotide nucleotidyltransferase	-1.21 (-1.41)	-0.1 (-0.19)	-1.06 (-1.5)
DVU0504	rpsO	ribosomal protein S15	0.83 (0.94)	-0.61 (-0.86)	1.29 (1.95)
DVU0508	infB	translation initiation factor IF-2	-0.58 (-0.68)	-1.26 (-1.72)	0.51 (0.8)
DVU0510	nusA	N utilization substance protein A	-0.4 (-0.47)	-0.88 (-1.22)	0.36 (0.58)
DVU0522	yviF	conserved hypothetical protein	0.56 (0.63)	1.12 (1.44)	-0.44 (-0.6)
DVU0523	flgM	negative regulator of flagellin synthesis FlgM	0.52 (0.58)	1.34 (1.72)	-0.67 (-0.93)
DVU0547	-	high-affinity branched chain amino acid ABC transporter, periplasmic	1.38 (1.57)	0.95 (1.21)	0.47 (0.74)
DVU0551	livF	high-affinity branched-chain amino acid ABC transporter	0.24 (0.26)	-0.05 (-0.13)	0.26 (0.44)
DVU0565	gap-1	glyceraldehyde 3-phosphate dehydrogenase	0.78 (0.87)	0.81 (1.01)	0.02 (0.09)
DVU0566	-	GAF domain protein	0.76 (0.86)	0.7 (0.88)	0.1 (0.21)
DVU0587	fdnG-1	formate dehydrogenase, alpha subunit, selenocysteine-containing	1.5 (1.71)	2.59 (3.38)	-0.8 (-1.13)
DVU0588	hybA	formate dehydrogenase, beta subunit, putative	1.94 (2.21)	2.11 (2.74)	0 (0.06)
DVU0601	b1396	phenylacetic acid degradation protein PaaI	1.13 (1.28)	0.28 (0.33)	0.81 (1.24)
DVU0607	ahcY	adenosylhomocysteinase	-2.06 (-2.39)	0.71 (0.89)	-2.56 (-3.72)
DVU0614	-	hypothetical protein	-0.04 (-0.07)	-0.38 (-0.56)	0.27 (0.45)

DVU0625	nrfA	cytochrome c nitrite reductase, catalytic subunit NrfA, putative	0.56 (0.63)	0.91 (1.16)	-0.27 (-0.35)
DVU0627	ptB	phosphotransbutyrylase	-1.17 (-1.36)	-0.03 (-0.09)	-1.1 (-1.56)
DVU0631	-	conserved hypothetical protein	0.24 (0.26)	0.31 (0.36)	-0.05 (-0.02)
DVU0645	-	methyl-accepting chemotaxis protein	0.64 (0.72)	0.19 (0.21)	0.42 (0.67)
DVU0646	cobI	precorrin-2 C20-methyltransferase	-0.86 (-1)	0.1 (0.08)	-0.93 (-1.32)
DVU0647	-	iron compound ABC transporter, periplasmic	-0.62 (-0.73)	-0.03 (-0.09)	-0.57 (-0.78)
DVU0650	-	chelatase, putative	-0.9 (-1.05)	0.55 (0.68)	-1.33 (-1.91)
DVU0663	cysK	cysteine synthase A	0.9 (1.02)	0.65 (0.81)	0.27 (0.45)
DVU0664	-	cysteine desulfurase	-1.11 (-1.3)	-0.47 (-0.68)	-0.65 (-0.91)
DVU0665	-	nitrogen fixation protein nifU	-0.83 (-0.97)	-0.48 (-0.69)	-0.37 (-0.49)
DVU0671	-	conserved hypothetical protein	-0.74 (-0.87)	-1.05 (-1.44)	0.18 (0.32)
DVU0675	fliY	amino acid ABC transporter, periplasmic amino acid-binding protein	0.55 (0.62)	0.64 (0.8)	-0.04 (-0.01)
DVU0682	-	DNA-binding protein, putative	-0.68 (-0.8)	-0.57 (-0.8)	-0.18 (-0.21)
DVU0683	-	hflC protein, putative	-0.63 (-0.75)	-0.07 (-0.14)	-0.55 (-0.77)
DVU0684	hflK	hflK protein, putative	-0.36 (-0.44)	0.33 (0.39)	-0.64 (-0.9)
DVU0693	narH	molybdopterin oxidoreductase, cytochrome c heme-binding	0.44 (0.49)	-0.21 (-0.33)	0.57 (0.89)
DVU0694	-	molybdopterin oxidoreductase, molybdopterin-binding subunit	0.13 (0.14)	0.16 (0.16)	-0.02 (0.01)
DVU0701	glcB	malate synthase G	0 (-0.01)	0.25 (0.28)	-0.22 (-0.28)
DVU0707	dctP	TRAP dicarboxylate family transporter	0.63 (0.71)	-0.42 (-0.61)	0.93 (1.42)
DVU0712	-	amino acid ABC transporter, periplasmic-binding protein	1.45 (1.65)	1 (1.28)	0.48 (0.77)
DVU0715	livG	branched-chain amino acid ABC transporter, ATP binding protein	#VALUE!	#VALUE!	-0.19 (-0.23)
DVU0716	livF	branched-chain amino acid ABC transporter, ATP-binding protein	0.46 (0.51)	0.23 (0.26)	0.21 (0.37)
DVU0734	cysG-1	uroporphyrinogen III synthase/methyltransferase	-0.57 (-0.68)	0.06 (0.02)	-0.6 (-0.84)
DVU0752	-	amino acid ABC transporter, amino acid-binding protein	1.95 (2.22)	0.6 (0.75)	1.29 (1.95)
DVU0756	-	TPR domain protein	-0.2 (-0.25)	-0.59 (-0.84)	0.3 (0.49)
DVU0761	-	lipoprotein, putative	-0.68 (-0.8)	0.42 (0.51)	-1.01 (-1.44)
DVU0764	hup-2	DNA-binding protein HU	0.41 (0.46)	0.09 (0.07)	0.3 (0.49)
DVU0774	atpC	ATP synthase, F1 epsilon subunit	1.62 (1.84)	0.4 (0.48)	1.15 (1.75)
DVU0775	atpD	ATP synthase, F1 beta subunit	-0.09 (-0.12)	-0.92 (-1.26)	0.68 (1.05)
DVU0776	atpG	ATP synthase, F1 gamma subunit	0.15 (0.16)	-0.68 (-0.95)	0.71 (1.1)
DVU0777	atpA	ATP synthase, F1 alpha subunit	0.72 (0.81)	0.02 (-0.02)	0.64 (0.99)
DVU0778	atpH	ATP synthase, F1 delta subunit	0.92 (1.04)	-0.38 (-0.56)	1.17 (1.78)
DVU0779	atpF2	ATP synthase F0, B subunit, putative	0.71 (0.8)	0.35 (0.41)	0.35 (0.57)
DVU0780	atpF1	ATP synthase F0, B subunit, putative	0 (-0.01)	-0.36 (-0.53)	0.3 (0.49)
DVU0789	mreB-1	rod shape-determining protein MreB	-0.63 (-0.74)	-0.25 (-0.38)	-0.38 (-0.52)
DVU0794	fabI	enoyl-acyl-carrier-protein reductase	-0.69 (-0.82)	-0.65 (-0.91)	-0.11 (-0.11)
DVU0795	purC	phosphoribosylaminoimidazole-succinocarboxamide synthase	-0.33 (-0.4)	-0.42 (-0.6)	0.02 (0.09)
DVU0797	-	conserved hypothetical protein	2.15 (2.45)	3.08 (4.02)	-0.61 (-0.85)
DVU0799	-	conserved hypothetical protein	3.02 (3.45)	3.65 (4.78)	-0.29 (-0.38)
DVU0808	gatA	glutamyl-tRNA Gln amidotransferase, A subunit	-0.76 (-0.89)	0.86 (1.09)	-1.46 (-2.1)
DVU0811	dnaK	dnaK protein	-1.04 (-1.22)	-0.52 (-0.74)	-0.55 (-0.76)
DVU0812	grpE	heat shock protein GrpE	-0.07 (-0.1)	-0.63 (-0.89)	0.45 (0.72)
DVU0819	isf-1	FMN reductase, NADPH-dependent	0.46 (0.52)	0.69 (0.87)	-0.16 (-0.19)
DVU0823	argJ	arginine biosynthesis bifunctional protein ArgJ	-0.6 (-0.71)	-0.97 (-1.34)	0.25 (0.42)
DVU0835	rplS	ribosomal protein L19	0.06 (0.05)	-0.06 (-0.14)	0.1 (0.2)
DVU0838	-	conserved hypothetical protein	-0.45 (-0.54)	-0.23 (-0.36)	-0.23 (-0.3)
DVU0839	rpsP	ribosomal protein S16	0.35 (0.38)	-0.01 (-0.07)	0.33 (0.54)
DVU0841	-	aspartate aminotransferase, putative	-0.55 (-0.65)	0 (-0.06)	-0.52 (-0.72)
DVU0846	ApsB	adenylylsulphate reductase, beta subunit	0.29 (0.31)	-0.76 (-1.05)	0.9 (1.38)
DVU0847	ApsA	adenylyl-sulphate reductase, alpha subunit	0.1 (0.09)	0.71 (0.89)	-0.52 (-0.72)
DVU0848	QmoA	Quinone-interacting membrane-bound oxidoreductase	0.64 (0.71)	0.24 (0.27)	0.37 (0.59)
DVU0849	QmoB	Quinone-interacting membrane-bound oxidoreductase	-0.15 (-0.19)	0.22 (0.24)	-0.34 (-0.45)

DVU0851	-	hypothetical protein	1.42 (1.61)	0.02 (-0.02)	1.29 (1.96)
DVU0856	hemB	porphobilinogen synthase	0 (-0.01)	0.18 (0.19)	-0.17 (-0.2)
DVU0857	nirJ-1	radical SAM domain protein	0.15 (0.16)	0.76 (0.95)	-0.5 (-0.69)
DVU0869	uppS	undecaprenyl diphosphate synthase	-0.45 (-0.53)	-0.91 (-1.26)	0.33 (0.53)
DVU0870	frr	ribosome recycling factor	-0.64 (-0.75)	0.16 (0.16)	-0.75 (-1.06)
DVU0873	tsf	translation elongation factor Ts	-0.57 (-0.67)	-1.4 (-1.91)	0.64 (1)
DVU0874	rpsB	ribosomal protein S2	-0.04 (-0.07)	-0.57 (-0.81)	0.43 (0.68)
DVU0890	hom	homoserine dehydrogenase	-1.36 (-1.58)	-0.11 (-0.2)	-1.18 (-1.69)
DVU0891	-	aminotransferase, classes I and II	-1.06 (-1.24)	0.23 (0.26)	-1.21 (-1.74)
DVU0893	-	universal stress protein family	0.01 (0)	0.06 (0.04)	-0.06 (-0.03)
DVU0894	aroC	chorismate synthase	-0.28 (-0.35)	-0.23 (-0.36)	-0.08 (-0.08)
DVU0900	gmk	guanylate kinase	-0.28 (-0.34)	-0.47 (-0.67)	0.1 (0.2)
DVU0902	-	TPR domain protein	0.09 (0.09)	-0.27 (-0.42)	0.31 (0.51)
DVU0927	rplU	ribosomal protein L21	0.75 (0.84)	-0.05 (-0.12)	0.73 (1.13)
DVU0928	rpmA	ribosomal protein L27	1.14 (1.29)	0.18 (0.19)	0.89 (1.36)
DVU0929	obg	GTP-binding protein, GTP1/OBG family	0.24 (0.26)	0.07 (0.05)	0.14 (0.26)
DVU0951	moeA-2	molybdopterin biosynthesis MoeA protein, putative	-0.62 (-0.73)	-1.03 (-1.42)	0.28 (0.46)
DVU0953	tyrS	tyrosyl-tRNA synthetase	-0.72 (-0.85)	-0.63 (-0.89)	-0.15 (-0.17)
DVU0956	rpsF	ribosomal protein S6	0.64 (0.72)	0.19 (0.2)	0.42 (0.67)
DVU0957	rpsR	ribosomal protein S18	0.11 (0.11)	-0.57 (-0.81)	0.58 (0.9)
DVU0958	rplI	ribosomal protein L9	0.69 (0.77)	0.15 (0.15)	0.5 (0.79)
DVU0961	-	conserved hypothetical protein	0.18 (0.19)	0.39 (0.47)	-0.18 (-0.22)
DVU0964	fragment	Glu/Leu/Phe/Val dehydrogenase family protein	-0.56 (-0.66)	0.47 (0.57)	-0.94 (-1.33)
DVU0966	-	amino acid ABC transporter, periplasmic amino acid-binding protein	-0.68 (-0.8)	-1.2 (-1.64)	0.36 (0.59)
DVU0979	b1200	DAK1 domain protein	0.88 (1)	1.07 (1.36)	-0.09 (-0.08)
DVU0987	-	heavy metal-binding domain protein	0.7 (0.79)	0.32 (0.37)	0.36 (0.58)
DVU0988	cbhK	carbohydrate kinase, PfkB family	-0.88 (-1.03)	-0.69 (-0.96)	-0.25 (-0.31)
DVU0992	cheV-3	chemotaxis protein CheV	0.29 (0.32)	-0.14 (-0.24)	0.37 (0.6)
DVU0995	-	ThiJ/PfpI family protein	-0.83 (-0.97)	-0.38 (-0.55)	-0.47 (-0.64)
DVU0997	metF	5,10-methylenetetrahydrofolate reductase	-1.79 (-2.08)	-0.15 (-0.25)	-1.57 (-2.26)
DVU1012	-	hemolysin-type calcium-binding repeat protein	1.33 (1.51)	0.73 (0.92)	0.61 (0.94)
DVU1013	-	type I secretion outer membrane protein, TolC family	0.12 (0.13)	0.2 (0.21)	-0.06 (-0.04)
DVU1021	-	conserved hypothetical protein	-0.45 (-0.53)	-0.6 (-0.85)	0.07 (0.16)
DVU1025	upp	uracil phosphoribosyltransferase	0.3 (0.33)	-0.24 (-0.37)	0.47 (0.75)
DVU1030	-	universal stress protein family	0.18 (0.19)	0.3 (0.34)	-0.1 (-0.09)
DVU1038	hisA	phosphoribosylformimino-5-aminoimidazole carboxamide ribotide isomerase	-1.56 (-1.81)	-0.95 (-1.31)	-0.66 (-0.93)
DVU1044	guaB	inosine-5`-monophosphate dehydrogenase	0.17 (0.18)	-0.06 (-0.13)	0.2 (0.34)
DVU1064	aco	aconitate hydratase, putative	-0.93 (-1.09)	0.5 (0.62)	-1.33 (-1.91)
DVU1065	-	peptidyl-prolyl cis-trans isomerse domain protein	-1.42 (-1.65)	-0.79 (-1.1)	-0.67 (-0.94)
DVU1066	gpt	xanthine-guanine phosphoribosyltransferase	0.39 (0.43)	-0.14 (-0.23)	0.47 (0.74)
DVU1067	-	membrane protein, Bmp family	0.84 (0.95)	1.6 (2.06)	-0.58 (-0.81)
DVU1074	rpmH	ribosomal protein L34	1.15 (1.31)	-0.4 (-0.58)	1.4 (2.11)
DVU1078	-	R3H domain protein	0.45 (0.5)	-0.22 (-0.35)	0.6 (0.93)
DVU1090	recA	recA protein	-0.01 (-0.03)	0.09 (0.07)	-0.1 (-0.11)
DVU1094	argH	argininosuccinate lyase	-0.98 (-1.14)	0.1 (0.08)	-1.01 (-1.44)
DVU1095	argG	argininosuccinate synthase	-1.96 (-2.27)	-0.36 (-0.53)	-1.54 (-2.22)
DVU1096	argF	ornithine carbamoyltransferase	-0.55 (-0.65)	-0.15 (-0.25)	-0.41 (-0.55)
DVU1174	-	hypothetical protein	0.65 (0.73)	0.62 (0.77)	0.07 (0.15)
DVU1176	-	hypothetical protein	-0.37 (-0.45)	-1.22 (-1.66)	0.66 (1.02)
DVU1177	-	hypothetical protein	-0.34 (-0.4)	-0.44 (-0.64)	0.05 (0.12)
DVU1179	aor	aldehyde:ferredoxin oxidoreductase, tungsten-containing	0.47 (0.53)	0.77 (0.96)	-0.22 (-0.27)
DVU1197	nusB	N utilization substance protein B	0.67 (0.76)	0.69 (0.86)	0.03 (0.09)
DVU1198	ribH	riboflavin synthase, beta subunit	0.3 (0.33)	-0.33 (-0.49)	0.56 (0.87)
DVU1199	ribAB	3,4-dihydroxy-2-butanone 4-phosphate synthase/GTP cyclohydrolase II	-0.38 (-0.45)	-0.8 (-1.1)	0.3 (0.5)
DVU1203	glyA	serine hydroxymethyltransferase	-0.81 (-0.94)	-0.57 (-0.81)	-0.28 (-0.36)
DVU1204	fabF	3-oxoacyl-acyl-carrier-protein synthase II	1.02 (1.16)	0.24 (0.26)	0.74 (1.15)
DVU1206	fabG	3-oxoacyl-acyl-carrier-protein reductase	-0.69 (-0.81)	-0.46 (-0.67)	-0.26 (-0.33)

DVU1207	fabH	3-oxoacyl-acyl-carrier-protein synthase III	-1.72 (-2)	-1.04 (-1.42)	-0.74 (-1.05)
DVU1211	rpmB	ribosomal protein L28	0.16 (0.16)	-0.46 (-0.65)	0.52 (0.82)
DVU1222	-	hypothetical protein	-0.19 (-0.23)	-0.95 (-1.31)	0.62 (0.96)
DVU1228	tpX	thiol peroxidase	0.12 (0.12)	-0.39 (-0.57)	0.43 (0.68)
DVU1232	glnB-1	nitrogen regulatory protein P-II	0.61 (0.69)	1.38 (1.77)	-0.6 (-0.84)
DVU1236	-	amino acid ABC transporter, ATP-binding protein	-0.87 (-1.02)	-1.24 (-1.69)	0.21 (0.37)
DVU1238	-	amino acid ABC transporter, periplasmic amino acid-binding protein	0.86 (0.98)	-0.06 (-0.13)	0.85 (1.3)
DVU1241	-	conserved hypothetical protein	-2.55 (-2.95)	0.96 (1.22)	-3.21 (-4.67)
DVU1248	argS	arginyl-tRNA synthetase	-0.1 (-0.13)	0 (-0.03)	-0.11 (-0.12)
DVU1249	fabD	malonyl CoA-acyl carrier protein transacylase	-0.5 (-0.59)	-0.74 (-1.04)	0.15 (0.27)
DVU1251	-	hypothetical protein	0.97 (1.1)	0.58 (0.71)	0.39 (0.63)
DVU1257	-	RNA-binding protein	-0.15 (-0.19)	-2 (-2.69)	1.53 (2.3)
DVU1265	-	hypothetical protein	0.72 (0.81)	0.6 (0.75)	0.15 (0.27)
DVU1279	folP	dihydropteroate synthase	0.37 (0.41)	-0.36 (-0.53)	0.64 (1)
DVU1283	galU	UTP-glucose-1-phosphate uridylyltransferase	-1.19 (-1.38)	-0.88 (-1.21)	-0.38 (-0.51)
DVU1287	DsrO	Periplasmic Tat, binds 2[4Fe-4S]	0.53 (0.59)	0.44 (0.53)	0.11 (0.22)
DVU1289	DsrK	Cytoplasmic, binds 2 [4Fe-4S]	0.26 (0.28)	0.22 (0.24)	0.04 (0.11)
DVU1290	DsrM	Inner membrane protein binds 2 heme b	1.49 (1.69)	0.61 (0.76)	0.85 (1.3)
DVU1295	sat	sulfate adenylyltransferase	0.91 (1.03)	1.17 (1.5)	-0.15 (-0.17)
DVU1298	rpsL	ribosomal protein S12	-0.59 (-0.7)	-0.76 (-1.06)	0.08 (0.17)
DVU1299	rpsG	ribosomal protein S7	0.95 (1.07)	0.3 (0.35)	0.61 (0.95)
DVU1300	fusA-1	translation elongation factor G	-0.64 (-0.75)	0.99 (1.26)	-1.46 (-2.1)
DVU1302	rpsJ	ribosomal protein S10	0.82 (0.92)	0.13 (0.12)	0.64 (0.99)
DVU1303	rplC	ribosomal protein L3	0.3 (0.32)	-0.41 (-0.6)	0.62 (0.96)
DVU1304	rplD	ribosomal protein L4	0.1 (0.09)	-0.06 (-0.13)	0.13 (0.24)
DVU1305	rplW	ribosomal protein L23	0.61 (0.68)	-0.15 (-0.25)	0.68 (1.06)
DVU1306	rplB	ribosomal protein L2	-0.72 (-0.85)	-1.17 (-1.6)	0.3 (0.5)
DVU1307	rpsS	ribosomal protein S19	0.46 (0.51)	-0.14 (-0.23)	0.54 (0.84)
DVU1308	rplV	ribosomal protein L22	-0.37 (-0.44)	-0.58 (-0.81)	0.12 (0.23)
DVU1309	rpsC	ribosomal protein S3	0.15 (0.16)	-0.41 (-0.6)	0.48 (0.76)
DVU1310	rplP	ribosomal protein L16	0.03 (0.02)	-0.65 (-0.92)	0.58 (0.9)
DVU1311	rpmC	ribosomal protein L29	0.36 (0.4)	-0.26 (-0.39)	0.55 (0.86)
DVU1312	rpsQ	ribosomal protein S17	0.26 (0.29)	0.25 (0.28)	0.02 (0.08)
DVU1313	rplN	ribosomal protein L14	-0.27 (-0.33)	-0.8 (-1.1)	0.4 (0.65)
DVU1315	rplE	ribosomal protein L5	0.37 (0.41)	-0.42 (-0.61)	0.7 (1.08)
DVU1316	rpsN	ribosomal protein S14	0.55 (0.62)	-0.29 (-0.44)	0.75 (1.16)
DVU1317	rpsH	ribosomal protein S8	-0.29 (-0.35)	-0.36 (-0.53)	0.03 (0.09)
DVU1318	rplF	ribosomal protein L6	0.25 (0.27)	-0.25 (-0.38)	0.43 (0.69)
DVU1319	rplR	ribosomal protein L18	0.67 (0.75)	0.2 (0.22)	0.43 (0.69)
DVU1320	rpsE	ribosomal protein S5	0.08 (0.07)	0 (-0.06)	0.07 (0.15)
DVU1321	rpmD	ribosomal protein L30	0.3 (0.33)	-0.06 (-0.13)	0.32 (0.52)
DVU1322	rplO	ribosomal protein L15	-0.1 (-0.14)	-0.73 (-1.02)	0.5 (0.8)
DVU1326	rpsM	ribosomal protein S13	0.34 (0.37)	-0.44 (-0.64)	0.68 (1.06)
DVU1327	rpsK	ribosomal protein S11	-0.6 (-0.71)	-0.23 (-0.35)	-0.39 (-0.53)
DVU1328	rpsD	ribosomal protein S4	-0.49 (-0.58)	-0.94 (-1.3)	0.32 (0.52)
DVU1329	rpoA	DNA-directed RNA polymerase, alpha subunit	-0.49 (-0.58)	-0.99 (-1.36)	0.36 (0.58)
DVU1330	rplQ	ribosomal protein L17	0.2 (0.21)	-0.19 (-0.31)	0.32 (0.53)
DVU1334	tig	trigger factor	0.49 (0.55)	0.11 (0.09)	0.35 (0.57)
DVU1336	clpX	ATP-dependent Clp protease, ATP-binding subunit ClpX	-1.02 (-1.19)	-1.12 (-1.53)	0 (0.03)
DVU1337	lon	ATP-dependent protease La	-1.09 (-1.27)	-0.75 (-1.04)	-0.4 (-0.54)
DVU1343	znuA	periplasmic component of zinc ABC transporter	-1.48 (-1.72)	-0.54 (-0.76)	-0.95 (-1.35)
DVU1344	ispG	1-hydroxy-2-methyl-2-E-butenyl 4-diphosphate synthase	-0.09 (-0.12)	-1.05 (-1.44)	0.79 (1.22)
DVU1360	galE	UDP-glucose 4-epimerase	0.19 (0.2)	0.33 (0.38)	-0.1 (-0.1)
DVU1368	-	rhodanese-like domain protein	-0.79 (-0.92)	0.06 (0.03)	-0.81 (-1.14)
DVU1375	-	hypothetical protein	1.25 (1.42)	1.52 (1.96)	-0.13 (-0.15)
DVU1378	ilvC	ketol-acid reductoisomerase	-0.88 (-1.03)	-0.62 (-0.87)	-0.31 (-0.41)
DVU1397	bfr	bacterioferritin	0.56 (0.63)	0.89 (1.12)	-0.24 (-0.3)
DVU1406	purM	phosphoribosylformylglycinamidine cyclo-ligase	-0.03 (-0.05)	0.27 (0.31)	-0.28 (-0.36)

DVU1411	thiC	thiamine biosynthesis protein ThiC	-0.96 (-1.12)	-0.27 (-0.41)	-0.67 (-0.94)
DVU1420	-	Hpt domain protein	0.47 (0.53)	0.88 (1.11)	-0.31 (-0.41)
DVU1422	-	OmpA family protein	-0.86 (-1.01)	1.06 (1.35)	-1.73 (-2.5)
DVU1424	gcvPB	glycine cleavage system P protein, subunit 2	-0.58 (-0.68)	-0.18 (-0.29)	-0.37 (-0.5)
DVU1427	-	response regulator	0.45 (0.5)	0.14 (0.14)	0.28 (0.46)
DVU1430	-	peptidase, M16 family	0.28 (0.31)	0.25 (0.28)	0.02 (0.09)
DVU1431	-	hpt domain protein/STAS domain protein	1.31 (1.49)	0.53 (0.65)	0.76 (1.17)
DVU1434	-	hypothetical protein	0.2 (0.21)	-0.36 (-0.52)	0.48 (0.76)
DVU1441	flaB1	flagellin	0.44 (0.49)	0.18 (0.19)	0.24 (0.4)
DVU1443	flgE	flagellar hook protein FlgE	1.09 (1.24)	1.29 (1.66)	-0.08 (-0.07)
DVU1455	-	conserved hypothetical protein	0.04 (0.03)	-0.1 (-0.18)	0.11 (0.21)
DVU1457	trxB	thioredoxin reductase, putative	0.04 (0.02)	0.37 (0.44)	-0.29 (-0.38)
DVU1458	-	chemotaxis protein CheZ, putative	0.48 (0.54)	0.11 (0.1)	0.34 (0.55)
DVU1466	argB	acetylglutamate kinase	0.08 (0.07)	-0.47 (-0.67)	0.46 (0.73)
DVU1467	hslU	heat shock protein HslVU, ATPase subunit HslU	-0.54 (-0.63)	-0.6 (-0.84)	0 (0.03)
DVU1468	htrA	peptidase/PDZ domain protein	0.35 (0.39)	0.73 (0.92)	-0.3 (-0.4)
DVU1469	rpsA	ribosomal protein S1, putative	0.12 (0.13)	-0.58 (-0.81)	0.59 (0.92)
DVU1470	ppiC	peptidyl-prolyl cis-trans isomerase C	0.65 (0.73)	0 (-0.06)	0.6 (0.94)
DVU1537	-	lipoprotein, putative	-0.31 (-0.37)	-0.05 (-0.12)	-0.26 (-0.33)
DVU1539	glpX	fructose-1,6-bisphosphatase, class II	0.02 (0.01)	-0.29 (-0.43)	0.25 (0.43)
DVU1545	-	hemolysin-type calcium-binding repeat/calx-beta domain protein	0.92 (1.04)	-0.04 (-0.1)	0.89 (1.36)
DVU1568	ftn	ferritin	0.53 (0.59)	0.77 (0.97)	-0.18 (-0.21)
DVU1569	porA	pyruvate ferredoxin oxidoreductase, alpha subunit	-0.39 (-0.46)	1.06 (1.35)	-1.28 (-1.84)
DVU1570	porB	pyruvate ferredoxin oxidoreductase, beta subunit	-0.1 (-0.14)	1.25 (1.6)	-1.16 (-1.66)
DVU1574	rplY	ribosomal protein L25	0.79 (0.89)	0.12 (0.1)	0.62 (0.97)
DVU1575	prsA	ribose-phosphate pyrophosphokinase	-0.36 (-0.43)	0.27 (0.31)	-0.58 (-0.81)
DVU1580	rpiB	ribose 5-phosphate isomerase, putative	0.36 (0.4)	-0.45 (-0.65)	0.72 (1.11)
DVU1588	hpt	hypoxanthine phosphoribosyltransferase	-0.31 (-0.37)	-0.04 (-0.11)	-0.27 (-0.35)
DVU1593	cheY-1	chemotaxis protein CheY	-0.23 (-0.29)	0.38 (0.46)	-0.57 (-0.78)
DVU1597	sir	sulfite reductase, assimilatory-type	0.27 (0.29)	0.47 (0.57)	-0.16 (-0.18)
DVU1602	clpA	ATP-dependent Clp protease, ATP-binding subunit ClpA	-0.09 (-0.12)	0.1 (0.08)	-0.19 (-0.23)
DVU1604	-	hypothetical protein	-0.58 (-0.68)	-0.72 (-1)	0.04 (0.12)
DVU1612	-	ACT domain protein	0.38 (0.42)	0.64 (0.8)	-0.2 (-0.25)
DVU1618	-	iojap-related protein	-0.5 (-0.59)	-0.37 (-0.54)	-0.16 (-0.19)
DVU1621	-	hypothetical protein	0.28 (0.3)	0.23 (0.26)	0.05 (0.12)
DVU1622	purQ	phosphoribosylformylglycinamidine synthase I	-0.09 (-0.12)	-0.59 (-0.83)	0.4 (0.64)
DVU1627	-	ABC transporter, ATP-binding protein	-0.72 (-0.85)	-0.13 (-0.23)	-0.58 (-0.81)
DVU1629	yfiA	ribosomal subunit interface protein	0.52 (0.58)	0.55 (0.67)	0 (0.05)
DVU1636	ppaC	inorganic pyrophosphatase, manganese-dependent	1.03 (1.16)	-0.28 (-0.42)	1.19 (1.81)
DVU1655	aspC4	aminotransferase, classes I and II	0.56 (0.63)	1.35 (1.74)	-0.64 (-0.89)
DVU1664	-	GTP-binding protein	0.03 (0.01)	-0.06 (-0.13)	0.06 (0.14)
DVU1666	efp	translation elongation factor P	0.82 (0.92)	0.27 (0.31)	0.51 (0.8)
DVU1677	tpiA	triosephosphate isomerase	0.34 (0.37)	1.44 (1.85)	-0.92 (-1.31)
DVU1681	mreB-2	rod shape-determining protein MreB	-0.17 (-0.21)	-0.41 (-0.59)	0.17 (0.31)
DVU1684	gcvT	glycine cleavage system T protein	-0.68 (-0.8)	-0.35 (-0.52)	-0.35 (-0.46)
DVU1685	-	conserved hypothetical protein TIGR00046	-0.12 (-0.15)	0.13 (0.12)	-0.26 (-0.33)
DVU1690	-	transcriptional regulator, TetR family	0.43 (0.47)	-0.12 (-0.21)	0.49 (0.77)
DVU1703	-	type I restriction-modification enzyme, R subunit	0.82 (0.92)	0.35 (0.41)	0.46 (0.72)
DVU1784	-	oxidoreductase, short-chain dehydrogenase/reductase family	-0.43 (-0.51)	0.01 (-0.02)	-0.43 (-0.58)
DVU1791	-	GatB/Yqey family protein	1.63 (1.85)	0.83 (1.05)	0.8 (1.22)
DVU1792	rpsU	ribosomal protein S21	0.22 (0.23)	-1.03 (-1.42)	1.07 (1.63)
DVU1795	hup-3	DNA-binding protein HU	1.34 (1.53)	0.35 (0.41)	0.95 (1.45)
DVU1817	cyf	cytochrome c-553	2.31 (2.64)	2.44 (3.18)	0.07 (0.16)
DVU1819	secD	protein-export membrane protein SecD	-0.73 (-0.85)	-0.39 (-0.56)	-0.37 (-0.5)
DVU1820	yajC	preprotein translocase, YajC subunit	0.44 (0.49)	0.9 (1.14)	-0.37 (-0.49)
DVU1826	-	hypothetical protein	0.77 (0.87)	-0.46 (-0.65)	1.1 (1.67)

DVU1827	-	acetylornithine deacetylase/succinyl-diaminopimelate desuccinylase	-1.39 (-1.62)	-1.36 (-1.85)	-0.15 (-0.17)
DVU1833	ppsA	phosphoenolpyruvate synthase, putative	-0.4 (-0.48)	0.1 (0.08)	-0.48 (-0.66)
DVU1834	pyc	pyruvate carboxylase, putative	-0.71 (-0.83)	-0.07 (-0.15)	-0.61 (-0.85)
DVU1838	trxB-2	thioredoxin reductase	0.38 (0.42)	0.14 (0.13)	0.22 (0.38)
DVU1839	trx	thioredoxin	-0.57 (-0.68)	-0.62 (-0.87)	-0.02 (0.01)
DVU1850	-	CBS domain protein	1.29 (1.46)	0.63 (0.78)	0.65 (1.01)
DVU1857	-	methyl-accepting chemotaxis protein	0.4 (0.44)	0.75 (0.95)	-0.28 (-0.36)
DVU1864	ihfB	DNA-binding protein HU, beta subunit, putative	0.63 (0.71)	0.35 (0.42)	0.27 (0.44)
DVU1868	dapA	dihydrodipicolinate synthase	-1.02 (-1.19)	-0.5 (-0.71)	-0.53 (-0.73)
DVU1873	ppiB-2	peptidyl-prolyl cis-trans isomerase B	-1.78 (-2.07)	-1.35 (-1.84)	-0.53 (-0.73)
DVU1874	clpB	ATP-dependent Clp protease, ATP-binding subunit ClpB	-1.14 (-1.33)	-0.16 (-0.27)	-0.93 (-1.32)
DVU1876	dnaJ	dnaJ protein, putative	0.99 (1.13)	1.56 (2.02)	-0.41 (-0.55)
DVU1878	ltaE	threonine aldolase, low-specificity	-1.54 (-1.79)	-0.08 (-0.16)	-1.39 (-1.99)
DVU1885	gatB	glutamyl-tRNA Gln amidotransferase, B subunit	-0.84 (-0.98)	-0.67 (-0.94)	-0.23 (-0.29)
DVU1886	-	hypothetical protein	-0.33 (-0.39)	-0.76 (-1.06)	0.31 (0.51)
DVU1890	hemC	porphobilinogen deaminase	0.03 (0.01)	-0.12 (-0.21)	0.11 (0.21)
DVU1891	-	conserved hypothetical protein	-0.24 (-0.3)	-0.71 (-0.99)	0.35 (0.56)
DVU1901	-	peptidyl-prolyl cis-trans isomerase domain protein	0.06 (0.05)	-0.15 (-0.25)	0.17 (0.31)
DVU1908	pdxJ	pyridoxal phosphate biosynthetic protein PdxJ	0.87 (0.98)	1.06 (1.35)	-0.09 (-0.08)
DVU1911	-	CBS domain protein	-0.42 (-0.5)	-0.66 (-0.92)	0.14 (0.26)
DVU1913	-	aspartate kinase, monofunctional class	-0.44 (-0.53)	0.05 (0.01)	-0.47 (-0.65)
DVU1917	hysB	periplasmic [NiFeSe] hydrogenase, small subunit	1.69 (1.92)	0.65 (0.81)	1 (1.53)
DVU1918	hysA	periplasmic [NiFeSe] hydrogenase, large subunit	1.62 (1.85)	0.78 (0.99)	0.83 (1.28)
DVU1919	-	hydrogenase expression/formation protein, putative	0.25 (0.27)	-0.61 (-0.86)	0.75 (1.15)
DVU1922	hynA-1	periplasmic [NiFe] hydrogenase, large subunit, isozyme 1	0.03 (0.02)	0.47 (0.57)	-0.37 (-0.49)
DVU1932	adk	adenylate kinase	0.77 (0.87)	-0.26 (-0.4)	0.93 (1.42)
DVU1936	phnC	phosphonate ABC transporter, ATP-binding protein	-0.59 (-0.7)	-0.8 (-1.1)	0.1 (0.2)
DVU1937	-	phosphonate ABC transporter, periplasmic phosphonate-binding protein	1.63 (1.86)	0.88 (1.11)	0.76 (1.18)
DVU1950	-	indolepyruvate ferredoxin oxidoreductase, beta subunit, putative	-0.94 (-1.1)	-1.46 (-1.98)	0.33 (0.55)
DVU1952	-	hypothetical protein	-0.8 (-0.93)	-0.91 (-1.25)	0.01 (0.07)
DVU1953	proA	gamma-glutamyl phosphate reductase	-0.3 (-0.36)	0.7 (0.87)	-0.91 (-1.29)
DVU1964	-	transcriptional regulator, rrf2 protein, putative	-0.34 (-0.41)	-0.15 (-0.26)	-0.19 (-0.23)
DVU1973	-	rhodanese-like domain protein	-0.56 (-0.66)	0.01 (-0.03)	-0.55 (-0.77)
DVU1976	groEL	chaperonin, 60 kDa	-0.35 (-0.42)	-0.51 (-0.73)	0.09 (0.18)
DVU1977	groES	chaperonin, 10 kDa	0.98 (1.11)	0.55 (0.67)	0.43 (0.69)
DVU1981	-	conserved hypothetical protein	0.68 (0.76)	-0.32 (-0.48)	0.9 (1.38)
DVU1986	-	conserved hypothetical protein	1.37 (1.56)	0.8 (1.01)	0.58 (0.91)
DVU2013	-	hybrid cluster protein	1.31 (1.49)	0.77 (0.97)	0.56 (0.87)
DVU2014	fprA-1	metallo-beta-lactamase family protein	0.99 (1.12)	0.36 (0.42)	0.61 (0.95)
DVU2072	cheA-3	chemotaxis protein CheA	-0.89 (-1.04)	-0.84 (-1.17)	-0.13 (-0.14)
DVU2073	cheY-2	chemotaxis protein CheY	0.71 (0.8)	0.44 (0.54)	0.26 (0.44)
DVU2078	cheB-2	protein-glutamate methylesterase CheB	-1.04 (-1.21)	-1.25 (-1.7)	0.06 (0.15)
DVU2082	flaD	flagellin, putative	0.28 (0.3)	0.79 (0.99)	-0.41 (-0.56)
DVU2093	thiH	thiH protein	-1.45 (-1.69)	-0.55 (-0.77)	-0.9 (-1.28)
DVU2094	thiG	thiG protein	-1.69 (-1.97)	-1.29 (-1.76)	-0.5 (-0.69)
DVU2100	-	universal stress protein family	1.21 (1.37)	1.85 (2.39)	-0.45 (-0.61)
DVU2103	-	iron-sulfur cluster-binding/ATPase domain protein	-1.08 (-1.26)	-0.68 (-0.95)	-0.45 (-0.61)
DVU2105	-	hypothetical protein	-0.17 (-0.21)	0.64 (0.79)	-0.72 (-1.01)
DVU2108	-	MTH1175-like domain family protein	-0.38 (-0.45)	-0.26 (-0.4)	-0.14 (-0.16)
DVU2109	mrp	MTH1175-like domain family protein	-0.39 (-0.47)	-0.07 (-0.14)	-0.32 (-0.42)
DVU2135	-	hypothetical protein	1.56 (1.77)	2.39 (3.1)	-0.58 (-0.8)
DVU2138	-	conserved hypothetical protein	0.54 (0.61)	0.21 (0.23)	0.31 (0.51)

DVU2140	tmk	thymidylate kinase	0.28 (0.31)	0.24 (0.27)	0.04 (0.11)
DVU2143	fba	fructose-1,6-bisphosphate aldolase, class II	0.15 (0.16)	0.51 (0.62)	-0.3 (-0.4)
DVU2144	gap-2	glyceraldehyde 3-phosphate dehydrogenase	0.68 (0.77)	0.25 (0.28)	0.41 (0.65)
DVU2150	dksA	dnaK suppressor protein, putative	0.57 (0.64)	0.07 (0.05)	0.46 (0.73)
DVU2201	b3011	alcohol dehydrogenase, iron-containing	0.04 (0.03)	0.15 (0.15)	-0.11 (-0.11)
DVU2203	-	endoribonuclease, L-PSP family	1.16 (1.31)	-0.91 (-1.26)	1.85 (2.77)
DVU2212	-	conserved hypothetical protein	0.03 (0.02)	-0.29 (-0.44)	0.27 (0.45)
DVU2215	-	RNA-binding protein	-0.12 (-0.16)	0.47 (0.57)	-0.53 (-0.73)
DVU2222	ssb	single-strand binding protein	-0.52 (-0.61)	-0.75 (-1.04)	0.13 (0.25)
DVU2224	-	hypothetical protein	0.72 (0.81)	0.2 (0.22)	0.48 (0.76)
DVU2226	accC	acetyl-CoA carboxylase, biotin carboxylase, putative	-0.91 (-1.06)	-0.01 (-0.06)	-0.86 (-1.21)
DVU2231	typA	GTP-binding protein TypA	-1.06 (-1.24)	-0.35 (-0.52)	-0.71 (-1)
DVU2247	ahpC	alkyl hydroperoxide reductase C	-0.42 (-0.5)	-0.16 (-0.27)	-0.27 (-0.35)
DVU2250	-	AMP-binding protein	-1.13 (-1.32)	-0.9 (-1.24)	-0.32 (-0.42)
DVU2259	-	conserved hypothetical protein TIGR01033	-0.57 (-0.68)	-0.99 (-1.36)	0.28 (0.47)
DVU2290	-	hydrogenase, CooU subunit, putative	-1.13 (-1.32)	-1.37 (-1.86)	0.08 (0.17)
DVU2291	-	carbon monoxide-induced hydrogenase CooH, putative	-0.97 (-1.14)	-1.33 (-1.81)	0.19 (0.34)
DVU2297	-	glycine/betaine/L-proline ABC transporter, periplasmic-binding protein	0.4 (0.44)	0.33 (0.39)	0.07 (0.16)
DVU2299	proV	glycine/betaine/L-proline ABC transporter, ATP binding protein	0.22 (0.24)	-0.35 (-0.51)	0.49 (0.78)
DVU2310	-	metallo-beta-lactamase family protein	0.62 (0.7)	0.16 (0.16)	0.42 (0.67)
DVU2313	pgl	6-phosphogluconolactonase	-0.66 (-0.78)	-0.04 (-0.11)	-0.6 (-0.83)
DVU2318	rbr2	rubrerythrin, putative	1.02 (1.16)	-0.22 (-0.34)	1.14 (1.72)
DVU2325	merP	mercuric transport protein periplasmic component	0.73 (0.82)	0.44 (0.54)	0.29 (0.47)
DVU2326	-	conserved domain protein	-0.36 (-0.43)	0.44 (0.53)	-0.73 (-1.03)
DVU2329	hypB	hydrogenase accessory protein HypB	-0.63 (-0.75)	-0.82 (-1.13)	0.08 (0.17)
DVU2330	E.coliMir	MRP family protein	0.75 (0.85)	0.94 (1.19)	-0.11 (-0.11)
DVU2333	ndk	nucleoside diphosphate kinase	1.77 (2.02)	0.42 (0.5)	1.28 (1.94)
DVU2336	-	carboxyl-terminal protease	0.46 (0.51)	0.2 (0.21)	0.24 (0.41)
DVU2342	-	amino acid ABC transporter, periplasmic amino acid-binding protein	2.34 (2.67)	1.5 (1.93)	0.9 (1.38)
DVU2347	argD	acetylornithine aminotransferase	-1.51 (-1.75)	0.64 (0.79)	-1.98 (-2.86)
DVU2364	-	aminotransferase, classes I and II	-1.43 (-1.66)	0.2 (0.22)	-1.55 (-2.22)
DVU2370	-	outer membrane protein OmpH, putative	0.29 (0.31)	0.13 (0.12)	0.14 (0.26)
DVU2373	-	outer membrane protein, OMP85 family	-0.26 (-0.32)	-0.02 (-0.08)	-0.24 (-0.3)
DVU2376	lysS	lysyl-tRNA synthetase	-1.67 (-1.93)	-0.93 (-1.28)	-0.81 (-1.14)
DVU2398	-	conserved hypothetical protein	-0.09 (-0.13)	-0.9 (-1.24)	0.66 (1.02)
DVU2399	-	hydrogenase, putative	0.85 (0.96)	0.8 (1.01)	0.09 (0.19)
DVU2400	-	hydrogenase, putative	0.09 (0.09)	1.41 (1.81)	-1.12 (-1.6)
DVU2401	-	hydrogenase, iron-sulfur cluster-binding subunit, putative	-0.55 (-0.65)	0.64 (0.8)	-1.08 (-1.54)
DVU2402	hdrA	heterodisulfide reductase, A subunit	-1.35 (-1.57)	0.06 (0.03)	-1.34 (-1.92)
DVU2403	hdrB	heterodisulfide reductase, B subunit	-0.07 (-0.1)	0.13 (0.12)	-0.2 (-0.24)
DVU2405	-	alcohol dehydrogenase, iron-containing	0.03 (0.01)	1.5 (1.94)	-1.26 (-1.81)
DVU2410	sodB	superoxide dismutase, Fe	0.94 (1.06)	2.02 (2.62)	-0.85 (-1.2)
DVU2420	-	conserved hypothetical protein	0.44 (0.49)	-0.44 (-0.64)	0.78 (1.2)
DVU2427	-	hypothetical protein	0.91 (1.03)	0.99 (1.26)	0 (0.04)
DVU2434	-	hypothetical protein	1.39 (1.58)	-0.29 (-0.43)	1.54 (2.31)
DVU2441	hspC	heat shock protein, Hsp20 family	0.38 (0.42)	1.63 (2.1)	-1.05 (-1.49)
DVU2442	-	heat shock protein, Hsp20 family	0.5 (0.56)	2.3 (2.99)	-1.5 (-2.15)
DVU2444	flaB3	flagellin	0.44 (0.49)	0.75 (0.94)	-0.24 (-0.3)
DVU2448	panC	pantoate--beta-alanine ligase	-1.11 (-1.3)	-0.48 (-0.69)	-0.64 (-0.9)
DVU2449	metK	S-adenosylmethionine synthetase	-1.88 (-2.18)	0.05 (0.01)	-1.82 (-2.63)
DVU2481	fdoH	formate dehydrogenase, beta subunit, putative	1.52 (1.73)	1.36 (1.74)	0.25 (0.42)
DVU2482	fdnG-2	formate dehydrogenase, alpha subunit, selenocysteine-containing	1.5 (1.71)	1.17 (1.5)	0.39 (0.62)
DVU2483	-	cytochrome c family protein	0.29 (0.32)	0.52 (0.63)	-0.18 (-0.22)
DVU2499	ftsZ	cell division protein FtsZ	-0.98 (-1.14)	-1.36 (-1.85)	0.23 (0.39)
DVU2500	ftsA	cell division protein FtsA	0.14 (0.15)	0.14 (0.13)	0 (0.04)
DVU2514	pyk	pyruvate kinase	-0.28 (-0.34)	0.56 (0.69)	-0.76 (-1.07)
DVU2515	-	HD domain protein	0.51 (0.57)	0.39 (0.46)	0.1 (0.21)

DVU2518	rplM	ribosomal protein L13	-0.02 (-0.04)	-0.17 (-0.27)	0.11 (0.21)
DVU2519	rpsI	ribosomal protein S9	0.31 (0.34)	0.14 (0.13)	0.16 (0.29)
DVU2529	pgk	phosphoglycerate kinase	-0.34 (-0.41)	-0.12 (-0.21)	-0.23 (-0.3)
DVU2531	rpe	ribulose-phosphate 3-epimerase	0.08 (0.07)	0.15 (0.14)	-0.06 (-0.04)
DVU2535	rplT	ribosomal protein L20	1.01 (1.15)	0.47 (0.58)	0.52 (0.82)
DVU2536	rpmI	ribosomal protein L35	0.35 (0.38)	-0.86 (-1.18)	1.04 (1.59)
DVU2537	infC	translation initiation factor IF-3	0.13 (0.14)	-0.91 (-1.25)	0.88 (1.35)
DVU2543	b0873	hybrid cluster protein	0.74 (0.83)	1.8 (2.33)	-0.85 (-1.2)
DVU2548	acpD	acyl carrier protein phosphodiesterase	0.96 (1.09)	1.04 (1.32)	0 (0.05)
DVU2552	gltX-2	glutamyl-tRNA synthetase	0.59 (0.66)	0.49 (0.59)	0.12 (0.23)
DVU2569	slyD	peptidyl-prolyl cis-trans isomerase, FKBP-type	0.45 (0.5)	0.16 (0.16)	0.27 (0.45)
DVU2572	feoA	ferrous iron transport protein A	0.39 (0.43)	0.5 (0.61)	-0.07 (-0.05)
DVU2577	-	DNA-binding response regulator, LuxR family	0.58 (0.65)	0.5 (0.61)	0.1 (0.2)
DVU2582	-	transcriptional regulator, TetR family	-0.04 (-0.06)	0.03 (0)	-0.08 (-0.07)
DVU2586	-	ABC transporter, ATP-binding protein	-0.98 (-1.15)	0.1 (0.08)	-1.03 (-1.47)
DVU2590	-	sensory box protein	-2.38 (-2.75)	-1.81 (-2.44)	-0.71 (-0.99)
DVU2614	-	hypothetical protein	-0.73 (-0.85)	-0.07 (-0.14)	-0.64 (-0.89)
DVU2643	htpG	heat shock protein HtpG	-0.84 (-0.99)	0.37 (0.45)	-1.13 (-1.61)
DVU2652	-	hypothetical protein	1.34 (1.52)	-0.39 (-0.56)	1.57 (2.36)
DVU2662	-	conserved hypothetical protein	0.73 (0.82)	0.47 (0.57)	0.26 (0.43)
DVU2669	-	conserved domain protein	0.98 (1.11)	0.29 (0.34)	0.65 (1)
DVU2717	-	hypothetical protein	-0.33 (-0.39)	-0.67 (-0.94)	0.23 (0.4)
DVU2754	qor	quinone oxidoreductase	-0.66 (-0.77)	0.68 (0.85)	-1.22 (-1.74)
DVU2755	-	conserved hypothetical protein	-0.83 (-0.97)	-0.14 (-0.24)	-0.67 (-0.93)
DVU2770	-	response regulator	0.06 (0.05)	-0.17 (-0.27)	0.18 (0.32)
DVU2771	-	conserved hypothetical protein	0.38 (0.42)	-0.48 (-0.68)	0.75 (1.15)
DVU2774	-	CBS domain protein/ACT domain protein	0.91 (1.03)	0.64 (0.8)	0.29 (0.48)
DVU2776	dsrC	dissimilatory sulfite reductase, gamma subunit	0.27 (0.3)	-0.64 (-0.89)	0.78 (1.21)
DVU2781	-	hypothetical protein	2.3 (2.63)	0.35 (0.42)	1.83 (2.75)
DVU2784	lldD	dehydrogenase, FMN-dependent family	0.02 (0)	0.14 (0.14)	-0.12 (-0.12)
DVU2787	-	conserved domain protein	-0.27 (-0.33)	-0.22 (-0.35)	-0.09 (-0.08)
DVU2837	-	conserved hypothetical protein	0.36 (0.4)	-0.04 (-0.1)	0.37 (0.59)
DVU2892	-	conserved hypothetical protein	0.62 (0.69)	0.44 (0.53)	0.2 (0.34)
DVU2897	-	conserved hypothetical protein	0.25 (0.27)	1.07 (1.37)	-0.68 (-0.95)
DVU2902	pyrC	dihydroorotase	0.14 (0.14)	0 (-0.06)	0.12 (0.23)
DVU2903	pcnB2	HD domain protein	-0.76 (-0.89)	0.52 (0.63)	-1.17 (-1.67)
DVU2912	rpmE	ribosomal protein L31	0.22 (0.24)	-0.58 (-0.82)	0.69 (1.07)
DVU2914	prfA	peptide chain release factor 1	-0.8 (-0.94)	-0.76 (-1.06)	-0.12 (-0.13)
DVU2920	tuf	translation elongation factor Tu	-0.7 (-0.83)	-0.48 (-0.68)	-0.26 (-0.33)
DVU2923	nusG	transcription antitermination protein NusG	0.02 (0.01)	0.23 (0.26)	-0.19 (-0.23)
DVU2924	rplK	ribosomal protein L11	0.33 (0.36)	-0.33 (-0.48)	0.57 (0.9)
DVU2925	rplA	ribosomal protein L1	0.32 (0.35)	-0.43 (-0.62)	0.65 (1.01)
DVU2926	rplJ	ribosomal protein L10	-0.35 (-0.42)	-0.97 (-1.33)	0.47 (0.74)
DVU2928	rpoB	DNA-directed RNA polymerase, beta subunit	-0.86 (-1.01)	0 (-0.05)	-0.82 (-1.15)
DVU2929	rpoC	DNA-directed RNA polymerase, beta prime subunit	-0.03 (-0.05)	0.8 (1.01)	-0.73 (-1.02)
DVU2930	-	hypothetical protein	#VALUE!	#VALUE!	#VALUE!
DVU2935	gpm	phosphoglycerate mutase	-0.55 (-0.65)	0.01 (-0.03)	-0.54 (-0.74)
DVU2938	-	conserved hypothetical protein	-0.55 (-0.66)	-0.49 (-0.7)	-0.12 (-0.12)
DVU2943	pyrE	orotate phosphoribosyltransferase	0.42 (0.46)	0.23 (0.25)	0.16 (0.29)
DVU2951	glnS	glutaminyl-tRNA synthetase	0.66 (0.74)	0.21 (0.23)	0.42 (0.67)
DVU2966	-	response regulator	-0.03 (-0.06)	0.92 (1.17)	-0.83 (-1.17)
DVU2973	hup	integration host factor, beta subunit	0.3 (0.32)	1.11 (1.42)	-0.7 (-0.98)
DVU2976	-	conserved hypothetical protein	0.54 (0.6)	0.78 (0.99)	-0.17 (-0.21)
DVU2978	-	hydrolase, haloacid dehalogenase-like family	0.13 (0.13)	-0.68 (-0.96)	0.68 (1.06)
DVU2981	leuA	2-isopropylmalate synthase	-0.15 (-0.19)	-0.25 (-0.38)	0.06 (0.14)
DVU2982	leuC	3-isopropylmalate dehydratase, large subunit, putative	1.78 (2.03)	1.49 (1.92)	0.38 (0.61)
DVU2985	leuB	3-isopropylmalate dehydrogenase	-0.72 (-0.85)	-0.52 (-0.74)	-0.25 (-0.32)
DVU2990	moeA	molybdopterin biosynthesis MoeA protein	-0.04 (-0.07)	0.52 (0.64)	-0.51 (-0.7)
DVU3005	rfbE	aminotransferase, DegT/DnrJ/EryC1/StrS family	-0.88 (-1.03)	-0.34 (-0.51)	-0.55 (-0.76)
DVU3025	por	pyruvate-ferredoxin oxidoreductase	-1.06 (-1.24)	-0.1 (-0.19)	-0.92 (-1.31)
DVU3027	glcD	glycolate oxidase, subunit GlcD	-0.94 (-1.1)	-1.8 (-2.43)	0.62 (0.97)

DVU3028	-	iron-sulfur cluster-binding protein	0.12 (0.12)	0.62 (0.76)	-0.42 (-0.57)
DVU3029	pta	phosphate acetyltransferase	-1.87 (-2.17)	-0.36 (-0.53)	-1.45 (-2.09)
DVU3030	ackA	acetate kinase	-0.54 (-0.64)	-0.36 (-0.53)	-0.21 (-0.26)
DVU3031	-	conserved hypothetical protein	-0.9 (-1.05)	0.24 (0.26)	-1.06 (-1.51)
DVU3032	-	conserved hypothetical protein	0.16 (0.17)	-0.95 (-1.3)	0.94 (1.44)
DVU3033	-	iron-sulfur cluster-binding protein	-0.05 (-0.07)	0.53 (0.65)	-0.51 (-0.71)
DVU3037	-	rhodanese-like domain protein	-0.33 (-0.4)	0 (-0.06)	-0.33 (-0.44)
DVU3041	-	cytochrome c553	1.1 (1.25)	1.16 (1.49)	0.02 (0.09)
DVU3048	asd	aspartate-semialdehyde dehydrogenase	0.35 (0.39)	0.66 (0.83)	-0.24 (-0.31)
DVU3053	-	conserved hypothetical protein	0.08 (0.07)	0.06 (0.03)	0 (0.05)
DVU3066	-	DNA-binding protein	-1.12 (-1.3)	-0.97 (-1.33)	-0.25 (-0.32)
DVU3080	-	transcriptional regulator, putative	0.02 (0)	0.07 (0.04)	-0.05 (-0.03)
DVU3084	-	transcriptional regulator, putative	-0.24 (-0.29)	-1.14 (-1.56)	0.72 (1.11)
DVU3094	rbr	rubrerythrin	0.9 (1.02)	-0.23 (-0.35)	1.02 (1.56)
DVU3103	-	tolB protein, putative	0.02 (0.01)	0.31 (0.37)	-0.25 (-0.32)
DVU3104	pal	peptidoglycan-associated lipoprotein, putative	0.38 (0.42)	0.91 (1.15)	-0.42 (-0.57)
DVU3117	-	hypothetical protein	1.16 (1.32)	1.13 (1.45)	0.11 (0.21)
DVU3118	-	conserved hypothetical protein	-0.39 (-0.46)	-0.26 (-0.4)	-0.16 (-0.18)
DVU3136	-	nitroreductase family protein	-0.42 (-0.5)	0.38 (0.46)	-0.73 (-1.02)
DVU3150	rpsA	ribosomal protein S1	0.27 (0.29)	-0.06 (-0.13)	0.29 (0.47)
DVU3154	-	HAM1 family protein	-0.24 (-0.3)	-0.5 (-0.71)	0.17 (0.31)
DVU3162	-	ABC transporter, periplasmic substrate-binding protein	-0.21 (-0.25)	-0.02 (-0.07)	-0.19 (-0.23)
DVU3167	-	heme biosynthesis protein, putative	0.22 (0.24)	-0.22 (-0.34)	0.38 (0.61)
DVU3168	hemL	glutamate-1-semialdehyde-2,1-aminomutase	-0.94 (-1.09)	-0.08 (-0.16)	-0.82 (-1.15)
DVU3172	-	hypothetical protein	1.21 (1.38)	0.95 (1.21)	0.31 (0.51)
DVU3173	-	hypothetical protein	0.57 (0.64)	0.02 (-0.01)	0.5 (0.79)
DVU3183	rbO	desulfoferrodoxin	-0.11 (-0.14)	-0.29 (-0.44)	0.13 (0.24)
DVU3185	roO	rubredoxin-oxygen oxidoreductase	0.43 (0.48)	-0.5 (-0.72)	0.82 (1.26)
DVU3187	hup-4	DNA-binding protein HU	0.96 (1.08)	0.63 (0.78)	0.34 (0.56)
DVU3190	-	hypothetical protein	0.1 (0.1)	-0.06 (-0.13)	0.13 (0.25)
DVU3193	-	DNA-binding domain, excisionase family	1.03 (1.17)	0.62 (0.77)	0.42 (0.68)
DVU3194	engA	GTP-binding protein EngA	-1.56 (-1.81)	0.11 (0.09)	-1.57 (-2.26)
DVU3197	ilvE	branched-chain amino acid aminotransferase	0.28 (0.31)	0.1 (0.08)	0.16 (0.28)
DVU3199	-	conserved hypothetical protein TIGR00103	0.19 (0.2)	-0.4 (-0.58)	0.5 (0.79)
DVU3204	purA	adenylosuccinate synthetase	-1.13 (-1.31)	-0.73 (-1.02)	-0.44 (-0.6)
DVU3206	purH	phosphoribosylaminoimidazolecarboxamide formyltransferase, putative	-0.05 (-0.08)	-0.46 (-0.66)	0.32 (0.53)
DVU3212	nox	pyridine nucleotide-disulfide oxidoreductase	-0.2 (-0.25)	-0.68 (-0.95)	0.37 (0.59)
DVU3228	cheY-3	chemotaxis protein CheY	-1.14 (-1.33)	-0.29 (-0.44)	-0.85 (-1.2)
DVU3235	purH	IMP cyclohydrolase, putative	0.2 (0.21)	0.02 (-0.02)	0.15 (0.27)
DVU3242	rpoZ	DNA-directed RNA polymerase, omega subunit	0.79 (0.9)	-0.11 (-0.2)	0.83 (1.27)
DVU3245	greA	transcription elongation factor GreA	1.52 (1.73)	0.64 (0.79)	0.86 (1.32)
DVU3253	-	phenylacetate-coenzyme A ligase, putative	0.49 (0.55)	0.97 (1.23)	-0.38 (-0.51)
DVU3263	frdB	fumarate reductase, iron-sulfur protein	-0.66 (-0.78)	0.34 (0.4)	-0.95 (-1.34)
DVU3271	cydA	cytochrome d ubiquinol oxidase, subunit I	-0.51 (-0.61)	-0.24 (-0.37)	-0.29 (-0.38)
DVU3272	-	TPR domain protein	-0.37 (-0.45)	-0.98 (-1.35)	0.47 (0.74)
DVU3278	htrA	peptidase/PDZ domain protein	-0.95 (-1.11)	0.05 (0.02)	-0.95 (-1.35)
DVU3281	-	hypothetical protein	-1.16 (-1.35)	-1.35 (-1.84)	0.04 (0.12)
DVU3282	-	ADP-ribosylglycohydrolase family protein	1.03 (1.17)	1.32 (1.7)	-0.17 (-0.2)
DVU3294	areC	aldehyde dehydrogenase NADP family protein	-0.18 (-0.23)	0.11 (0.09)	-0.28 (-0.36)
DVU3319	putA	proline dehydrogenase/delta-1-pyrroline-5-carboxylate dehydrogenase	-1.05 (-1.22)	0.05 (0.01)	-1.04 (-1.49)
DVU3347	-	pyruvate ferredoxin/flavodoxin oxidoreductase family protein	0.01 (0)	0.07 (0.05)	-0.06 (-0.03)
DVU3348	-	pyruvate ferredoxin/flavodoxin oxidoreductase, beta subunit, putative	-1.47 (-1.71)	-1.4 (-1.9)	-0.2 (-0.24)
DVU3349	-	pyruvate flavodoxin/ferredoxin oxidoreductase, thiamine diP-binding	0.01 (0)	0.28 (0.32)	-0.23 (-0.29)
DVU3352	-	lipoprotein, putative	0.08 (0.07)	0.16 (0.16)	-0.08 (-0.07)
DVU3360	parB	ParB family protein	1.42 (1.61)	0.82 (1.04)	0.61 (0.95)
DVU3371	metE	5-methyltetrahydropteroyltriglutamate-homocysteine S-methyltransferase	-2.83 (-3.27)	0.94 (1.19)	-3.5 (-5.1)
DVU3373	ilvD	dihydroxy-acid dehydratase	-1.51 (-1.76)	0.11 (0.09)	-1.53 (-2.2)

DVU3384	zraP	zinc resistance-associated protein	1.24 (1.41)	0.21 (0.22)	0.97 (1.48)
DVUA0002	-	-	0.08 (0.08)	-0.16 (-0.26)	0.2 (0.34)
DVUA0020	-	-	0.97 (1.1)	-1.13 (-1.55)	1.86 (2.79)
DVUA0074	-	-	0.05 (0.05)	-0.54 (-0.76)	0.5 (0.78)
DVUA0091	-	-	1.06 (1.21)	1.29 (1.66)	-0.11 (-0.12)
DVUA0108	-	-	1.87 (2.13)	1.17 (1.49)	0.74 (1.14)
DVUA0109	-	-	-0.02 (-0.04)	-0.16 (-0.27)	0.1 (0.2)
DVUA0111	-	-	0.02 (0.01)	-0.16 (-0.27)	0.16 (0.29)
DVUA0114	-	-	0.13 (0.13)	0.23 (0.25)	-0.08 (-0.07)
DVUA0115	-	-	1.39 (1.58)	1.81 (2.34)	-0.25 (-0.32)
DVUA0116	-	-	0.17 (0.18)	-0.11 (-0.19)	0.24 (0.41)
DVUA0126	-	-	-0.98 (-1.14)	-0.88 (-1.21)	-0.24 (-0.3)
DVUA0132	-	-	0.21 (0.23)	-0.19 (-0.31)	0.35 (0.57)
DVUA0146	-	-	-0.04 (-0.07)	-0.1 (-0.18)	0.03 (0.09)
