# Supplementary material for: Transcriptomic and proteomic analyses of Desulfovibrio vulgaris biofilms: Carbon and energy flow contribute to the distinct biofilm growth state
Source: BMC Genomics. 2012 Apr 16;13:138. doi: 10.1186/1471-2164-13-138 (PMC3431258; doi:10.1186/1471-2164-13-138)
Supplement: Additional file 6 — Expression and z values for 15 ribosomal protein genes. Expo1 and Expo2 represent exponential-phase cells and Stat1 through Stat4 represent stationary-phase cells. Blue and red colors denote level of expression for genes with a significant z score for up- and down-expression, respectively. [file 1471-2164-13-138-S6.pdf]

Additional file 6. Expression and z values for 15 ribosomal protein genes. Expo1 and Expo2 represent exponential-phase cells and Stat1 through Stat4 represent stationary-phase cells. Blue and red colors denote level of expression for genes with a significant z score for up- and down-expression, respectively.

ORF #	Gene	Expo1	Expo2	Transition	Stat1	Stat2	Stat3	Stat4	Biofilm
DVU1896	*rps*T	0.72<br>z = 1.19	0.09<br>z = 0.15	-4.26<br>z = -4.29	-3.86<br>z = -3.74	-3.75<br>z = -3.60	-4.15<br>z = -4.74	-3.22<br>z = -4.81	-0.83<br>z = -1.29
DVU0014	*inf*A-1	0.48<br>z = 0.84	0.35<br>z = 0.62	-3.97<br>z = -4.09	-3.63<br>z = -4.22	-3.39<br>z = -5.43	-3.23<br>z = -4.42	-3.55<br>z = -5.29	-2.55<br>z = -3.92
DVU1574	*rpl*Y	1.32<br>z = 2.29	1.20<br>z = 2.17	-3.12<br>z = -3.02	-2.11<br>z = -2.38	-2.38<br>z = -2.11	-2.19<br>z = -3.00	-2.36<br>z = -3.58	-1.65<br>z = -2.54
DVU0928	*rpm*A	0.83<br>z = 1.38	0.82<br>z 1.41	-2.93<br>z = -3.26	-2.76<br>z = -3.09	-2.85<br>z = -2.62	-2.10<br>z = -3.21	-2.36<br>z = -4.01	-1.01<br>z = -1.82
DVU2912	*rpm*E	0.59<br>z = 1.04	NA	-2.90<br>z = -3.70	-2.20<br>z = -2.82	-2.38<br>z = -3.94	-2.06<br>z = -3.68	-2.07<br>z = -2.74	NA
DVU0957	*rps*R	1.63<br>z = 2.62	1.08<br>z = 1.96	-2.82<br>z = -2.63	-1.72<br>z = -1.60	-2.05<br>z = -2.16	-1.90<br>z = -2.13	-2.32<br>z = -3.06	-0.88<br>z = -1.56
DVU1211	*rpm*B	-0.37<br>z = -0.64	0.12<br>z = 0.10	-2.43<br>z = -2.66	-1.28<br>z = -1.86	-1.31<br>z = -1.95	-1.02<br>z = -1.62	-1.42<br>z = -2.48	-0.16<br>z = -0.17
DVU2926	*rpl*J	1.77<br>z = 2.79	1.27<br>z = 2.04	-2.75<br>z = -3.27	-1.51<br>z = -1.78	-1.64<br>z = -1.76	-1.92<br>z = -2.07	-2.34<br>z = -3.04	NA
DVU1328	*rps*D	1.39<br>z = 2.33	0.85<br>z = 1.43	-2.43<br>z = -2.66	-1.28<br>z = -1.86	-1.31<br>z = -1.95	-1.02<br>z = -1.62	-1.42<br>z = -2.48	-1.17<br>z = -1.99
DVU1299	*rps*G	1.12<br>z = 1.89	0.68<br>z = 1.21	-2.40<br>z = -3.28	-1.40<br>z = -2.23	-1.23<br>z = -1.69	-1.29<br>z = -1.80	-1.18<br>z = -1.86	-1.69<br>z = -2.58
DVU0839	*rps*P	0.68<br>z = 1.07	0.59<br>z = 0.82	-2.39<br>z = -3.16	-1.93<br>z = -2.89	-2.04<br>z = -3.10	-2.03<br>z = -3.25	-2.12<br>z = -3.03	0.15<br>z = 0.28
DVU1303	*rpl*C	1.53<br>z = 2.60	0.89<br>z = 1.41	-2.34<br>z = -3.59	-1.15<br>z = -1.46	-1.65<br>z = -2.45	-1.13<br>z = -1.49	-1.81<br>z = -3.08	-0.75<br>z = -1.20
DVU1315	*rpl*E	1.08<br>z = 1.55	0.24<br>z 0.31	-2.31<br>z = -2.78	-2.48<br>z = -3.02	-1.23<br>z = -1.72	-1.24<br>z = -1.51	-1.75<br>z = -2.45	-0.07<br>z = -0.13
DVU1326	*rps*M	1.09<br>z = 1.84	0.71<br>z = 1.20	-2.30<br>z = -3.36	-1.67<br>z = -1.89	-1.58<br>z = -2.24	-1.35<br>z = -2.39	-2.03<br>z = -3.46	-0.89<br>z = -1.57
DVU1792	*rps*U	0.85	NA	-2.21	-1.84	-1.91	-2.26	-1.90	-0.73

		z = 1.43		z = -1.97	z = -2.28	z = -2.26	z = -2.79	z -2.27	z = -1.36
