# Supplementary material for: Transcriptomic and proteomic analyses of Desulfovibrio vulgaris biofilms: Carbon and energy flow contribute to the distinct biofilm growth state
Source: BMC Genomics. 2012 Apr 16;13:138. doi: 10.1186/1471-2164-13-138 (PMC3431258; doi:10.1186/1471-2164-13-138)
Supplement: Additional file 7 — Analysis of enriched extracellular proteins from biofilms. [file 1471-2164-13-138-S7.pdf]

Additional file 7. Analysis of enriched extracellular proteins from biofilms.

DVU	Gene Name	description	TM	Signal	Abundance (%)	# Pep Obs	# Pep Est.	Diff.
DVU1012	-	hemolysin-type calcium-binding repeat protein	0	0	0.328	67	8	59
DVU0547	-	high-affinity branched chain amino acid ABC transporter	0	Y	0.343	26	8	18
DVU0169	-	oligopeptide/dipeptide ABC transporter, periplasmic oligopeptide/dipeptide-binding protein	0	Y	0.532	27	13	14
DVU1918	hysA	periplasmic [NiFeSe] hydrogenase, large subunit, selenocysteine-containing	0	0	0.125	16	3	13
DVU0797	-	conserved hypothetical protein	0	Y	0.139	16	3	13
DVU0386	glnH	amino acid ABC transporter	0	Y	0.160	16	4	12
DVU1545	-	hemolysin-type calcium-binding repeat/calx-beta domain protein	0	0	0.000	12	0	12
Keratin	-	-	-	-	0.000	11	0	11
DVU0712	-	amino acid ABC transporter, periplasmic-binding protein	0	Y	0.428	21	10	11
DVU2013	-	hybrid cluster protein	0	0	0.518	23	13	10
DVU1937	-	phosphonate ABC transporter, periplasmic phosphonate-binding	0	Y	0.213	15	5	10
DVU1655	aspC4	aminotransferase, classes I and II	0	0	0.234	15	6	9
DVU3030	ackA	acetate kinase	0	0	0.653	25	16	9
DVU1064	aco	aconitate hydratase, putative	0	0	0.182	13	4	9
DVU1013	-	type I secretion outer membrane protein, TolC family	0	Y	0.105	11	3	8
DVU2985	leuB	3-isopropylmalate dehydrogenase	0	0	0.131	11	3	8
DVU2966	-	response regulator	0	0	0.059	9	1	8
DVU0319	-	NAD-dependent epimerase/dehydratase family protein	0	0	0.148	11	4	7
DVU0752	-	amino acid ABC transporter	0	Y	0.114	10	3	7
DVU1873	ppiB-2	peptidyl-prolyl cis-trans isomerase B	0	0	0.080	9	2	7
DVU1443	flgE	flagellar hook protein FlgE	0	Y	0.006	7	0	7
DVU0761	-	lipoprotein, putative	0	Y	0.048	8	1	7
DVU0095	potD-1	polyamine ABC transporter	0	Y	0.132	10	3	7
DVU1204	fabF	3-oxoacyl-acyl-carrier-protein synthase II	0	0	0.100	9	2	7
DVU3212	nox	pyridine nucleotide-disulfide oxidoreductase	0	Y	0.318	14	8	6
DVU2543	b0873	hybrid cluster protein	0	0	0.319	14	8	6
DVU0799	-	conserved hypothetical protein	0	Y	0.280	13	7	6
DVU1441	flaB1	flagellin	0	0	0.038	7	1	6
DVU0487	purE	phosphoribosylaminoimidazole carboxylase, catalytic subunit	0	0	0.086	8	2	6
DVU0979	b1200	DAK1 domain protein	0	0	0.335	14	8	6
DVU0863	-	flagellar hook-associated protein 2	0	0	0.005	6	0	6
DVU1817	cyf	cytochrome c-553	0	Y	0.097	8	2	6
DVU0671	-	conserved hypothetical protein	0	0	0.388	15	9	6
DVU1260	-	outer membrane protein P1, putative	0	Y	0.000	5	0	5
DVU1238	-	amino acid ABC transporter	0	Y	0.336	13	8	5

DVU0353	-	alcohol dehydrogenase, iron-containing	0	0	0.133	8	3	5
DVU0107	glnH	glutamine ABC transporter	0	Y	0.218	10	5	5
DVU2697	-	hypothetical protein	0	Y	0.015	5	0	5
DVU2477	pstS	phosphate ABC transporter	0	Y	0.015	5	0	5
DVU0341	kdsB	3-deoxy-D-manno-octulosonate cytidylyltransferase	0	0	0.100	7	2	5
DVU0745	-	ABC transporter	0	Y	0.019	5	0	5
DVU0002	dnaN	DNA polymerase III, beta subunit	0	0	0.106	7	3	4
DVU1427	-	response regulator	0	0	0.024	5	1	4
Trypsin	-	-	-	-	0.000	4	0	4
DVU0575	-	hypothetical protein	0	Y	0.000	4	0	4
DVU0966	-	amino acid ABC transporter	0	Y	0.168	8	4	4
DVU0978	-	ABC transporter	0	Y	0.010	4	0	4
DVU3041	-	cytochrome c553	0	Y	0.018	4	0	4
DVU3183	rbO	desulfoferrodoxin	0	0	0.143	7	3	4
DVU0296	-	peptidase, M24 family	0	0	0.061	5	1	4
DVU0595	-	conserved hypothetical protein	0	0	0.021	4	1	3
DVU3048	asd	aspartate-semialdehyde dehydrogenase	0	Y	0.192	8	5	3
DVU0460	-	predicted phospho-2-dehydro-3-deoxyheptonate aldolase	0	0	0.277	10	7	3
DVU0339	serA	D-isomer specific 2-hydroxyacid dehydrogenase family protein	0	0	0.114	6	3	3
DVU2201	b3011	alcohol dehydrogenase, iron-containing	0	0	0.322	11	8	3
DVU2318	rbr2	rubrerythrin, putative	0	0	0.074	5	2	3
DVU0439	-	YCII-related domain protein	0	0	0.000	3	0	3
DVU3171	-	cytochrome c3	0	Y	0.000	3	0	3
DVU2680	fld	flavodoxin, iron-repressed	0	0	0.000	3	0	3
DVU1619	gpmA	phosphoglycerate mutase, 2,3-bisphosphoglycerate-independent	0	0	0.000	3	0	3
DVU1423	lpdA	2-oxoglutarate dehydrogenase, E3 component, lipoamide dehydrogenase	0	0	0.000	3	0	3
DVU0477	icd	isocitrate dehydrogenase, NADP-dependent	0	0	0.127	6	3	3
DVU3042	-	lipoprotein, putative	0	Y	0.005	3	0	3
DVU2496	-	lipoprotein, putative	0	Y	0.007	3	0	3
DVU2942	purB	adenylosuccinate lyase	0	0	0.090	5	2	3
DVU1833	ppsA	phosphoenolpyruvate synthase, putative	0	0	0.629	18	15	3
DVU2744	-	high-affinity branched-chain amino acid ABC transporter	0	Y	0.012	3	0	3
DVU3118	-	conserved hypothetical protein	0	0	0.055	4	1	3
DVU1317	rpsH	ribosomal protein S8	0	0	0.180	7	4	3
DVU2449	metK	S-adenosylmethionine synthetase	0	0	0.469	14	11	3
DVU1586	ccmG	thioredoxin family protein	0	Y	0.015	3	0	3
DVU0766	-	transporter, putative	0	Y	0.022	3	1	2
DVU1621	-	hypothetical protein	0	0	0.023	3	1	2
DVU2077	-	conserved hypothetical protein	0	0	0.232	8	6	2
DVU0675	fliY	amino acid ABC transporter, periplasmic amino acid-binding protein	0	Y	0.115	5	3	2
DVU1612	-	ACT domain protein	0	0	0.076	4	2	2
DVU0660	-	phosphoesterase, putative	0	0	0.035	3	1	2
DVU0284	ppiB-1	peptidyl-prolyl cis-trans isomerase B	0	0	0.036	3	1	2
DVU2569	slyD	peptidyl-prolyl cis-trans isomerase, FKBP-type	0	0	0.119	5	3	2

DVU2250	-	AMP-binding protein	0	0	0.163	6	4	2
DVU0277	-	transcriptional regulator, AraC family	0	0	0.000	2	0	2
DVU0074	-	polysaccharide biosynthesis domain protein	0	0	0.000	2	0	2
DVU1205	acpP	acyl carrier protein	0	0	0.042	3	1	2
DVU1319	rplR	ribosomal protein L18	0	0	0.044	3	1	2
DVU0255	-	hypothetical protein	1	0	0.003	2	0	2
DVU1933	-	peptidase, PfpI family	0	0	0.003	2	0	2
DVU1307	rpsS	ribosomal protein S19	0	0	0.046	3	1	2
DVU2333	ndk	nucleoside diphosphate kinase	0	0	0.005	2	0	2
DVU1684	gcvT	glycine cleavage system T protein	0	0	0.047	3	1	2
DVU0305	fd II	ferredoxin II	0	0	0.006	2	0	2
DVU3228	cheY-3	chemotaxis protein CheY	0	0	0.006	2	0	2
DVU2223	-	hypothetical protein	0	0	0.006	2	0	2
DVU2112	-	hypothetical protein	0	0	0.006	2	0	2
DVU1878	ltaE	threonine aldolase, low-specificity	0	0	0.048	3	1	2
DVU1198	ribH	riboflavin synthase, beta subunit	0	0	0.173	6	4	2
DVU2428	-	lipoprotein, putative	0	Y	0.011	2	0	2
DVU0488	purD	phosphoribosylamine--glycine ligase	0	0	0.180	6	4	2
DVU2444	flaB3	flagellin	0	0	0.058	3	1	2
DVU0607	ahcY	adenosylhomocysteinase	0	0	0.678	18	16	2
DVU0352	lpsC	aminotransferase, DegT/DnrJ/EryC1/StrS family	0	0	0.024	2	1	1
DVU0278	-	glyoxalase family protein	0	Y	0.025	2	1	1
DVU0401	-	hypothetical protein	0	0	0.030	2	1	1
DVU3168	hemL	glutamate-1-semialdehyde-2,1-aminomutase	0	0	0.114	4	3	1
DVU1980	-	hypothetical protein	1	0	0.034	2	1	1
DVU1580	rpiB	ribose 5-phosphate isomerase, putative	0	0	0.036	2	1	1
DVU1308	rplV	ribosomal protein L22	0	0	0.037	2	1	1
DVU1298	rpsL	ribosomal protein S12	0	0	0.037	2	1	1
DVU2342	-	amino acid ABC transporter, periplasmic amino acid-binding protein	0	Y	0.162	5	4	1
DVU1579	cysS	cysteinyl-tRNA synthetase	0	0	0.120	4	3	1
DVU3049	-	hemerythrin family protein	0	0	0.080	3	2	1
DVU2530	tkt	transketolase	0	0	0.122	4	3	1
DVU0835	rplS	ribosomal protein L19	0	0	0.049	2	1	1
DVU1207	fabH	3-oxoacyl-acyl-carrier-protein synthase III	0	0	0.049	2	1	1
DVU2144	gap-2	glyceraldehyde 3-phosphate dehydrogenase	0	0	0.092	3	2	1
DVU0007	asnS	asparaginyl-tRNA synthetase	0	0	0.216	6	5	1
DVU1677	tpiA	triosephosphate isomerase	0	0	0.093	3	2	1
DVU0138	-	response regulator	0	0	0.094	3	2	1
DVU2529	pgk	phosphoglycerate kinase	0	0	0.179	5	4	1
DVU0839	rpsP	ribosomal protein S16	0	0	0.056	2	1	1
DVU1311	rpmC	ribosomal protein L29	0	0	0.098	3	2	1
DVU1827	-	acetylornithine deacetylase/succinyl-diaminopimelate desuccinylase	0	0	0.058	2	1	1
DVU1924	hypC	hydrogenase assembly chaperone hypC/hupF	0	0	0.063	2	2	0
DVU3392	glnA	glutamine synthetase, type I	0	0	0.147	4	4	0
DVU2784	lldD	dehydrogenase, FMN-dependent family	0	0	0.194	5	5	0
DVU1839	trx	thioredoxin	0	0	0.279	7	7	0

DVU2108	-	MTH1175-like domain family	0	0	0.202	5	5	0
DVU0241	-	MTH1175-like domain family	0	0	0.085	2	2	0
DVU0418	lys1	saccharopine dehydrogenase	0	Y	0.087	2	2	0
DVU1539	glpX	fructose-1,6-bisphosphatase, class II	0	0	0.129	3	3	0
DVU3384	zraP	zinc resistance-associated protein	0	Y	0.336	8	8	0
DVU2983	leuD	3-isopropylmalate dehydratase, small subunit	0	0	0.131	3	3	0
DVU2347	argD	acetylornithine aminotransferase	0	0	0.258	6	6	0
DVU0795	purC	phosphoribosylaminoimidazole-succinocarboxamide synthase	0	0	0.135	3	3	0
DVU1568	ftn	ferritin	0	0	0.302	7	7	0
DVU1904	cheW-2	chemotaxis protein CheW	0	0	0.179	4	4	0
DVU1228	tpX	thiol peroxidase	0	0	0.221	5	5	0
DVU0841	-	aspartate aminotransferase, putative	0	0	0.103	2	2	0
DVU2519	rpsI	ribosomal protein S9	0	0	0.105	2	3	-1
DVU2982	leuC	3-isopropylmalate dehydratase, large subunit, putative	0	0	0.107	2	3	-1
DVU1320	rpsE	ribosomal protein S5	0	0	0.109	2	3	-1
DVU1302	rpsJ	ribosomal protein S10	0	0	0.151	3	4	-1
DVU3185	roO	rubredoxin-oxygen oxidoreductase	0	0	0.399	9	10	-1
DVU2776	dsrC	dissimilatory sulfite reductase, gamma subunit	0	0	0.321	7	8	-1
DVU1299	rpsG	ribosomal protein S7	0	0	0.197	4	5	-1
DVU1248	argS	arginyl-tRNA synthetase	0	0	0.198	4	5	-1
DVU1569	porA	pyruvate ferredoxin oxidoreductase, alpha subunit	0	0	0.117	2	3	-1
DVU0734	cysG-1	uroporphyrinogen III synthase/methyltransferase	0	0	0.127	2	3	-1
DVU2014	fprA-1	metallo-beta-lactamase family protein	0	0	0.170	3	4	-1
DVU1300	fusA-1	translation elongation factor G	0	0	0.753	17	18	-1
DVU0927	rplU	ribosomal protein L21	0	0	0.135	2	3	-1
DVU0849	QmoB	Quinone-interacting membrane-bound oxidoreductase	0	0	0.591	13	14	-1
DVU0995	-	ThiJ/PfpI family protein	0	0	0.137	2	3	-1
DVU3104	pal	peptidoglycan-associated lipoprotein, putative	0	Y	0.144	2	3	-1
DVU1932	adk	adenylate kinase	0	0	0.521	11	13	-2
DVU0019	ngr	nigerythrin	0	0	0.154	2	4	-2
DVU1257	-	RNA-binding protein	0	0	0.280	5	7	-2
DVU0142	trpS	tryptophanyl-tRNA synthetase	0	0	0.163	2	4	-2
DVU1179	aor	aldehyde:ferredoxin oxidoreductase, tungsten-containing	0	0	0.620	13	15	-2
DVU3187	hup-4	DNA-binding protein HU	0	0	0.175	2	4	-2
DVU2990	moeA	molybdopterin biosynthesis MoeA protein	0	0	0.177	2	4	-2
DVU0958	rplI	ribosomal protein L9	0	0	0.222	3	5	-2
DVU3373	ilvD	dihydroxy-acid dehydratase	0	Y	0.181	2	4	-2
DVU1043	guaA	GMP synthase	0	0	0.226	3	5	-2
DVU0848	QmoA	Quinone-interacting membrane-bound oxidoreductase	0	Y	0.227	3	5	-2
DVU3204	purA	adenylosuccinate synthetase	0	0	0.249	3	6	-3
DVU2215	-	RNA-binding protein	0	0	0.255	3	6	-3
DVU0650	-	chelatase, putative	0	Y	0.265	3	6	-3
DVU0403	dvsB	dissimilatory sulfite reductase beta subunit	0	0	0.956	19	23	-4
DVU0322	eno	enolase	0	0	0.266	2	6	-4

DVU1977	groES	chaperonin, 10 kDa	0	0	0.601	10	15	-5
DVU0956	rpsF	ribosomal protein S6	0	Y	0.314	3	8	-5
DVU0186	-	conserved hypothetical protein	0	0	0.336	3	8	-5
DVU1203	glyA	serine hydroxymethyltransferase	0	0	0.381	4	9	-5
DVU1378	ilvC	ketol-acid reductoisomerase	0	0	0.641	10	16	-6
DVU0404	dsrD	dissimilatory sulfite reductase D	0	0	0.331	2	8	-6
DVU2297	-	glycine/betaine/L-proline ABC transporter	0	Y	0.403	3	10	-7
DVU1334	tig	trigger factor	0	0	0.633	8	15	-7
DVU1328	rpsD	ribosomal protein S4	0	0	0.386	2	9	-7
DVU1636	ppaC	inorganic pyrophosphatase, manganese-dependent	0	0	1.370	24	33	-9
DVU1976	groEL	chaperonin, 60 kDa	0	0	1.283	20	31	-11
DVU3150	rpsA	ribosomal protein S1	0	0	0.615	3	15	-12
DVU2920	tuf	translation elongation factor Tu	0	0	0.936	4	23	-19
DVU0402	dsrA	dissimilatory sulfite reductase alpha subunit	0	0	1.686	19	41	-22
DVU3025	por	pyruvate-ferredoxin	0	0	1.869	18	45	-27
DVU2109	mrp	MTH1175-like domain family	0	0	1.464	7	35	-28
DVU1295	sat	sulfate adenylyltransferase	0	0	2.688	21	65	-44
DVU2405	-	alcohol dehydrogenase, iron-containing	0	0	2.061	2	50	-48
DVU0847	ApsA	adenylyl-sulphate reductase, alpha subunit	0	0	3.811	39	92	-53
